# Supplementary figures and images for: Nuclear rupture in confined cell migration triggers nuclear actin polymerization to limit chromatin leakage (part 1 of 2)
Source: EMBO J. 2025 Sep 22;44(21):6112–36. doi: 10.1038/s44318-025-00566-2 (PMC12583611; doi:10.1038/s44318-025-00566-2)

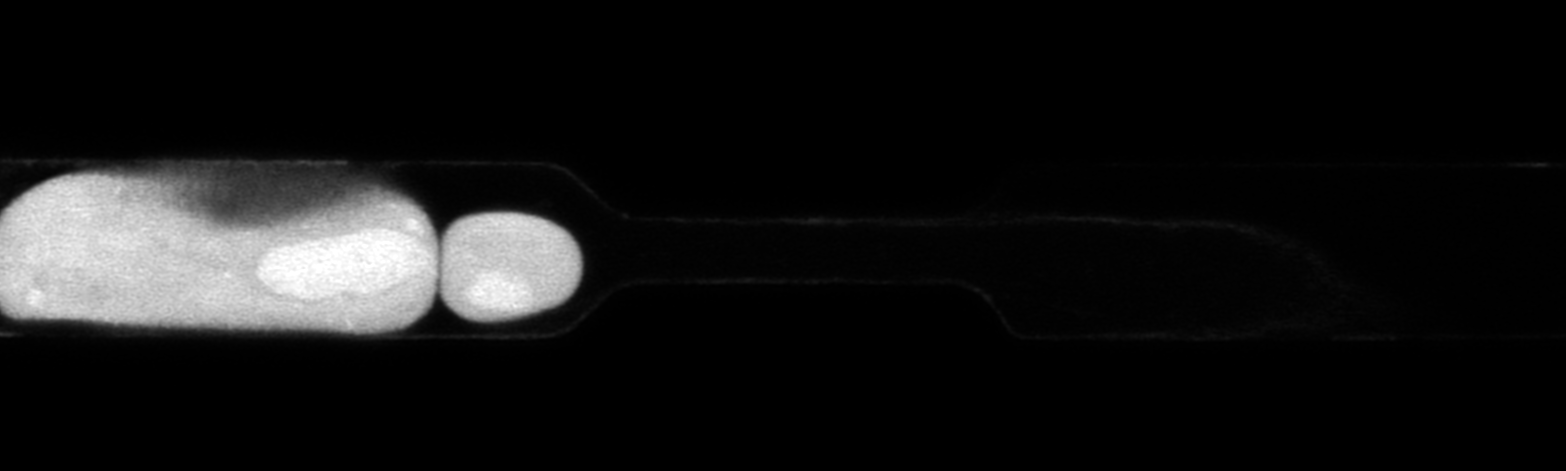

Supplement: Supplementary file 15 — Source data Fig. 1 [file 44318_2025_566_MOESM15_ESM.zip › Fig 1/Fig 1A/3um_0 min.tif]

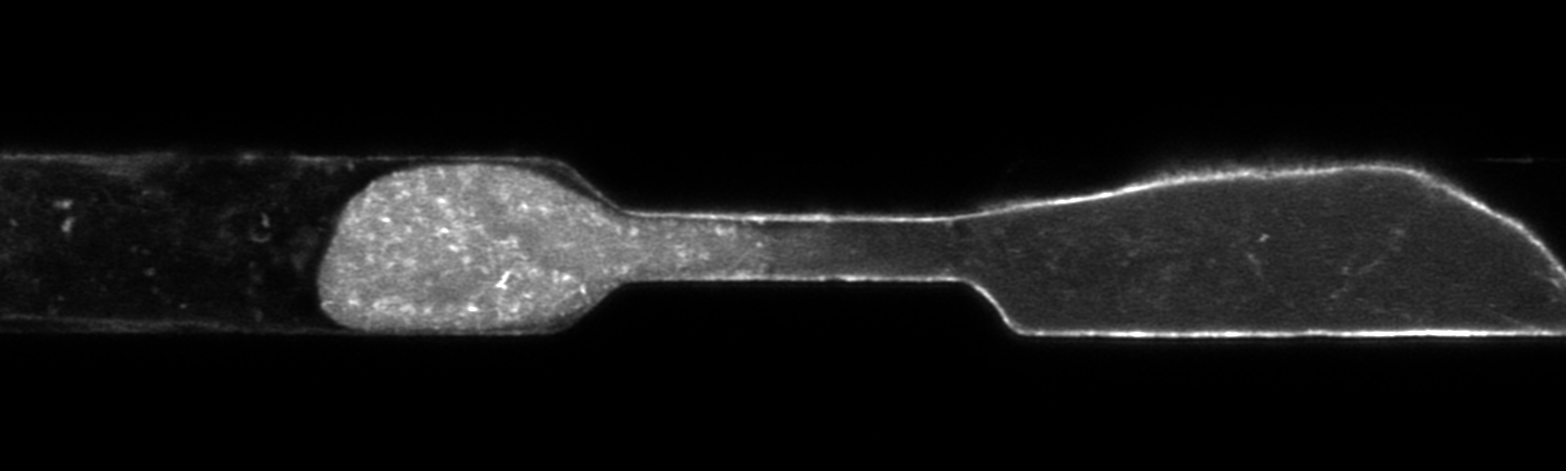

Supplement: Supplementary file 15 — Source data Fig. 1 [file 44318_2025_566_MOESM15_ESM.zip › Fig 1/Fig 1A/3um_10 min.tif]

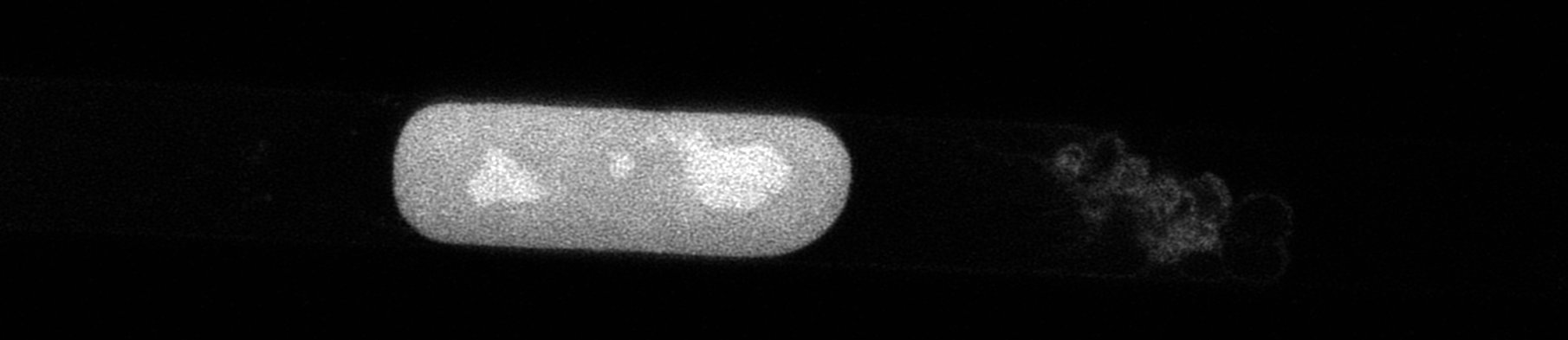

Supplement: Supplementary file 15 — Source data Fig. 1 [file 44318_2025_566_MOESM15_ESM.zip › Fig 1/Fig 1A/8um_0 min.tif]

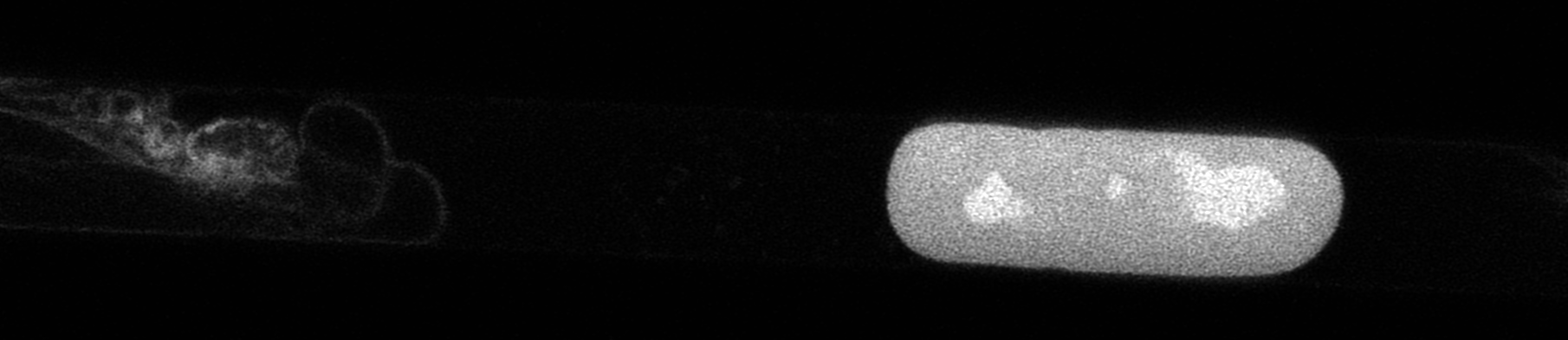

Supplement: Supplementary file 15 — Source data Fig. 1 [file 44318_2025_566_MOESM15_ESM.zip › Fig 1/Fig 1A/8um_20min.tif]

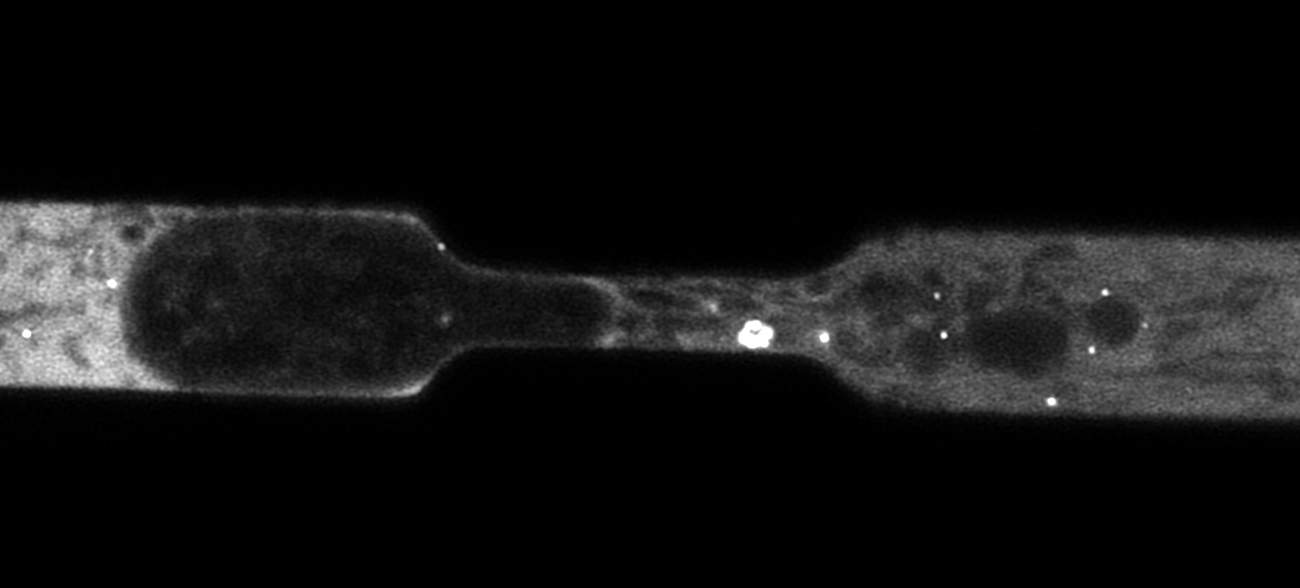

Supplement: Supplementary file 15 — Source data Fig. 1 [file 44318_2025_566_MOESM15_ESM.zip › Fig 1/Fig 1F/icGAS_0min.tif]

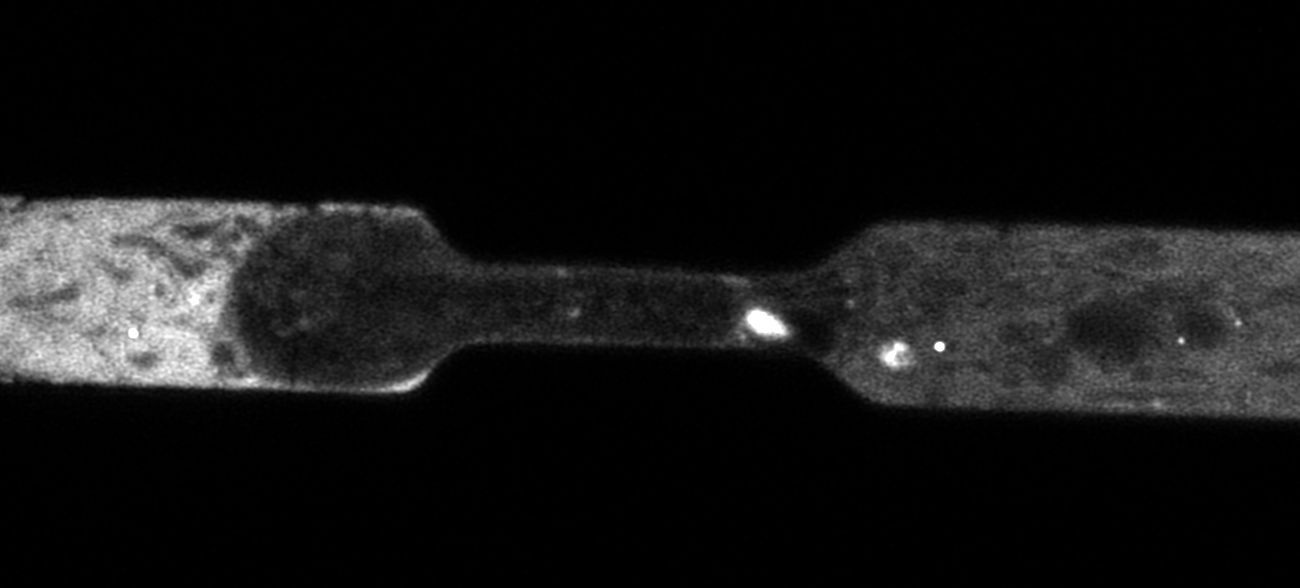

Supplement: Supplementary file 15 — Source data Fig. 1 [file 44318_2025_566_MOESM15_ESM.zip › Fig 1/Fig 1F/icGAS_5min.tif]

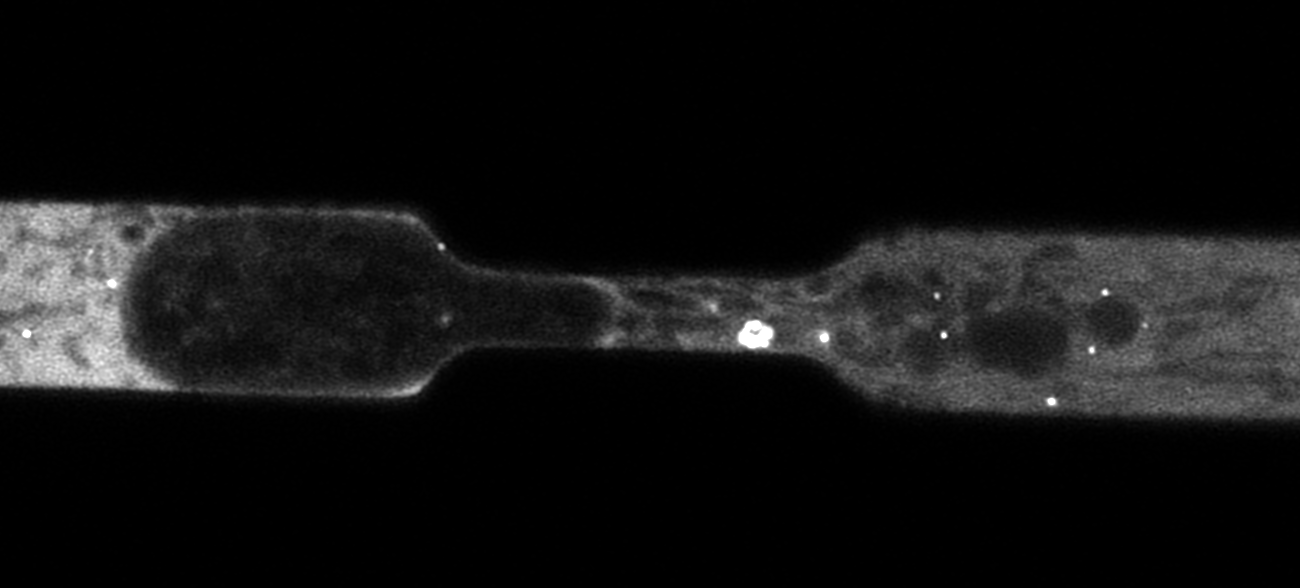

Supplement: Supplementary file 15 — Source data Fig. 1 [file 44318_2025_566_MOESM15_ESM.zip › Fig 1/Fig 1F/Merge_0min.tif]

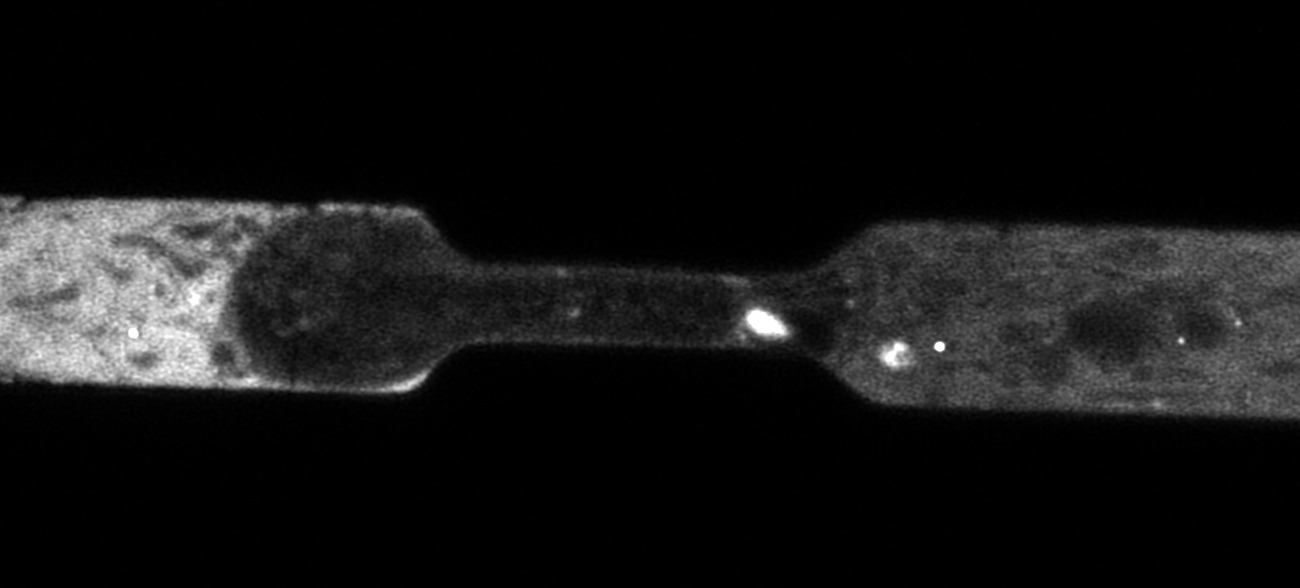

Supplement: Supplementary file 15 — Source data Fig. 1 [file 44318_2025_566_MOESM15_ESM.zip › Fig 1/Fig 1F/Merge_5min.tif]

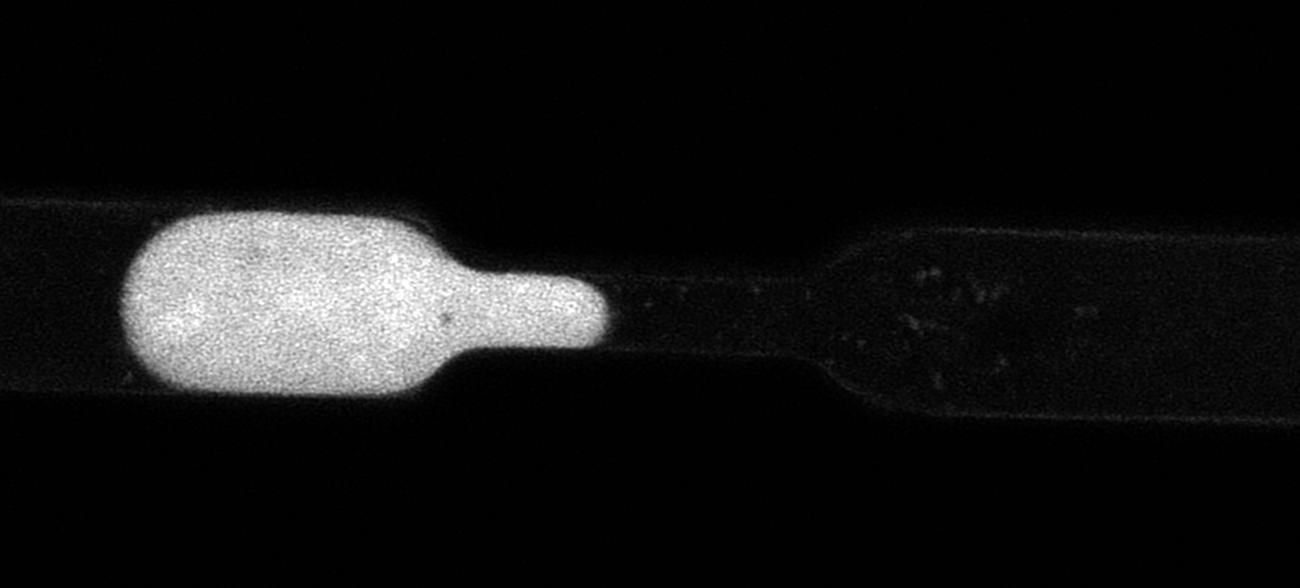

Supplement: Supplementary file 15 — Source data Fig. 1 [file 44318_2025_566_MOESM15_ESM.zip › Fig 1/Fig 1F/nAC-GFP_0min.tif]

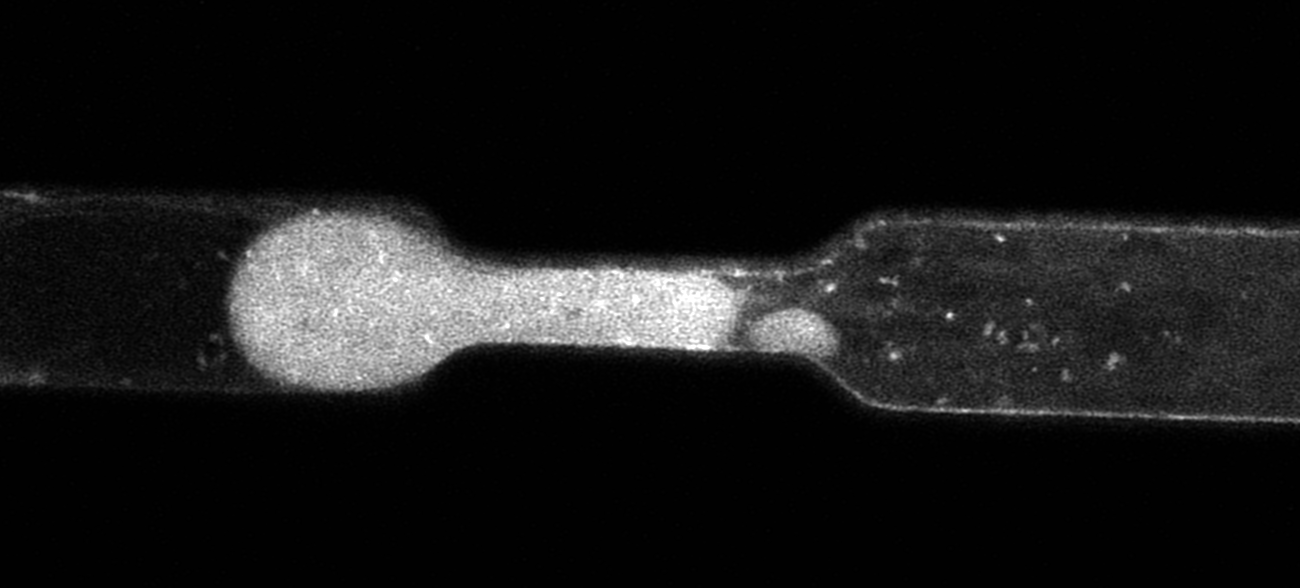

Supplement: Supplementary file 15 — Source data Fig. 1 [file 44318_2025_566_MOESM15_ESM.zip › Fig 1/Fig 1F/nAC-GFP_5min.tif]

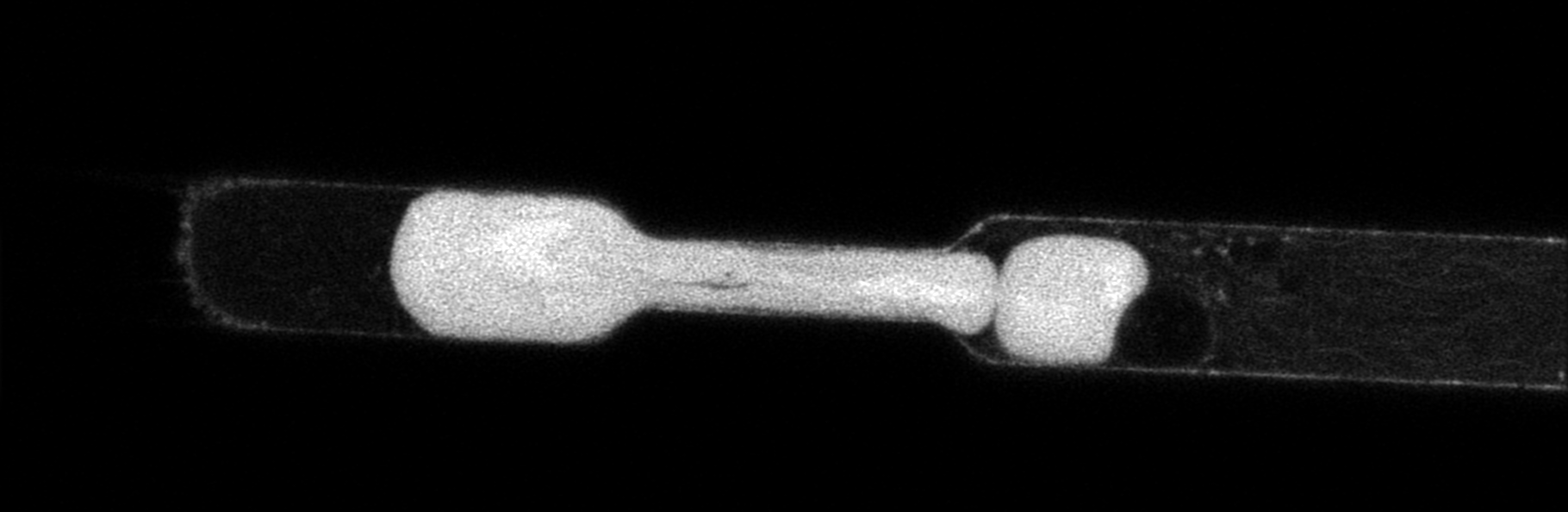

Supplement: Supplementary file 15 — Source data Fig. 1 [file 44318_2025_566_MOESM15_ESM.zip › Fig 1/Fig 1G/0s.tif]

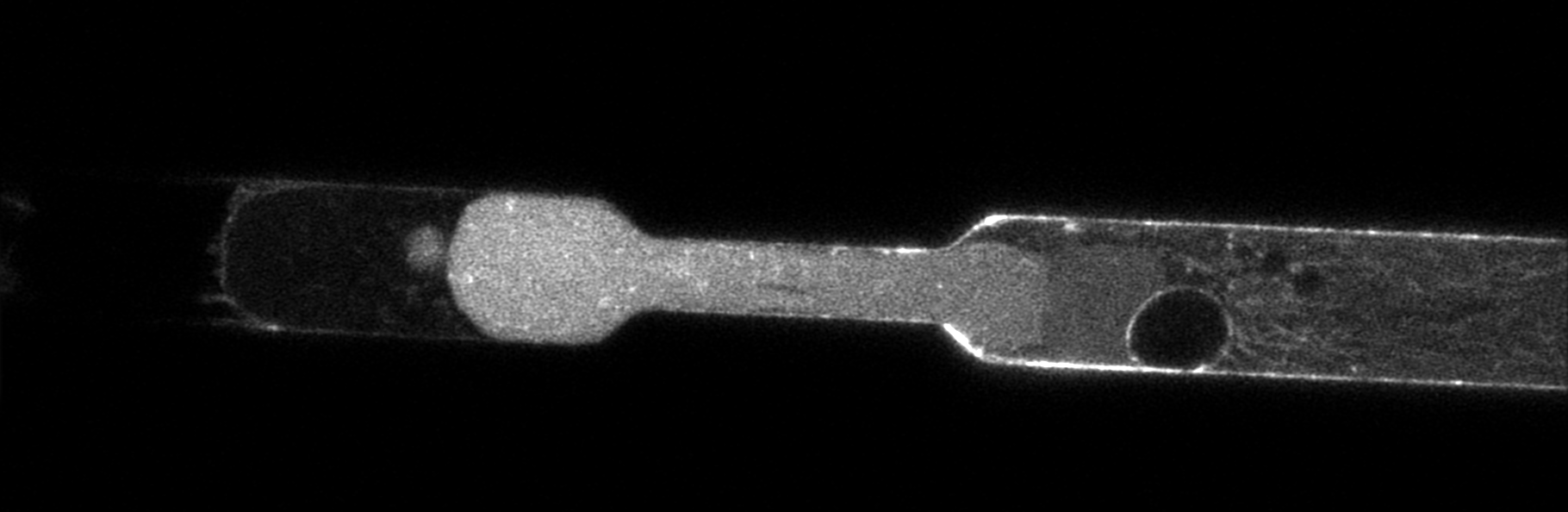

Supplement: Supplementary file 15 — Source data Fig. 1 [file 44318_2025_566_MOESM15_ESM.zip › Fig 1/Fig 1G/156s.tif]

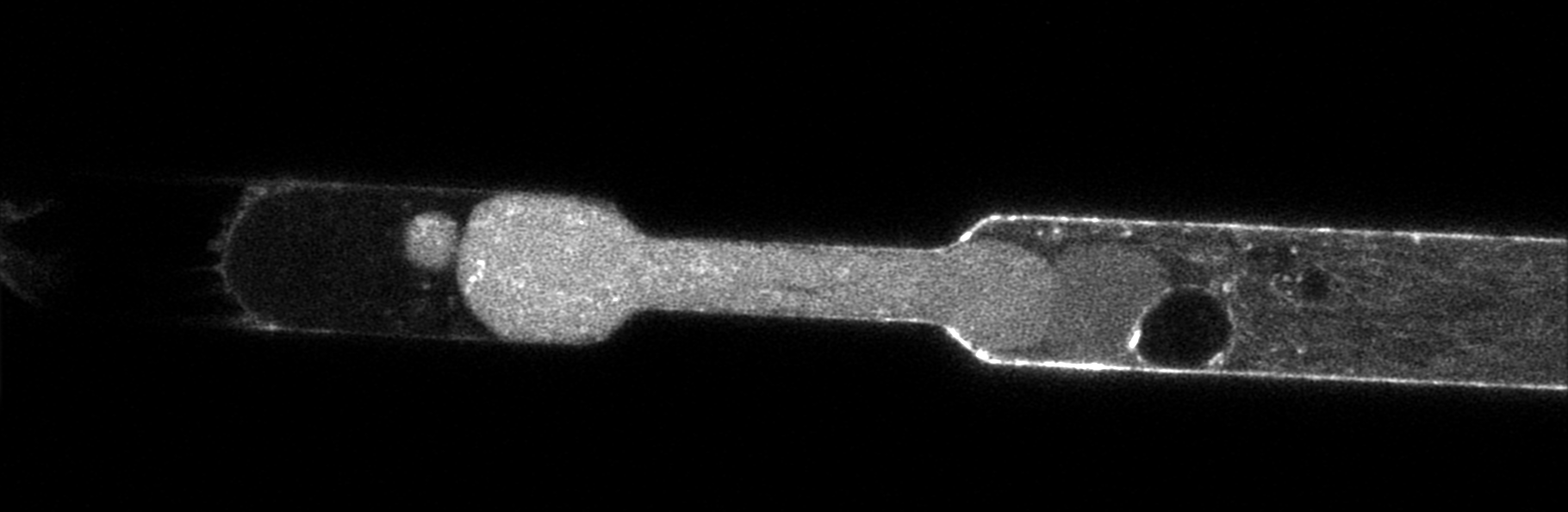

Supplement: Supplementary file 15 — Source data Fig. 1 [file 44318_2025_566_MOESM15_ESM.zip › Fig 1/Fig 1G/216s.tif]

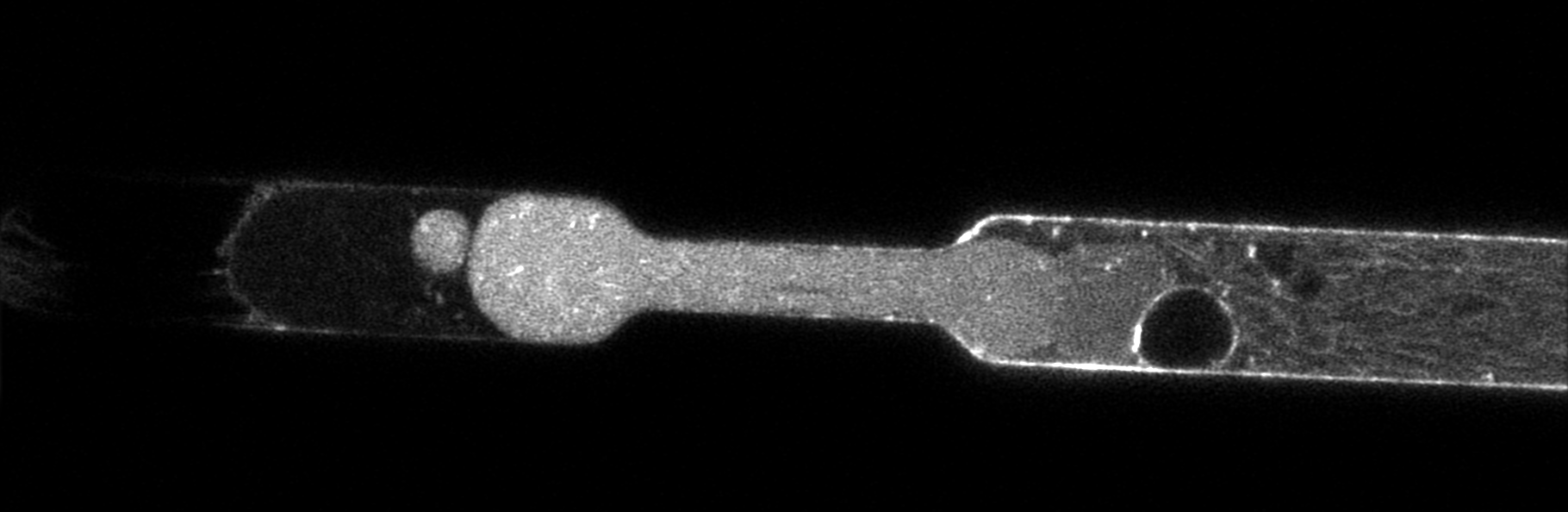

Supplement: Supplementary file 15 — Source data Fig. 1 [file 44318_2025_566_MOESM15_ESM.zip › Fig 1/Fig 1G/240s.tif]

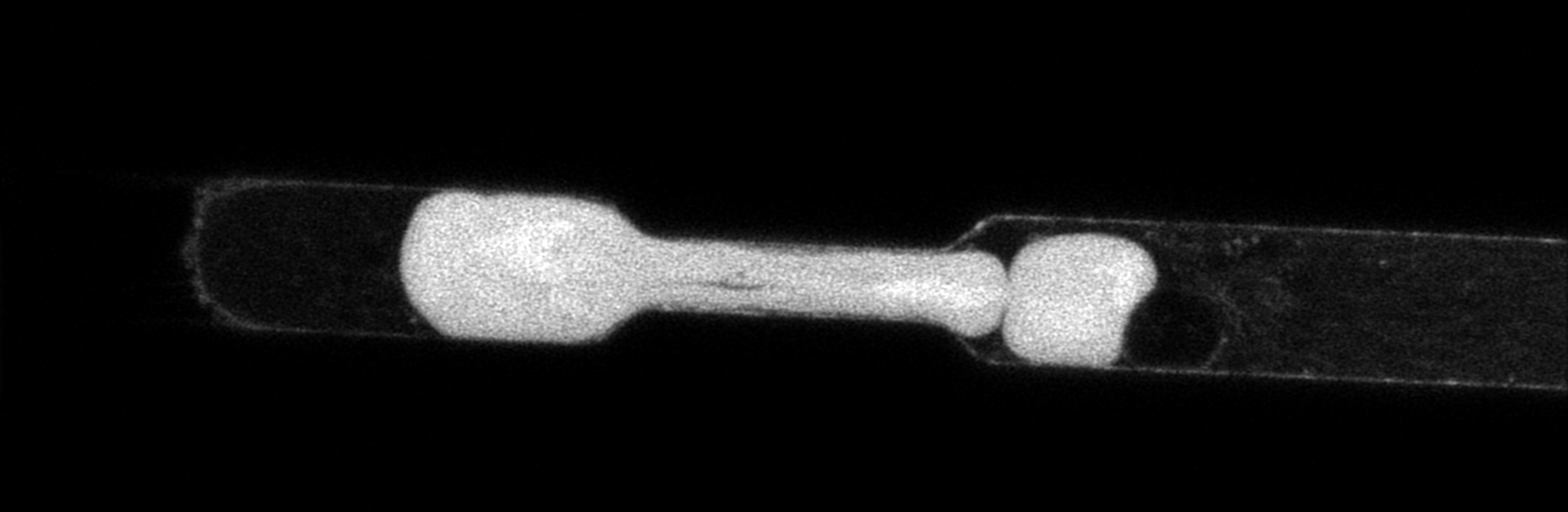

Supplement: Supplementary file 15 — Source data Fig. 1 [file 44318_2025_566_MOESM15_ESM.zip › Fig 1/Fig 1G/24s.tif]

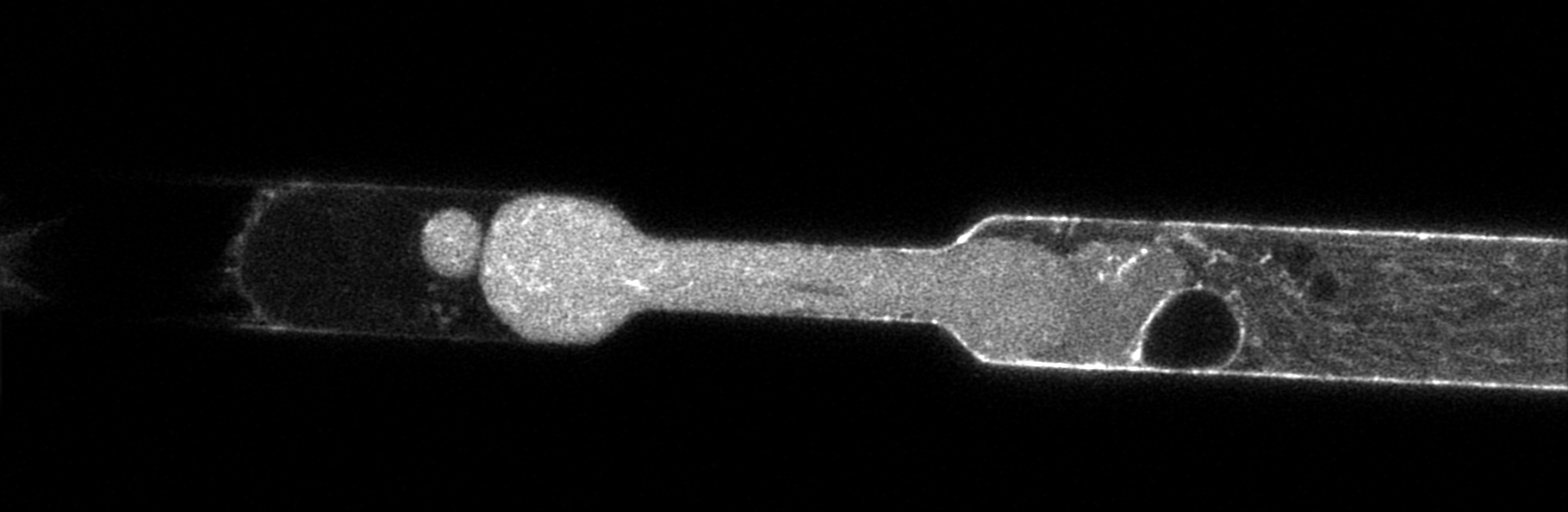

Supplement: Supplementary file 15 — Source data Fig. 1 [file 44318_2025_566_MOESM15_ESM.zip › Fig 1/Fig 1G/264s.tif]

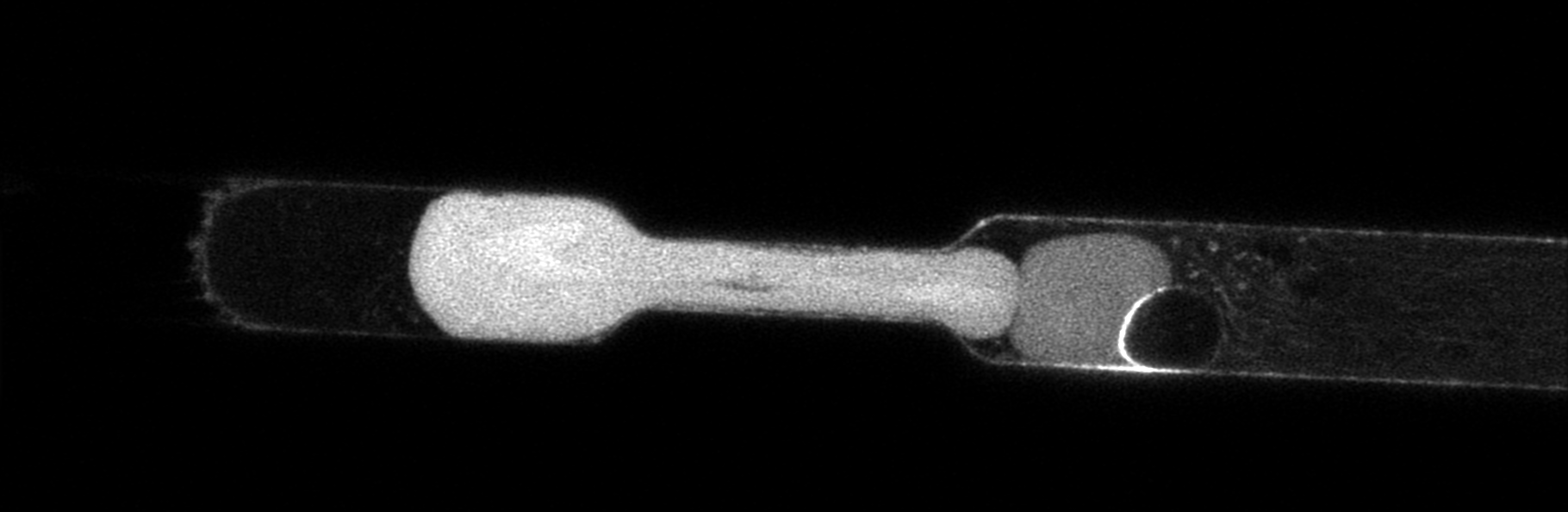

Supplement: Supplementary file 15 — Source data Fig. 1 [file 44318_2025_566_MOESM15_ESM.zip › Fig 1/Fig 1G/48s.tif]

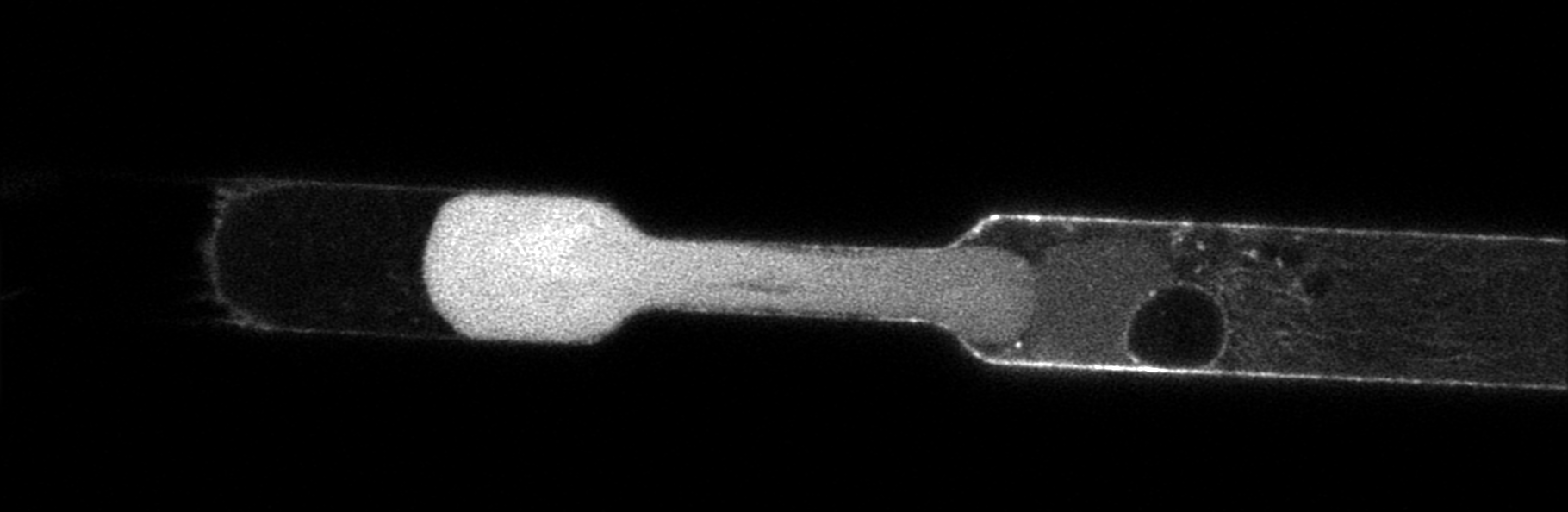

Supplement: Supplementary file 15 — Source data Fig. 1 [file 44318_2025_566_MOESM15_ESM.zip › Fig 1/Fig 1G/72s.tif]

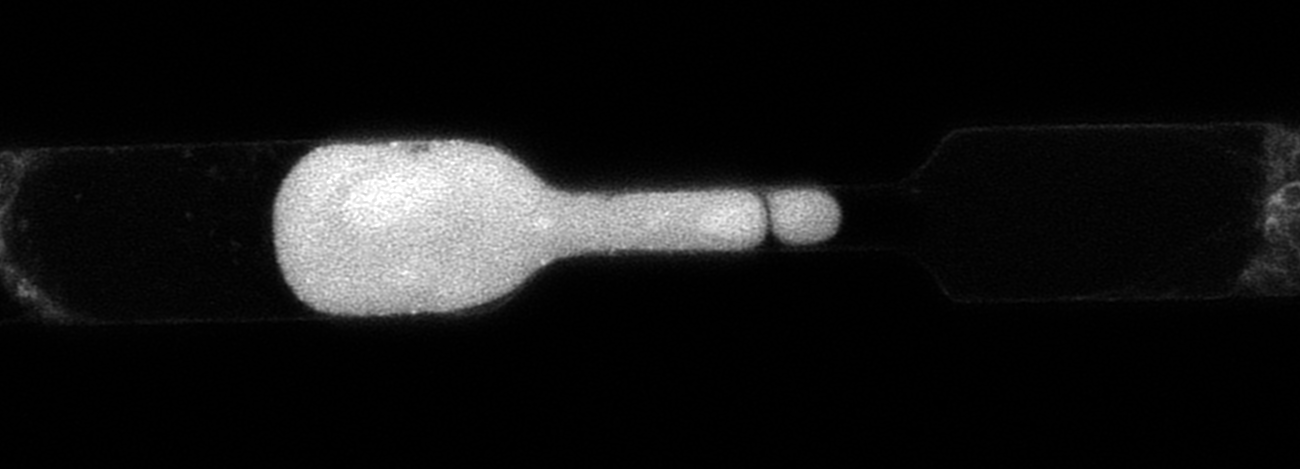

Supplement: Supplementary file 16 — Source data Fig. 2 [file 44318_2025_566_MOESM16_ESM.zip › Fig 2/Fig 2A/siCtrl_Before NE rupture.tif]

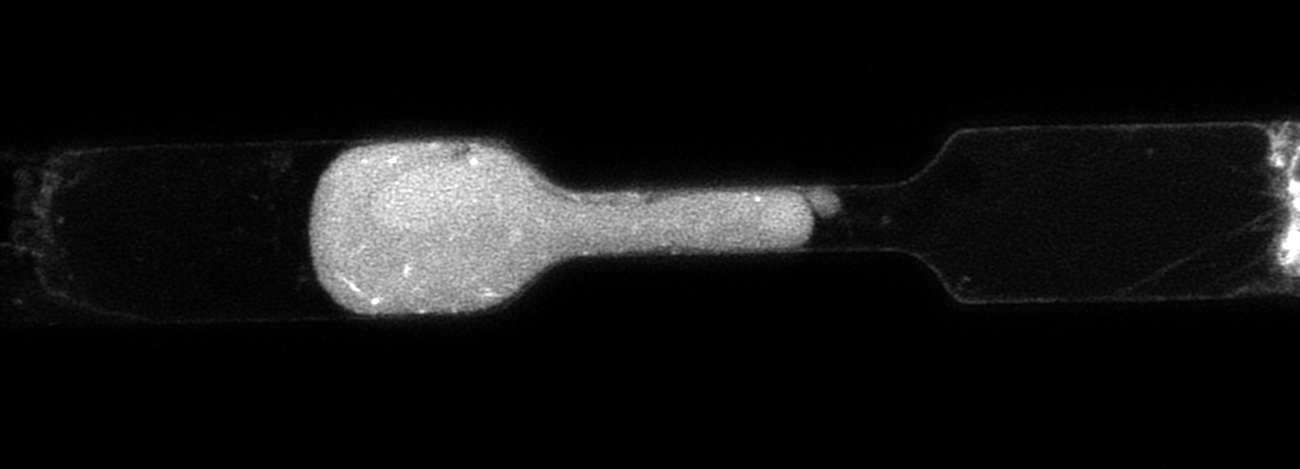

Supplement: Supplementary file 16 — Source data Fig. 2 [file 44318_2025_566_MOESM16_ESM.zip › Fig 2/Fig 2A/siCtrl_NE rupture.tif]

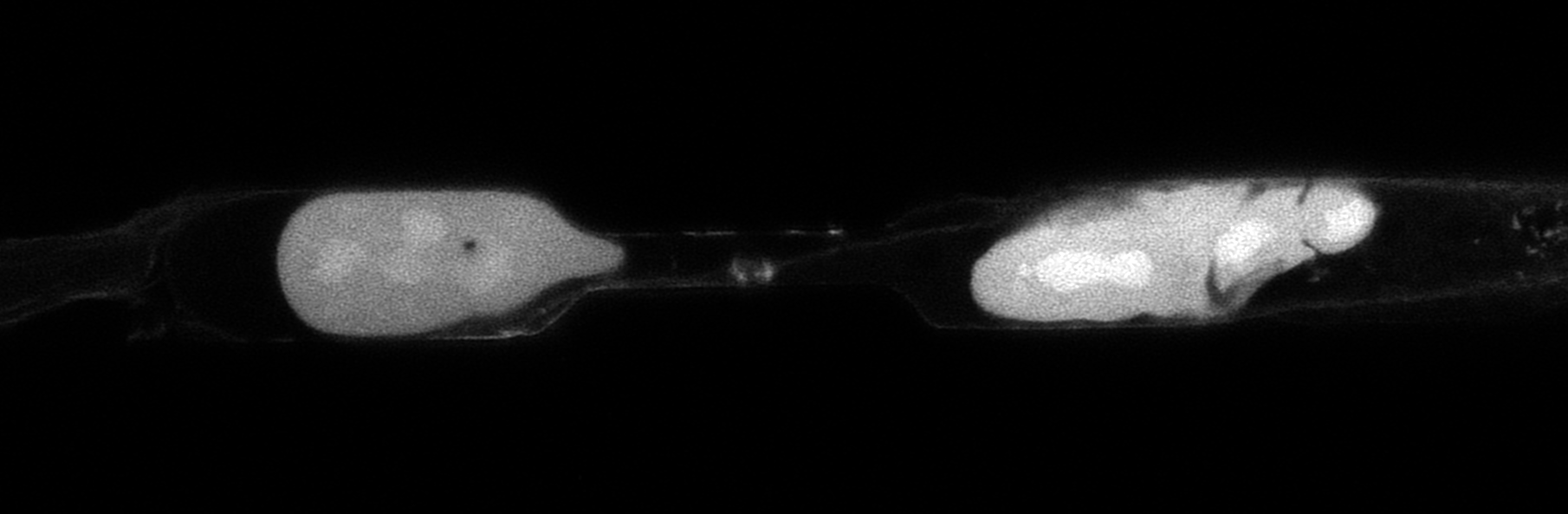

Supplement: Supplementary file 16 — Source data Fig. 2 [file 44318_2025_566_MOESM16_ESM.zip › Fig 2/Fig 2A/siDIAPH1_3_Before NE rupture.tif]

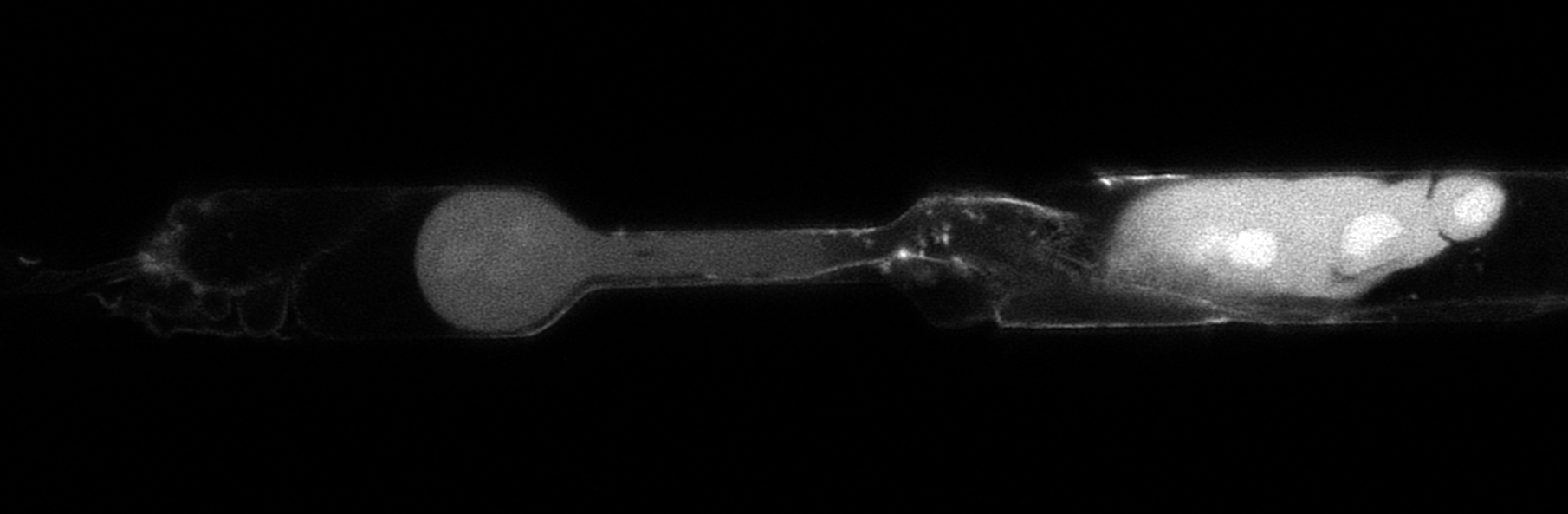

Supplement: Supplementary file 16 — Source data Fig. 2 [file 44318_2025_566_MOESM16_ESM.zip › Fig 2/Fig 2A/siDIAPH1_3_NE rupture.tif]

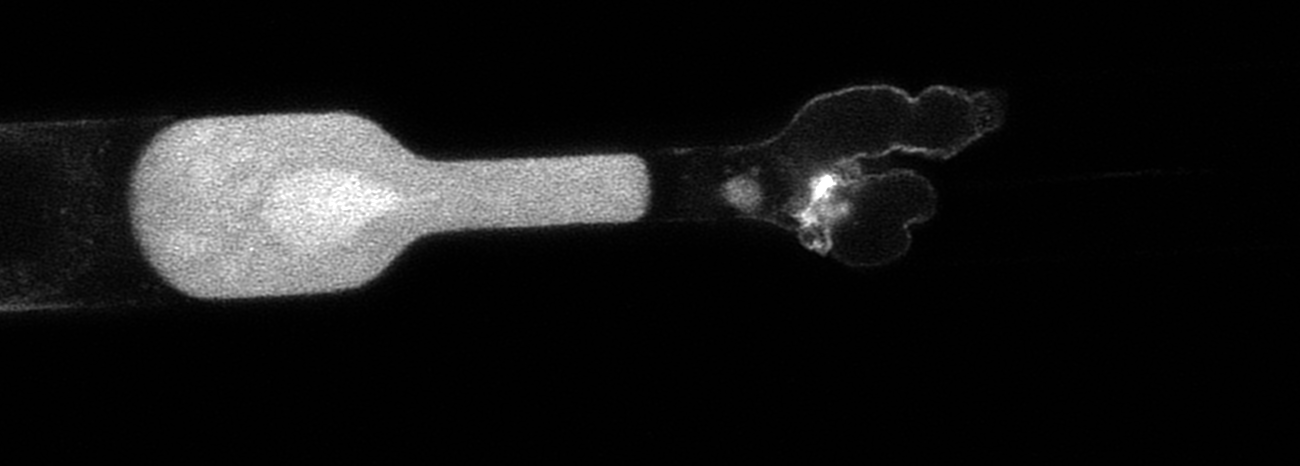

Supplement: Supplementary file 16 — Source data Fig. 2 [file 44318_2025_566_MOESM16_ESM.zip › Fig 2/Fig 2A/siDIAPH1_Before NE rupture.tif]

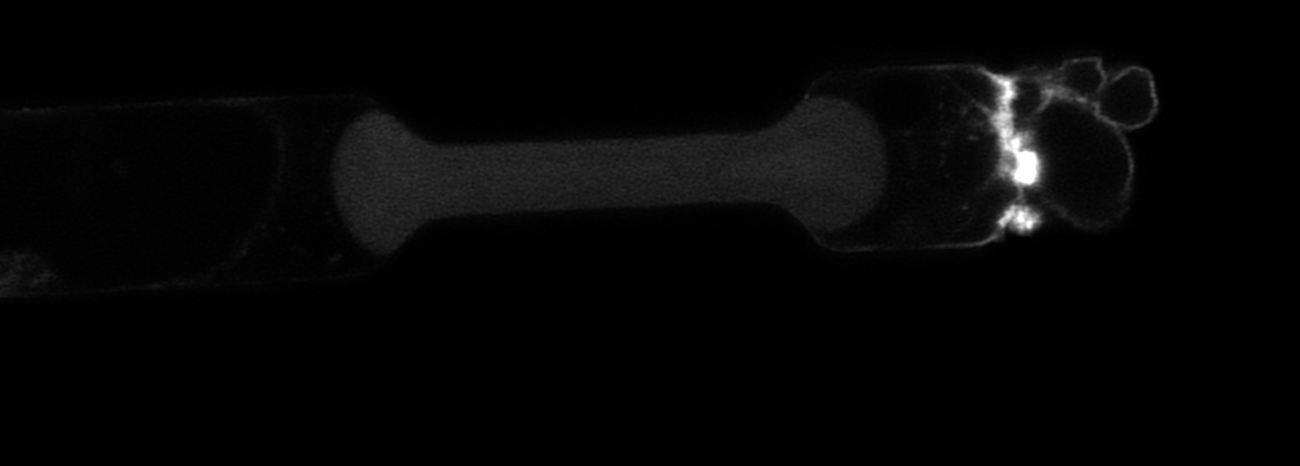

Supplement: Supplementary file 16 — Source data Fig. 2 [file 44318_2025_566_MOESM16_ESM.zip › Fig 2/Fig 2A/siDIAPH1_NE rupture.tif]

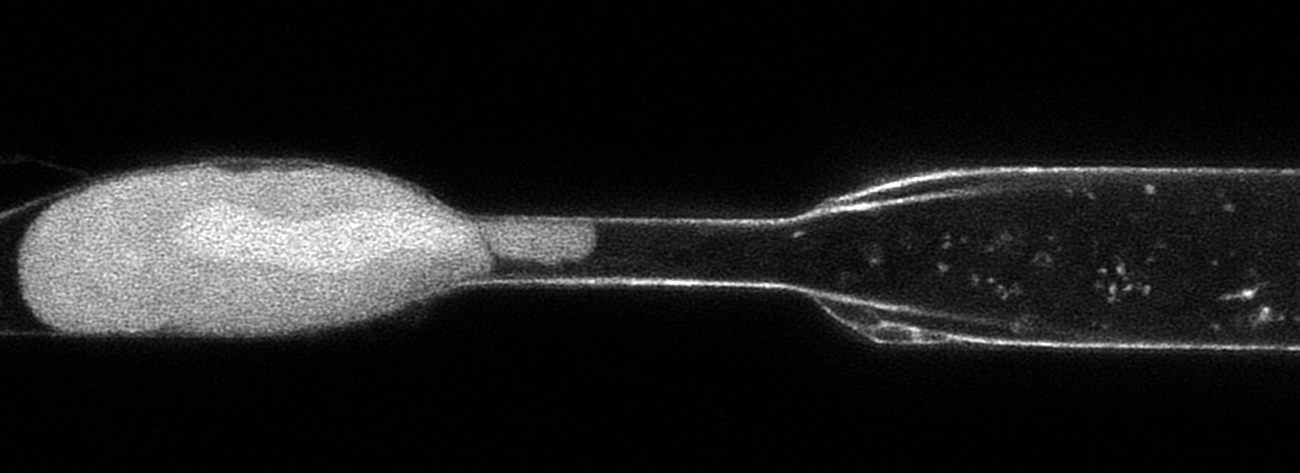

Supplement: Supplementary file 16 — Source data Fig. 2 [file 44318_2025_566_MOESM16_ESM.zip › Fig 2/Fig 2A/siDIAPH2_Before NE rupture.tif]

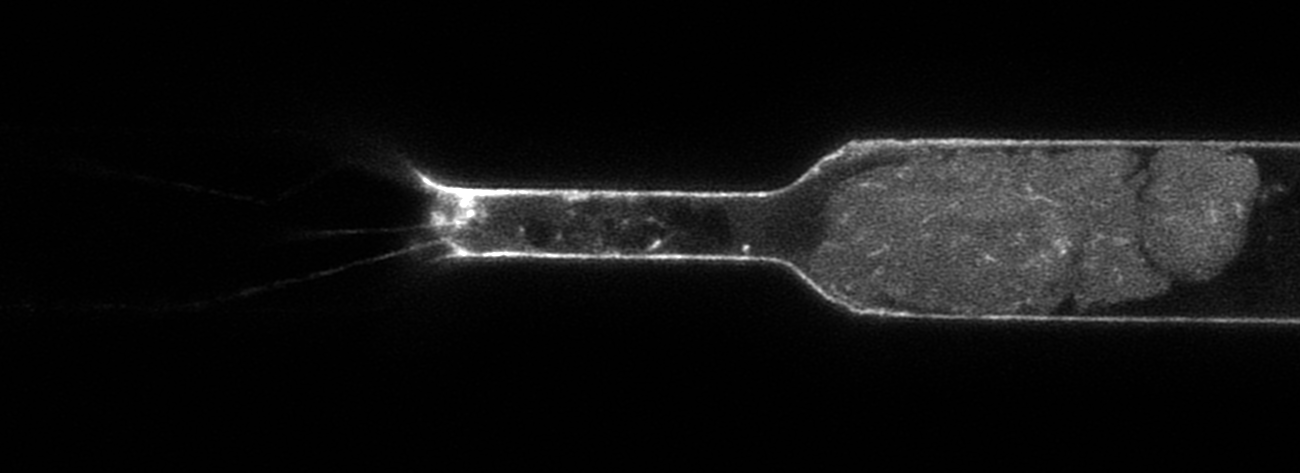

Supplement: Supplementary file 16 — Source data Fig. 2 [file 44318_2025_566_MOESM16_ESM.zip › Fig 2/Fig 2A/siDIAPH2_NE rupture.tif]

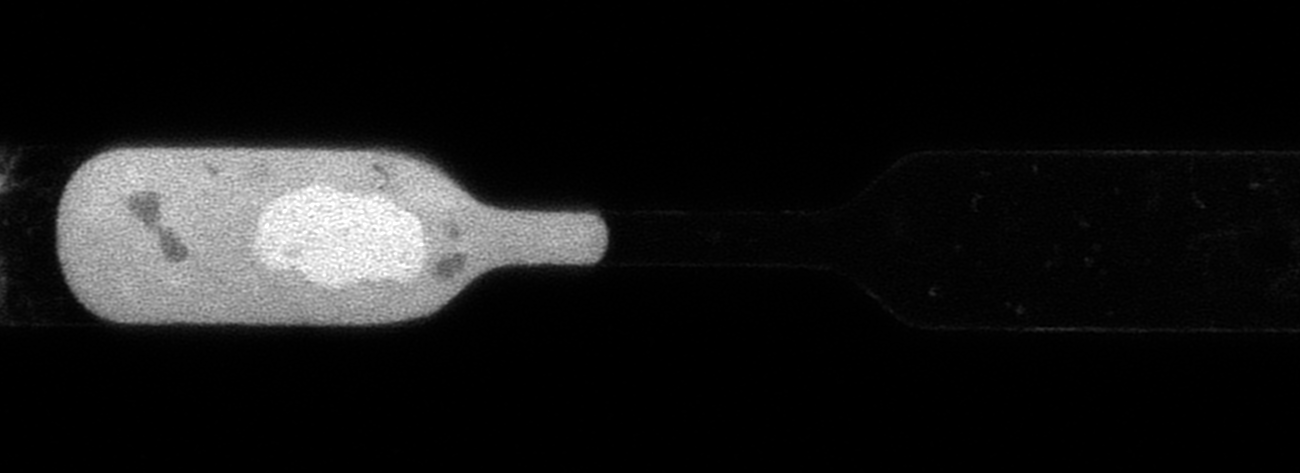

Supplement: Supplementary file 16 — Source data Fig. 2 [file 44318_2025_566_MOESM16_ESM.zip › Fig 2/Fig 2A/siDIAPH3_Before NE rupture.tif]

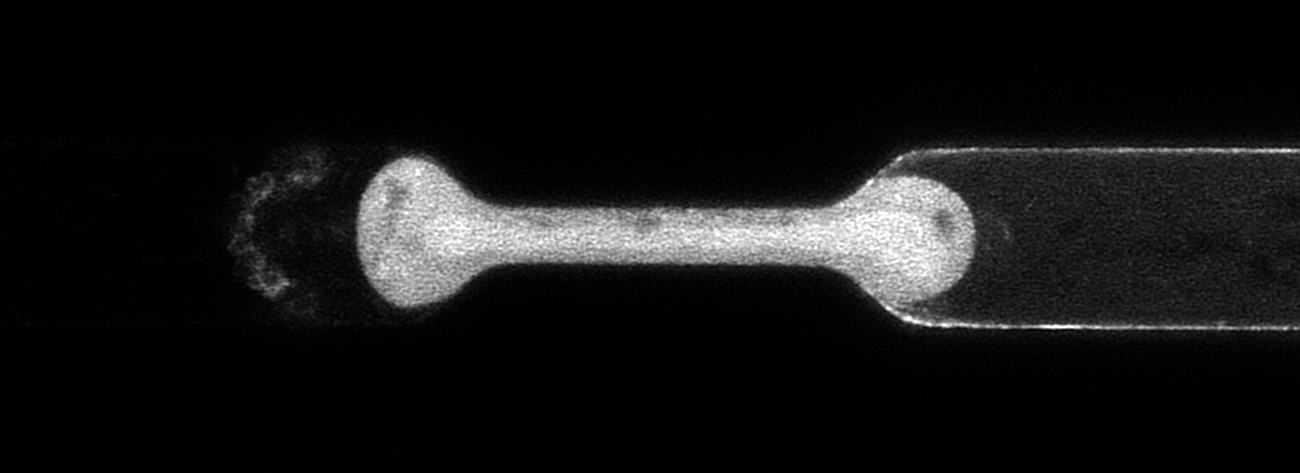

Supplement: Supplementary file 16 — Source data Fig. 2 [file 44318_2025_566_MOESM16_ESM.zip › Fig 2/Fig 2A/siDIAPH3_NE rupture.tif]

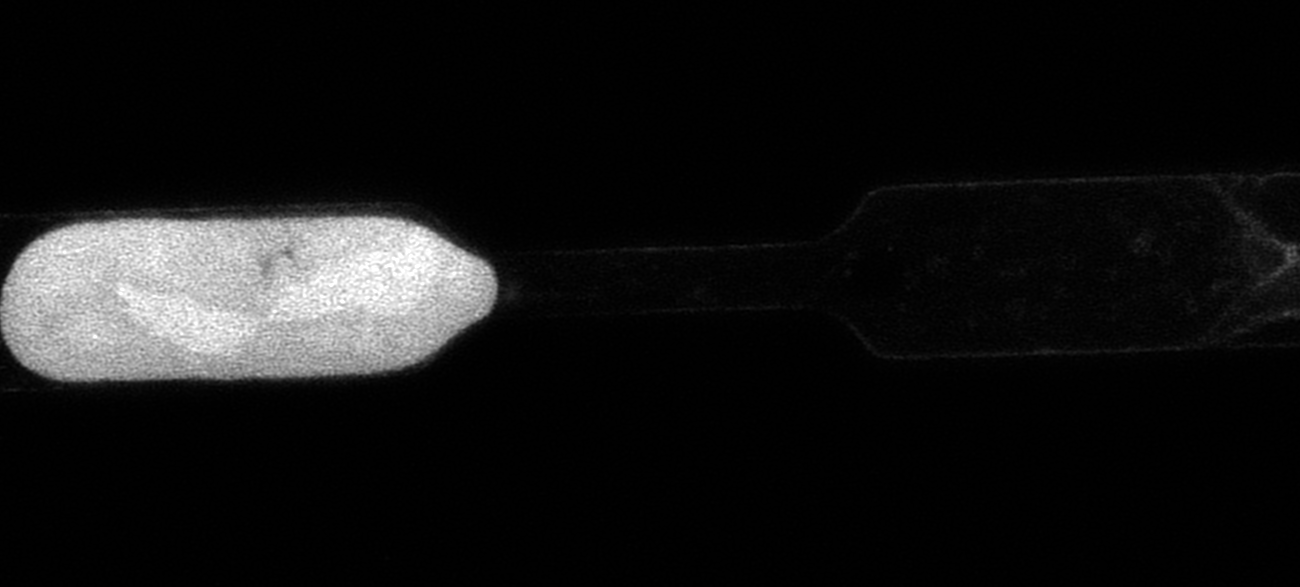

Supplement: Supplementary file 16 — Source data Fig. 2 [file 44318_2025_566_MOESM16_ESM.zip › Fig 2/Fig 2A/siINF2_Before NE rupture.tif]

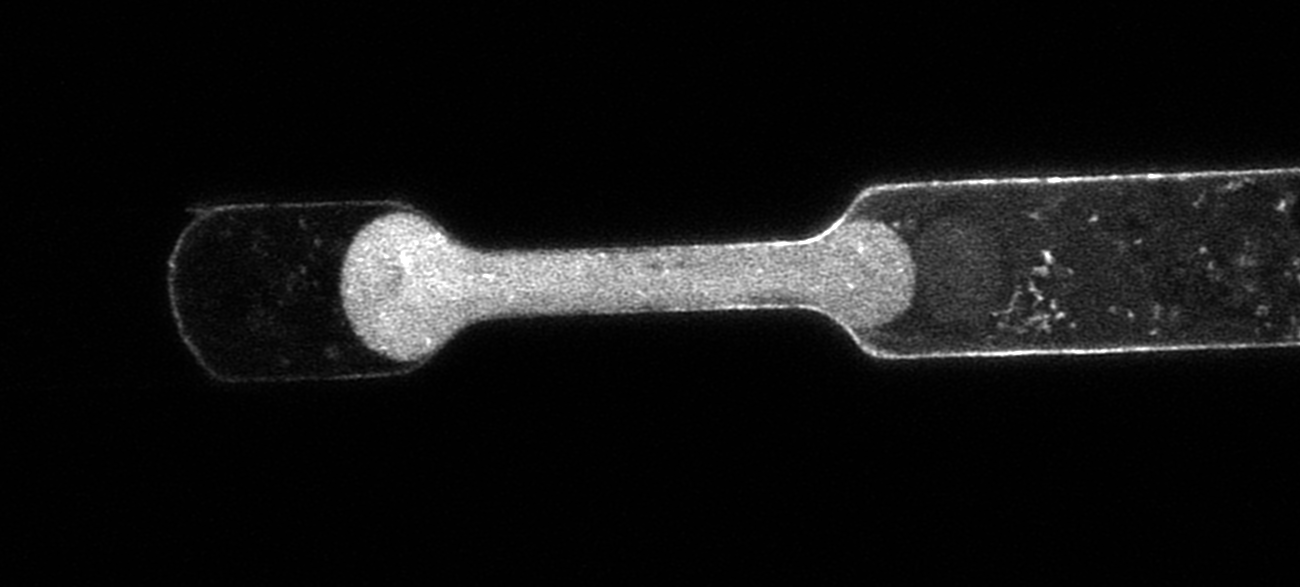

Supplement: Supplementary file 16 — Source data Fig. 2 [file 44318_2025_566_MOESM16_ESM.zip › Fig 2/Fig 2A/siINF2_NE rupture.tif]

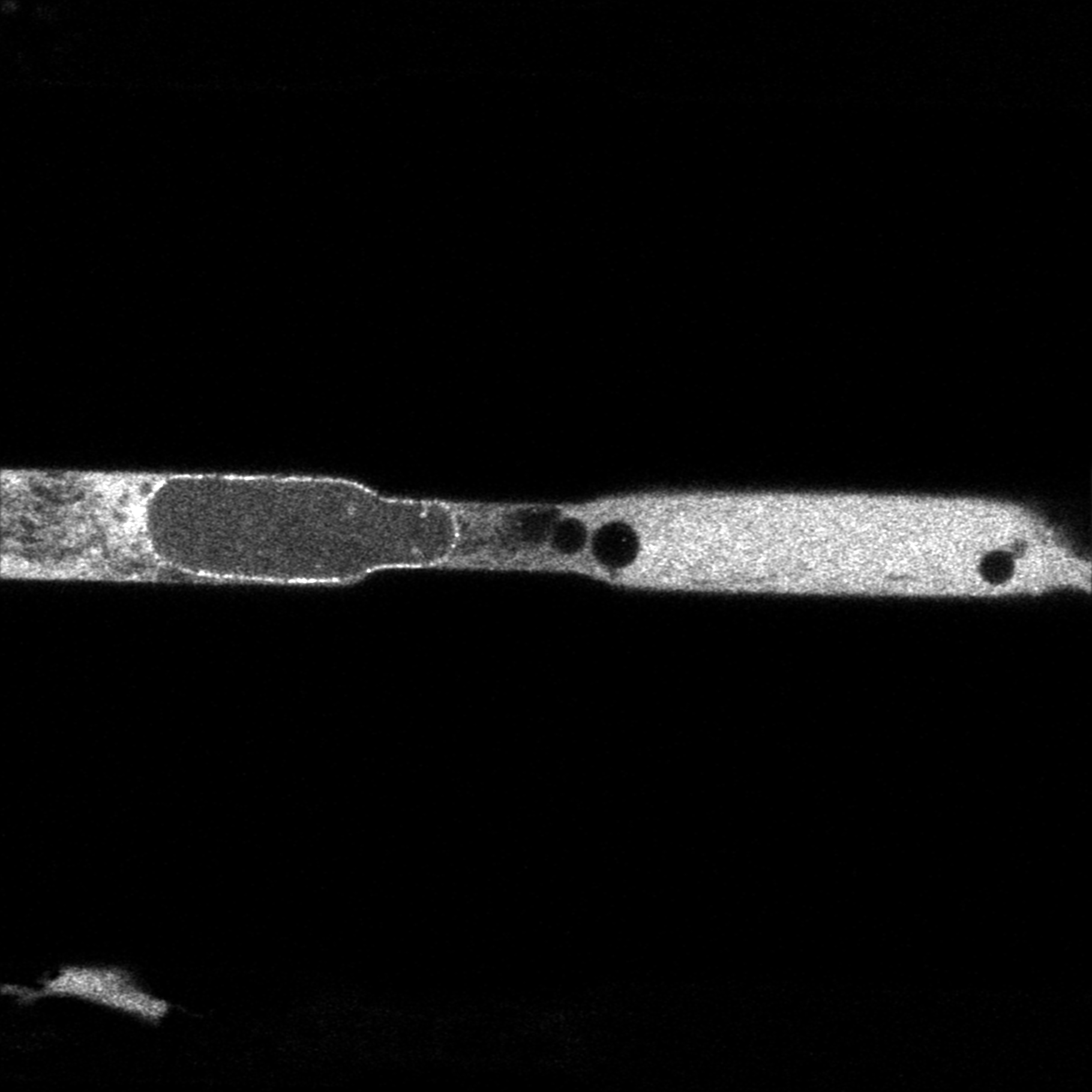

Supplement: Supplementary file 16 — Source data Fig. 2 [file 44318_2025_566_MOESM16_ESM.zip › Fig 2/Fig 2C/GFP-Diaph3_Before NE rupture.tif]

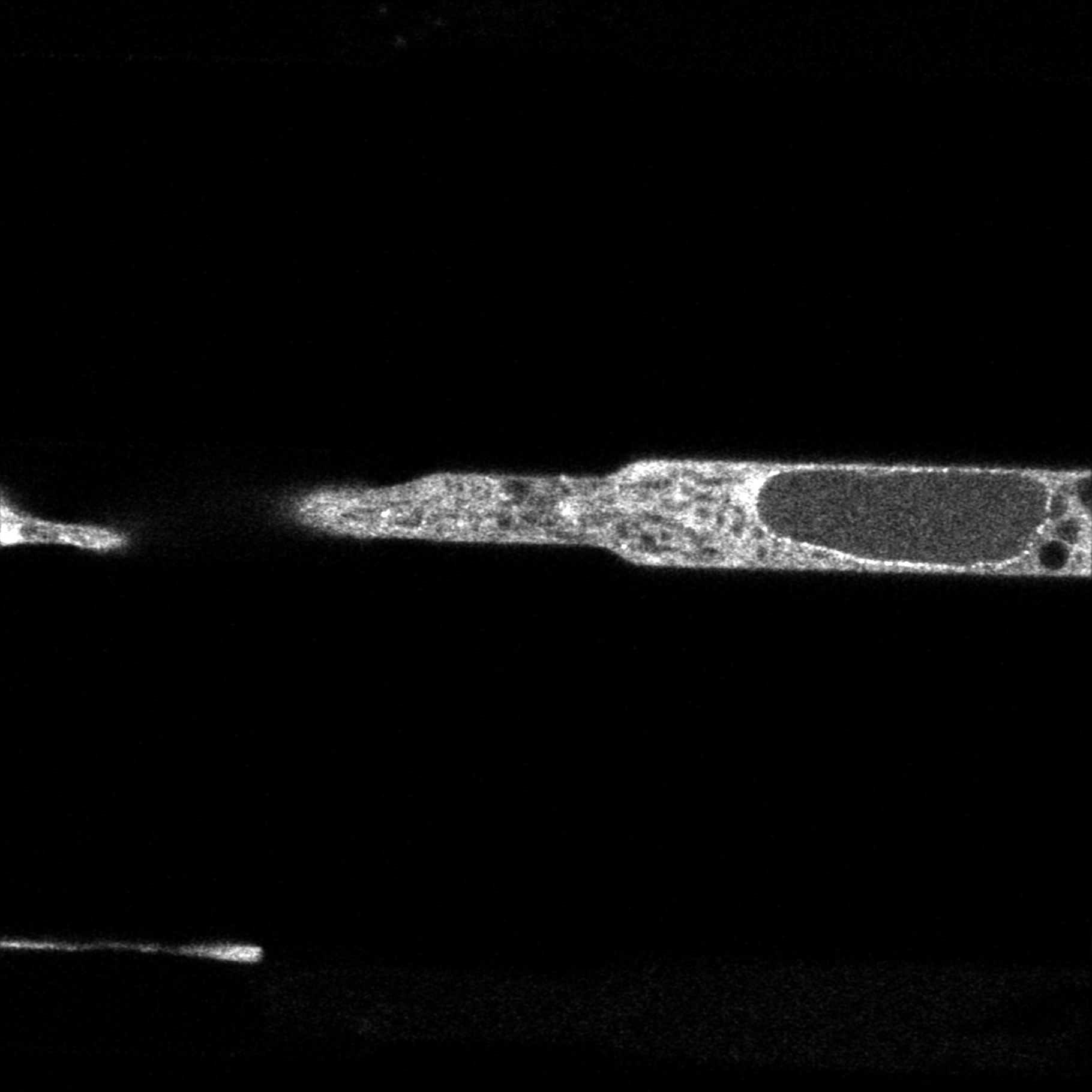

Supplement: Supplementary file 16 — Source data Fig. 2 [file 44318_2025_566_MOESM16_ESM.zip › Fig 2/Fig 2C/GFP-Diaph3_Diaph3 export.tif]

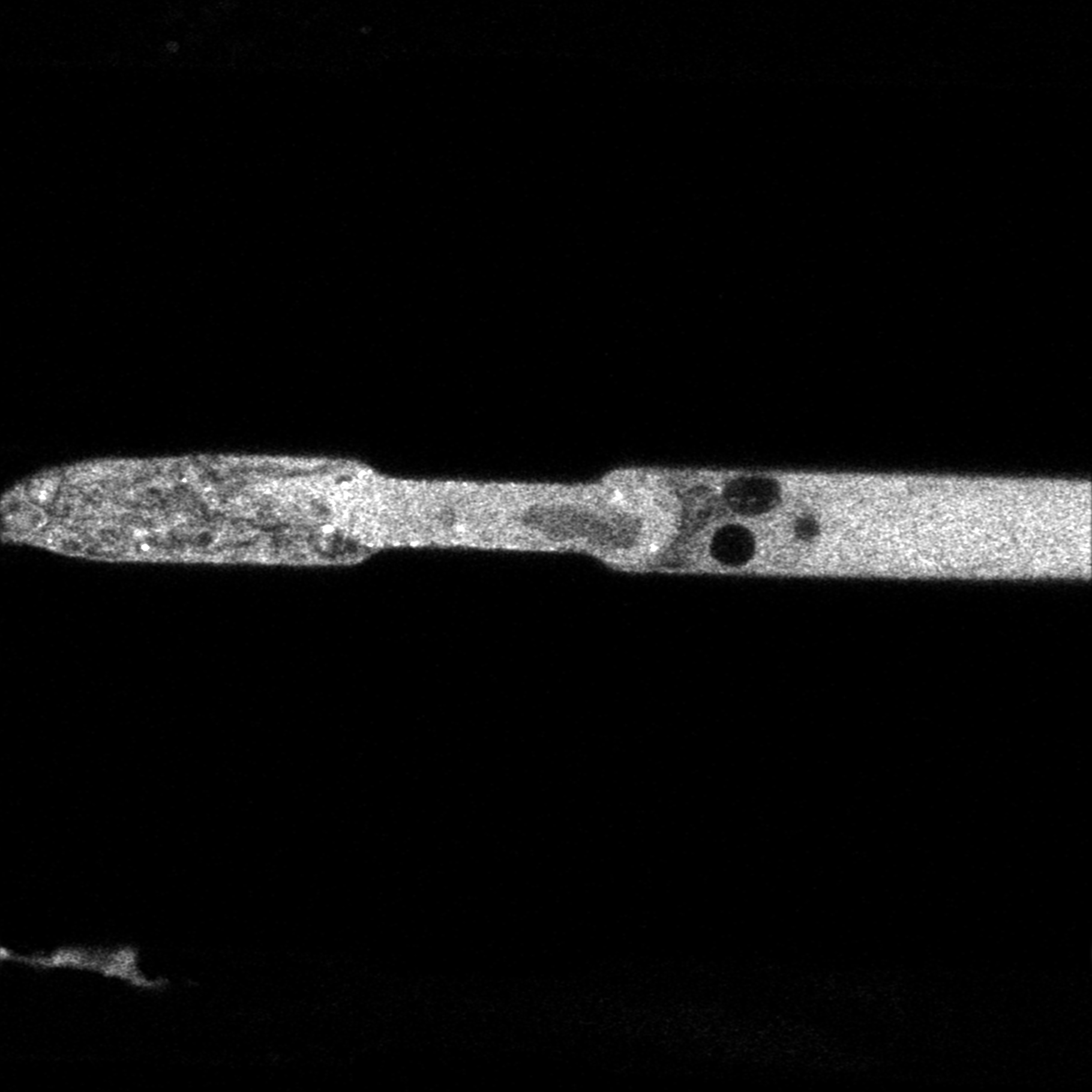

Supplement: Supplementary file 16 — Source data Fig. 2 [file 44318_2025_566_MOESM16_ESM.zip › Fig 2/Fig 2C/GFP-Diaph3_NE rupture.tif]

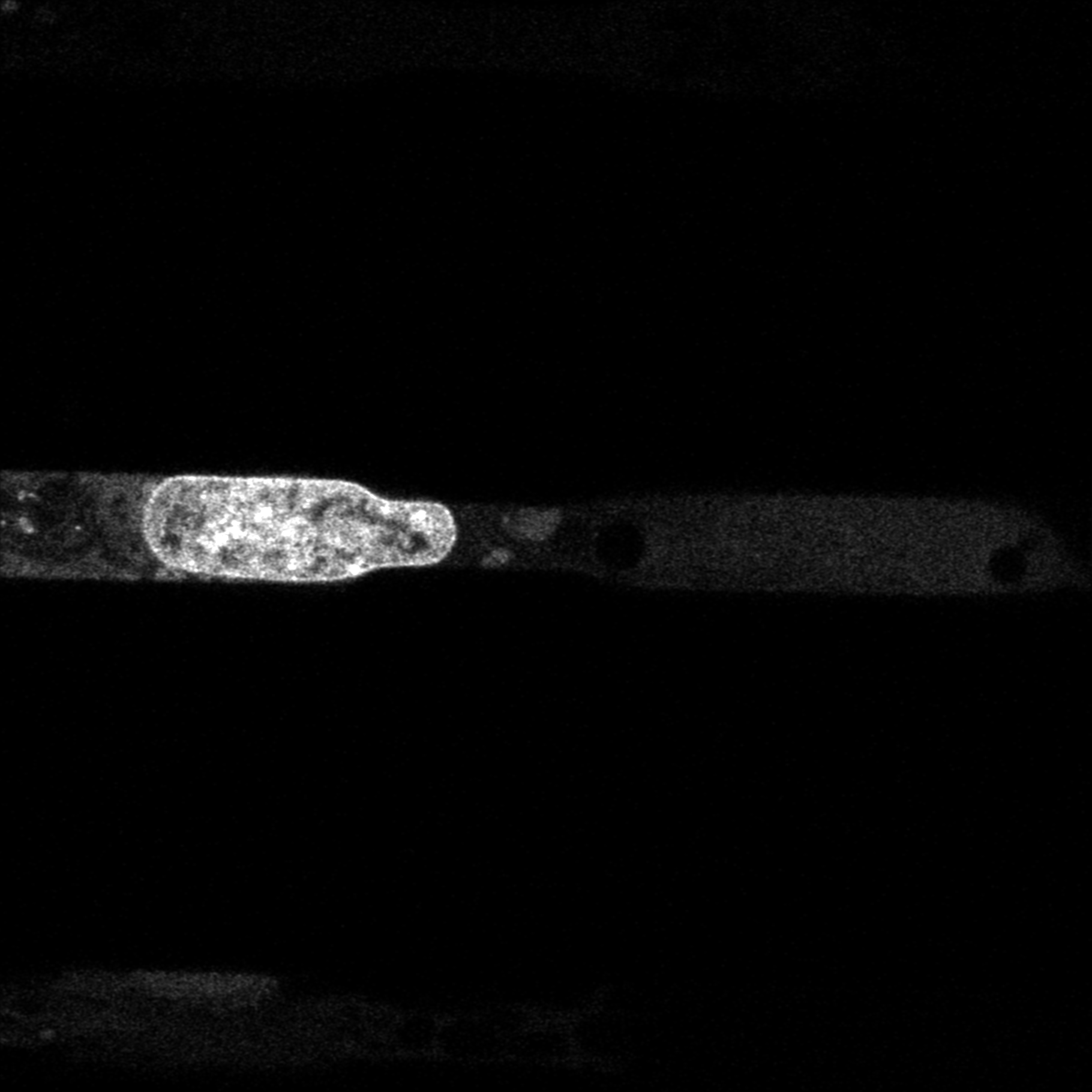

Supplement: Supplementary file 16 — Source data Fig. 2 [file 44318_2025_566_MOESM16_ESM.zip › Fig 2/Fig 2C/icGAS-mCherry_Before NE rupture.tif]

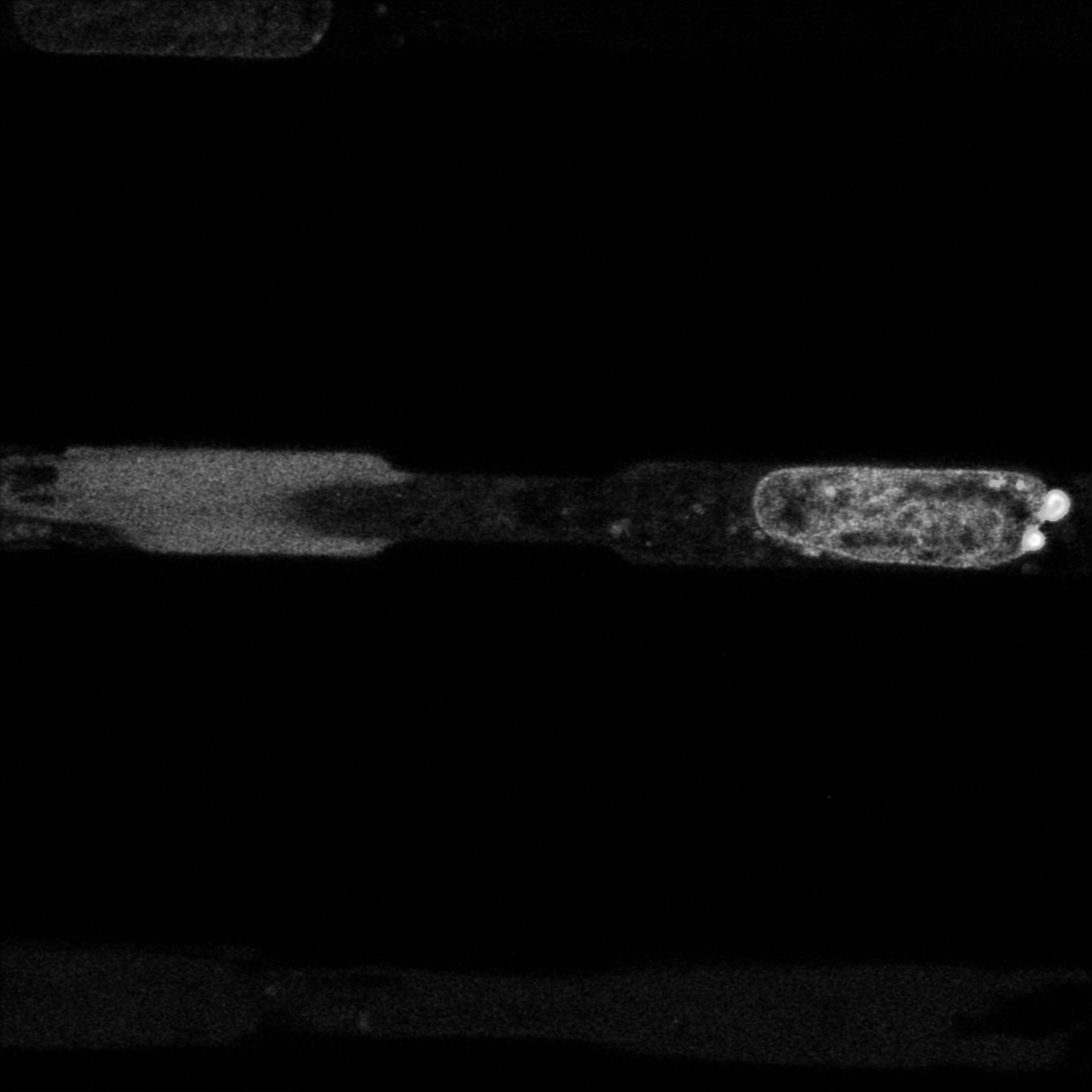

Supplement: Supplementary file 16 — Source data Fig. 2 [file 44318_2025_566_MOESM16_ESM.zip › Fig 2/Fig 2C/icGAS-mCherry_Diaph3 export.tif]

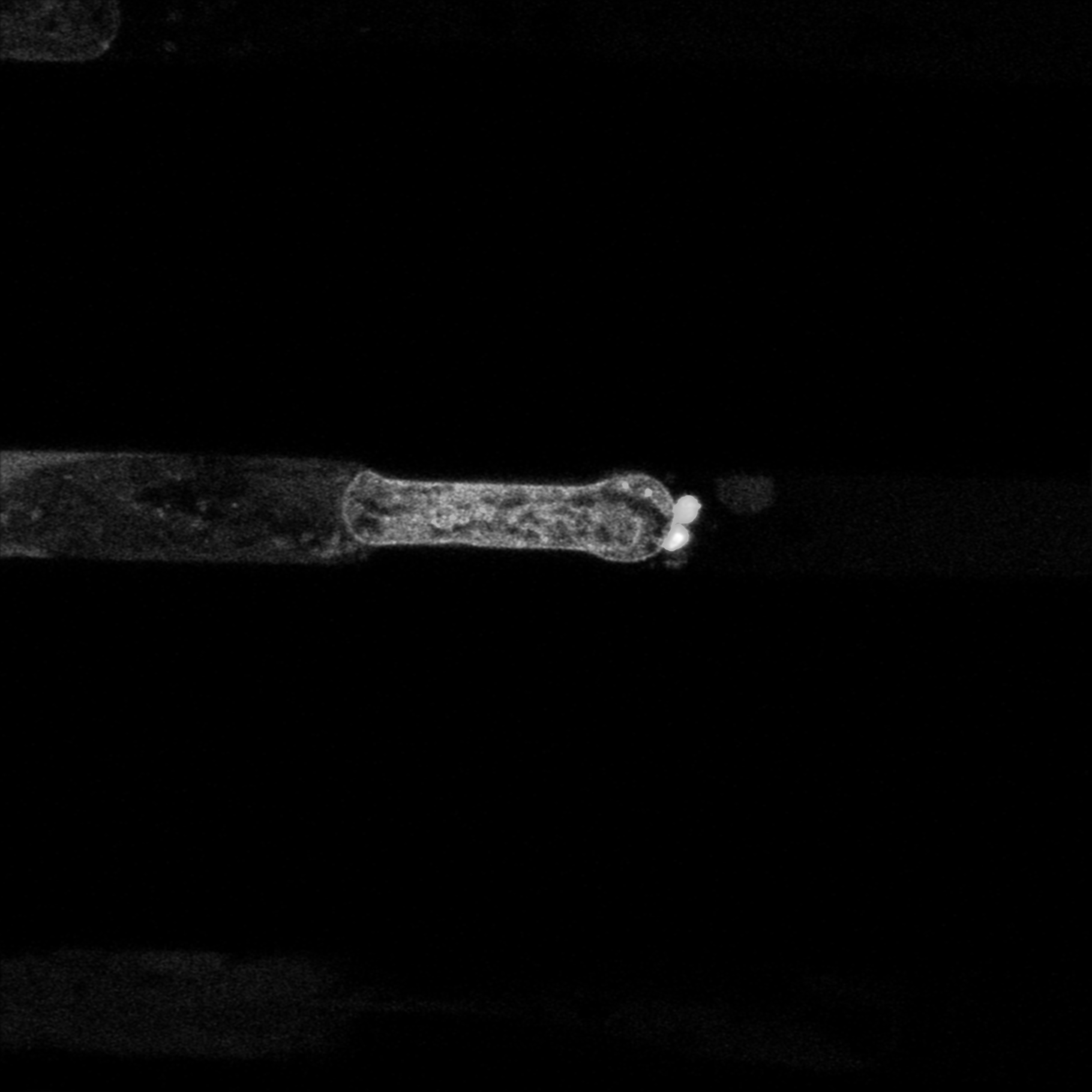

Supplement: Supplementary file 16 — Source data Fig. 2 [file 44318_2025_566_MOESM16_ESM.zip › Fig 2/Fig 2C/icGAS-mCherry_NE rupture.tif]

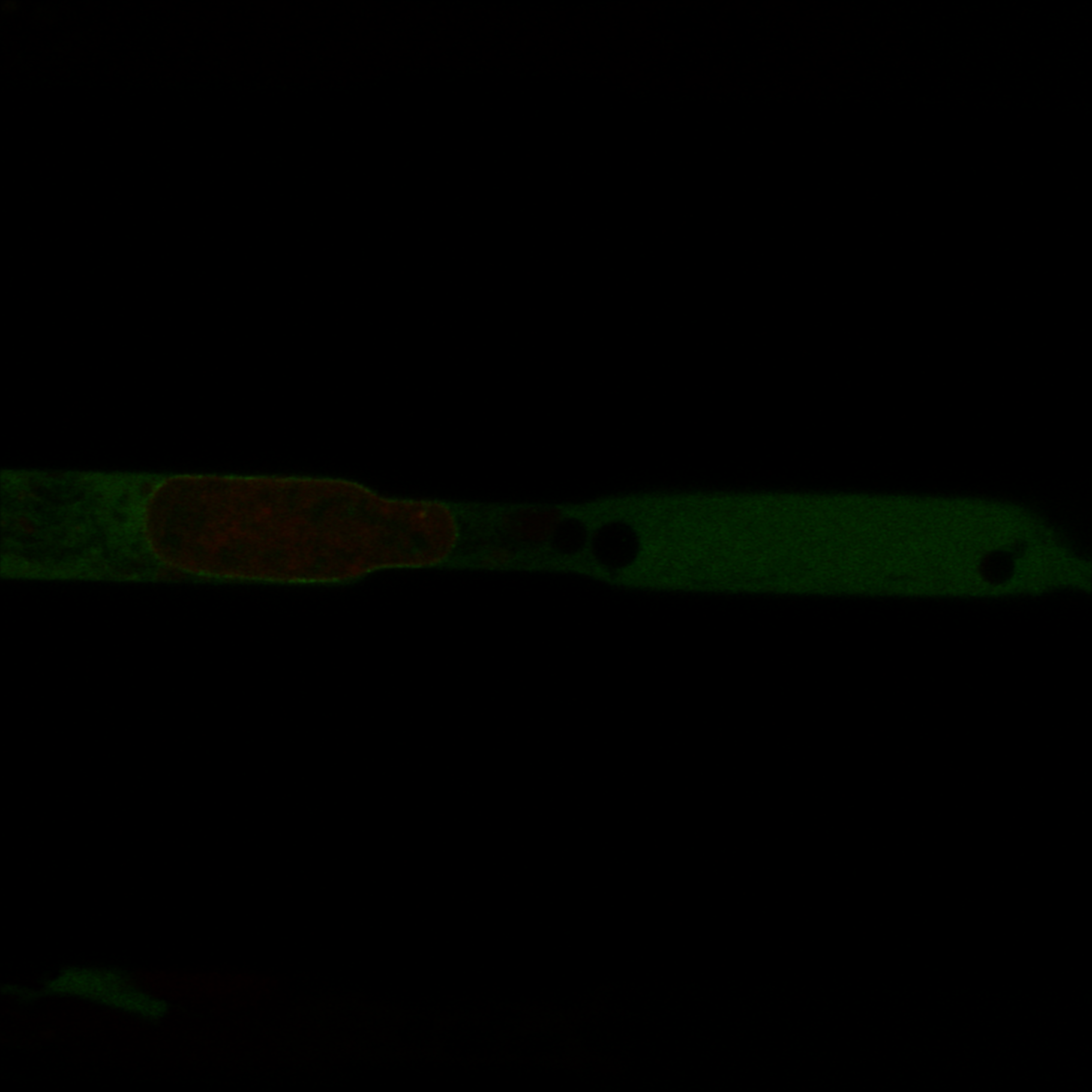

Supplement: Supplementary file 16 — Source data Fig. 2 [file 44318_2025_566_MOESM16_ESM.zip › Fig 2/Fig 2C/Merge_Before NE rupture.tif]

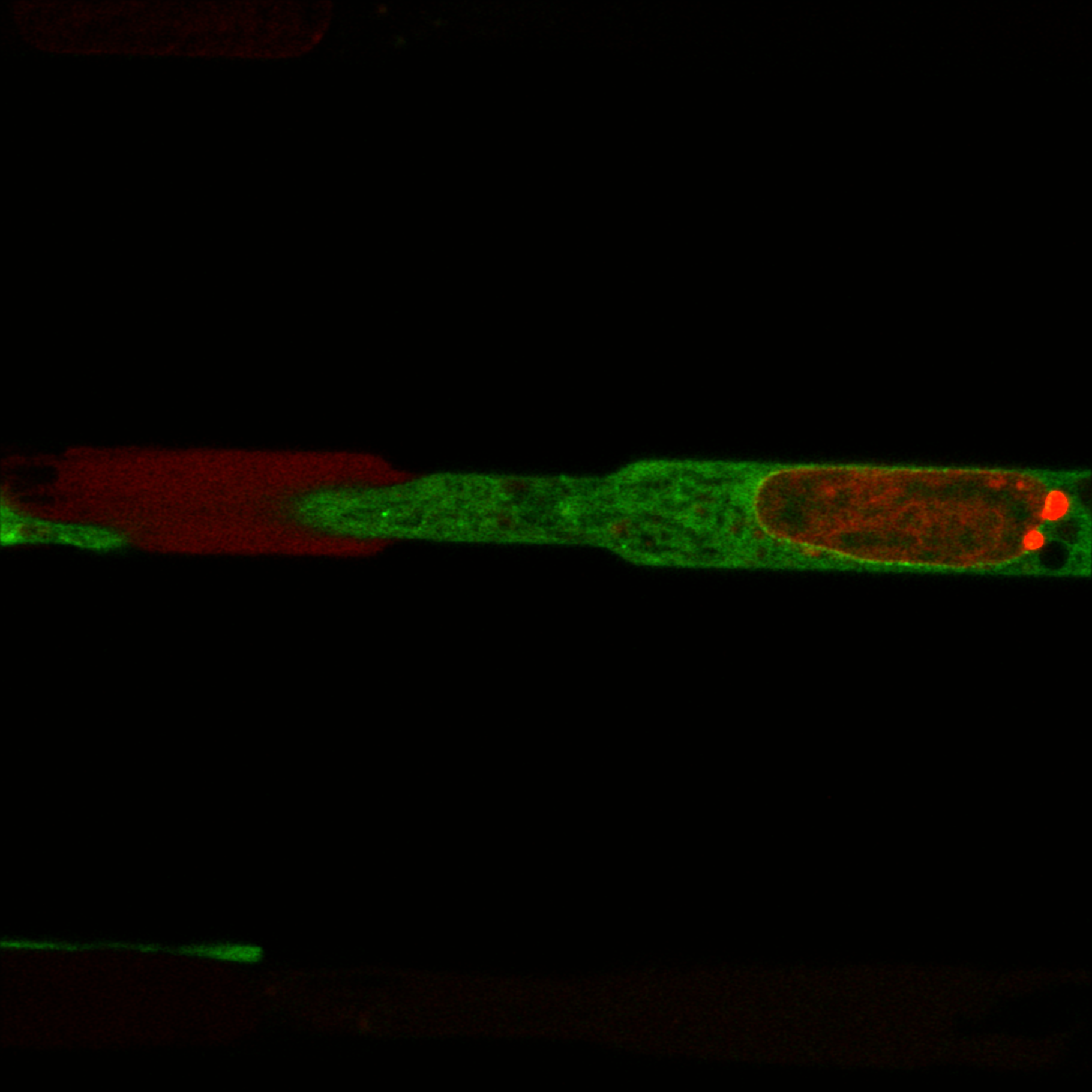

Supplement: Supplementary file 16 — Source data Fig. 2 [file 44318_2025_566_MOESM16_ESM.zip › Fig 2/Fig 2C/Merge_Diaph3 export.tif]

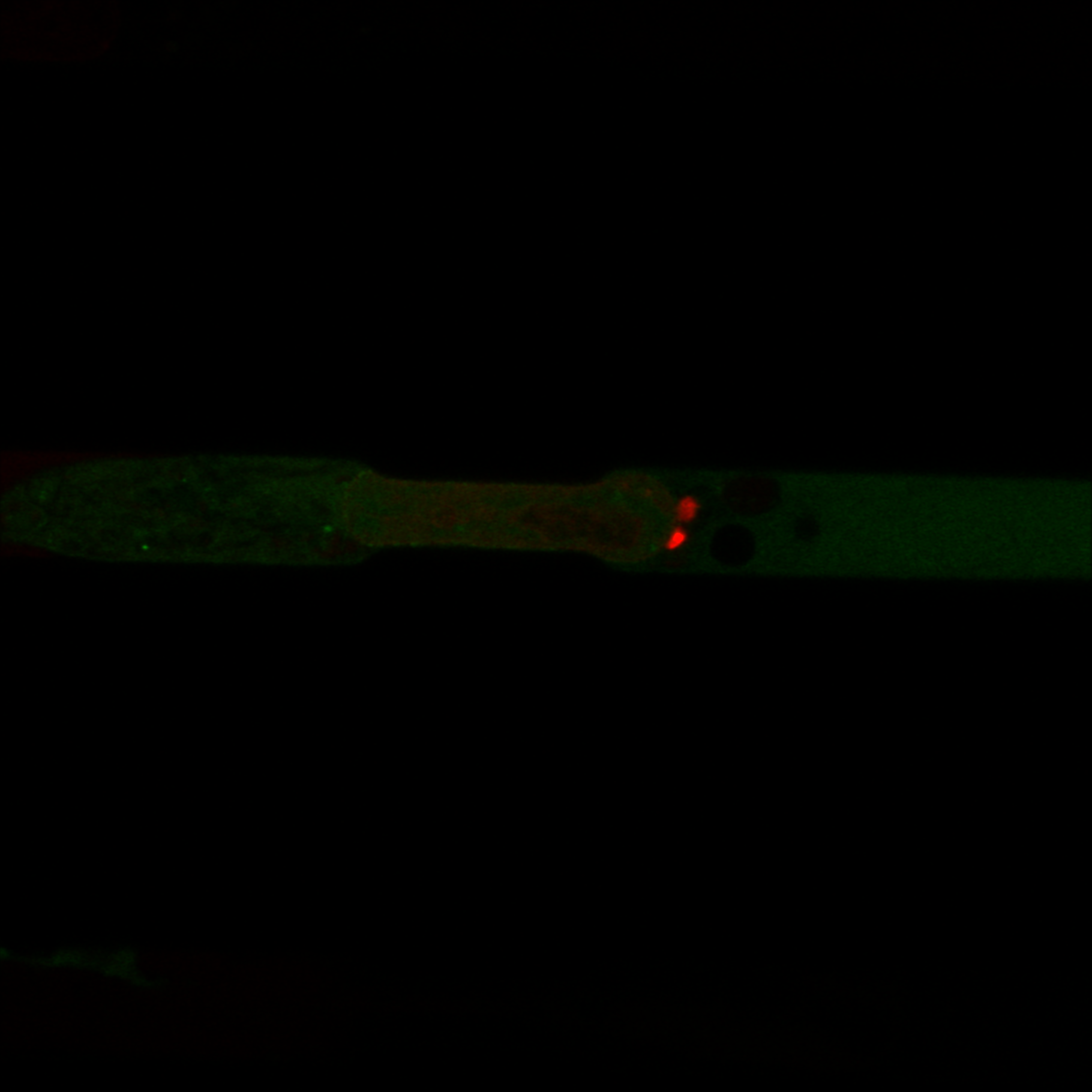

Supplement: Supplementary file 16 — Source data Fig. 2 [file 44318_2025_566_MOESM16_ESM.zip › Fig 2/Fig 2C/Merge_NE rupture.tif]

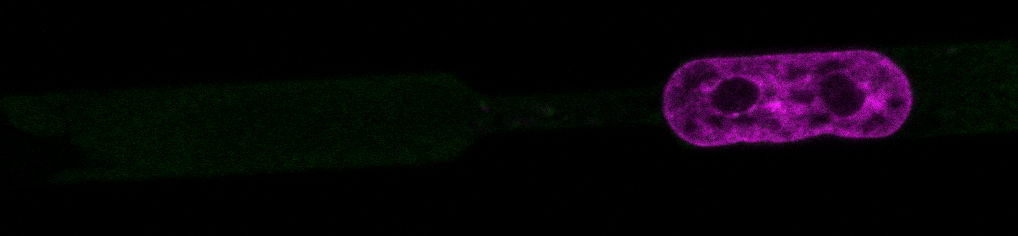

Supplement: Supplementary file 17 — Source data Fig. 3 [file 44318_2025_566_MOESM17_ESM.zip › Fig 3/Fig 3B/siCtrl/siCtrl_0min_Merge.tif]

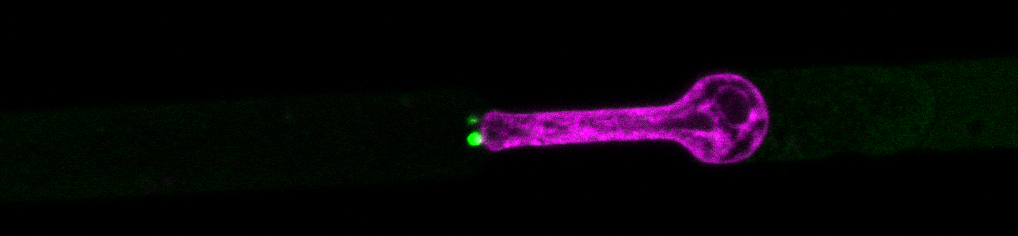

Supplement: Supplementary file 17 — Source data Fig. 3 [file 44318_2025_566_MOESM17_ESM.zip › Fig 3/Fig 3B/siCtrl/siCtrl_30min_Merge.tif]

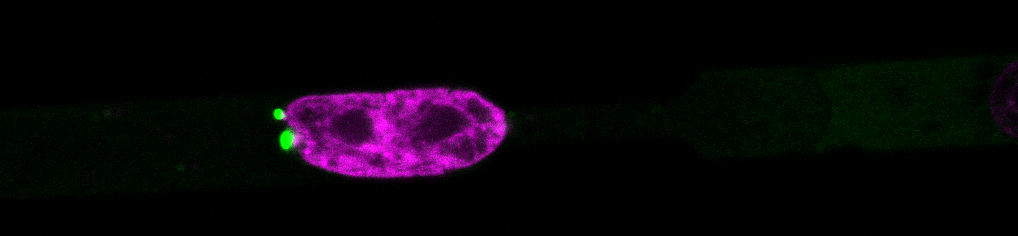

Supplement: Supplementary file 17 — Source data Fig. 3 [file 44318_2025_566_MOESM17_ESM.zip › Fig 3/Fig 3B/siCtrl/siCtrl_40min_Merge.tif]

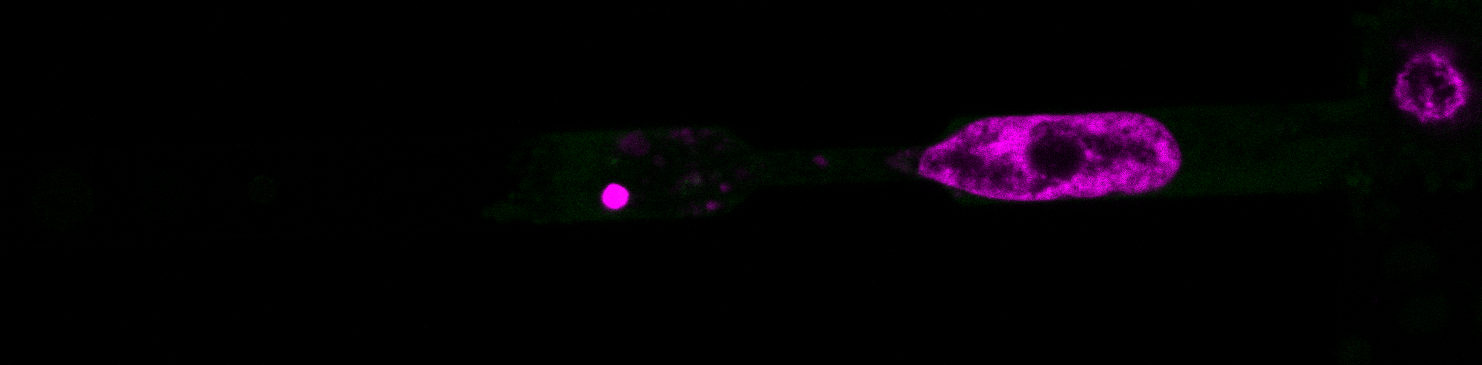

Supplement: Supplementary file 17 — Source data Fig. 3 [file 44318_2025_566_MOESM17_ESM.zip › Fig 3/Fig 3B/siDIAPH1_3/siDIAPH1_3 _0min_Merge.tif]

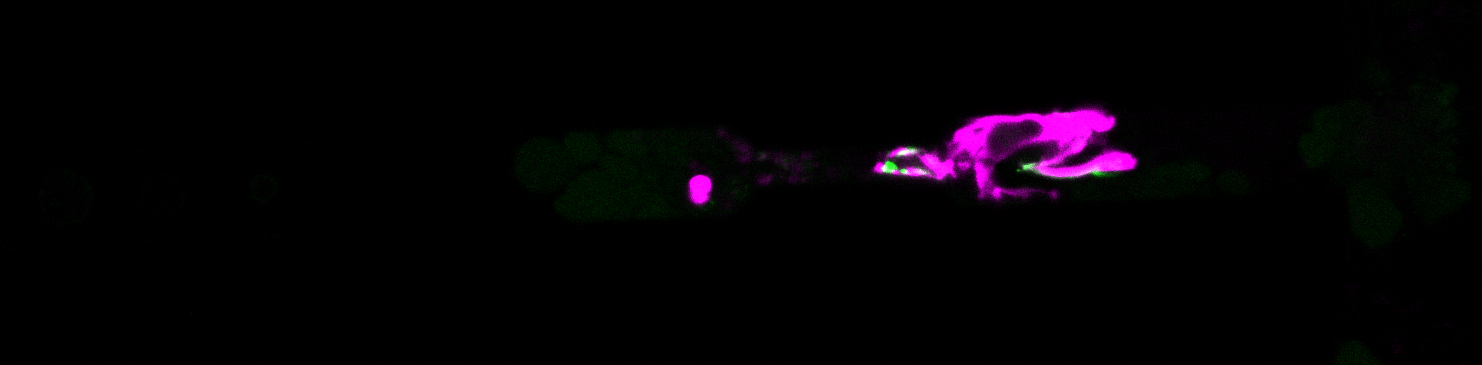

Supplement: Supplementary file 17 — Source data Fig. 3 [file 44318_2025_566_MOESM17_ESM.zip › Fig 3/Fig 3B/siDIAPH1_3/siDIAPH1_3 _170min_Merge.tif]

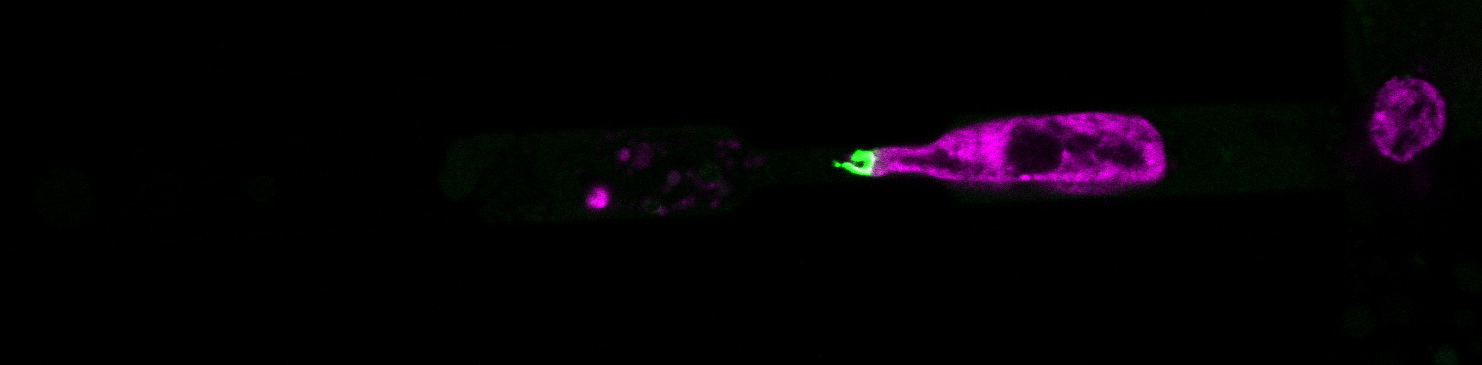

Supplement: Supplementary file 17 — Source data Fig. 3 [file 44318_2025_566_MOESM17_ESM.zip › Fig 3/Fig 3B/siDIAPH1_3/siDIAPH1_3 _40min_Merge.tif]

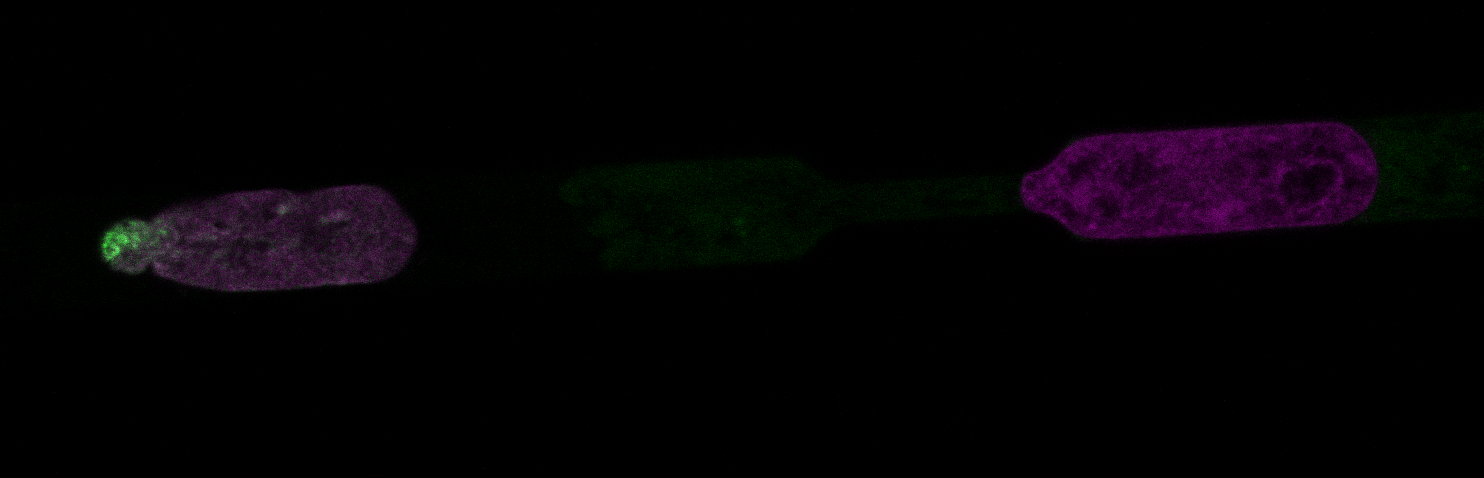

Supplement: Supplementary file 17 — Source data Fig. 3 [file 44318_2025_566_MOESM17_ESM.zip › Fig 3/Fig 3D/NLS-BFP/NLS-BFP_0min_Merge.tif]

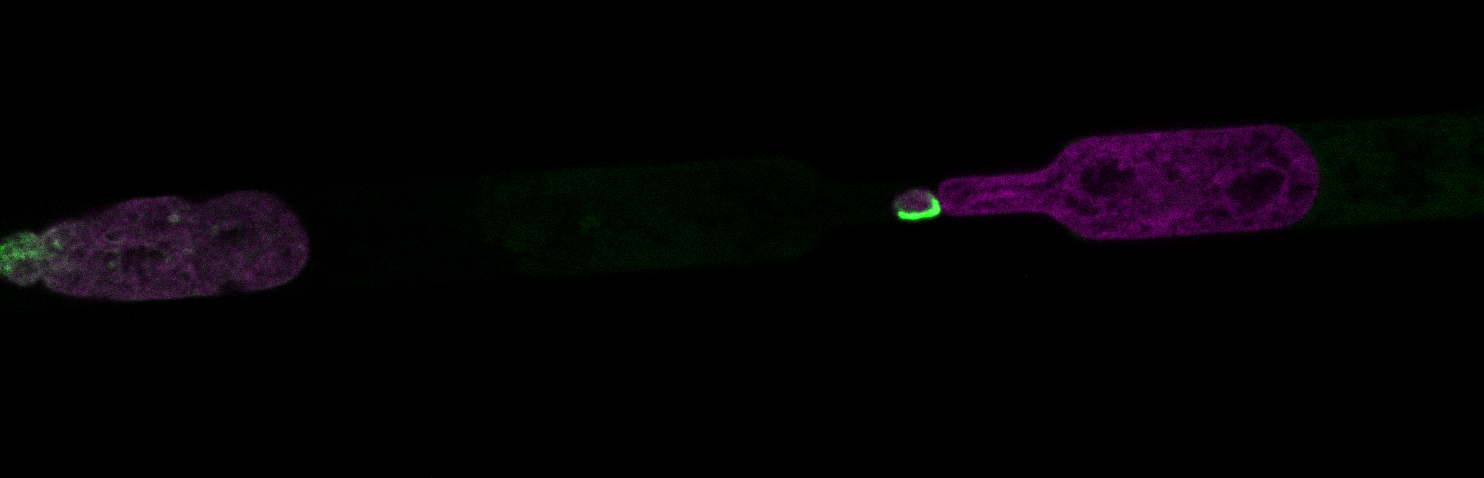

Supplement: Supplementary file 17 — Source data Fig. 3 [file 44318_2025_566_MOESM17_ESM.zip › Fig 3/Fig 3D/NLS-BFP/NLS-BFP_10min_Merge.tif]

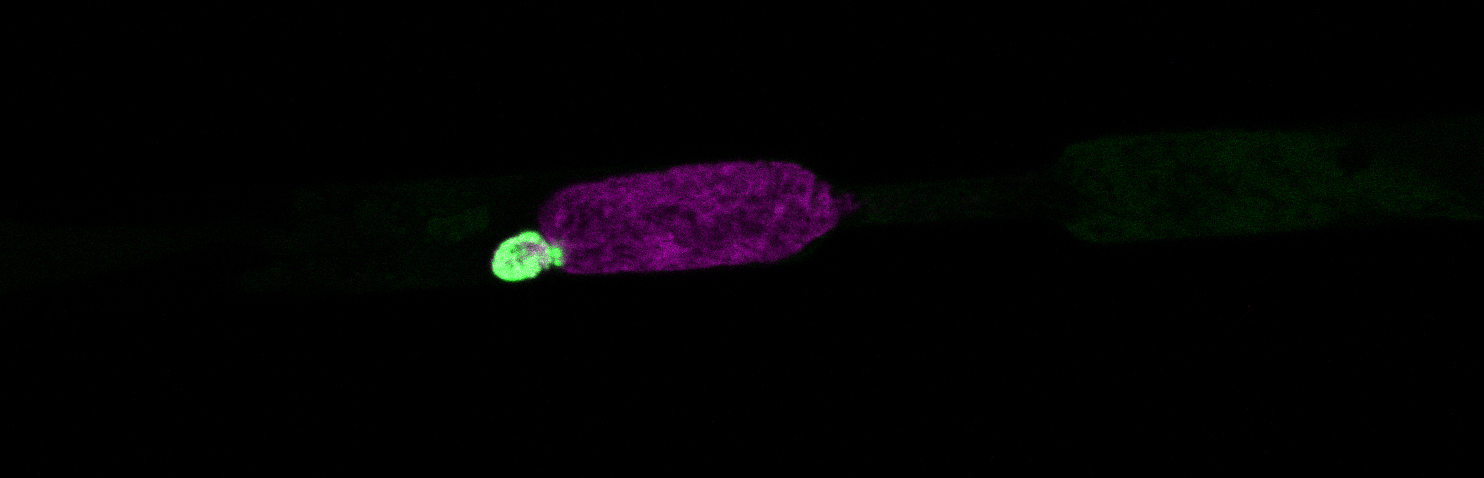

Supplement: Supplementary file 17 — Source data Fig. 3 [file 44318_2025_566_MOESM17_ESM.zip › Fig 3/Fig 3D/NLS-BFP/NLS-BFP_70min_Merge.tif]

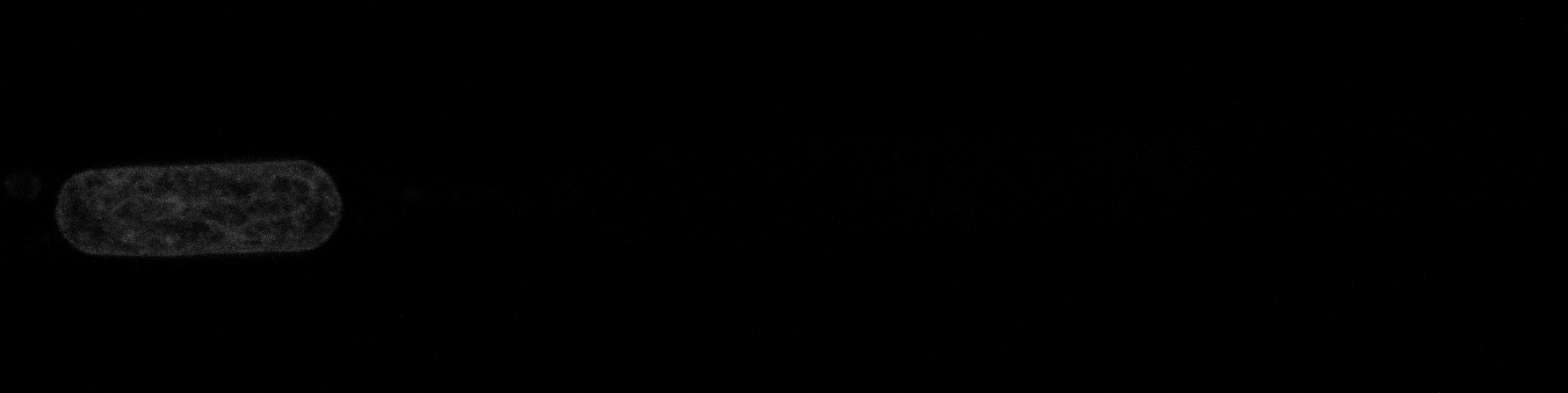

Supplement: Supplementary file 17 — Source data Fig. 3 [file 44318_2025_566_MOESM17_ESM.zip › Fig 3/Fig 3D/R62D-NLS/R62D-NLS_0min_H2B-mCherry.tif]

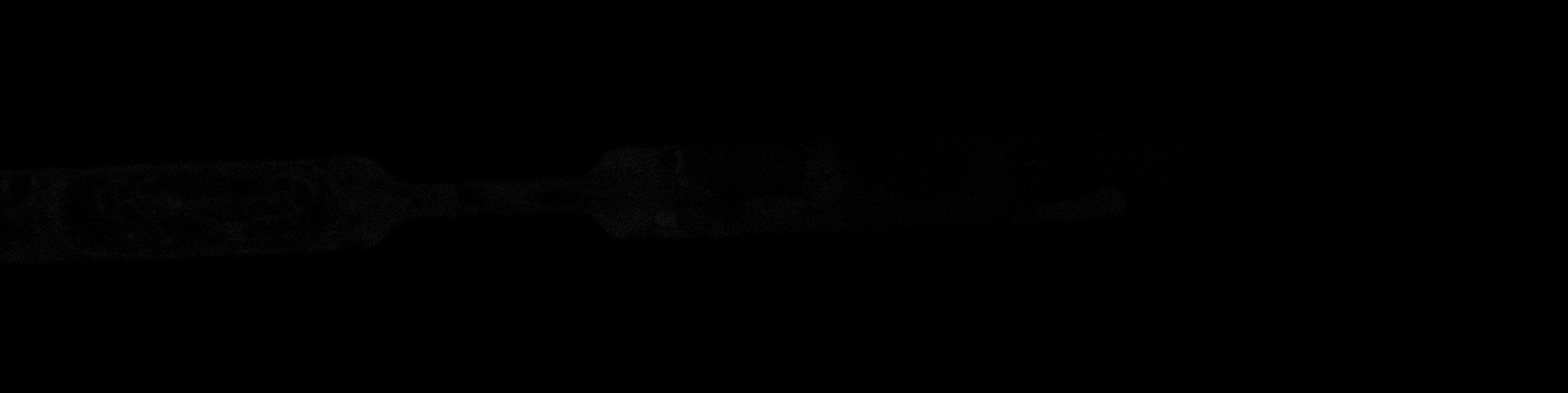

Supplement: Supplementary file 17 — Source data Fig. 3 [file 44318_2025_566_MOESM17_ESM.zip › Fig 3/Fig 3D/R62D-NLS/R62D-NLS_0min_icGAS-GFP.tif]

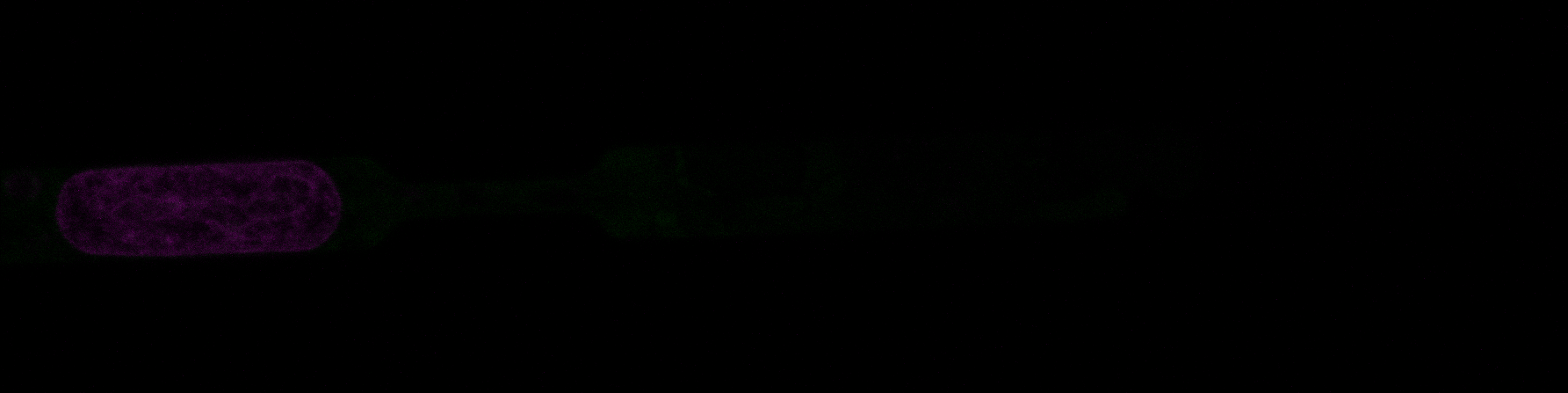

Supplement: Supplementary file 17 — Source data Fig. 3 [file 44318_2025_566_MOESM17_ESM.zip › Fig 3/Fig 3D/R62D-NLS/R62D-NLS_0min_Merge.tif]

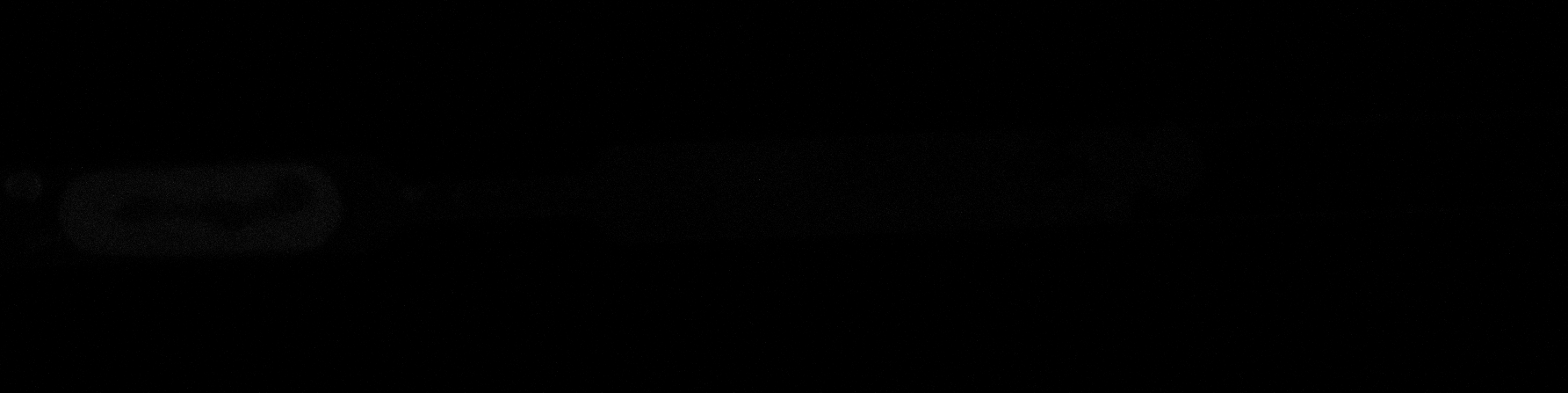

Supplement: Supplementary file 17 — Source data Fig. 3 [file 44318_2025_566_MOESM17_ESM.zip › Fig 3/Fig 3D/R62D-NLS/R62D-NLS_0min_NLS-BFP-Actin-R62D.tif]

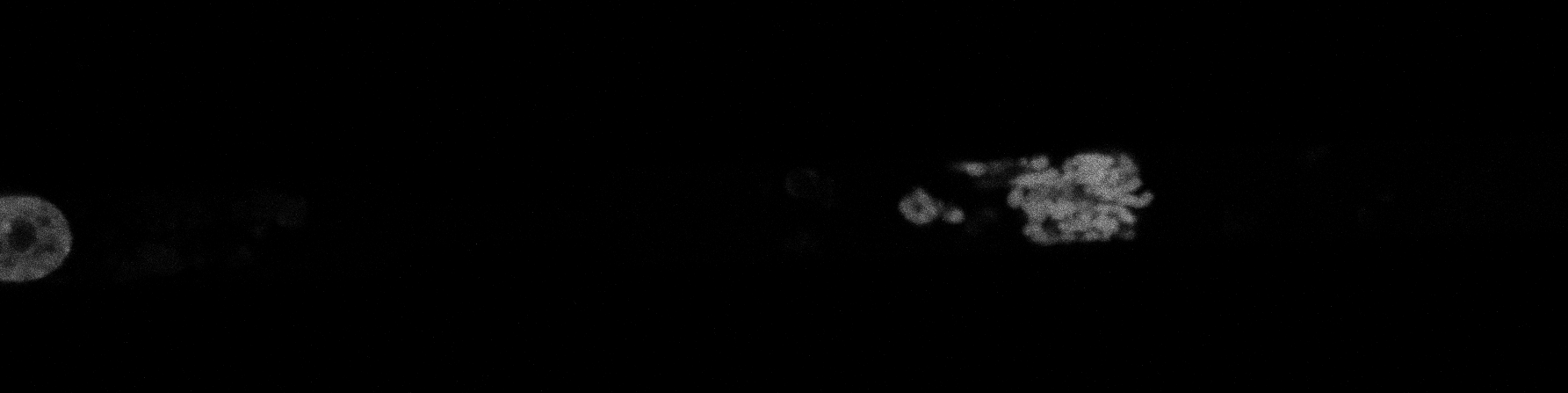

Supplement: Supplementary file 17 — Source data Fig. 3 [file 44318_2025_566_MOESM17_ESM.zip › Fig 3/Fig 3D/R62D-NLS/R62D-NLS_550min_H2B-mCherry.tif]

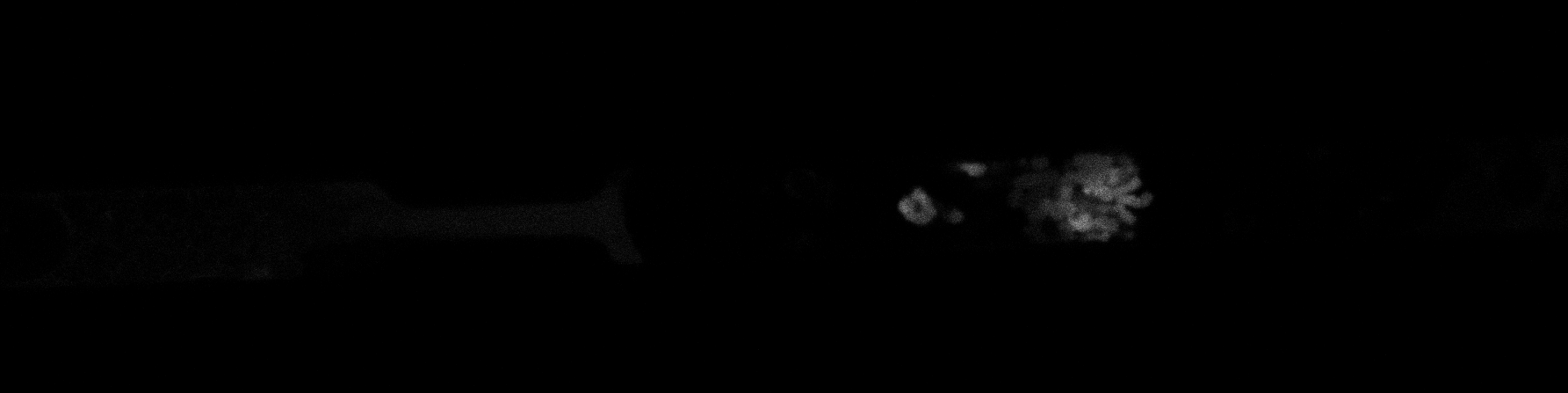

Supplement: Supplementary file 17 — Source data Fig. 3 [file 44318_2025_566_MOESM17_ESM.zip › Fig 3/Fig 3D/R62D-NLS/R62D-NLS_550min_icGAS-GFP.tif]

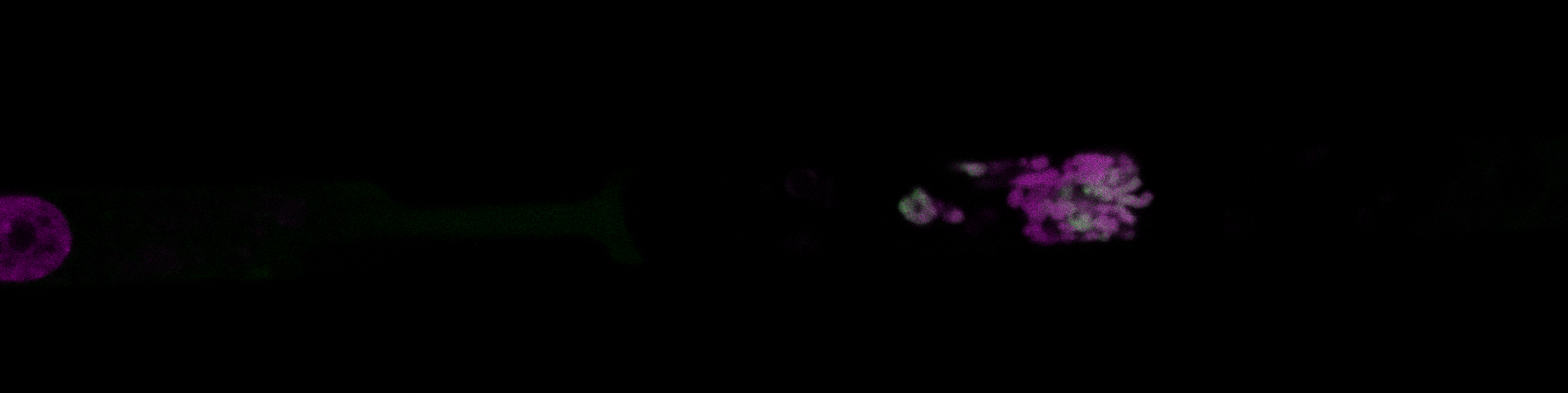

Supplement: Supplementary file 17 — Source data Fig. 3 [file 44318_2025_566_MOESM17_ESM.zip › Fig 3/Fig 3D/R62D-NLS/R62D-NLS_550min_Merge.tif]

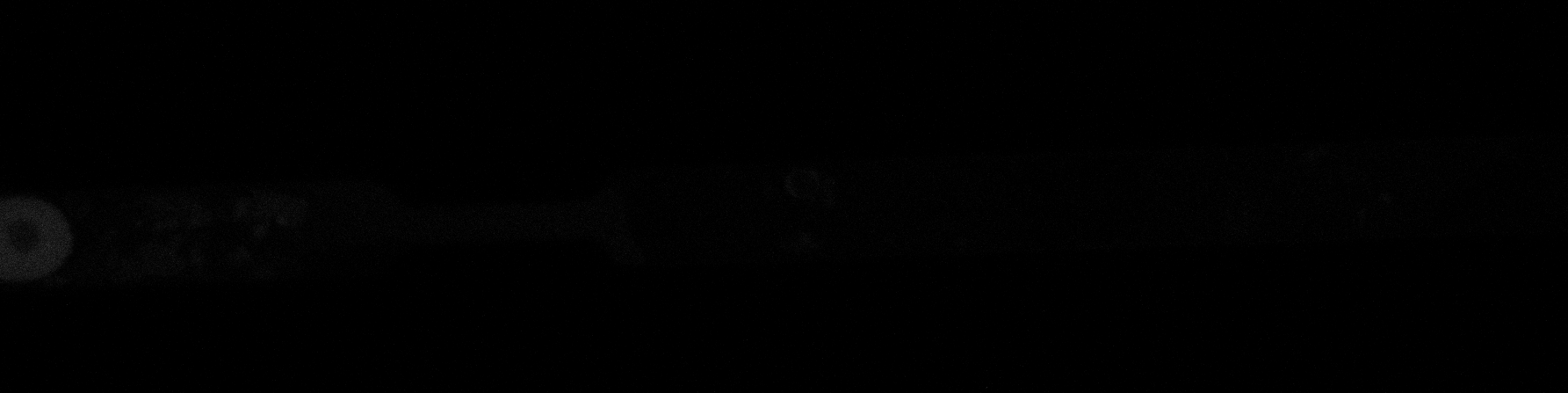

Supplement: Supplementary file 17 — Source data Fig. 3 [file 44318_2025_566_MOESM17_ESM.zip › Fig 3/Fig 3D/R62D-NLS/R62D-NLS_550min_NLS-BFP-Actin-R62D.tif]

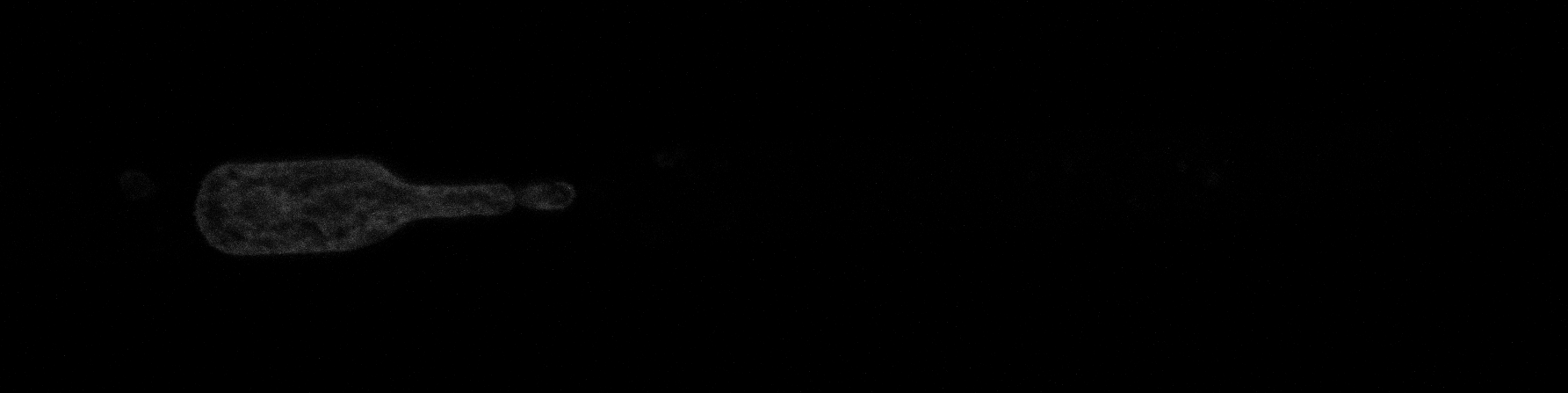

Supplement: Supplementary file 17 — Source data Fig. 3 [file 44318_2025_566_MOESM17_ESM.zip › Fig 3/Fig 3D/R62D-NLS/R62D-NLS_70min_H2B-mCherry.tif]

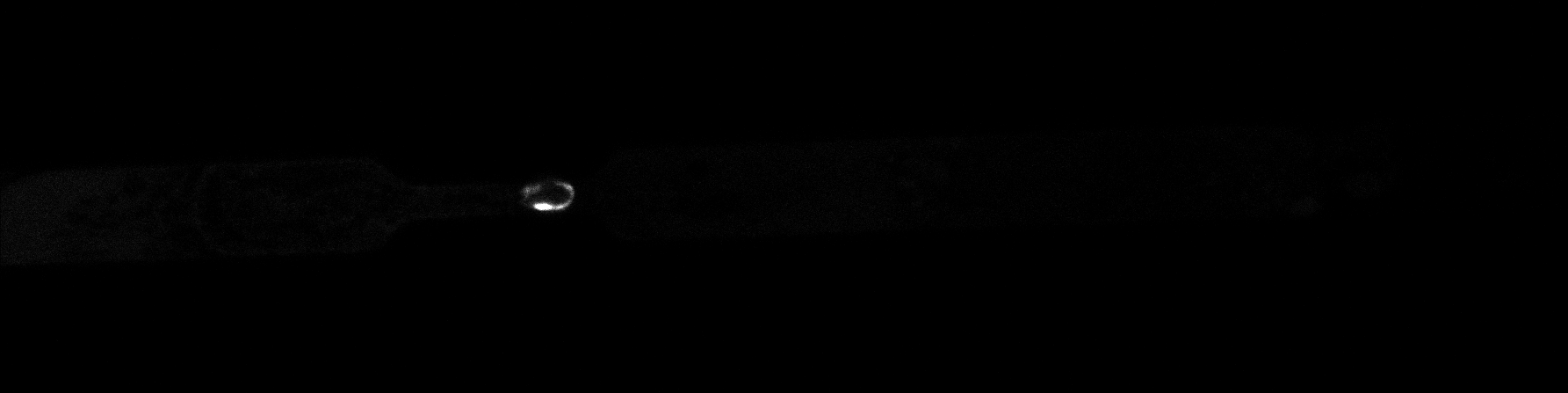

Supplement: Supplementary file 17 — Source data Fig. 3 [file 44318_2025_566_MOESM17_ESM.zip › Fig 3/Fig 3D/R62D-NLS/R62D-NLS_70min_icGAS-GFP.tif]

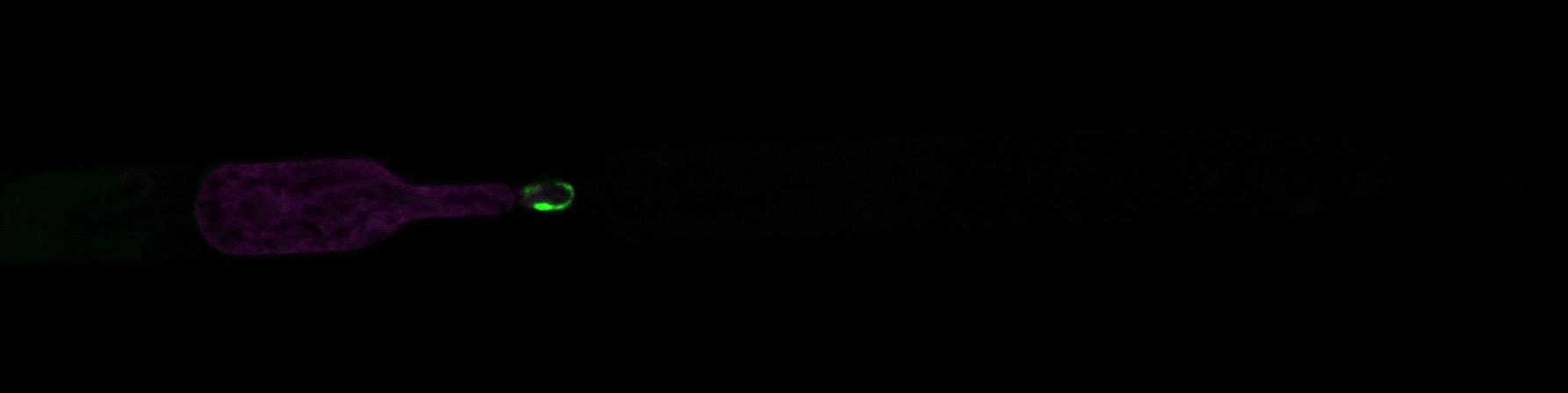

Supplement: Supplementary file 17 — Source data Fig. 3 [file 44318_2025_566_MOESM17_ESM.zip › Fig 3/Fig 3D/R62D-NLS/R62D-NLS_70min_Merge.tif]

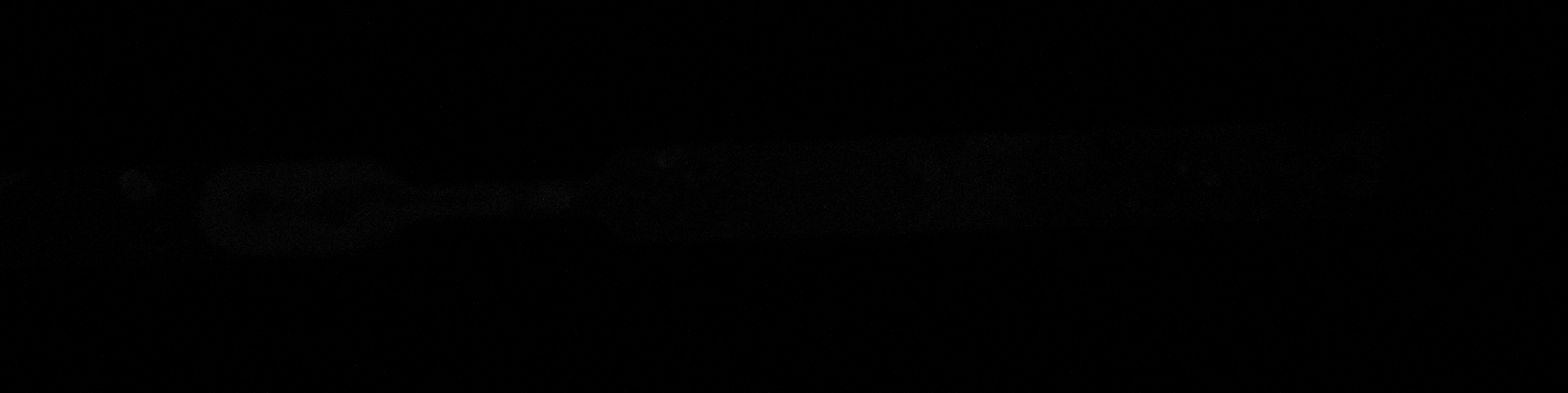

Supplement: Supplementary file 17 — Source data Fig. 3 [file 44318_2025_566_MOESM17_ESM.zip › Fig 3/Fig 3D/R62D-NLS/R62D-NLS_70min_NLS-BFP-Actin-R62D.tif]

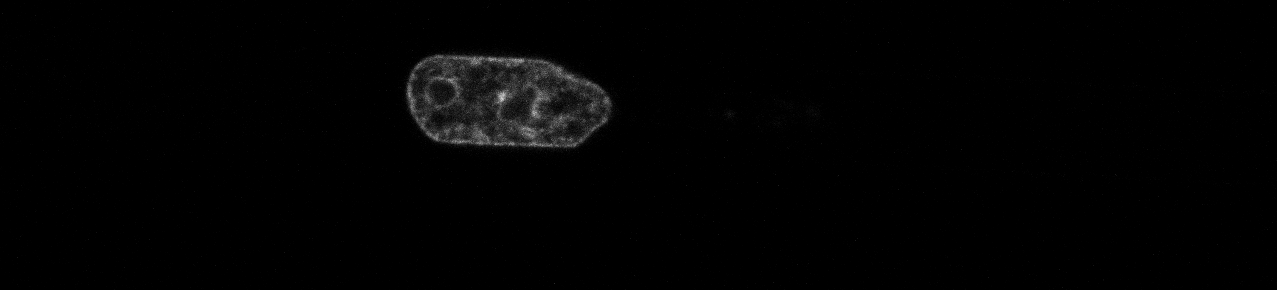

Supplement: Supplementary file 17 — Source data Fig. 3 [file 44318_2025_566_MOESM17_ESM.zip › Fig 3/Fig 3F/siCtrl/siCtrl_0min_H2B-mCherry.tif]

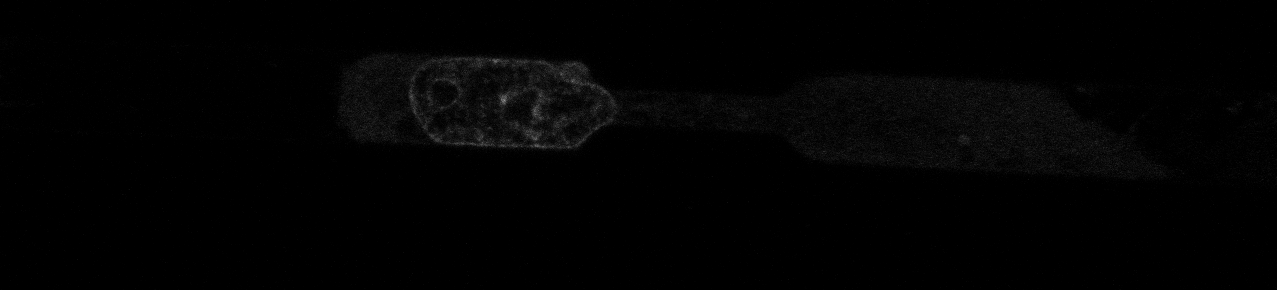

Supplement: Supplementary file 17 — Source data Fig. 3 [file 44318_2025_566_MOESM17_ESM.zip › Fig 3/Fig 3F/siCtrl/siCtrl_0min_icGAS-GFP.tif]

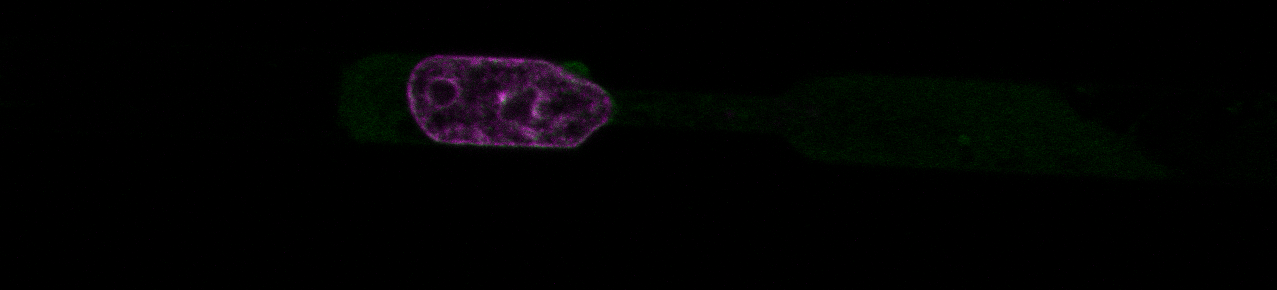

Supplement: Supplementary file 17 — Source data Fig. 3 [file 44318_2025_566_MOESM17_ESM.zip › Fig 3/Fig 3F/siCtrl/siCtrl_0min_Merge.tif]

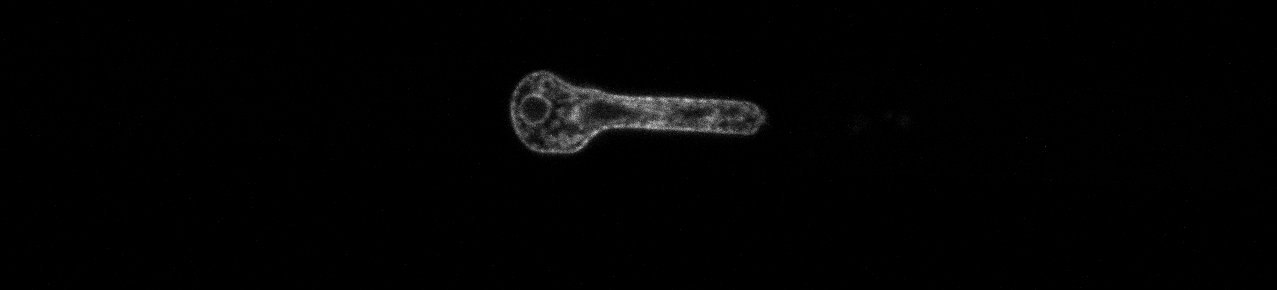

Supplement: Supplementary file 17 — Source data Fig. 3 [file 44318_2025_566_MOESM17_ESM.zip › Fig 3/Fig 3F/siCtrl/siCtrl_10min_H2B-mCherry.tif]

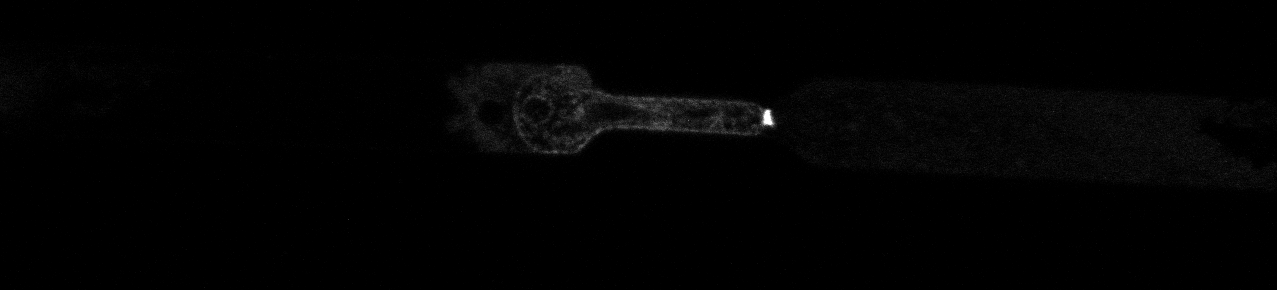

Supplement: Supplementary file 17 — Source data Fig. 3 [file 44318_2025_566_MOESM17_ESM.zip › Fig 3/Fig 3F/siCtrl/siCtrl_10min_icGAS-GFP.tif]

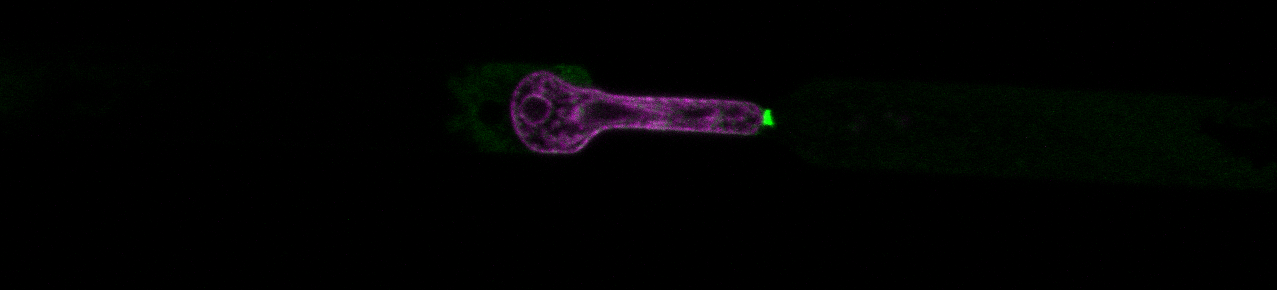

Supplement: Supplementary file 17 — Source data Fig. 3 [file 44318_2025_566_MOESM17_ESM.zip › Fig 3/Fig 3F/siCtrl/siCtrl_10min_Merge.tif]

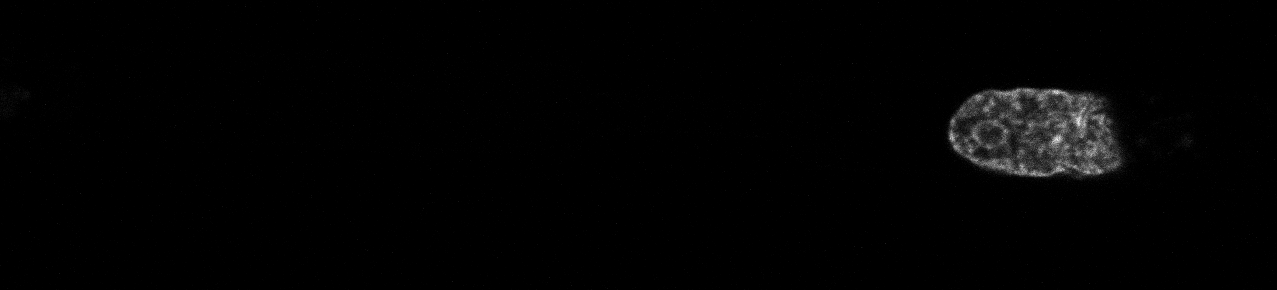

Supplement: Supplementary file 17 — Source data Fig. 3 [file 44318_2025_566_MOESM17_ESM.zip › Fig 3/Fig 3F/siCtrl/siCtrl_30min_H2B-mCherry.tif]

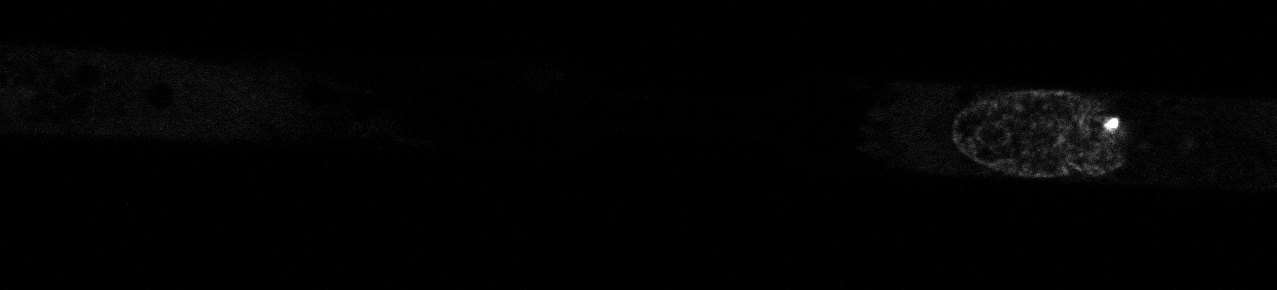

Supplement: Supplementary file 17 — Source data Fig. 3 [file 44318_2025_566_MOESM17_ESM.zip › Fig 3/Fig 3F/siCtrl/siCtrl_30min_icGAS-GFP.tif]

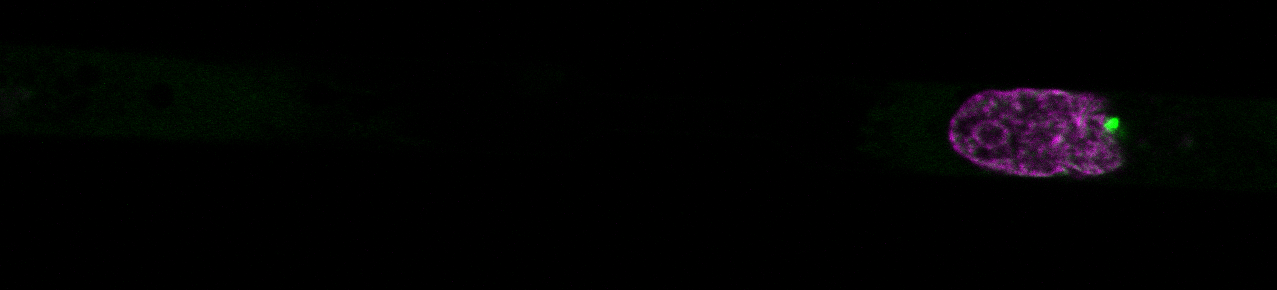

Supplement: Supplementary file 17 — Source data Fig. 3 [file 44318_2025_566_MOESM17_ESM.zip › Fig 3/Fig 3F/siCtrl/siCtrl_30min_Merge.tif]

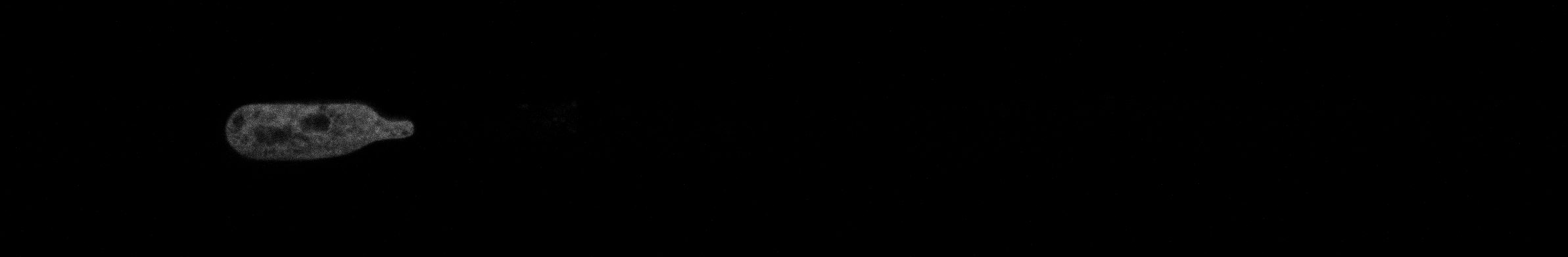

Supplement: Supplementary file 17 — Source data Fig. 3 [file 44318_2025_566_MOESM17_ESM.zip › Fig 3/Fig 3F/siDIAPH1_3/siDIAPH1_3_0min_H2B-mCherry.tif]

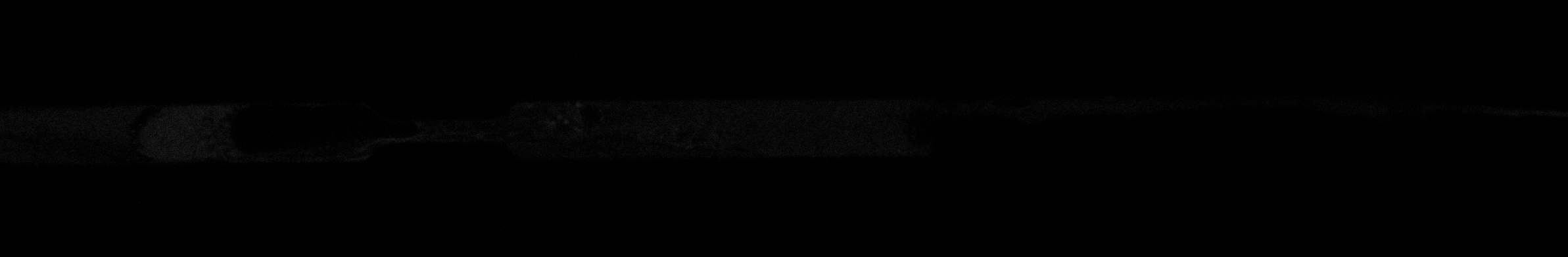

Supplement: Supplementary file 17 — Source data Fig. 3 [file 44318_2025_566_MOESM17_ESM.zip › Fig 3/Fig 3F/siDIAPH1_3/siDIAPH1_3_0min_icGAS-GFP.tif]

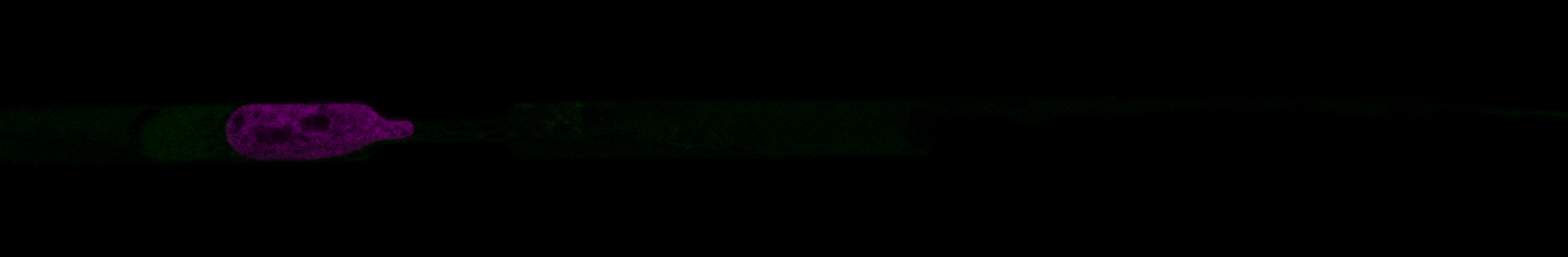

Supplement: Supplementary file 17 — Source data Fig. 3 [file 44318_2025_566_MOESM17_ESM.zip › Fig 3/Fig 3F/siDIAPH1_3/siDIAPH1_3_0min_Merge.tif]

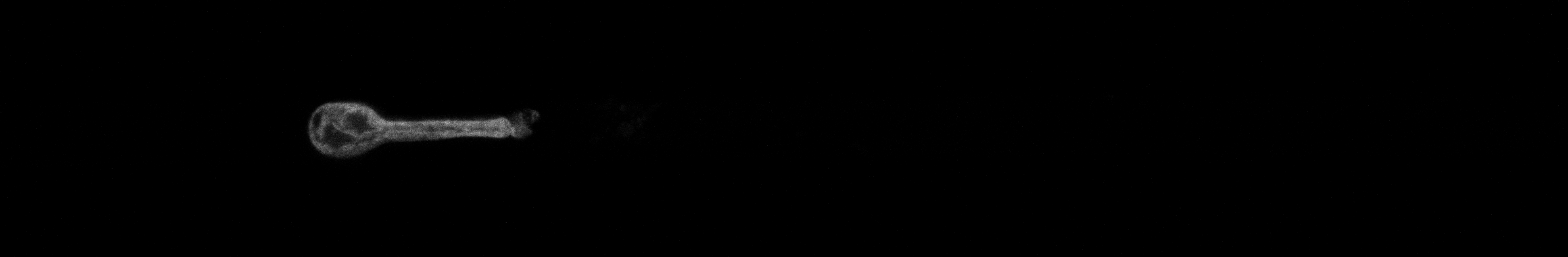

Supplement: Supplementary file 17 — Source data Fig. 3 [file 44318_2025_566_MOESM17_ESM.zip › Fig 3/Fig 3F/siDIAPH1_3/siDIAPH1_3_10min_H2B-mCherry.tif]

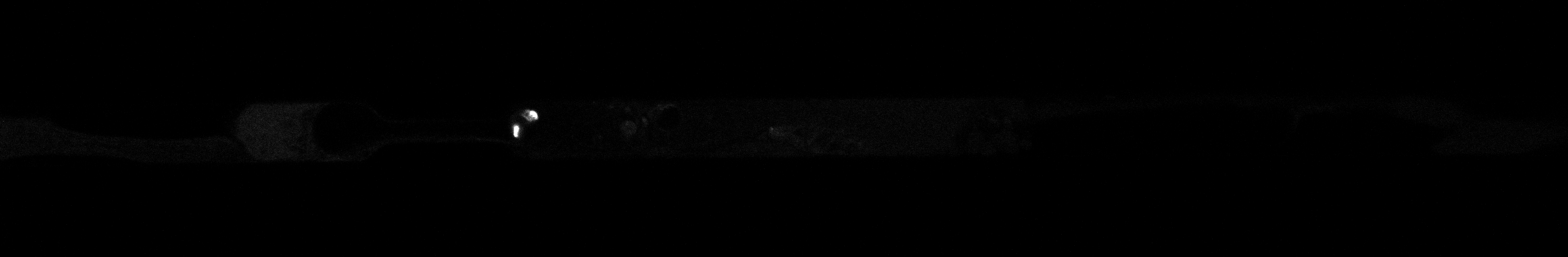

Supplement: Supplementary file 17 — Source data Fig. 3 [file 44318_2025_566_MOESM17_ESM.zip › Fig 3/Fig 3F/siDIAPH1_3/siDIAPH1_3_10min_icGAS-GFP.tif]

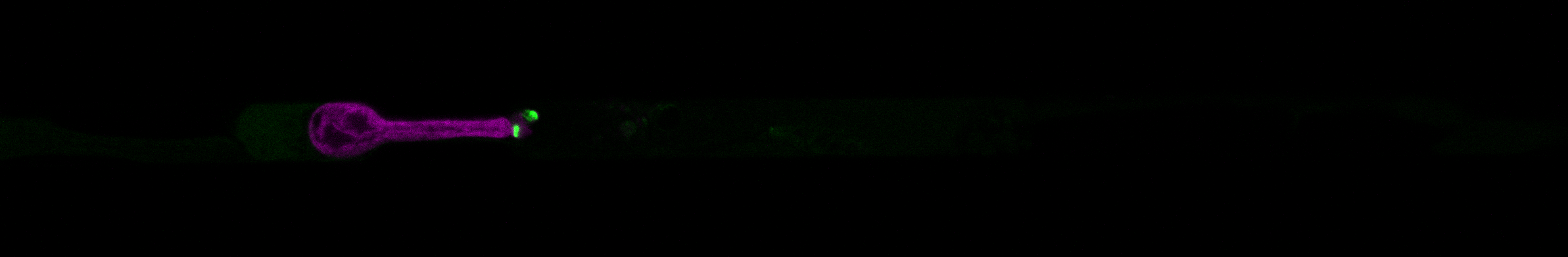

Supplement: Supplementary file 17 — Source data Fig. 3 [file 44318_2025_566_MOESM17_ESM.zip › Fig 3/Fig 3F/siDIAPH1_3/siDIAPH1_3_10min_Merge.tif]

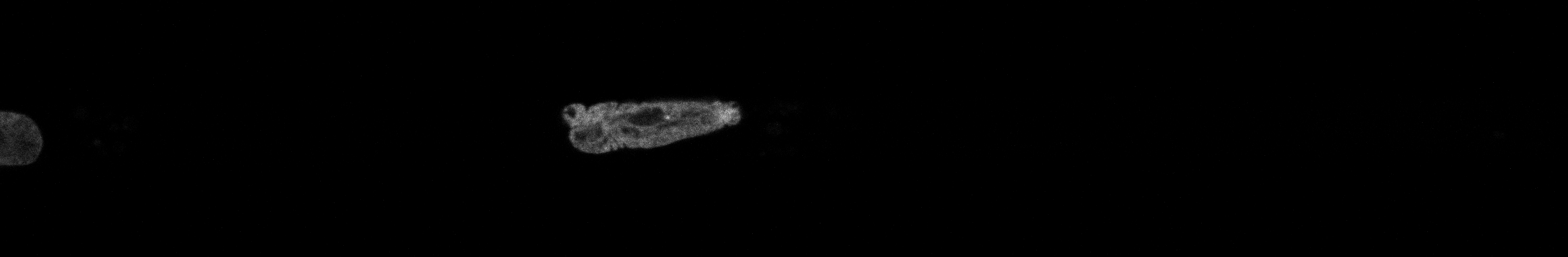

Supplement: Supplementary file 17 — Source data Fig. 3 [file 44318_2025_566_MOESM17_ESM.zip › Fig 3/Fig 3F/siDIAPH1_3/siDIAPH1_3_40min_H2B-mCherry.tif]

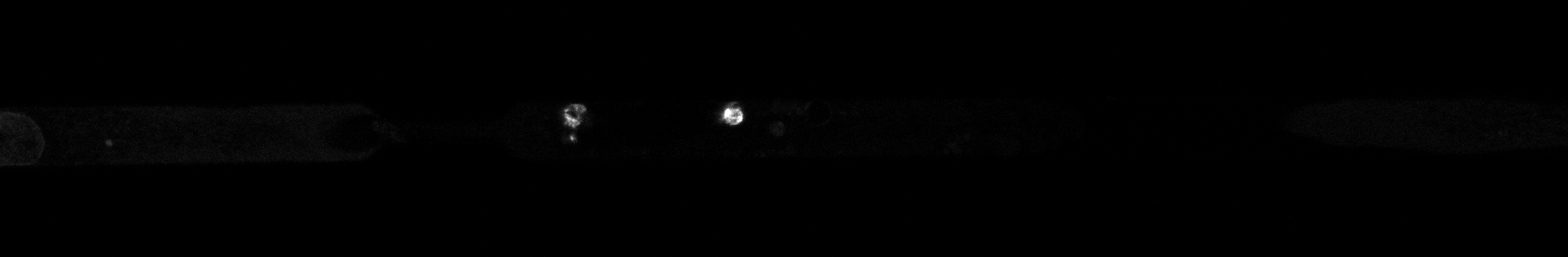

Supplement: Supplementary file 17 — Source data Fig. 3 [file 44318_2025_566_MOESM17_ESM.zip › Fig 3/Fig 3F/siDIAPH1_3/siDIAPH1_3_40min_icGAS-GFP.tif]

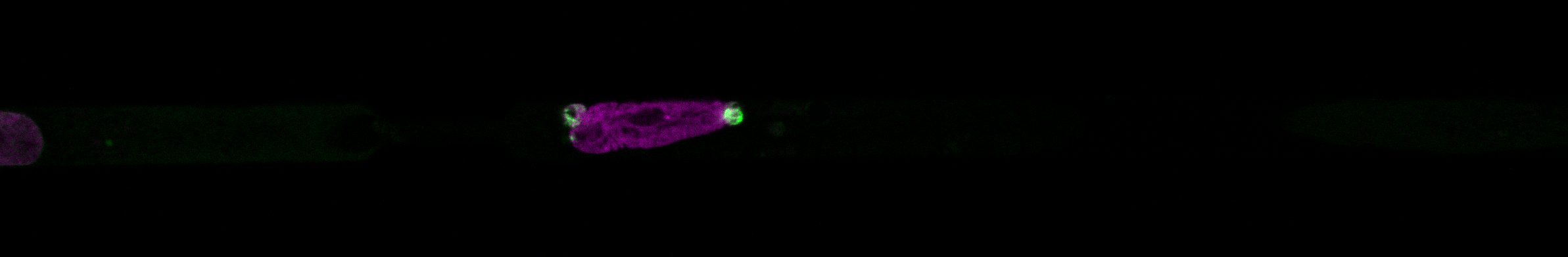

Supplement: Supplementary file 17 — Source data Fig. 3 [file 44318_2025_566_MOESM17_ESM.zip › Fig 3/Fig 3F/siDIAPH1_3/siDIAPH1_3_40min_Merge.tif]

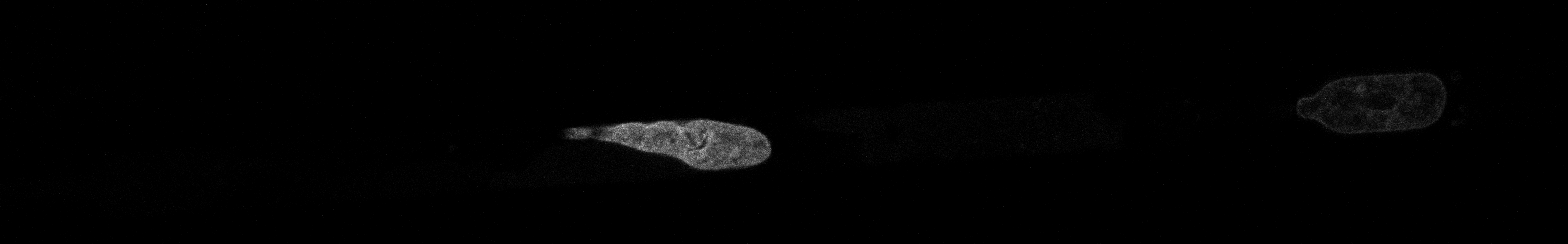

Supplement: Supplementary file 17 — Source data Fig. 3 [file 44318_2025_566_MOESM17_ESM.zip › Fig 3/Fig 3H/NLS-BFP/NLS-BFP_0min_H2B-mCherry.tif]

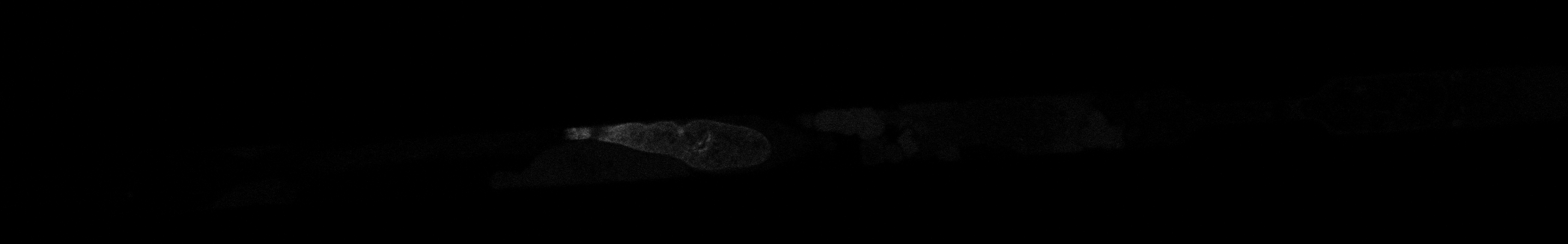

Supplement: Supplementary file 17 — Source data Fig. 3 [file 44318_2025_566_MOESM17_ESM.zip › Fig 3/Fig 3H/NLS-BFP/NLS-BFP_0min_icGAS-GFP.tif]

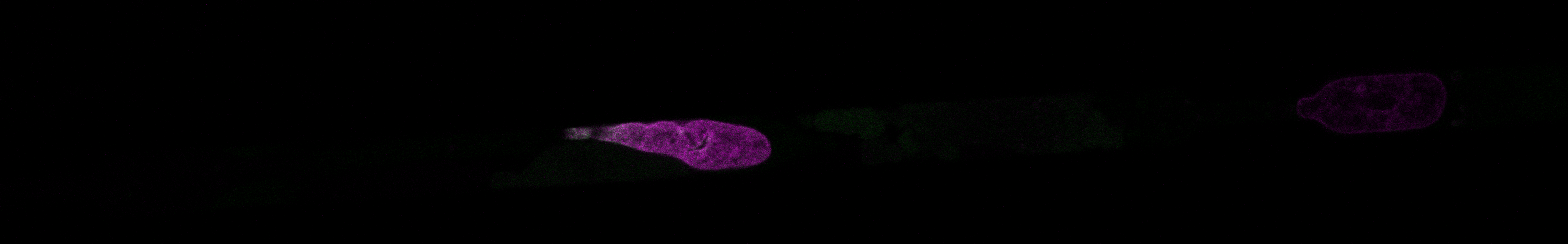

Supplement: Supplementary file 17 — Source data Fig. 3 [file 44318_2025_566_MOESM17_ESM.zip › Fig 3/Fig 3H/NLS-BFP/NLS-BFP_0min_Merge.tif]

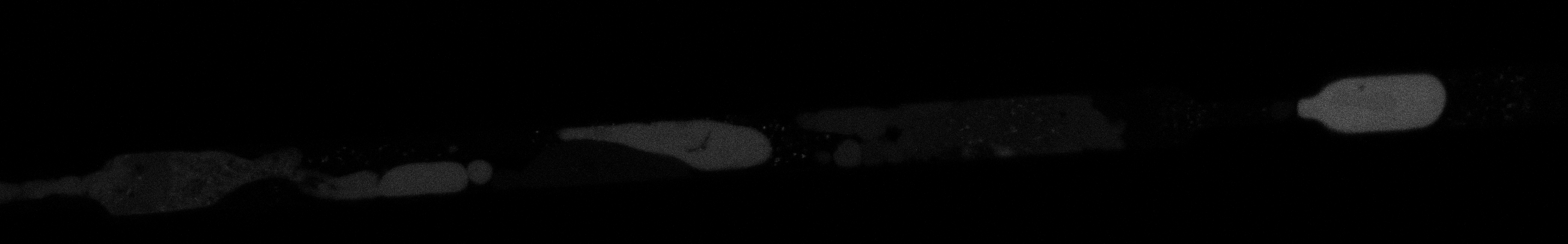

Supplement: Supplementary file 17 — Source data Fig. 3 [file 44318_2025_566_MOESM17_ESM.zip › Fig 3/Fig 3H/NLS-BFP/NLS-BFP_0min_NLS-BFP.tif]

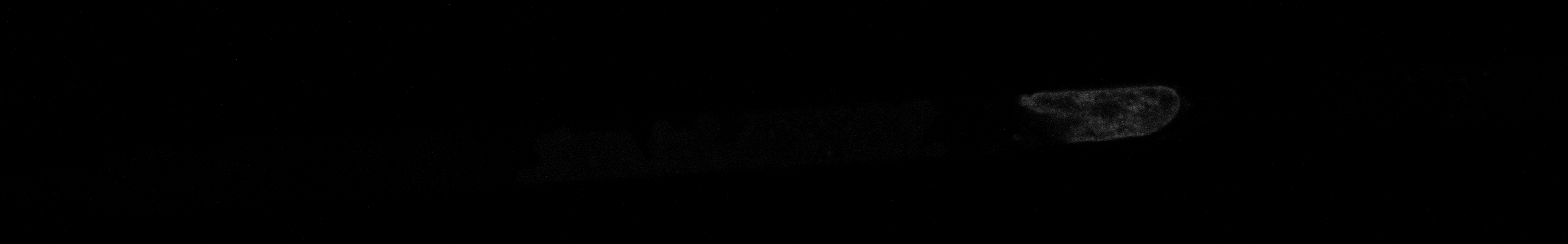

Supplement: Supplementary file 17 — Source data Fig. 3 [file 44318_2025_566_MOESM17_ESM.zip › Fig 3/Fig 3H/NLS-BFP/NLS-BFP_100min_H2B-mCherry.tif]

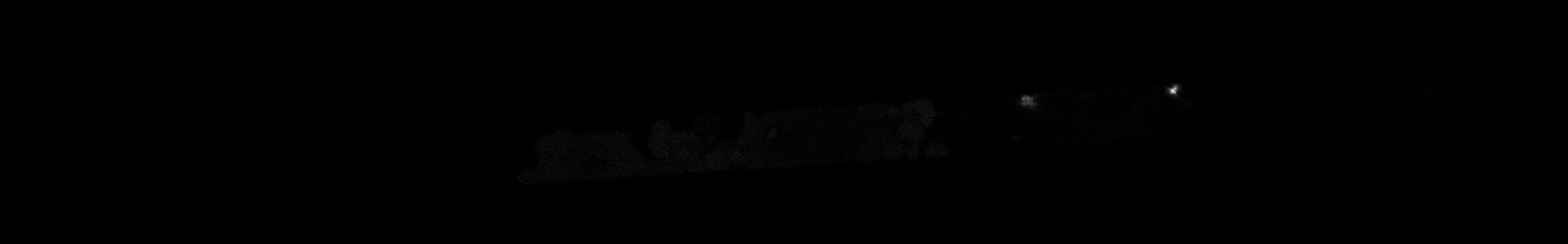

Supplement: Supplementary file 17 — Source data Fig. 3 [file 44318_2025_566_MOESM17_ESM.zip › Fig 3/Fig 3H/NLS-BFP/NLS-BFP_100min_icGAS-GFP.tif]

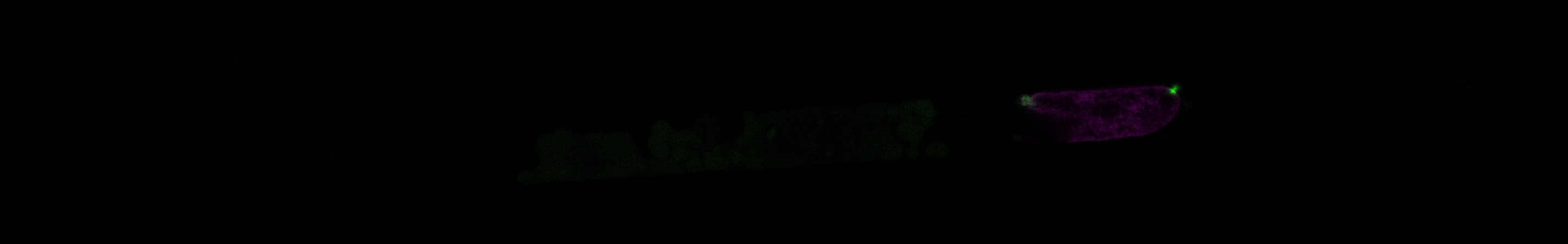

Supplement: Supplementary file 17 — Source data Fig. 3 [file 44318_2025_566_MOESM17_ESM.zip › Fig 3/Fig 3H/NLS-BFP/NLS-BFP_100min_Merge.tif]

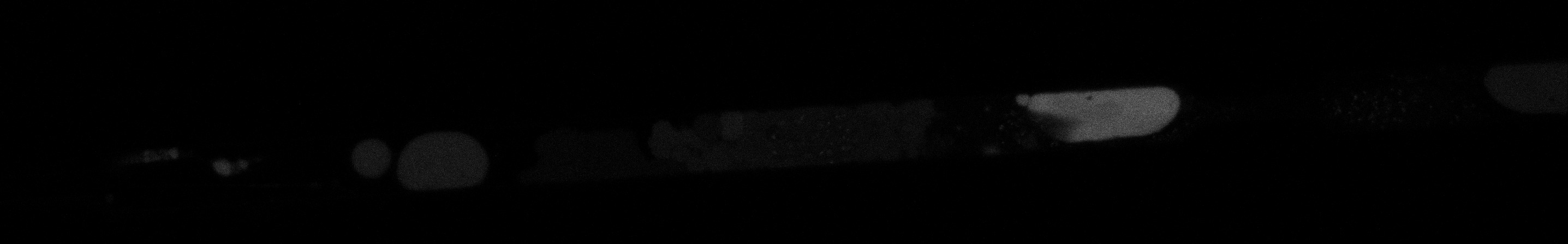

Supplement: Supplementary file 17 — Source data Fig. 3 [file 44318_2025_566_MOESM17_ESM.zip › Fig 3/Fig 3H/NLS-BFP/NLS-BFP_100min_NLS-BFP.tif]

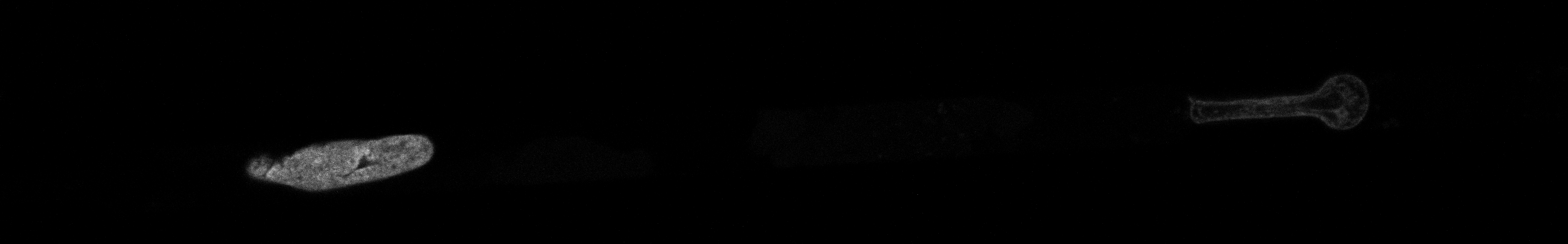

Supplement: Supplementary file 17 — Source data Fig. 3 [file 44318_2025_566_MOESM17_ESM.zip › Fig 3/Fig 3H/NLS-BFP/NLS-BFP_20min_H2B-mCherry.tif]

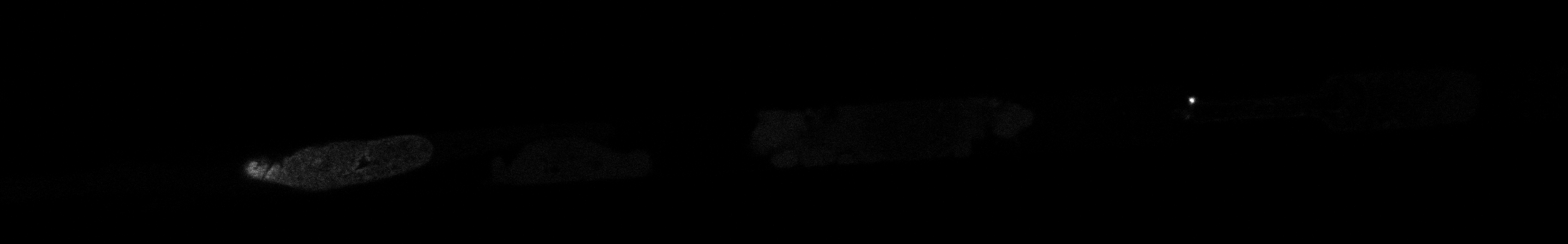

Supplement: Supplementary file 17 — Source data Fig. 3 [file 44318_2025_566_MOESM17_ESM.zip › Fig 3/Fig 3H/NLS-BFP/NLS-BFP_20min_icGAS-GFP.tif]

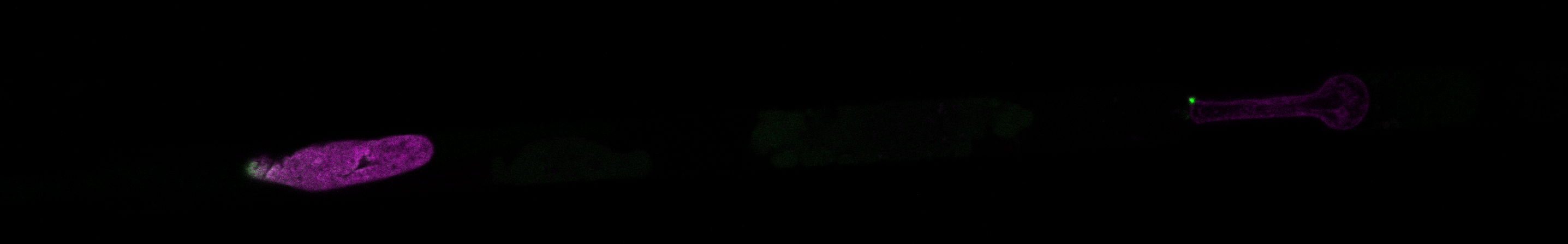

Supplement: Supplementary file 17 — Source data Fig. 3 [file 44318_2025_566_MOESM17_ESM.zip › Fig 3/Fig 3H/NLS-BFP/NLS-BFP_20min_Merge.tif]

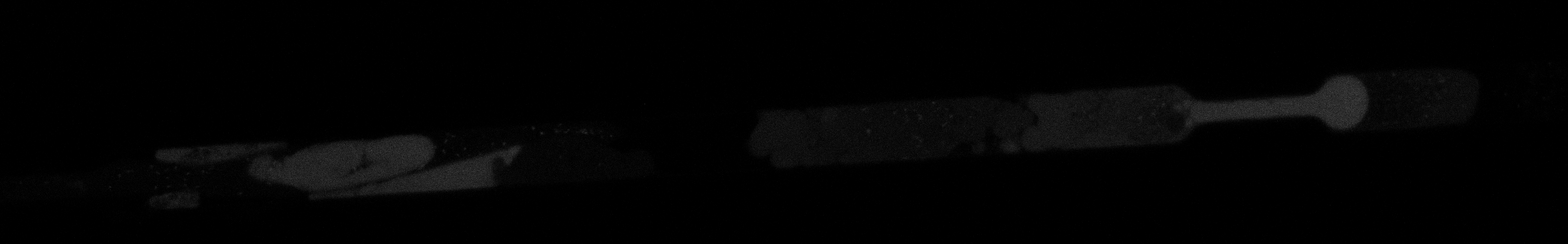

Supplement: Supplementary file 17 — Source data Fig. 3 [file 44318_2025_566_MOESM17_ESM.zip › Fig 3/Fig 3H/NLS-BFP/NLS-BFP_20min_NLS-BFP.tif]

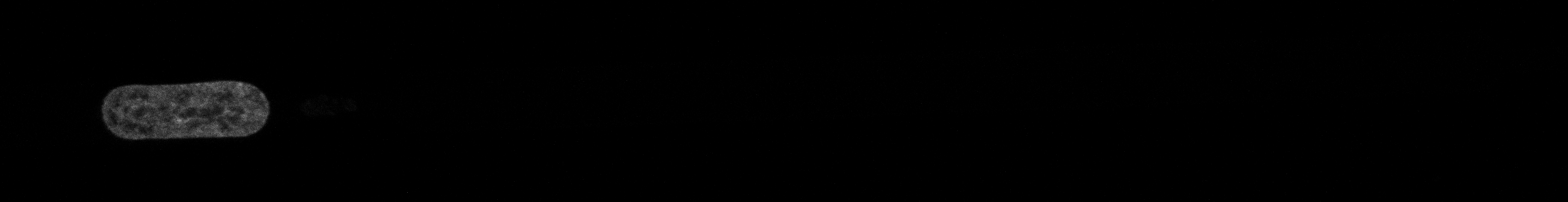

Supplement: Supplementary file 17 — Source data Fig. 3 [file 44318_2025_566_MOESM17_ESM.zip › Fig 3/Fig 3H/R62D-NLS/R62D-NLS_0min_H2B-mCherry.tif]

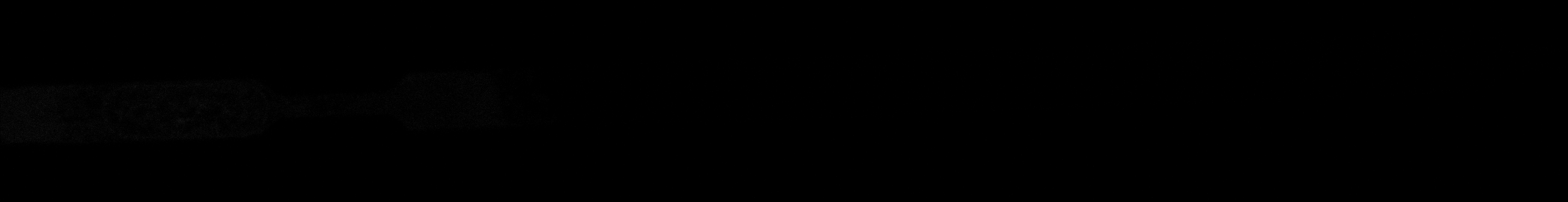

Supplement: Supplementary file 17 — Source data Fig. 3 [file 44318_2025_566_MOESM17_ESM.zip › Fig 3/Fig 3H/R62D-NLS/R62D-NLS_0min_icGAS-GFP.tif]

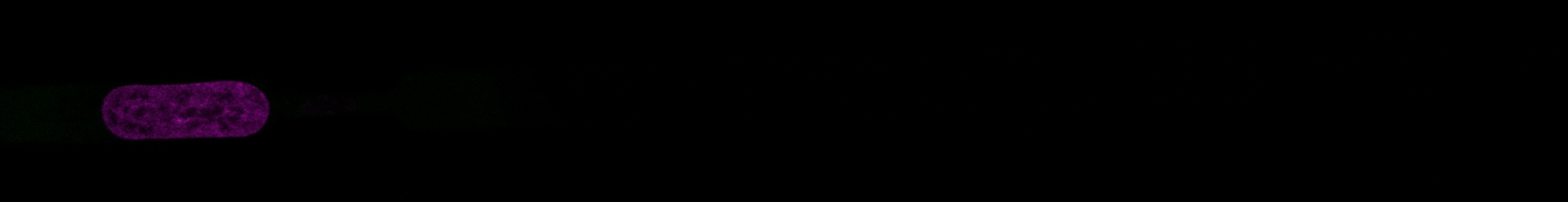

Supplement: Supplementary file 17 — Source data Fig. 3 [file 44318_2025_566_MOESM17_ESM.zip › Fig 3/Fig 3H/R62D-NLS/R62D-NLS_0min_Merge.tif]

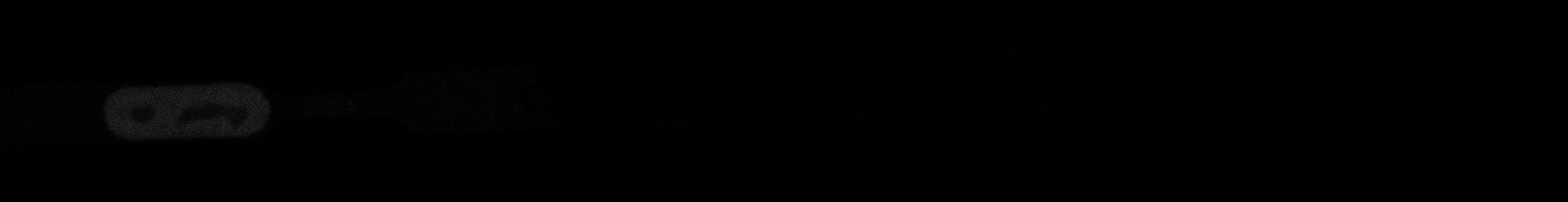

Supplement: Supplementary file 17 — Source data Fig. 3 [file 44318_2025_566_MOESM17_ESM.zip › Fig 3/Fig 3H/R62D-NLS/R62D-NLS_0min_NLS-BFP-Actin-R62D.tif]

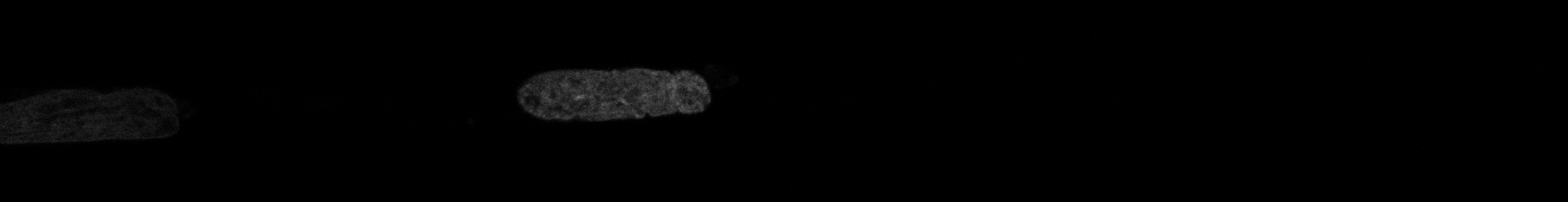

Supplement: Supplementary file 17 — Source data Fig. 3 [file 44318_2025_566_MOESM17_ESM.zip › Fig 3/Fig 3H/R62D-NLS/R62D-NLS_100min_H2B-mCherry.tif]

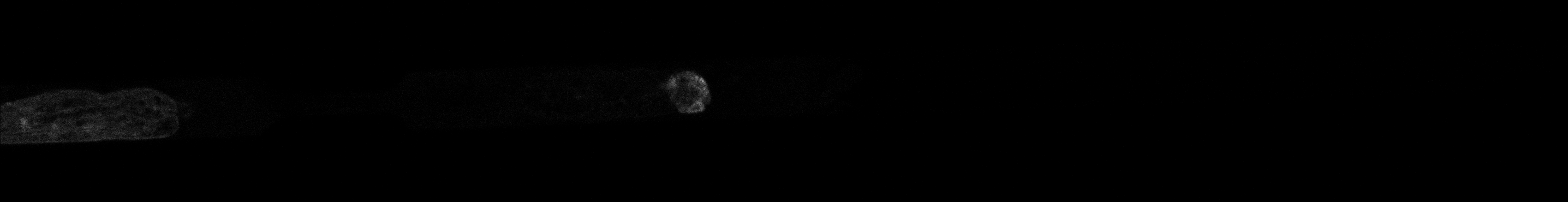

Supplement: Supplementary file 17 — Source data Fig. 3 [file 44318_2025_566_MOESM17_ESM.zip › Fig 3/Fig 3H/R62D-NLS/R62D-NLS_100min_icGAS-GFP.tif]

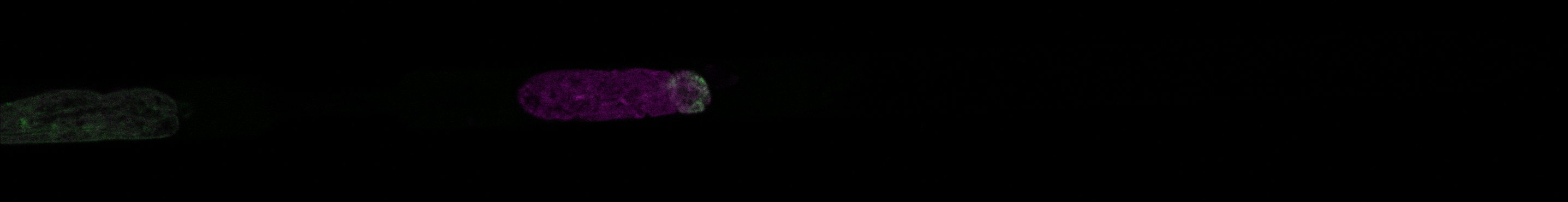

Supplement: Supplementary file 17 — Source data Fig. 3 [file 44318_2025_566_MOESM17_ESM.zip › Fig 3/Fig 3H/R62D-NLS/R62D-NLS_100min_Merge.tif]

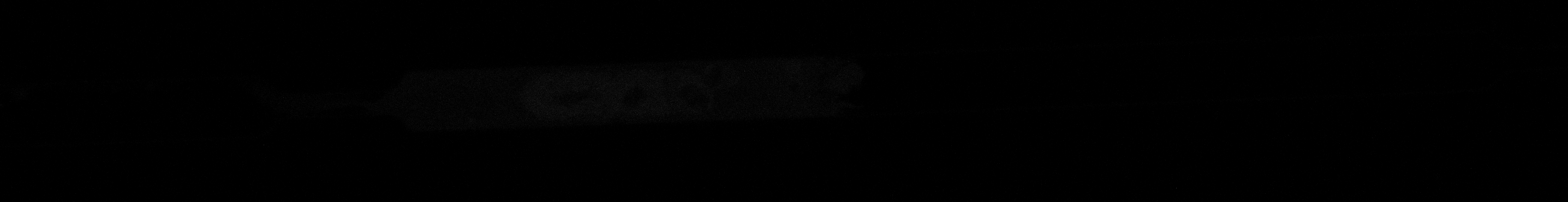

Supplement: Supplementary file 17 — Source data Fig. 3 [file 44318_2025_566_MOESM17_ESM.zip › Fig 3/Fig 3H/R62D-NLS/R62D-NLS_100min_NLS-BFP-Actin-R62D.tif]

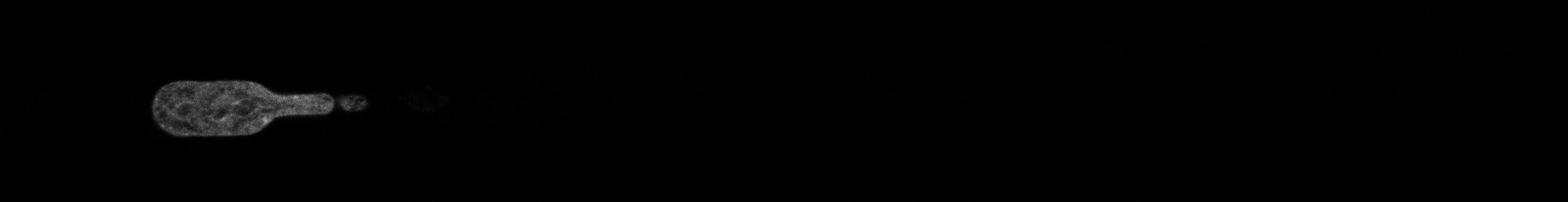

Supplement: Supplementary file 17 — Source data Fig. 3 [file 44318_2025_566_MOESM17_ESM.zip › Fig 3/Fig 3H/R62D-NLS/R62D-NLS_20min_H2B-mCherry.tif]

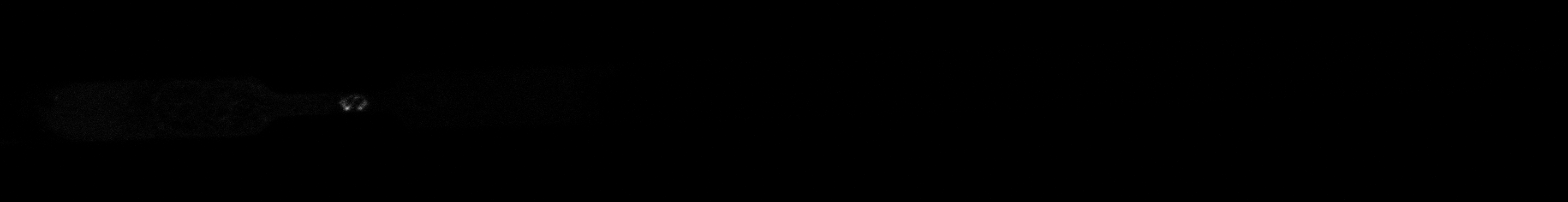

Supplement: Supplementary file 17 — Source data Fig. 3 [file 44318_2025_566_MOESM17_ESM.zip › Fig 3/Fig 3H/R62D-NLS/R62D-NLS_20min_icGAS-GFP.tif]
