# Supplementary figures and images for: Nuclear rupture in confined cell migration triggers nuclear actin polymerization to limit chromatin leakage (part 2 of 2)
Source: EMBO J. 2025 Sep 22;44(21):6112–36. doi: 10.1038/s44318-025-00566-2 (PMC12583611; doi:10.1038/s44318-025-00566-2)

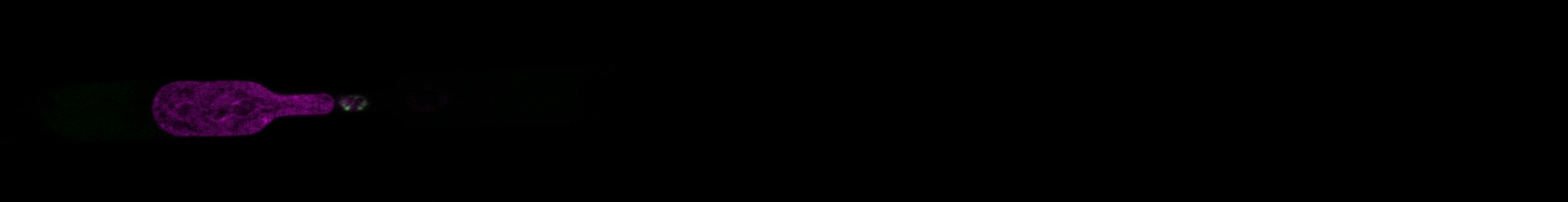

Supplement: Supplementary file 17 — Source data Fig. 3 [file 44318_2025_566_MOESM17_ESM.zip › Fig 3/Fig 3H/R62D-NLS/R62D-NLS_20min_Merge.tif]

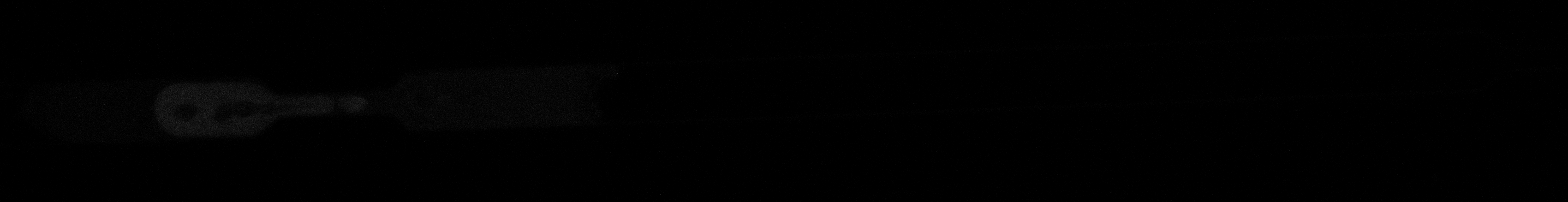

Supplement: Supplementary file 17 — Source data Fig. 3 [file 44318_2025_566_MOESM17_ESM.zip › Fig 3/Fig 3H/R62D-NLS/R62D-NLS_20min_NLS-BFP-Actin-R62D.tif]

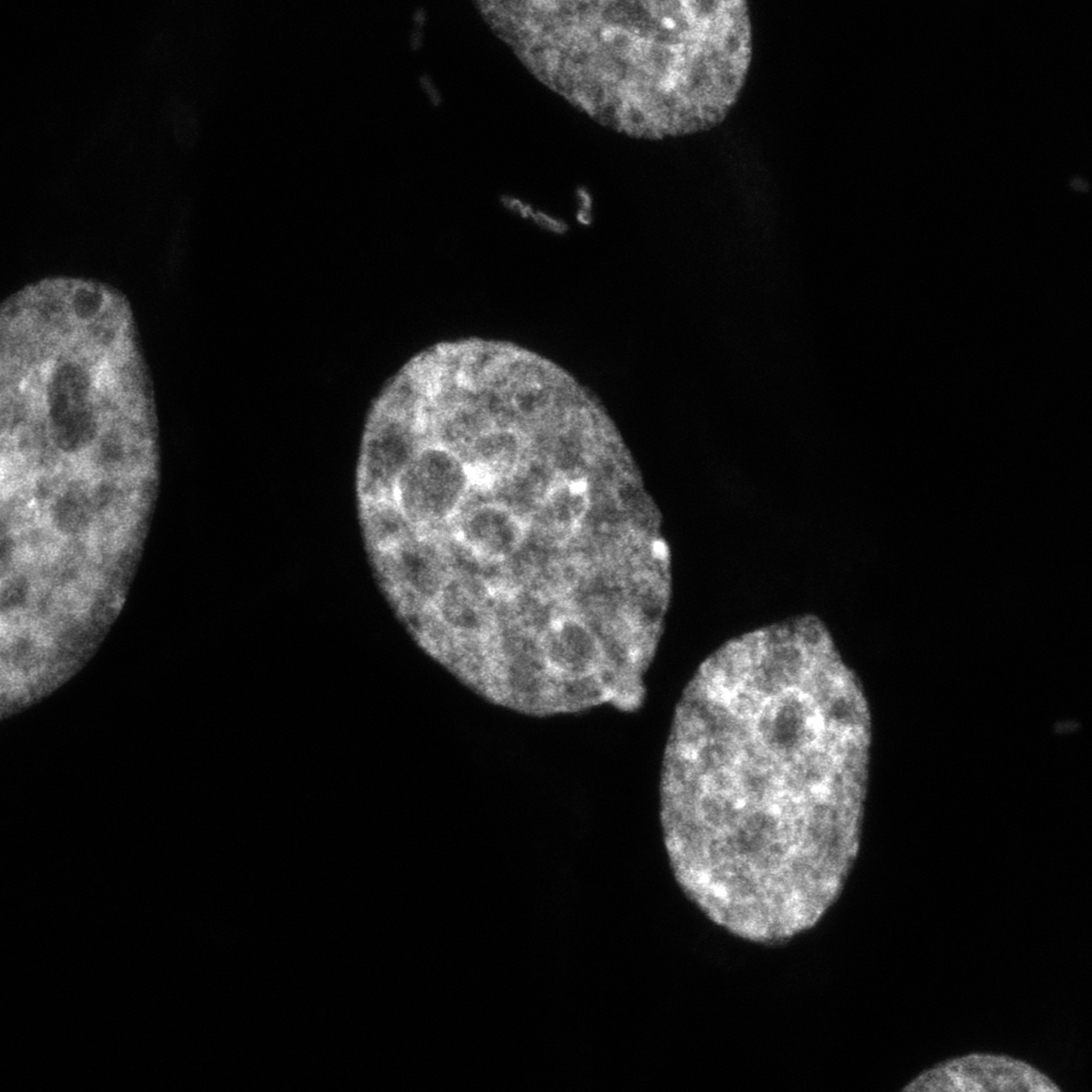

Supplement: Supplementary file 18 — Source data Fig. 4 [file 44318_2025_566_MOESM18_ESM.zip › Fig 4/Fig 4A/Fig 4A - siCtrl.tif]

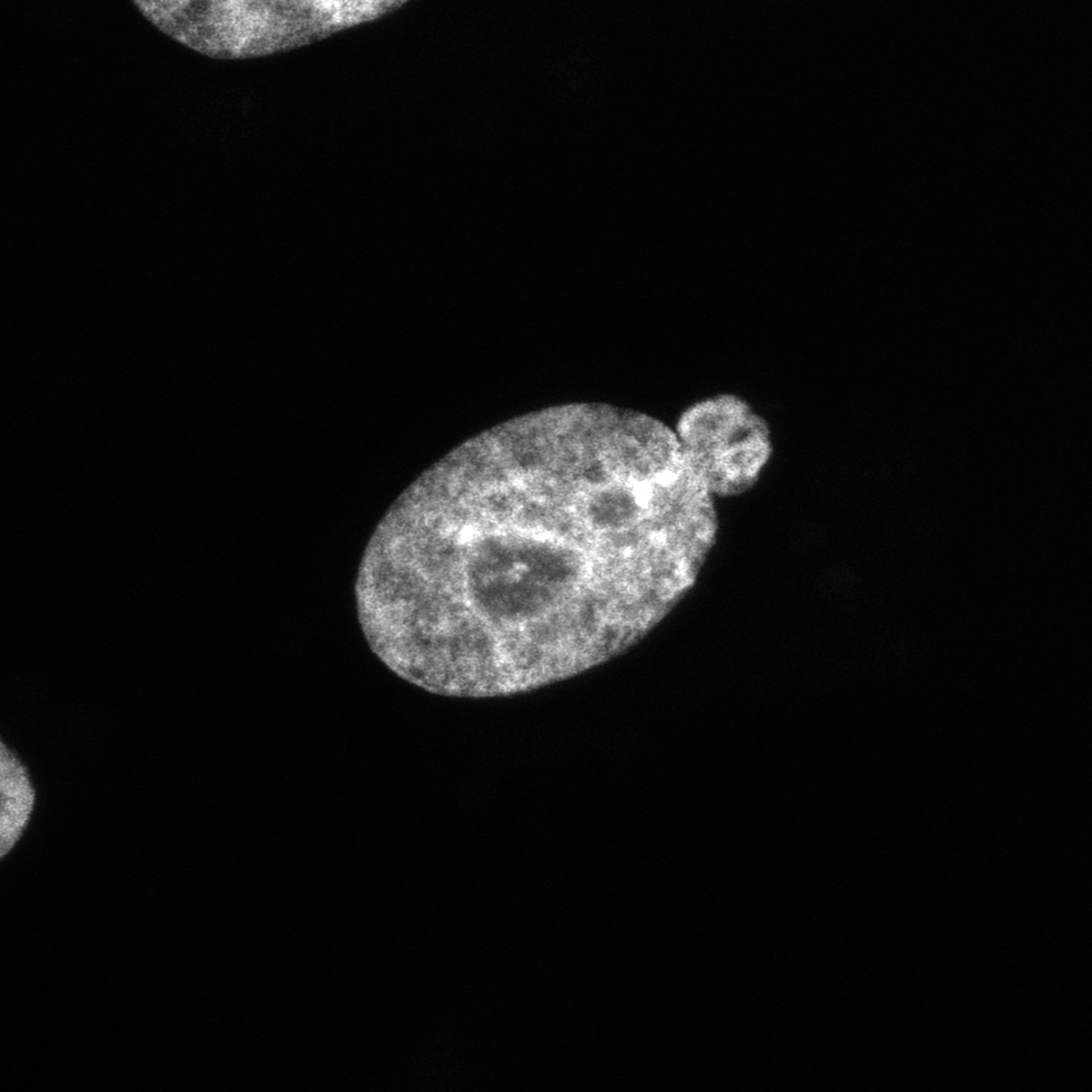

Supplement: Supplementary file 18 — Source data Fig. 4 [file 44318_2025_566_MOESM18_ESM.zip › Fig 4/Fig 4A/Fig 4A - siDIAPH1_3.tif]

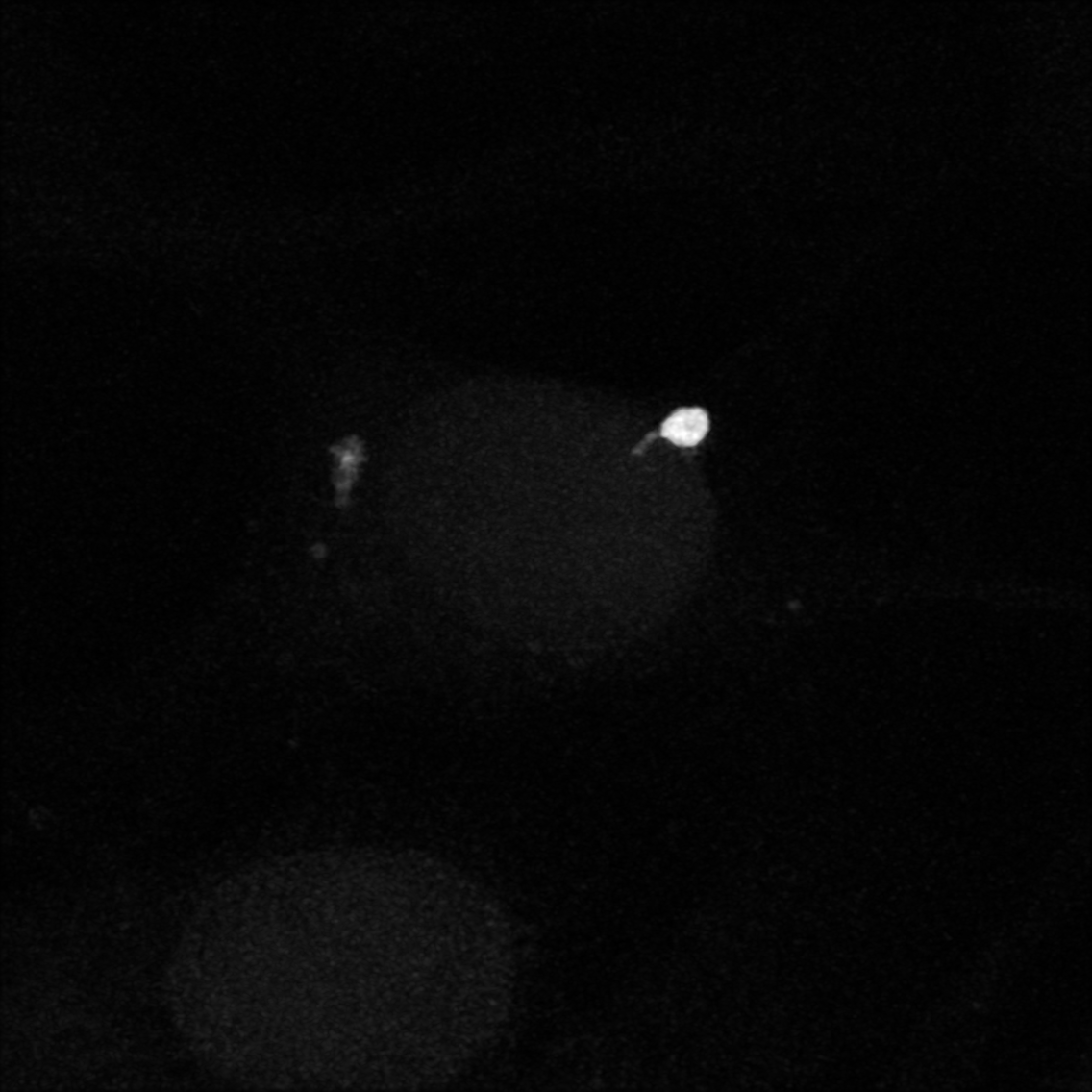

Supplement: Supplementary file 18 — Source data Fig. 4 [file 44318_2025_566_MOESM18_ESM.zip › Fig 4/Fig 4D/Fig 4D - NLS-Actin.tif]

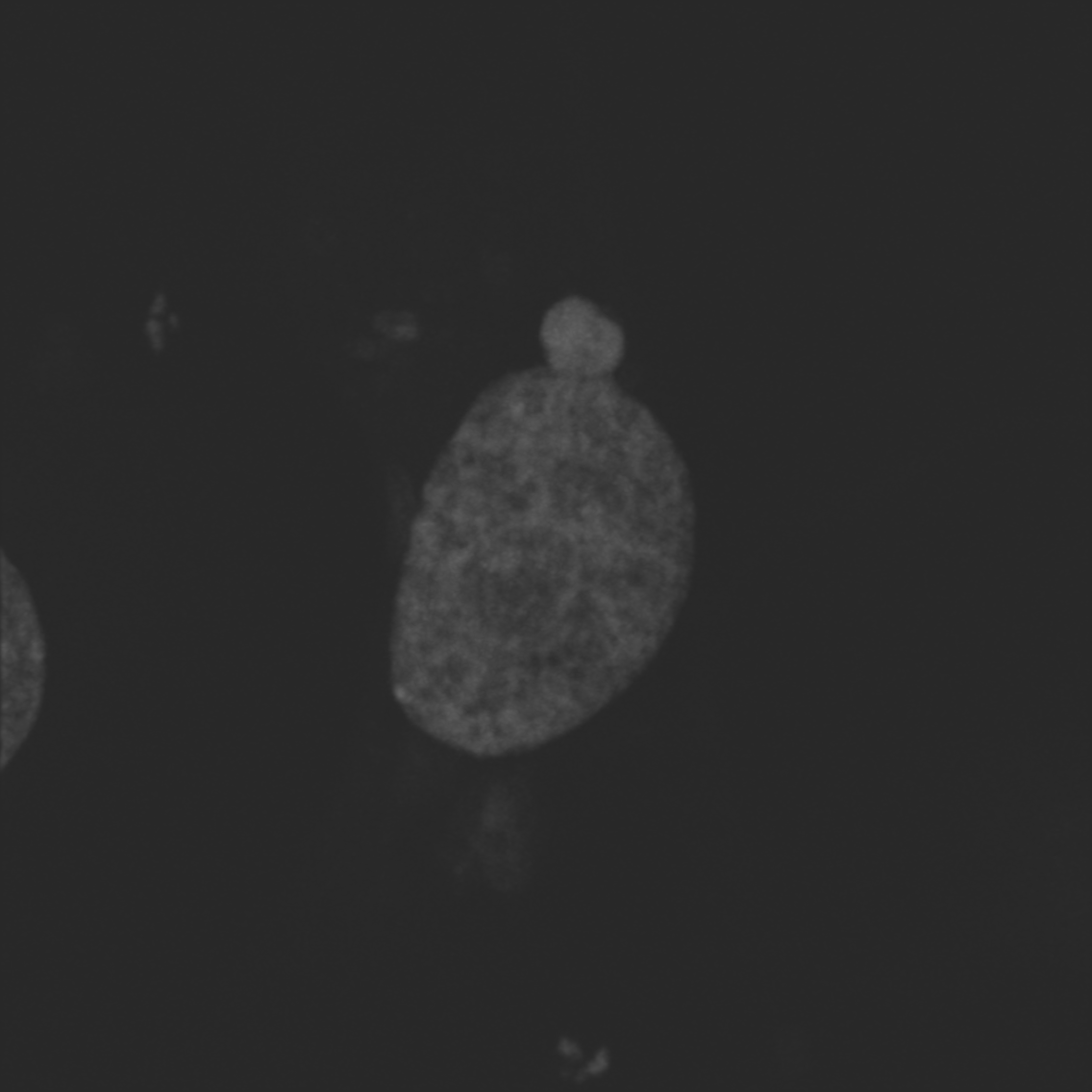

Supplement: Supplementary file 18 — Source data Fig. 4 [file 44318_2025_566_MOESM18_ESM.zip › Fig 4/Fig 4D/Fig 4D - R62D-NLS.tif]

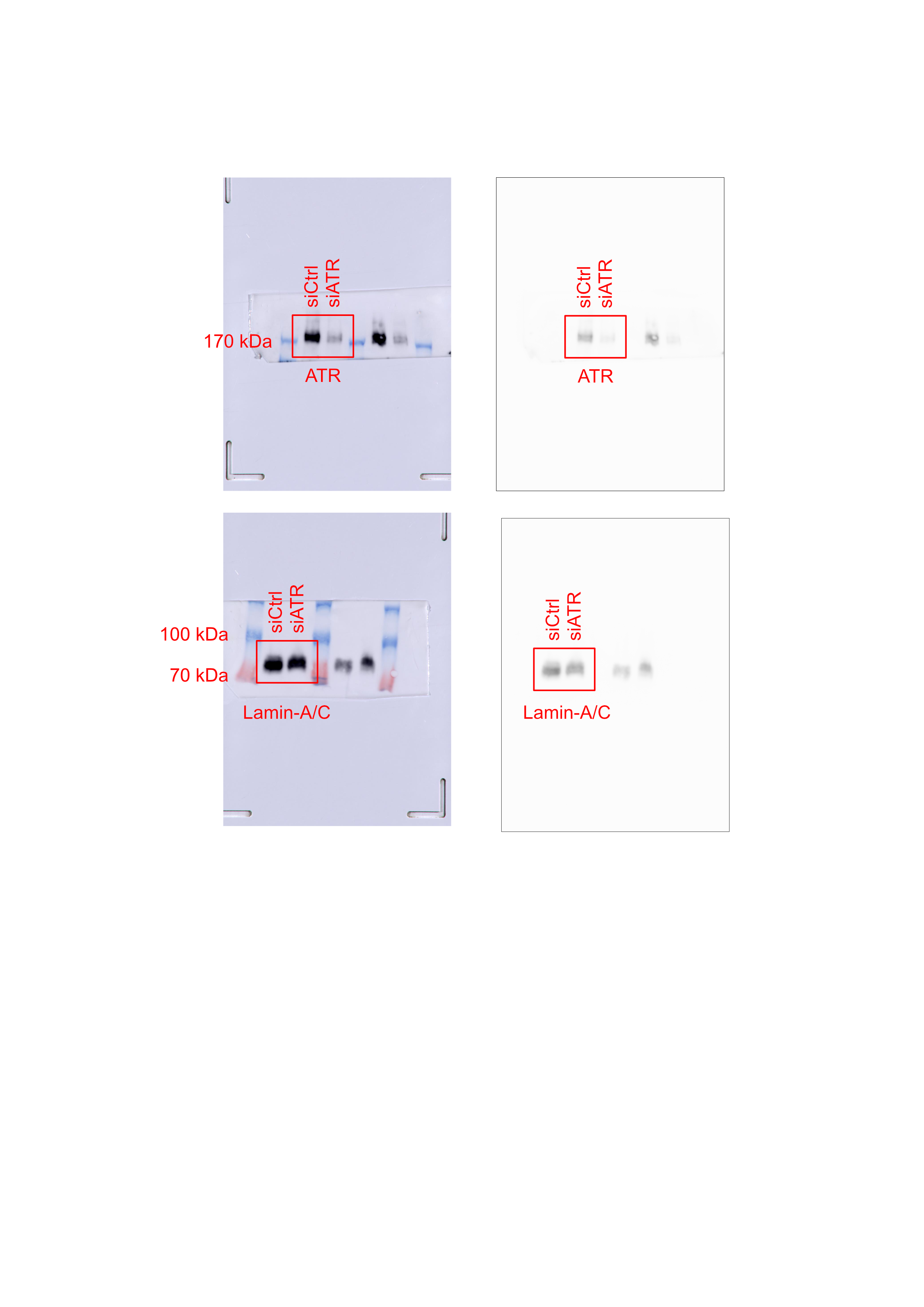

Supplement: Supplementary file 19 — Source data Fig. 5 [file 44318_2025_566_MOESM19_ESM.zip › Fig 5/Fig 5A/siATR.tiff]

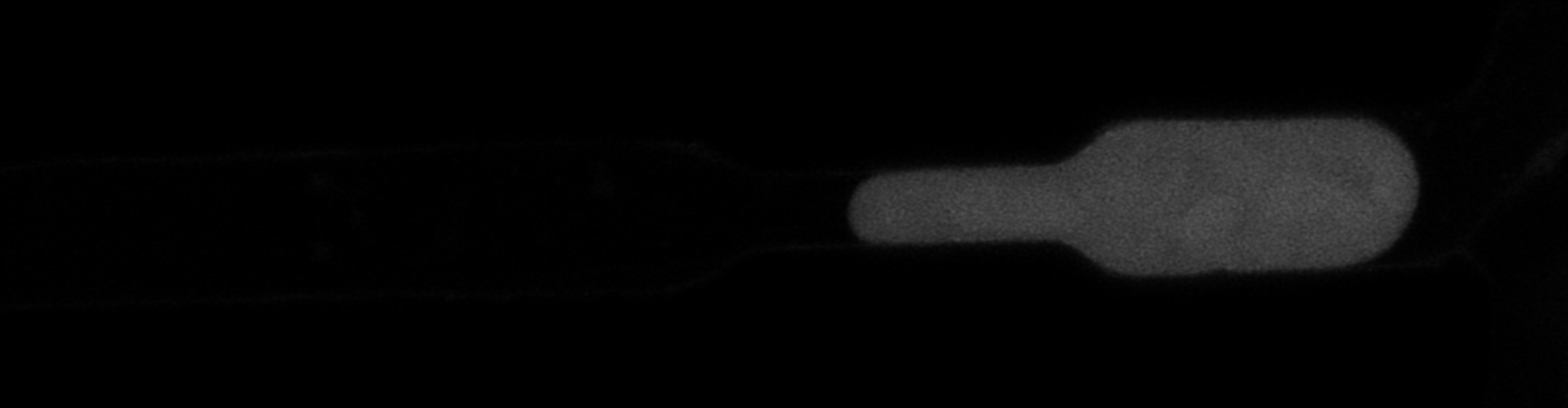

Supplement: Supplementary file 19 — Source data Fig. 5 [file 44318_2025_566_MOESM19_ESM.zip › Fig 5/Fig 5B/siATR_Before NE rupture.tif]

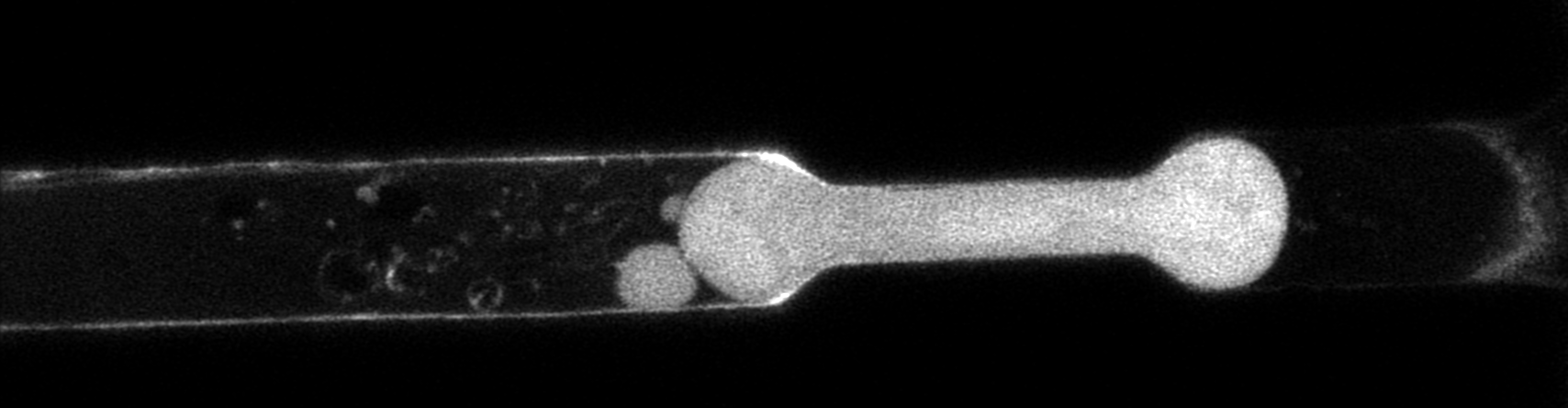

Supplement: Supplementary file 19 — Source data Fig. 5 [file 44318_2025_566_MOESM19_ESM.zip › Fig 5/Fig 5B/siATR_NE rupture.tif]

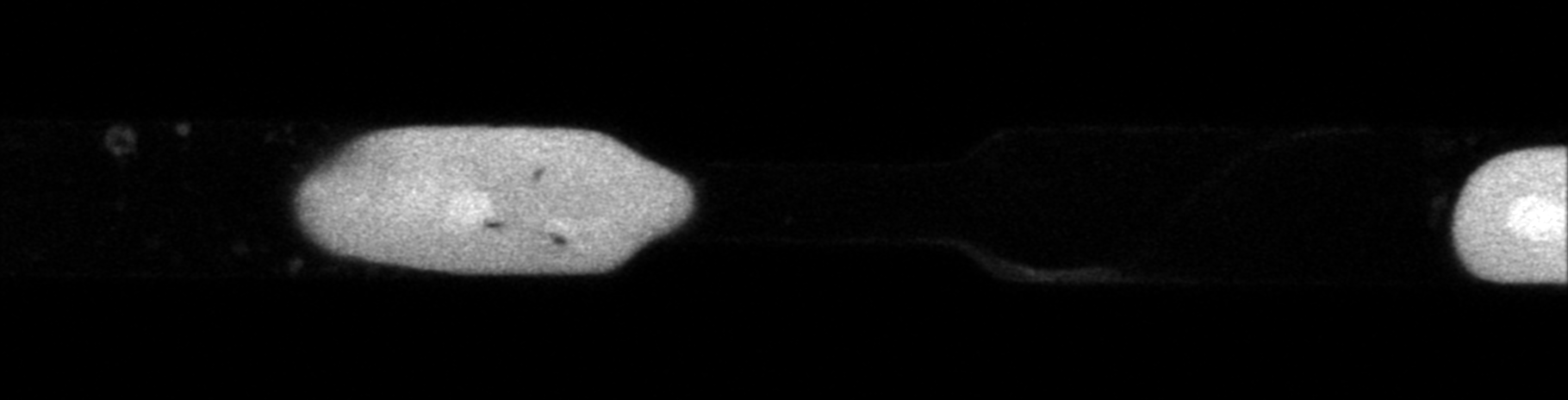

Supplement: Supplementary file 19 — Source data Fig. 5 [file 44318_2025_566_MOESM19_ESM.zip › Fig 5/Fig 5B/siCtrl_Before NE rupture.tif]

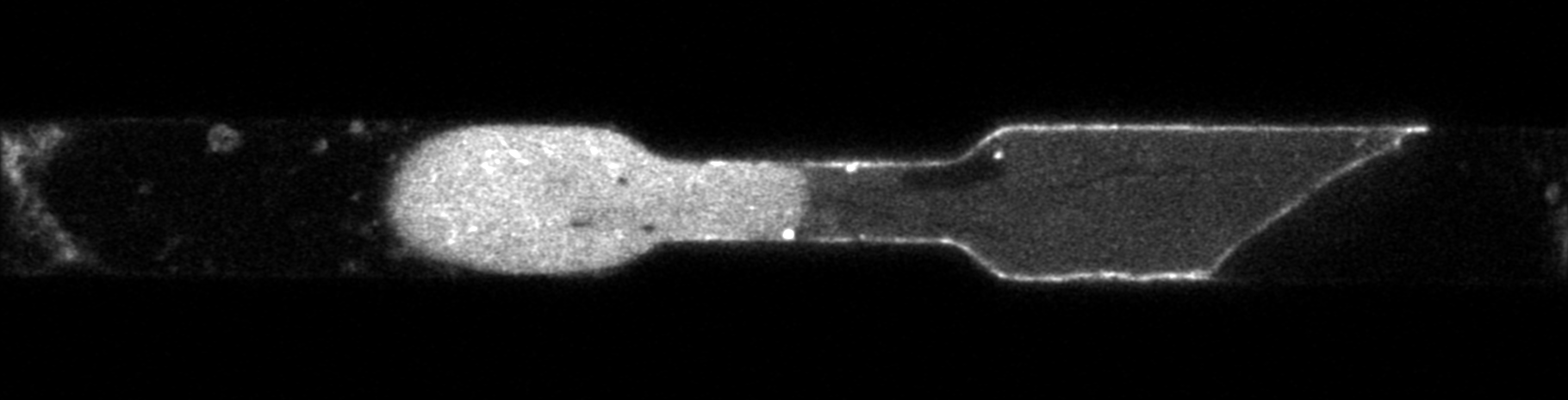

Supplement: Supplementary file 19 — Source data Fig. 5 [file 44318_2025_566_MOESM19_ESM.zip › Fig 5/Fig 5B/siCtrl_NE rupture.tif]

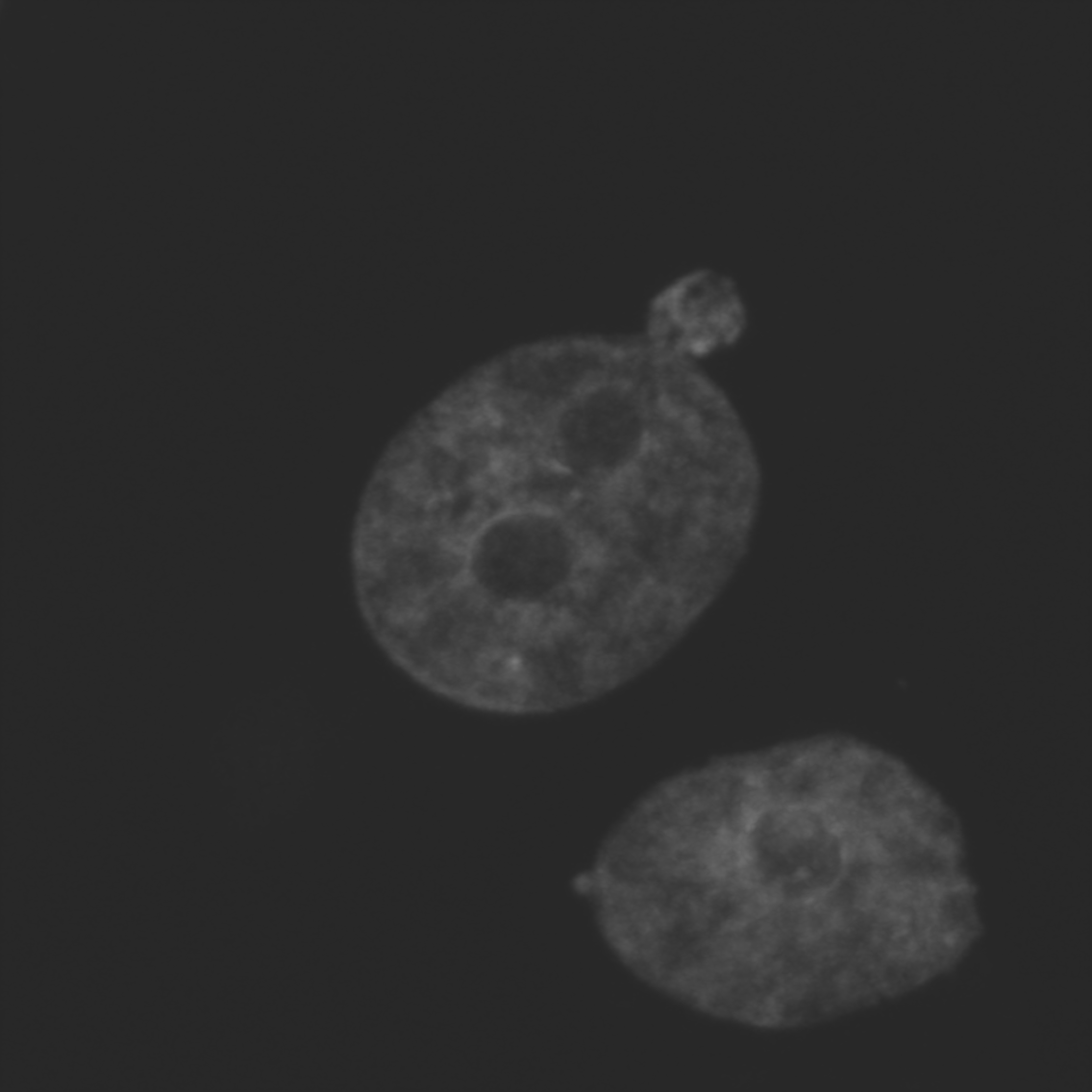

Supplement: Supplementary file 19 — Source data Fig. 5 [file 44318_2025_566_MOESM19_ESM.zip › Fig 5/Fig 5D/Fig 5D - siATR.tif]

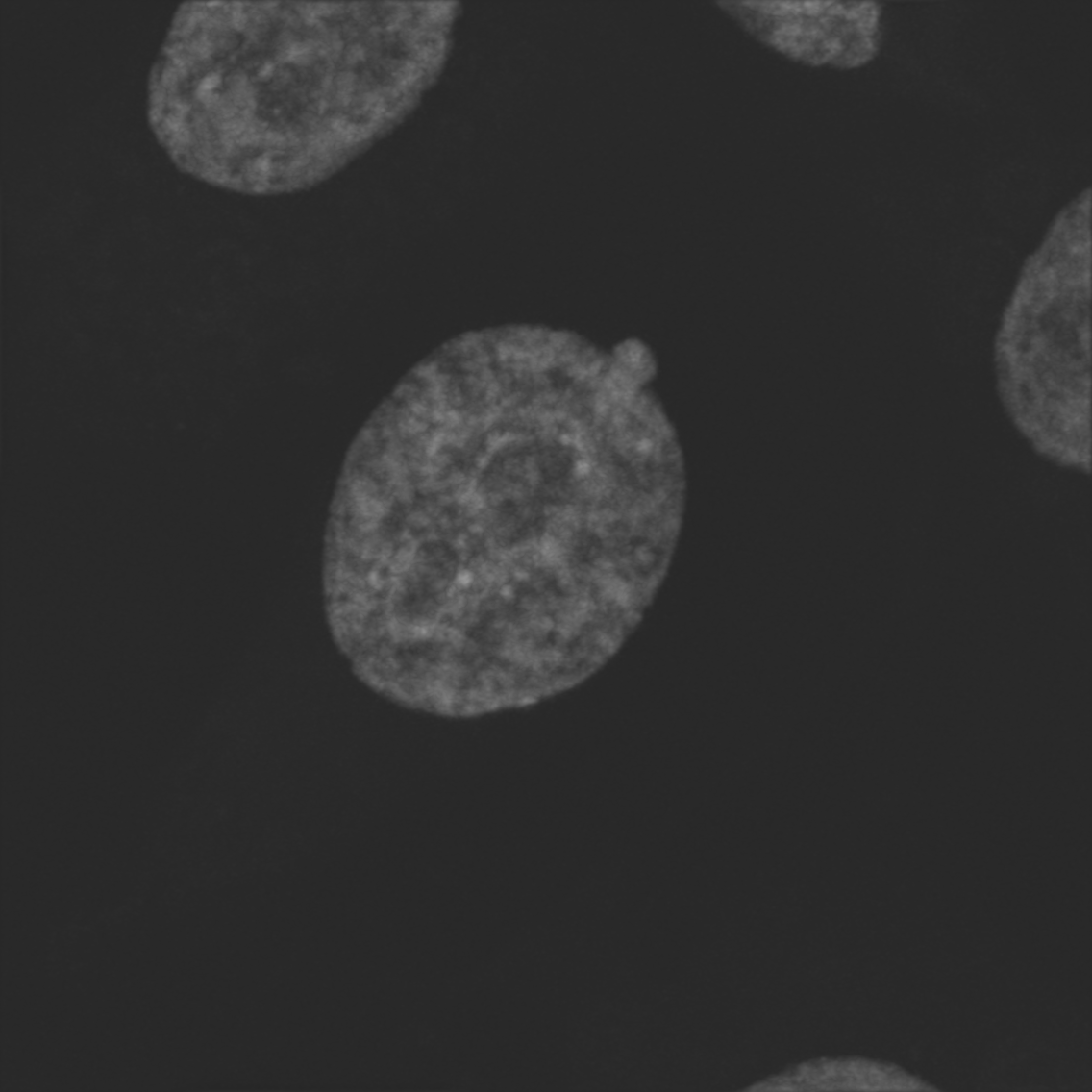

Supplement: Supplementary file 19 — Source data Fig. 5 [file 44318_2025_566_MOESM19_ESM.zip › Fig 5/Fig 5D/Fig 5D - siCtrl.tif]

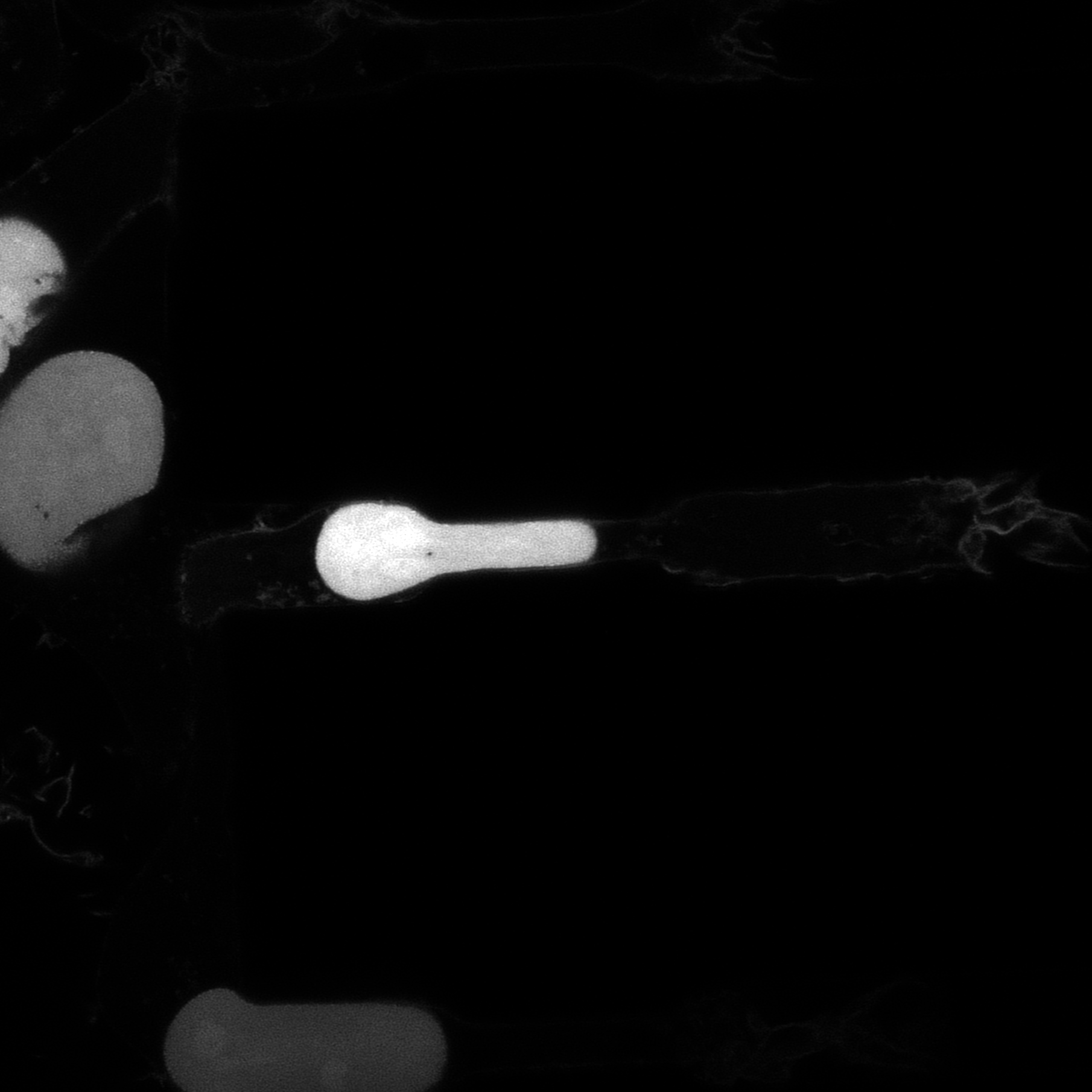

Supplement: Supplementary file 20 — Source data Fig. 6 [file 44318_2025_566_MOESM20_ESM.zip › Fig 6/Fig 6A/DMSO_Before NE rupture.tif]

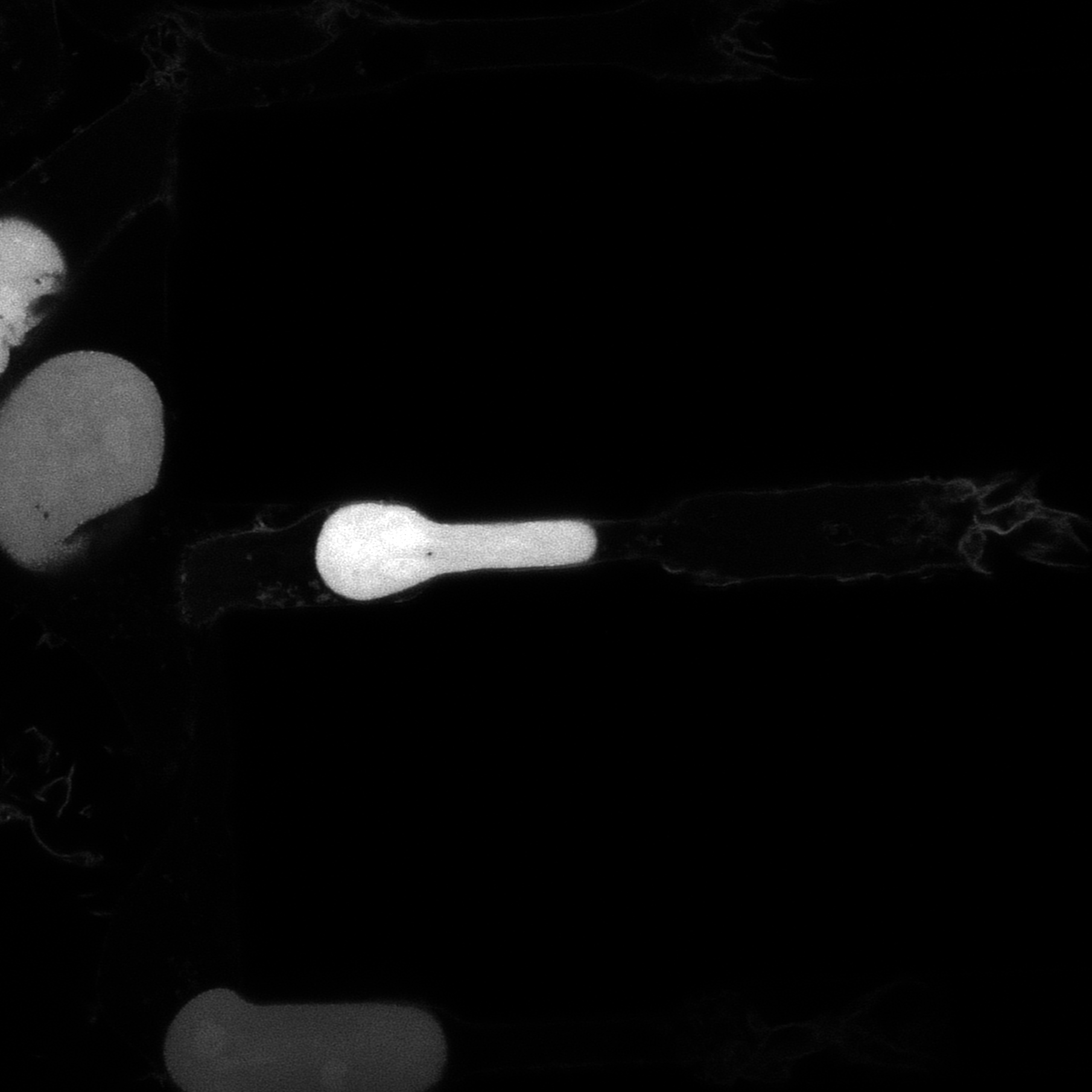

Supplement: Supplementary file 20 — Source data Fig. 6 [file 44318_2025_566_MOESM20_ESM.zip › Fig 6/Fig 6A/DMSO_NE rupture.tif]

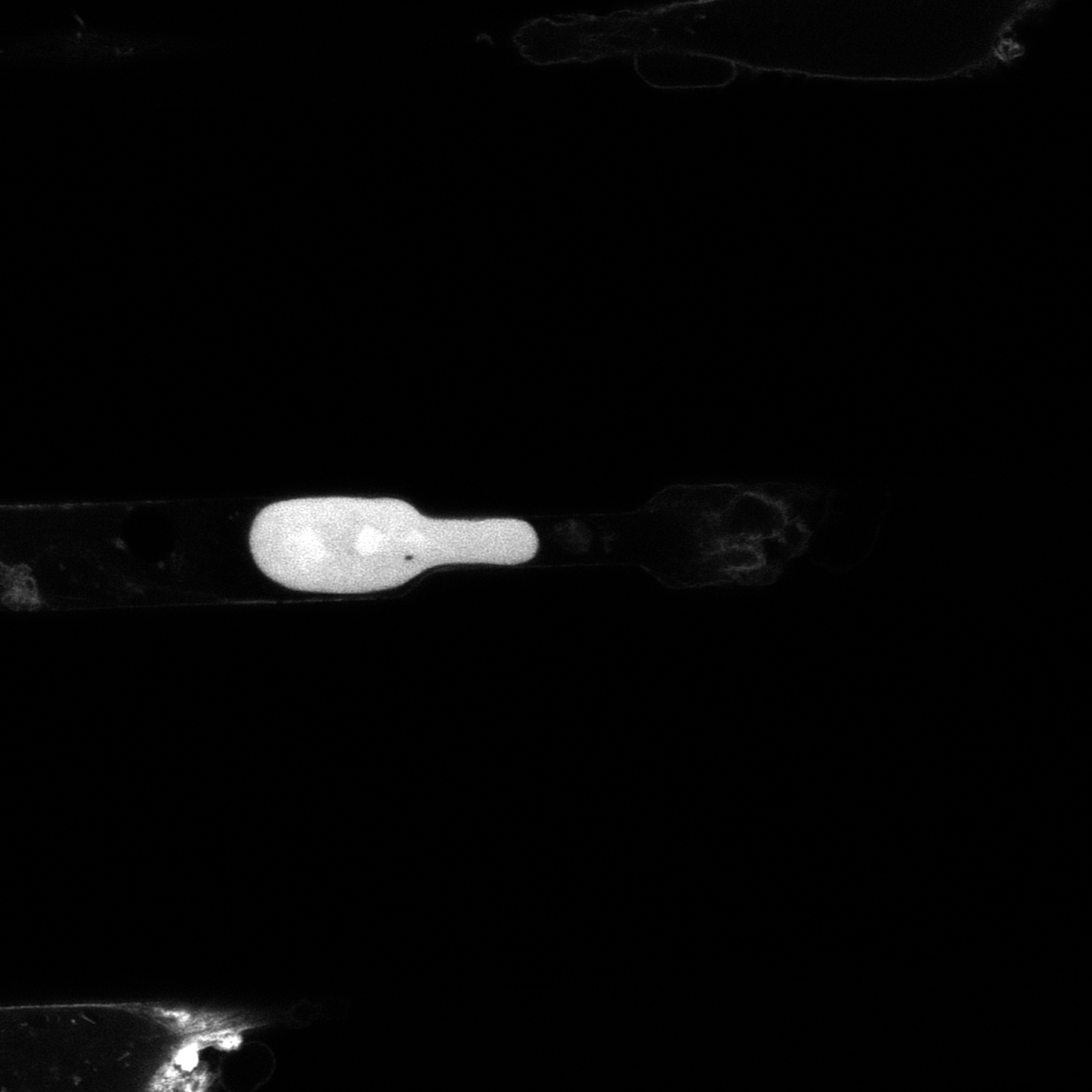

Supplement: Supplementary file 20 — Source data Fig. 6 [file 44318_2025_566_MOESM20_ESM.zip › Fig 6/Fig 6A/ETP46464_Before NE rupture.tif]

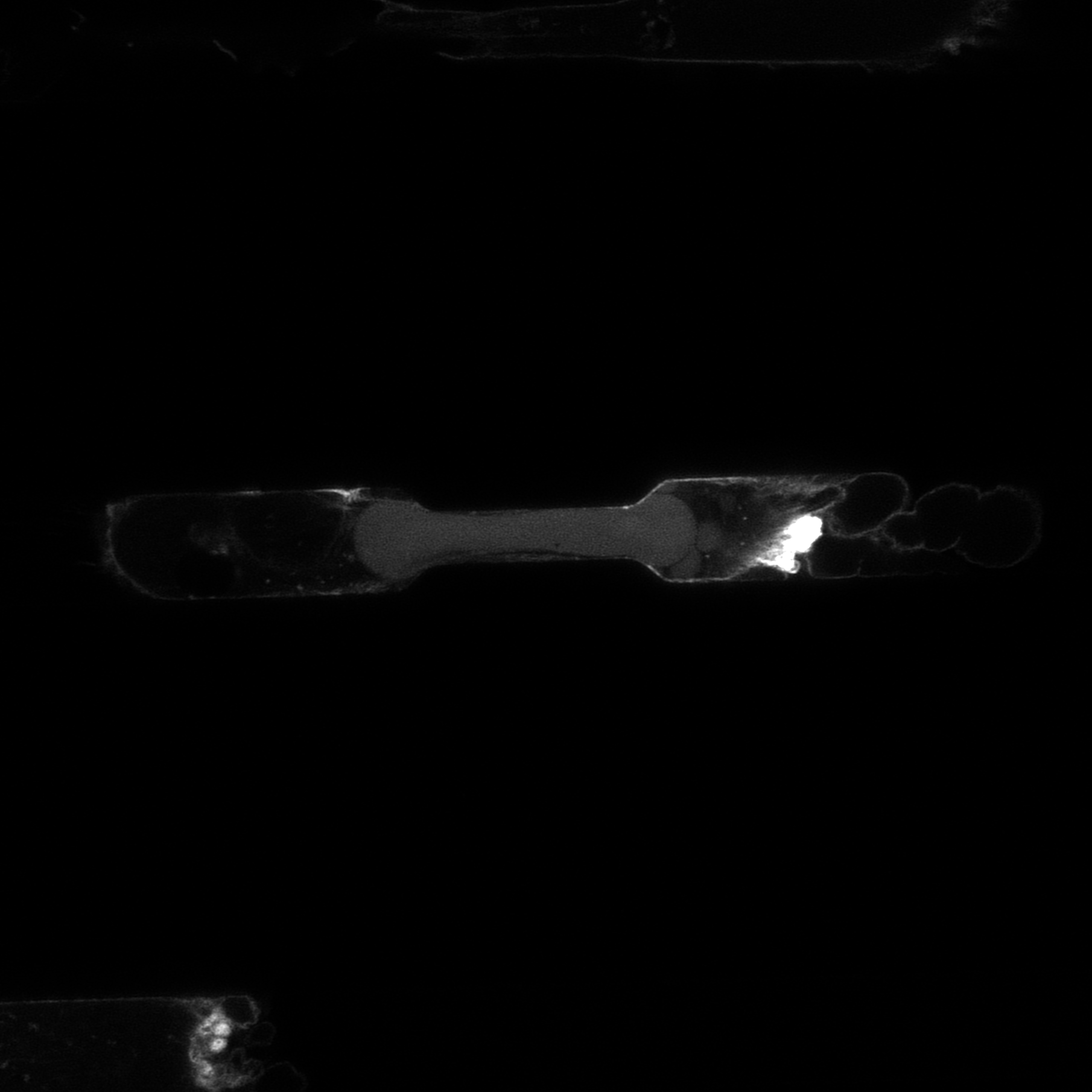

Supplement: Supplementary file 20 — Source data Fig. 6 [file 44318_2025_566_MOESM20_ESM.zip › Fig 6/Fig 6A/ETP46464_NE rupture.tif]

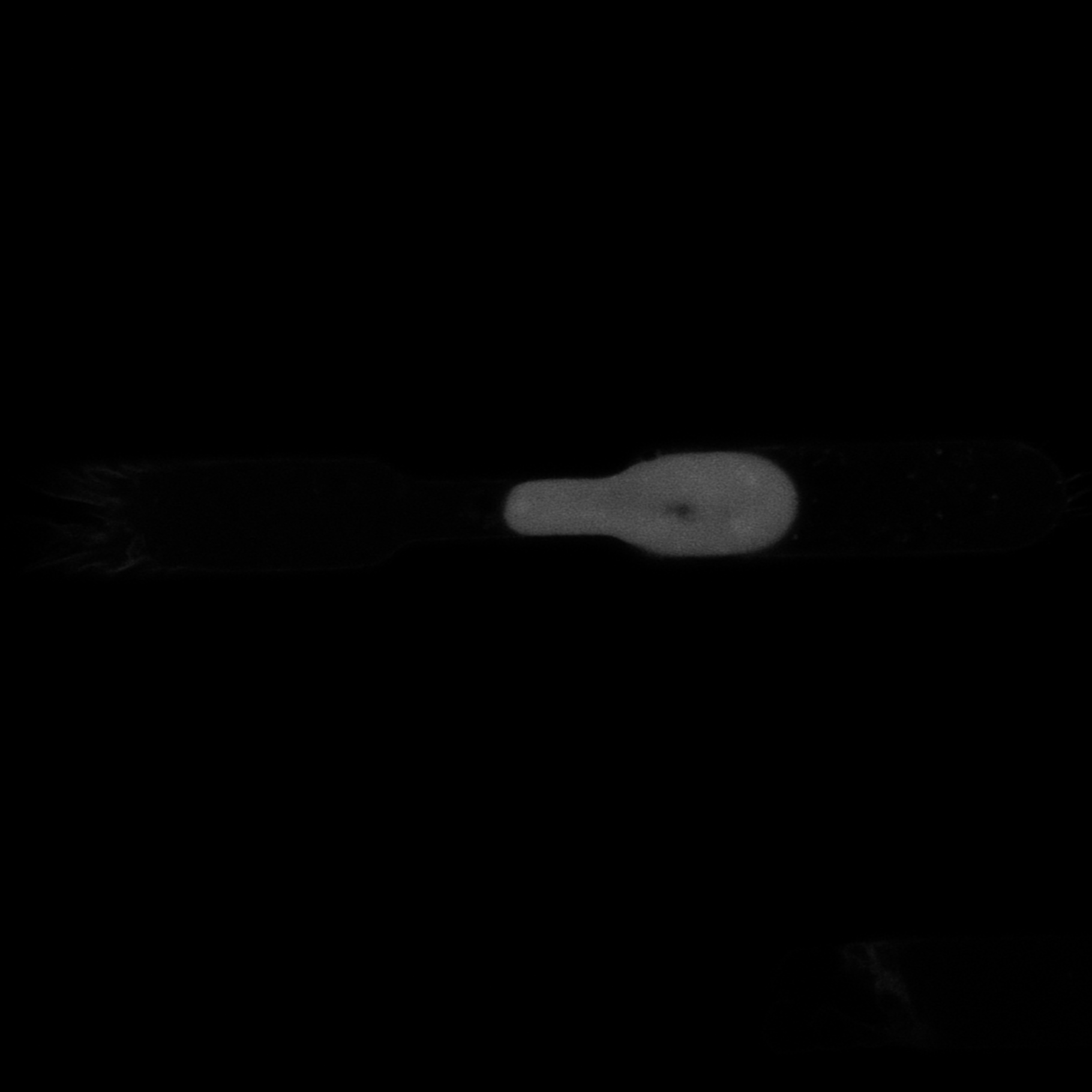

Supplement: Supplementary file 20 — Source data Fig. 6 [file 44318_2025_566_MOESM20_ESM.zip › Fig 6/Fig 6A/KU60019_Before NE rupture.tif]

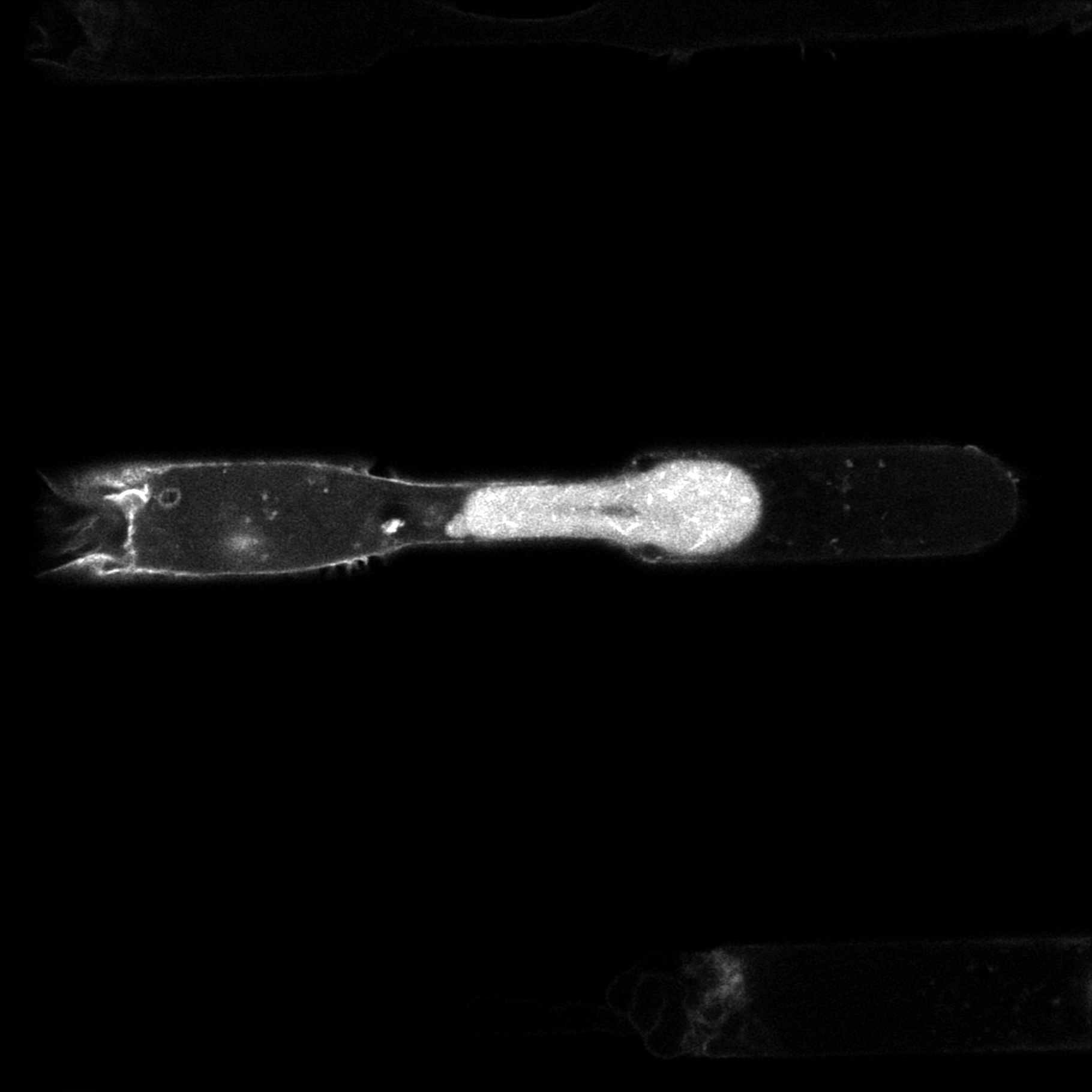

Supplement: Supplementary file 20 — Source data Fig. 6 [file 44318_2025_566_MOESM20_ESM.zip › Fig 6/Fig 6A/KU60019_NE rupture.tif]

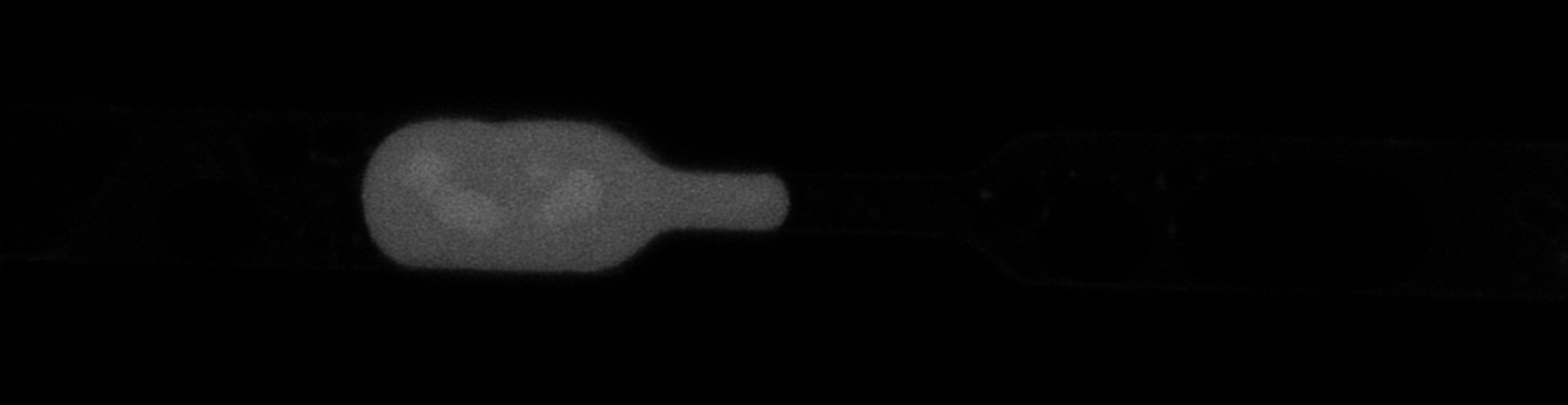

Supplement: Supplementary file 20 — Source data Fig. 6 [file 44318_2025_566_MOESM20_ESM.zip › Fig 6/Fig 6A/VE-821_Before NE rupture.tif]

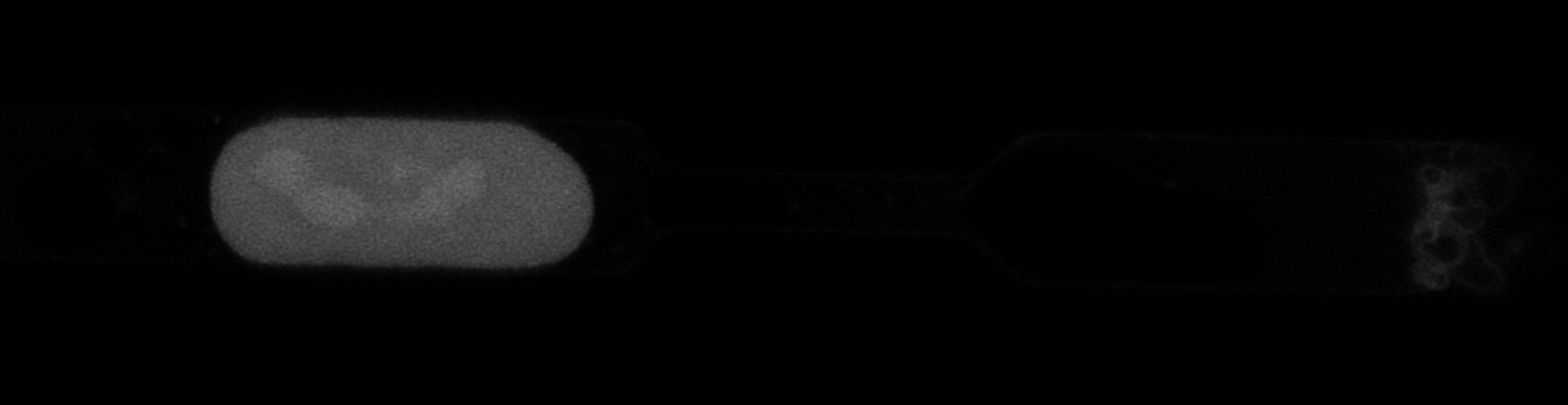

Supplement: Supplementary file 20 — Source data Fig. 6 [file 44318_2025_566_MOESM20_ESM.zip › Fig 6/Fig 6A/VE-821_NE rupture.tif]

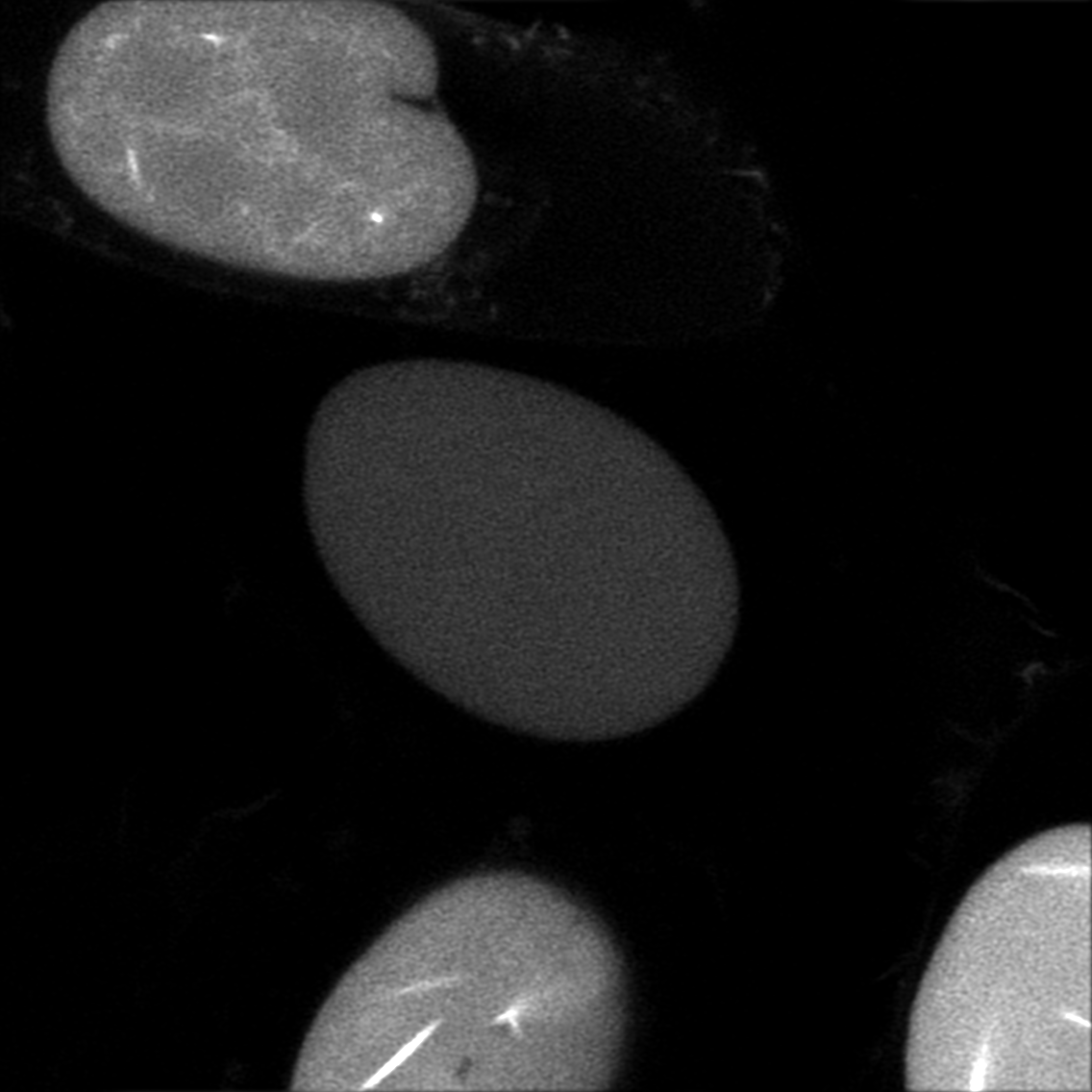

Supplement: Supplementary file 21 — Source data Fig. 7 [file 44318_2025_566_MOESM21_ESM.zip › Fig 7/Fig 7C/Fig 7C - Diaph3-S1072A.tif]

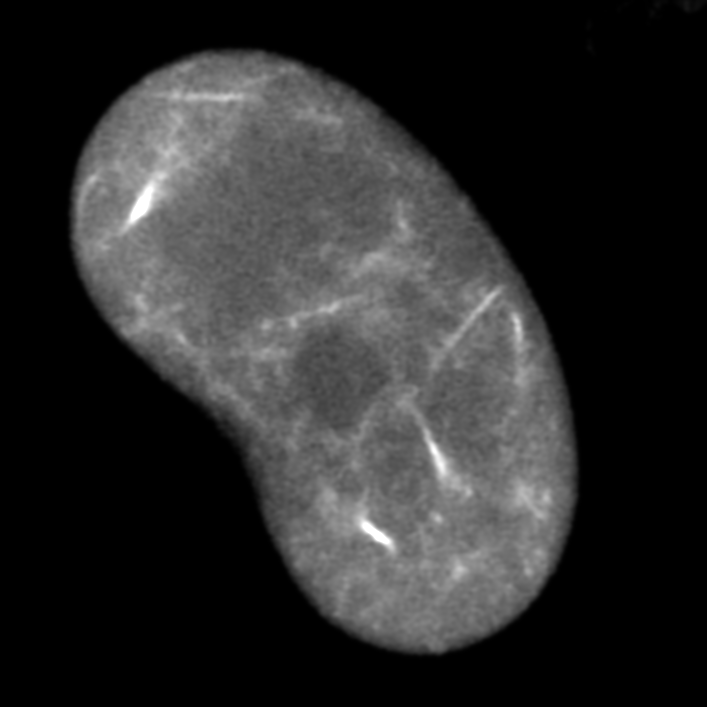

Supplement: Supplementary file 21 — Source data Fig. 7 [file 44318_2025_566_MOESM21_ESM.zip › Fig 7/Fig 7C/Fig 7C - Diaph3-S1072D.tif]

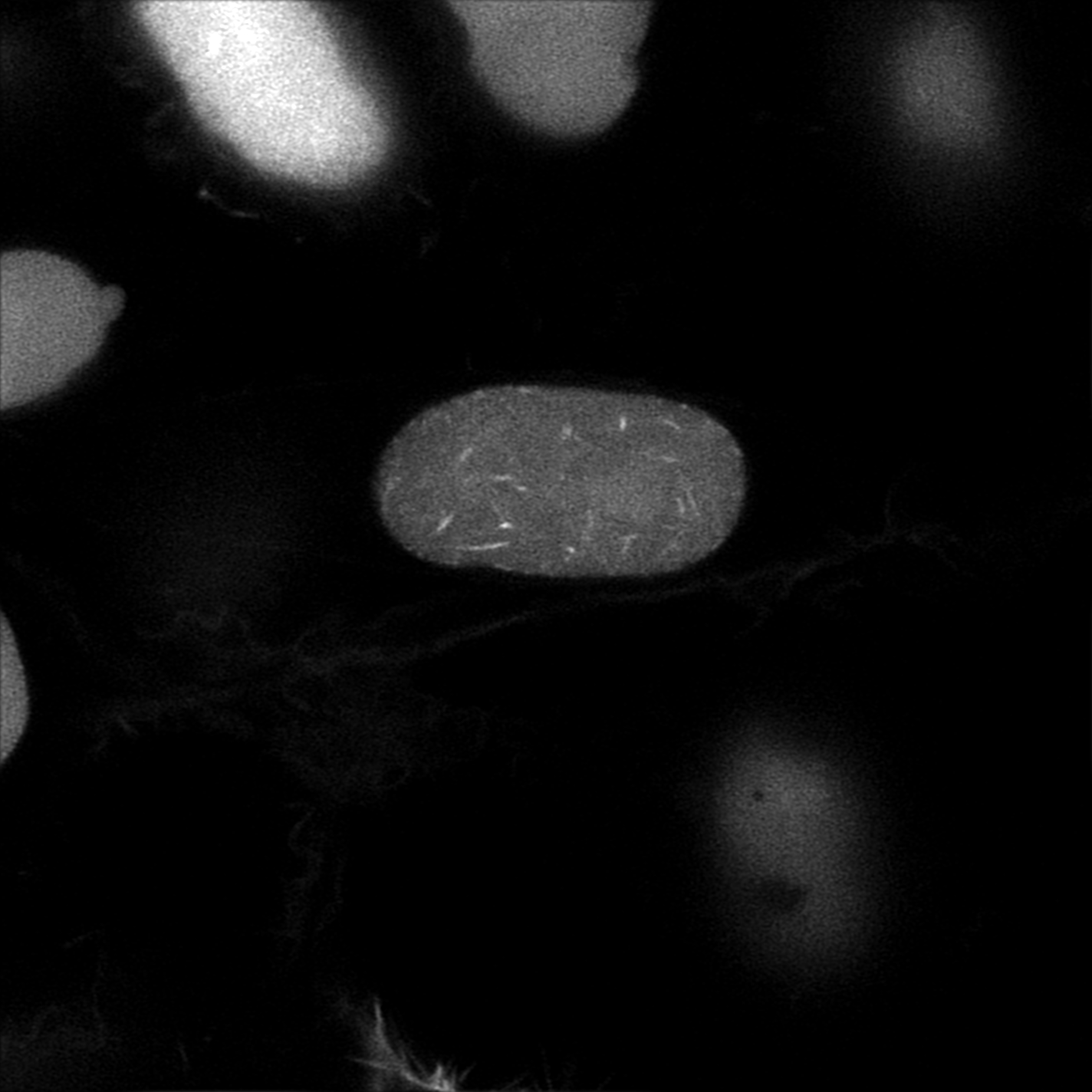

Supplement: Supplementary file 21 — Source data Fig. 7 [file 44318_2025_566_MOESM21_ESM.zip › Fig 7/Fig 7C/Fig 7C - Diaph3-wt.tif]

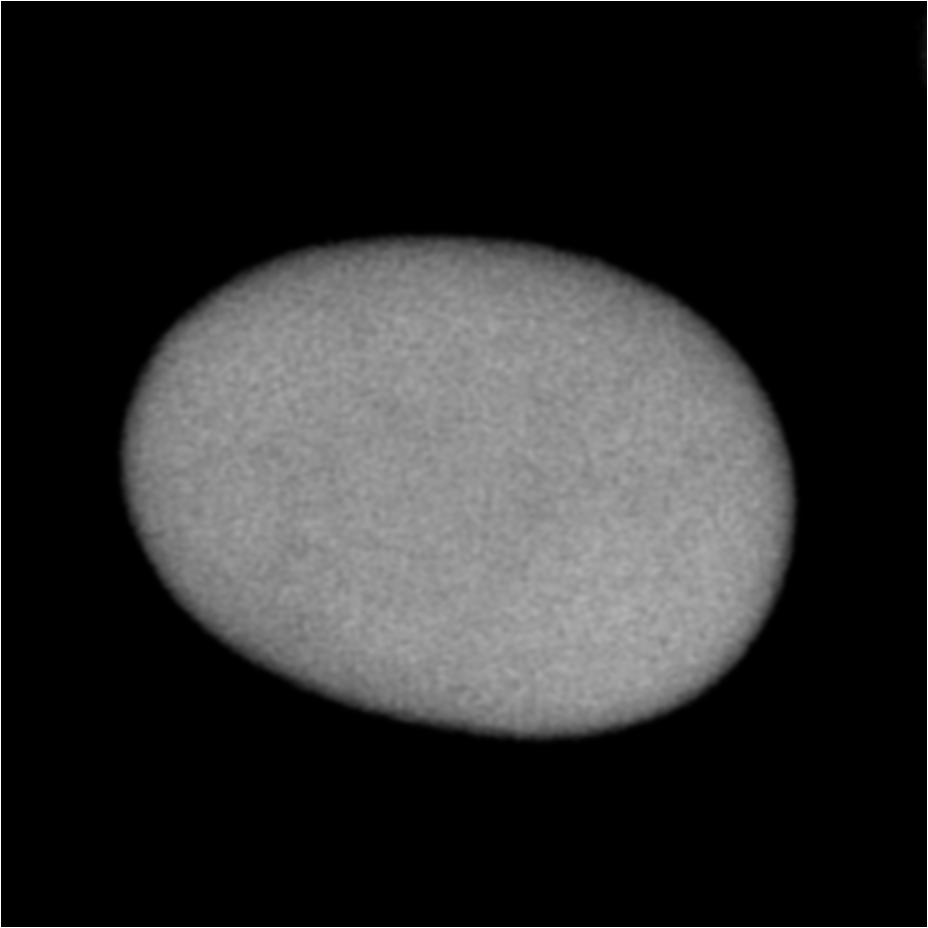

Supplement: Supplementary file 21 — Source data Fig. 7 [file 44318_2025_566_MOESM21_ESM.zip › Fig 7/Fig 7C/Fig 7C - NLS-BFP.tif]

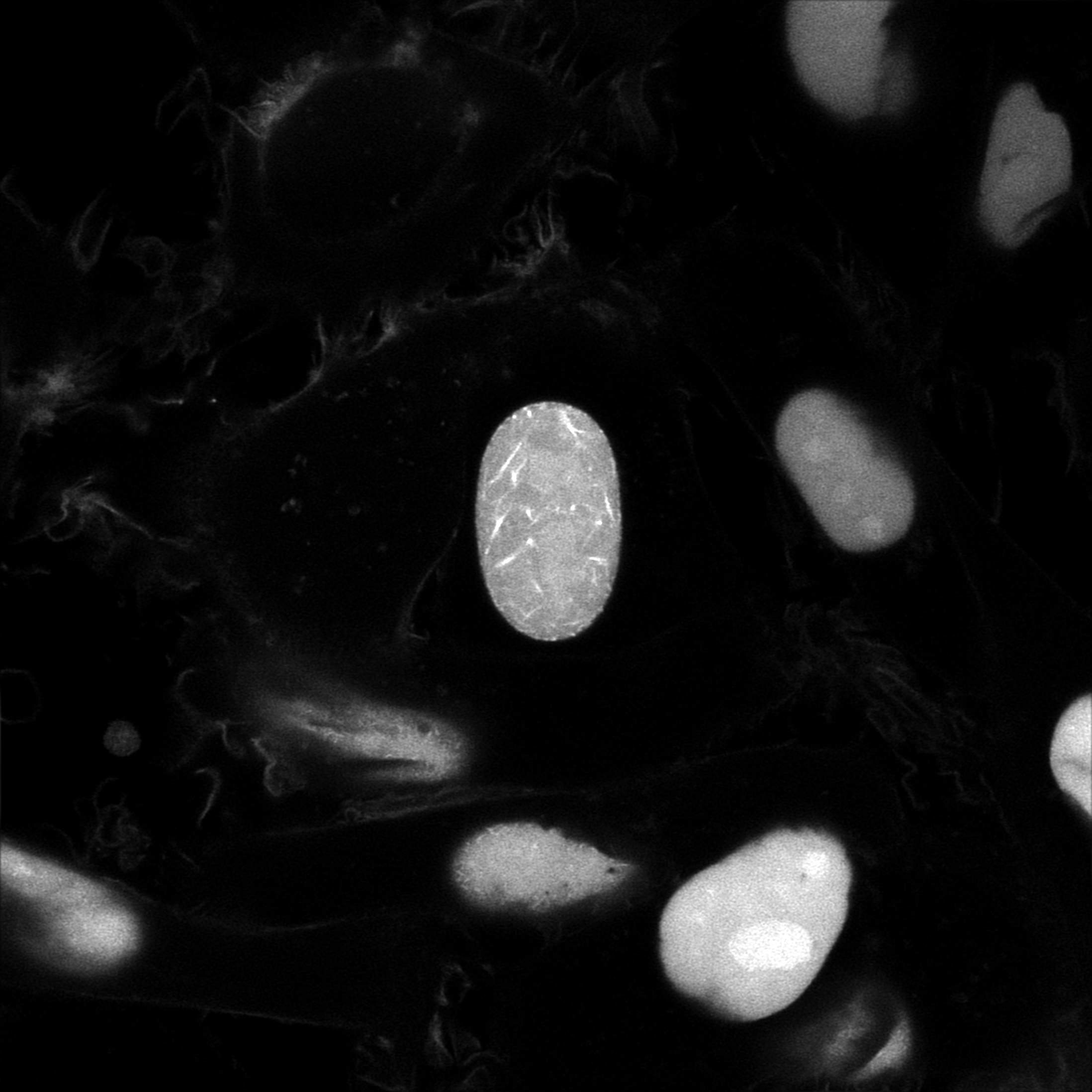

Supplement: Supplementary file 21 — Source data Fig. 7 [file 44318_2025_566_MOESM21_ESM.zip › Fig 7/Fig 7E/Fig 7F - S0172D_DMSO.tif]

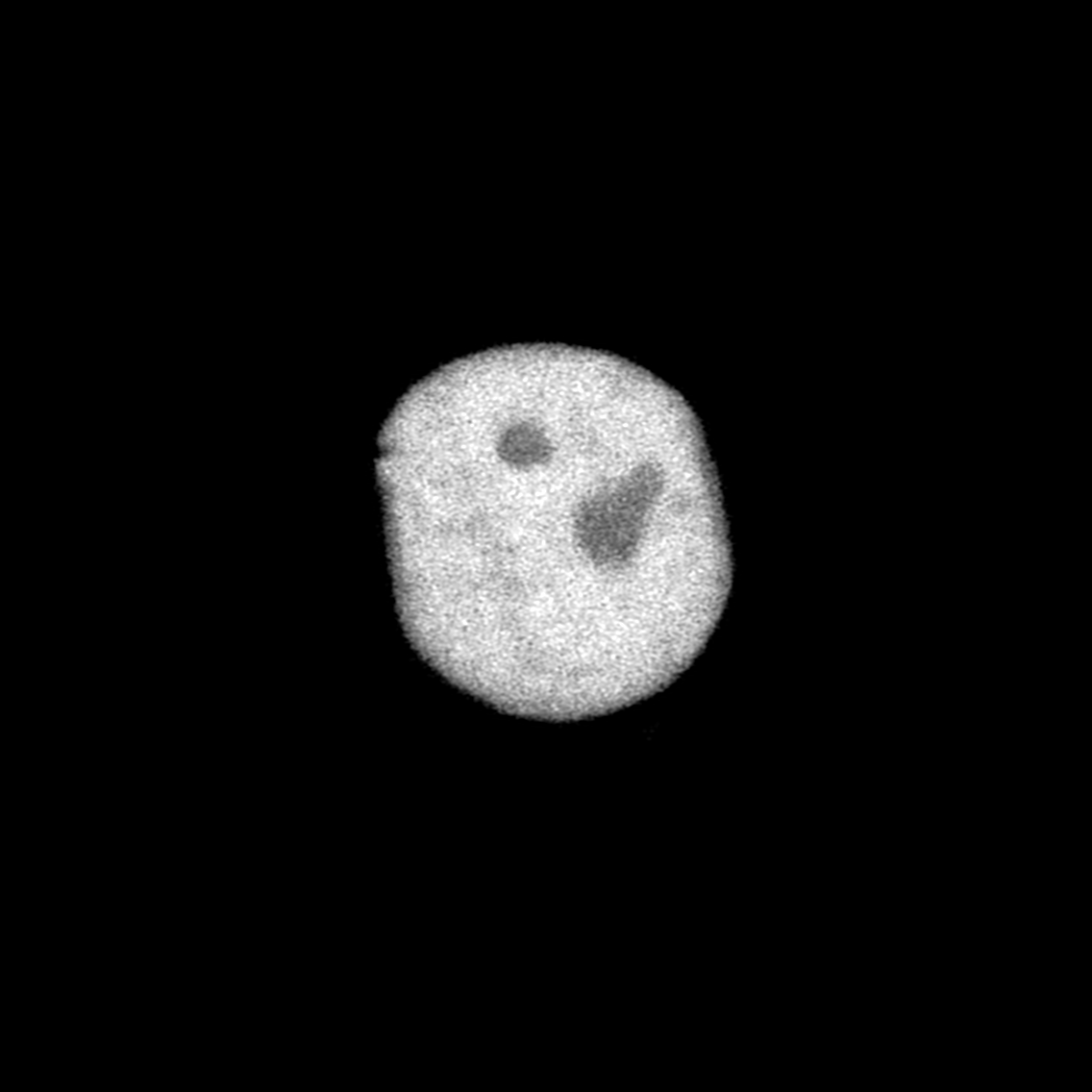

Supplement: Supplementary file 21 — Source data Fig. 7 [file 44318_2025_566_MOESM21_ESM.zip › Fig 7/Fig 7E/Fig 7F - S1072D_ETP46464.tif]

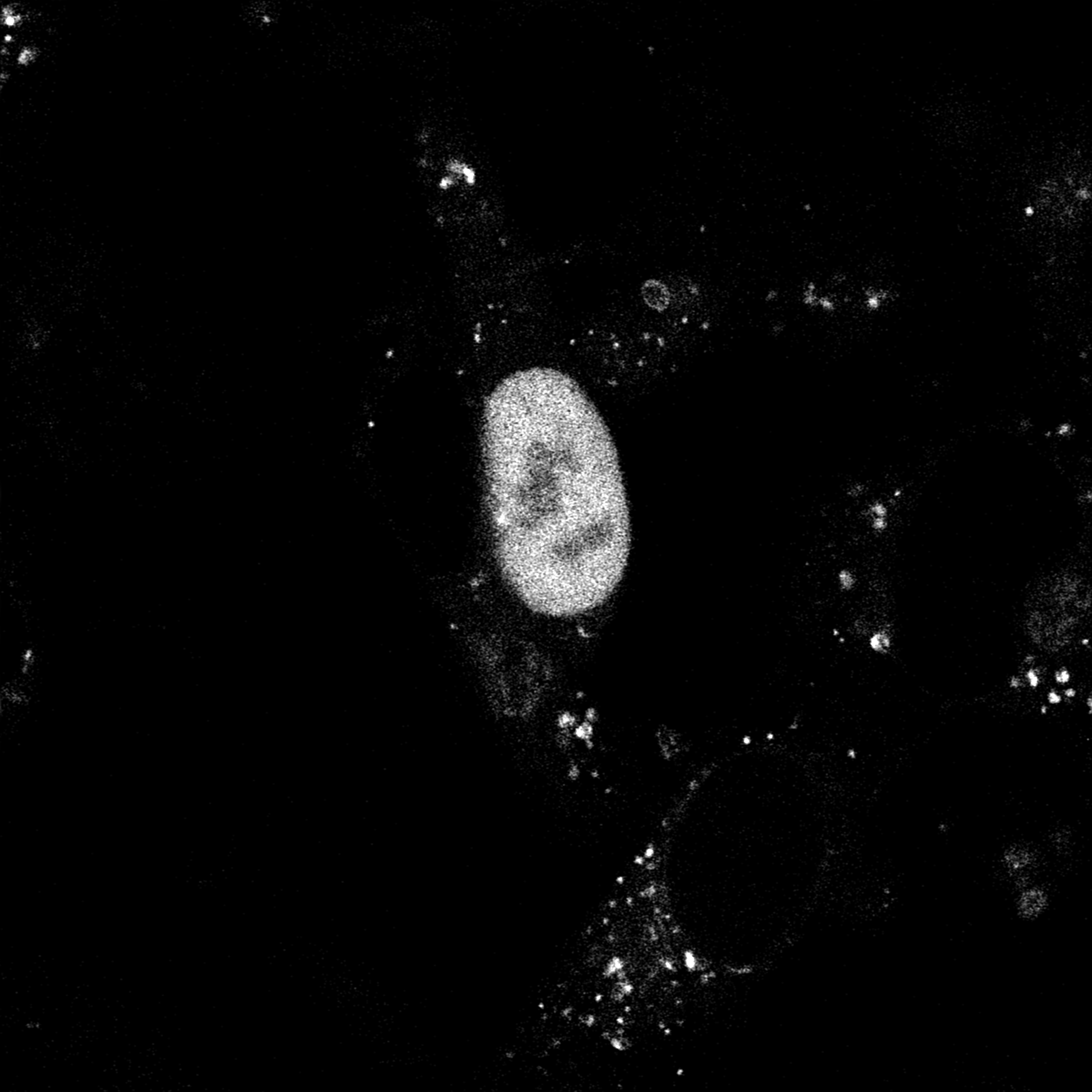

Supplement: Supplementary file 21 — Source data Fig. 7 [file 44318_2025_566_MOESM21_ESM.zip › Fig 7/Fig 7E/Fig 7F - WT_ET46464.tif]

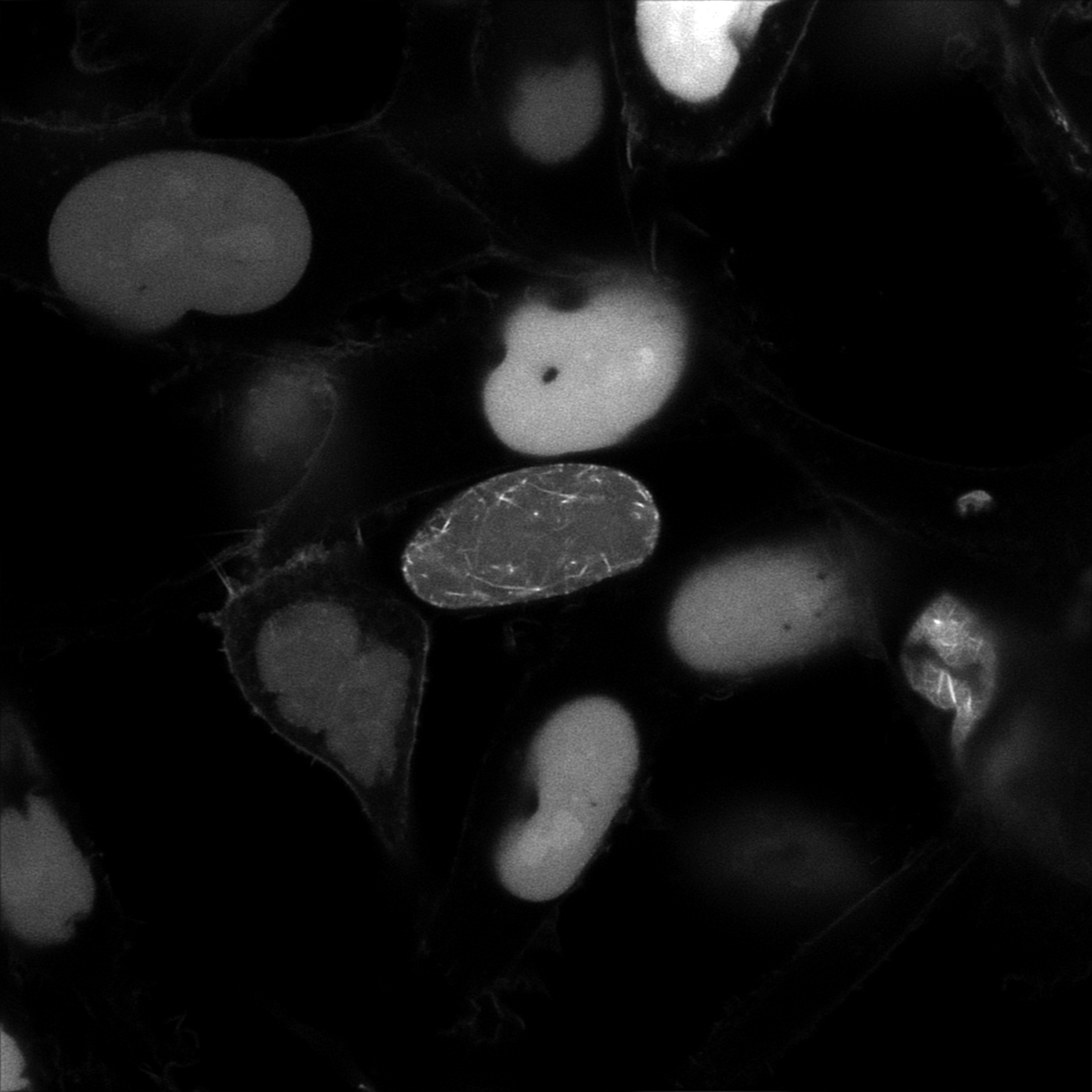

Supplement: Supplementary file 21 — Source data Fig. 7 [file 44318_2025_566_MOESM21_ESM.zip › Fig 7/Fig 7E/Fig7F - WT_DMSO.tif]

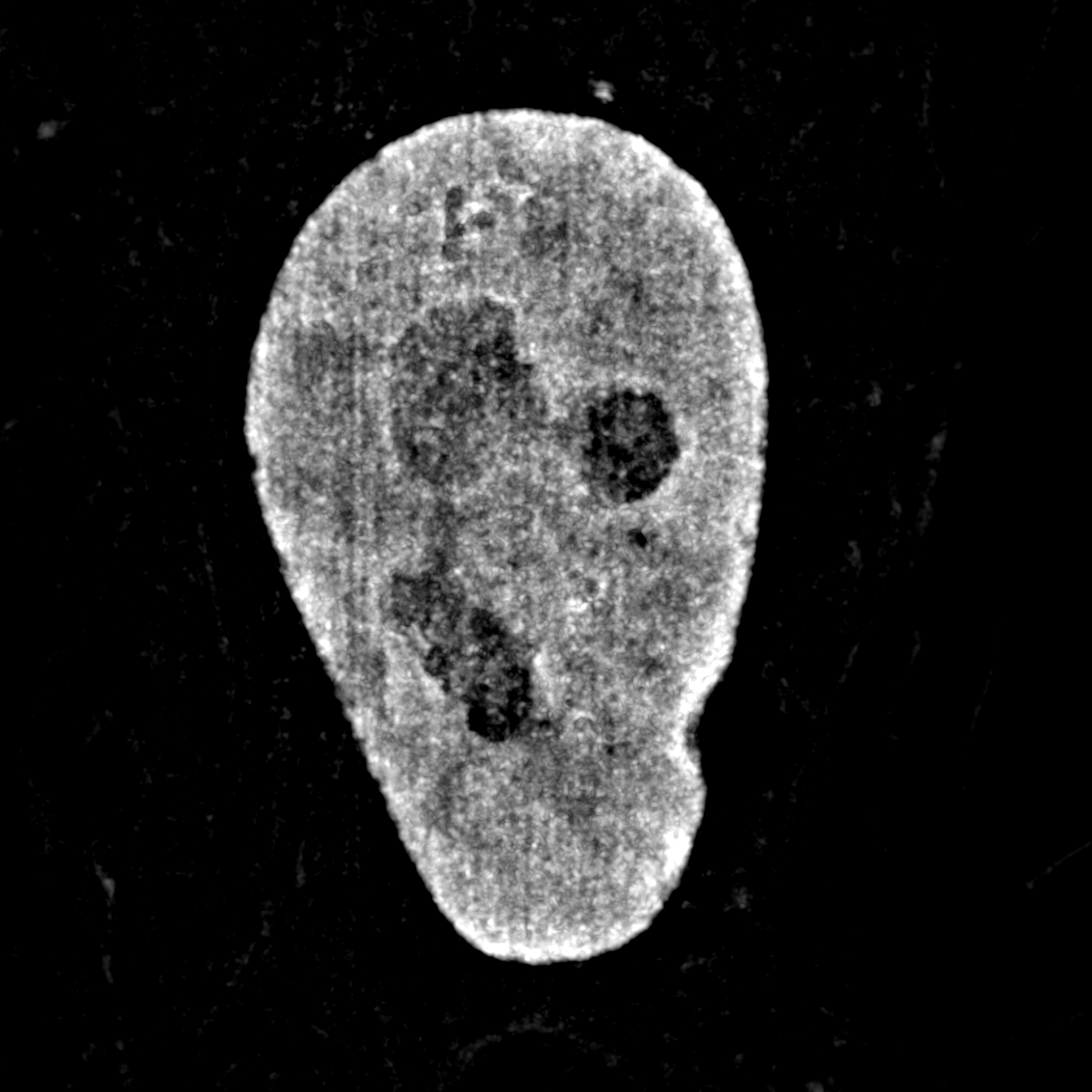

Supplement: Supplementary file 22 — Source data Fig. 8 [file 44318_2025_566_MOESM22_ESM.zip › Fig 8/Fig 8B/NLS_mScarlet.tif]

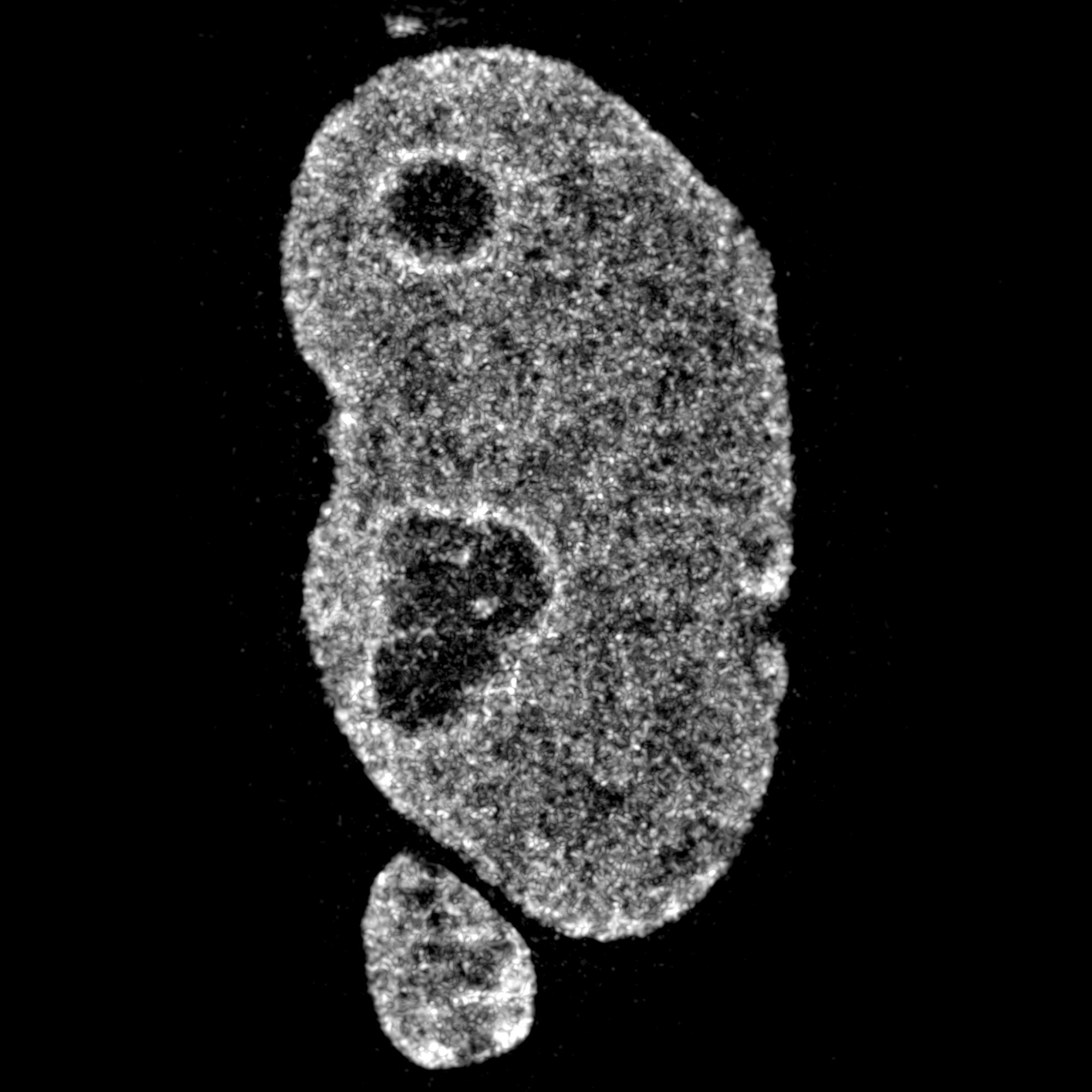

Supplement: Supplementary file 22 — Source data Fig. 8 [file 44318_2025_566_MOESM22_ESM.zip › Fig 8/Fig 8B/NLS_mScarlet_Actin_R62D.tif]

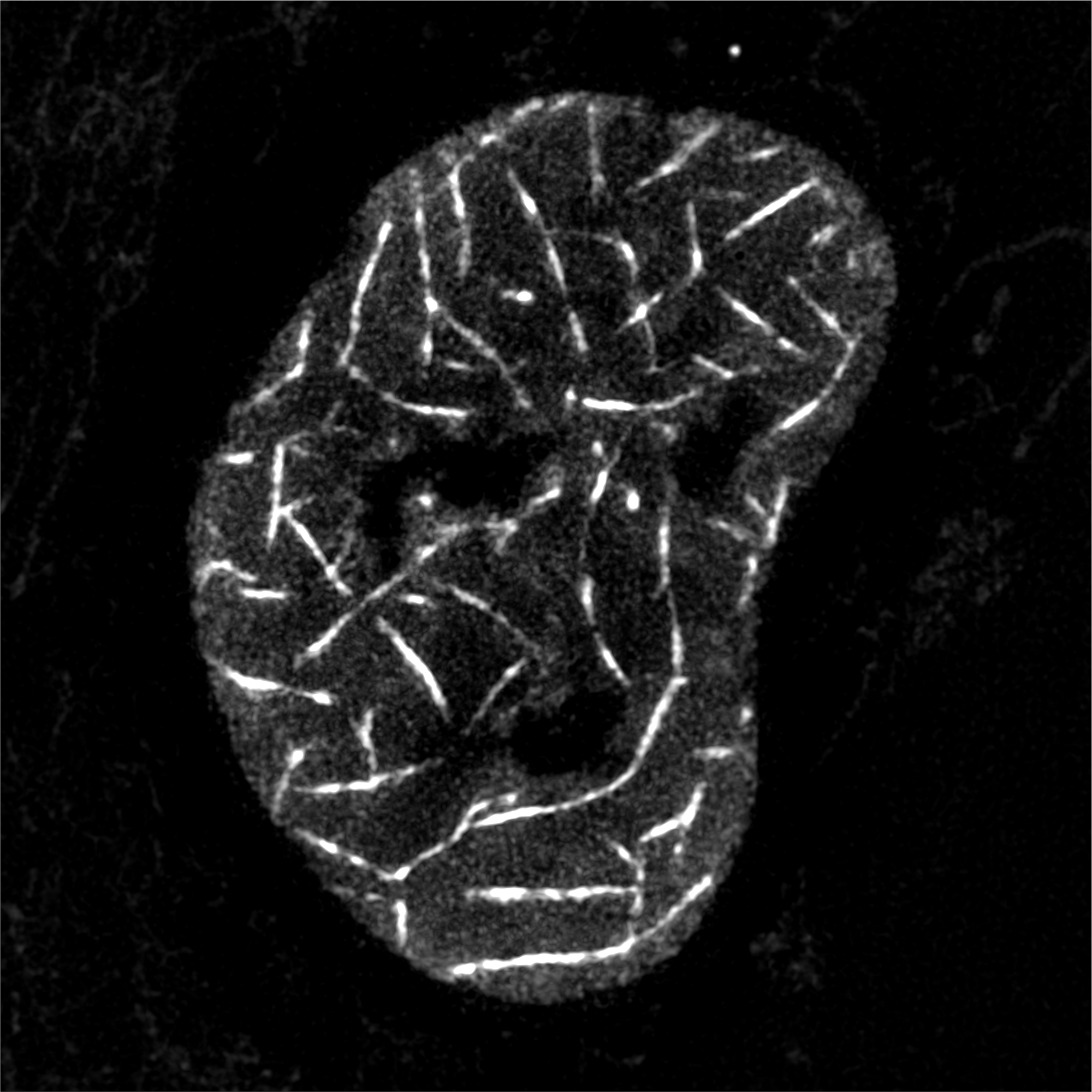

Supplement: Supplementary file 22 — Source data Fig. 8 [file 44318_2025_566_MOESM22_ESM.zip › Fig 8/Fig 8B/NLS_mScarlet_Actin_S14C.tif]

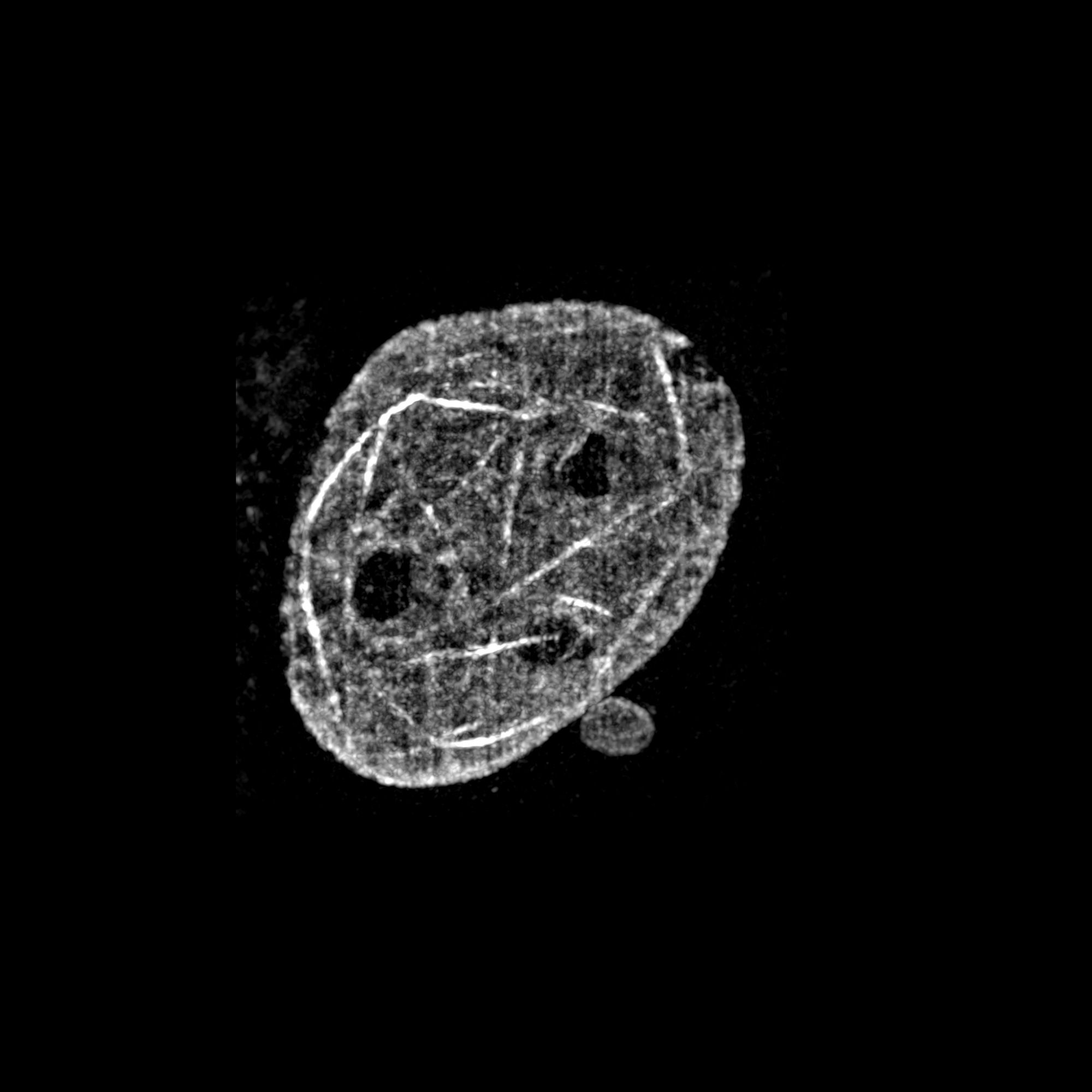

Supplement: Supplementary file 22 — Source data Fig. 8 [file 44318_2025_566_MOESM22_ESM.zip › Fig 8/Fig 8B/NLS_mScarlet_Actin_WT.tif]

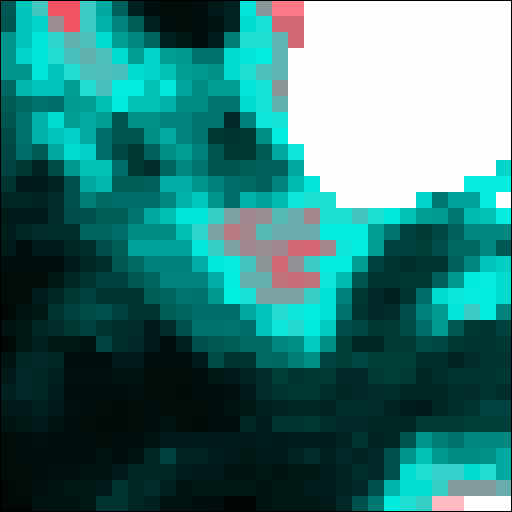

Supplement: Supplementary file 22 — Source data Fig. 8 [file 44318_2025_566_MOESM22_ESM.zip › Fig 8/Fig 8C/NLS_mScarlet_Actin_S14C_Force_MAP.png]

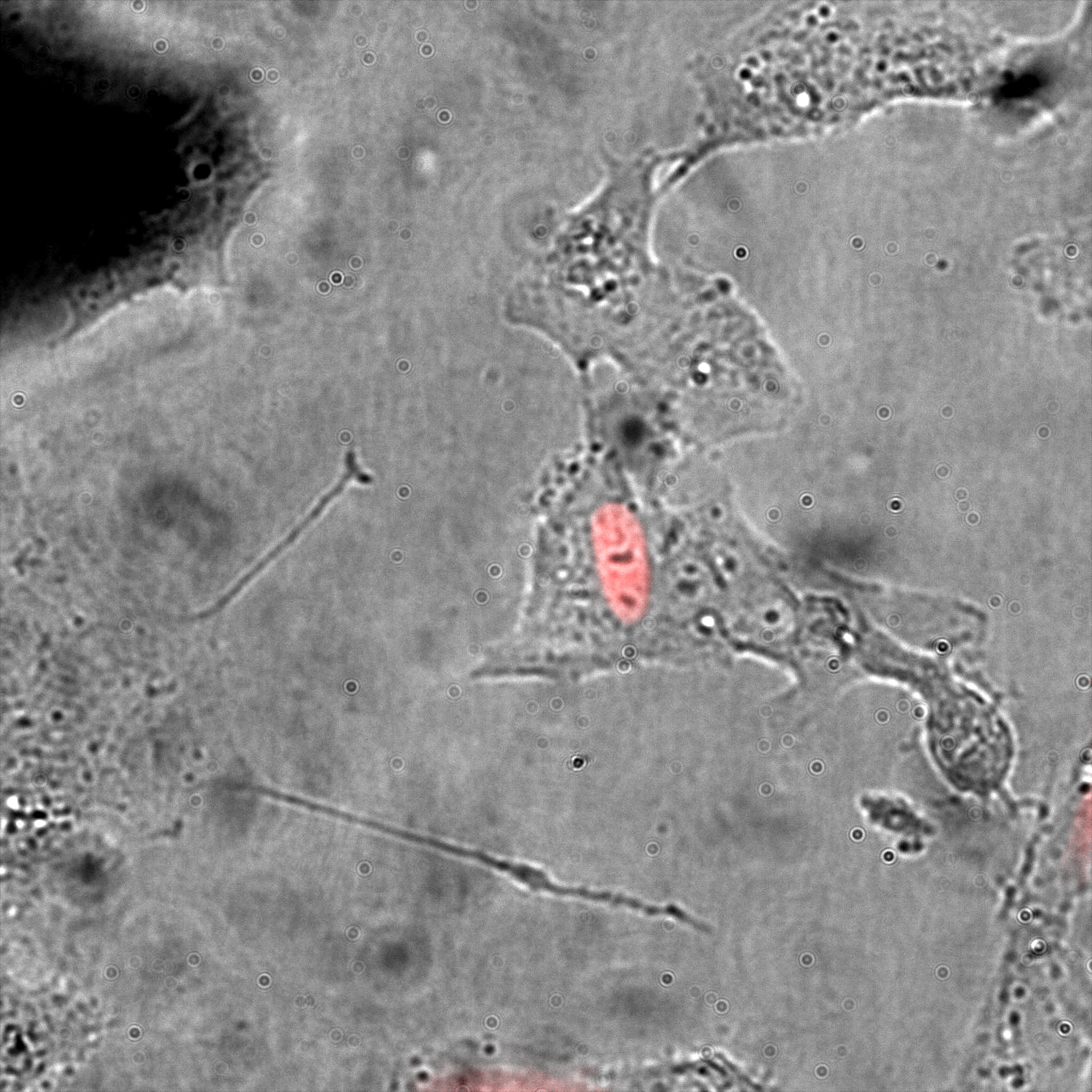

Supplement: Supplementary file 22 — Source data Fig. 8 [file 44318_2025_566_MOESM22_ESM.zip › Fig 8/Fig 8C/NLS_MScarlet_ActinR62D_AFM_FOR_FORCE_MAP.png]

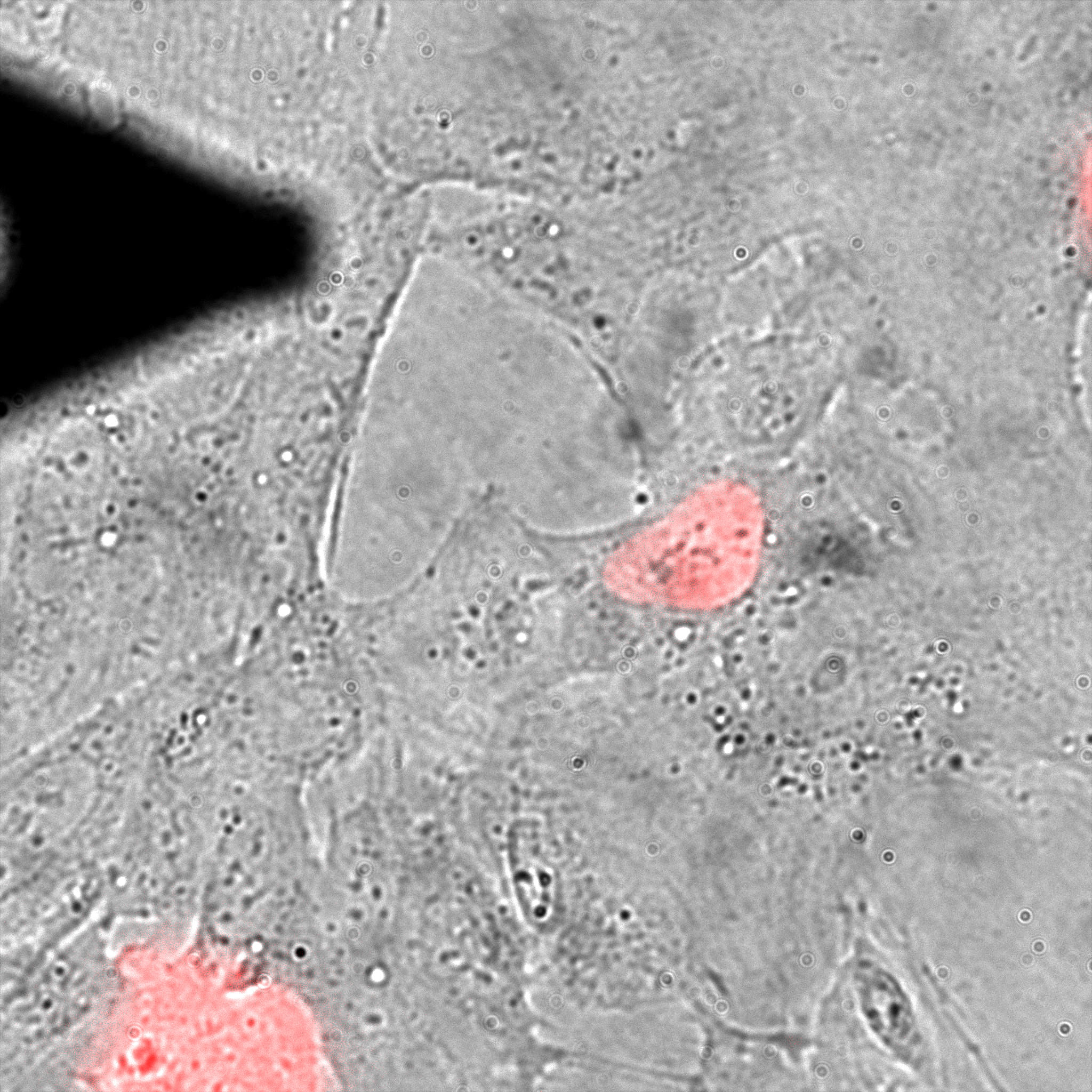

Supplement: Supplementary file 22 — Source data Fig. 8 [file 44318_2025_566_MOESM22_ESM.zip › Fig 8/Fig 8C/NLS_MScarlet_ActinS14C_AFM_FOR_FORCE_MAP.png]

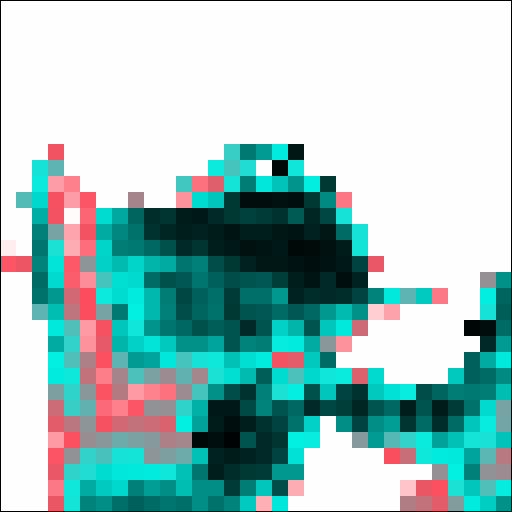

Supplement: Supplementary file 22 — Source data Fig. 8 [file 44318_2025_566_MOESM22_ESM.zip › Fig 8/Fig 8C/NLS-mScarlet_Actin_R62D_Force_MAP.png]

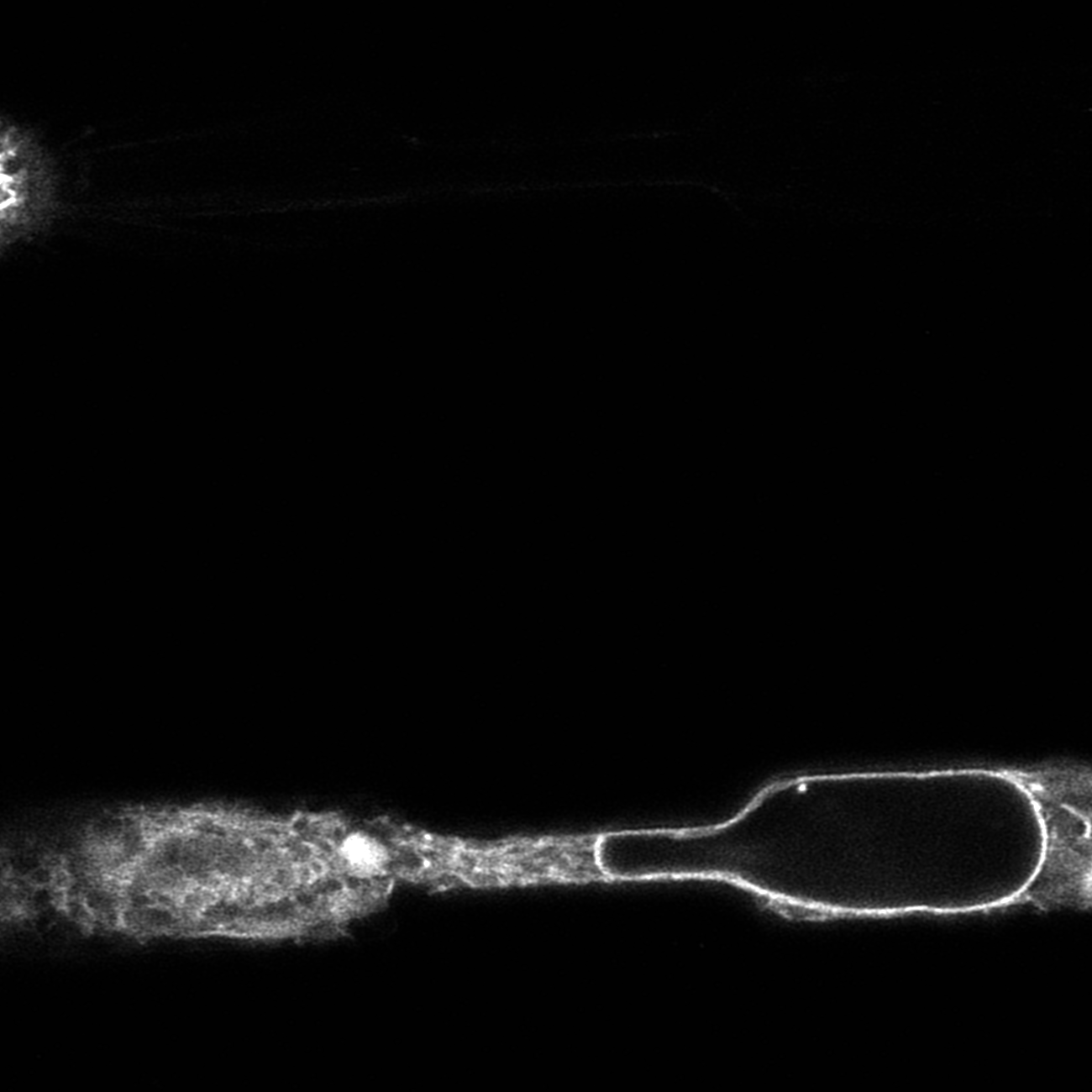

Supplement: Supplementary file 24 — Figure EV1 Source Data [file 44318_2025_566_MOESM24_ESM.zip › Fig EV1/Fig EV1A/Dn.KASH/DN.KASH_Before NE rupture_DN.KASH.tif]

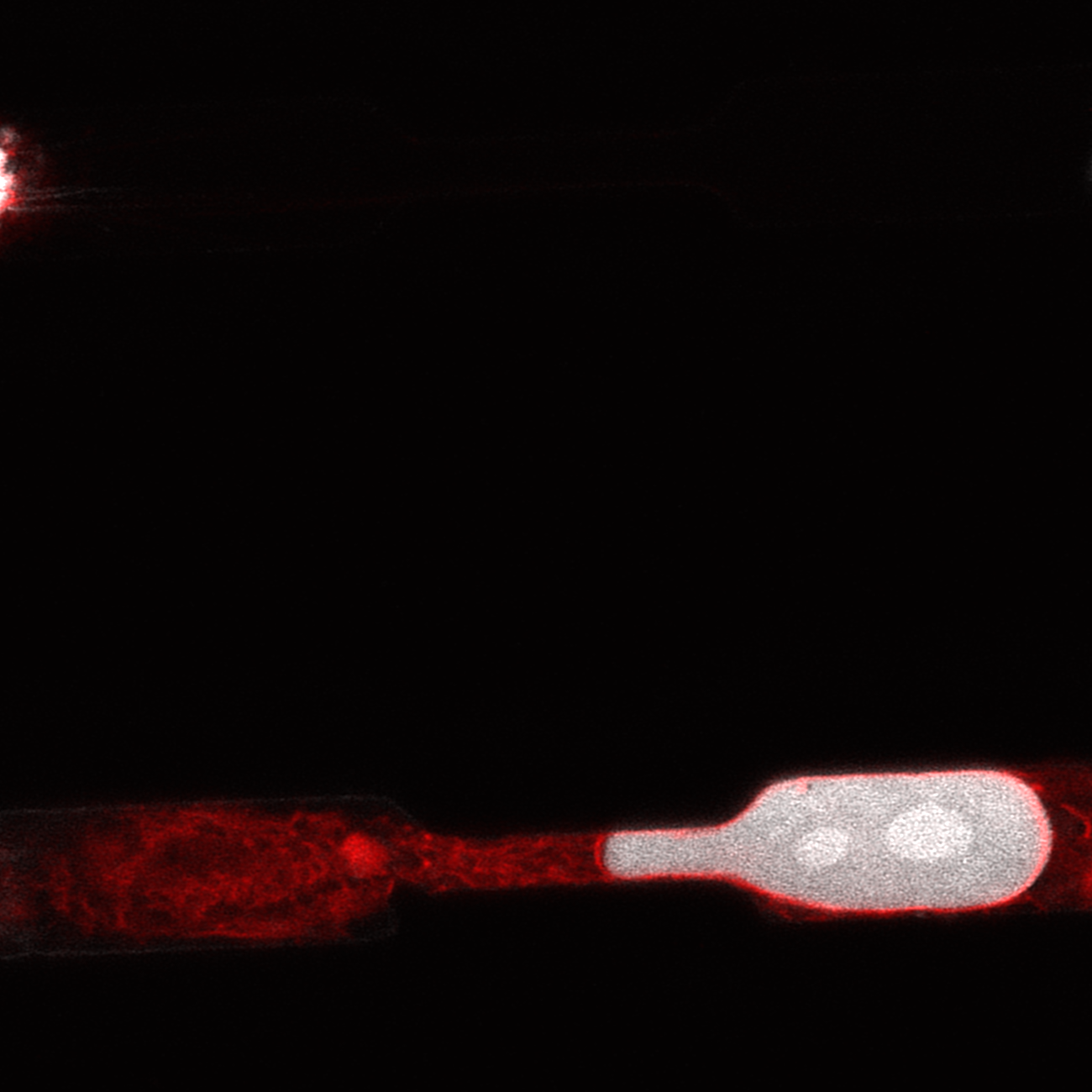

Supplement: Supplementary file 24 — Figure EV1 Source Data [file 44318_2025_566_MOESM24_ESM.zip › Fig EV1/Fig EV1A/Dn.KASH/DN.KASH_Before NE rupture_Merge.tif]

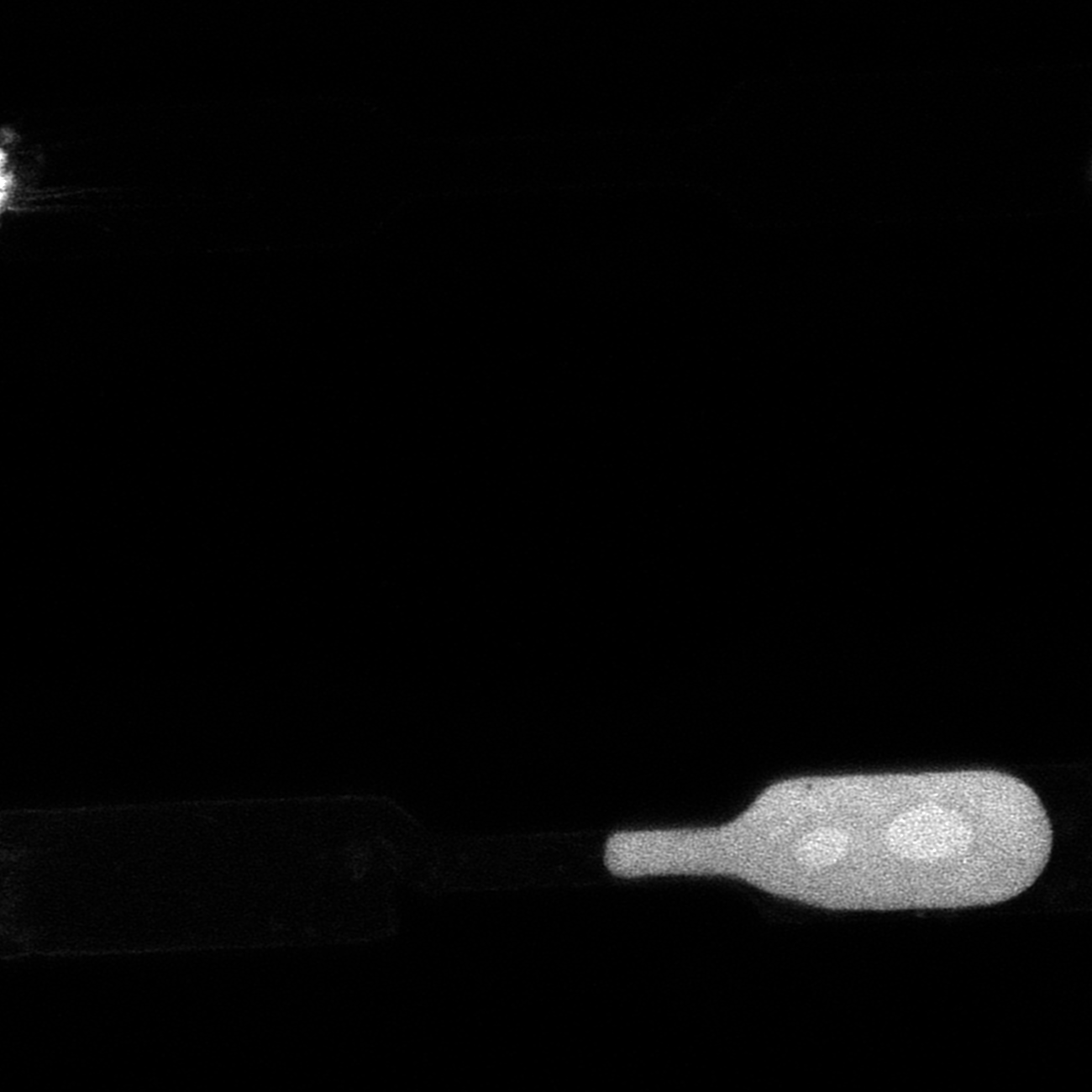

Supplement: Supplementary file 24 — Figure EV1 Source Data [file 44318_2025_566_MOESM24_ESM.zip › Fig EV1/Fig EV1A/Dn.KASH/DN.KASH_Before NE rupture_nAC-GFP.tif]

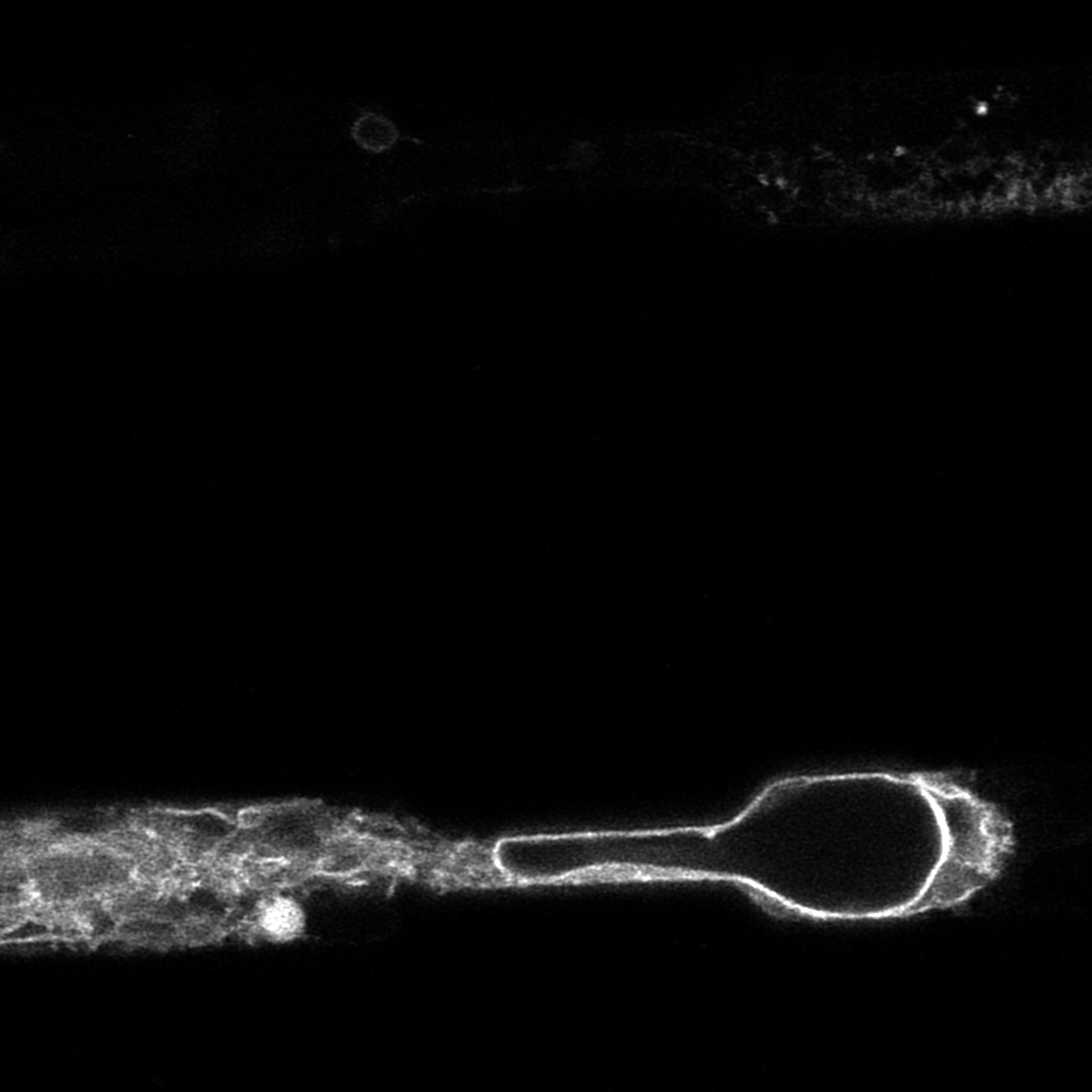

Supplement: Supplementary file 24 — Figure EV1 Source Data [file 44318_2025_566_MOESM24_ESM.zip › Fig EV1/Fig EV1A/Dn.KASH/DN.KASH_NE rupture_DN.KASH.tif]

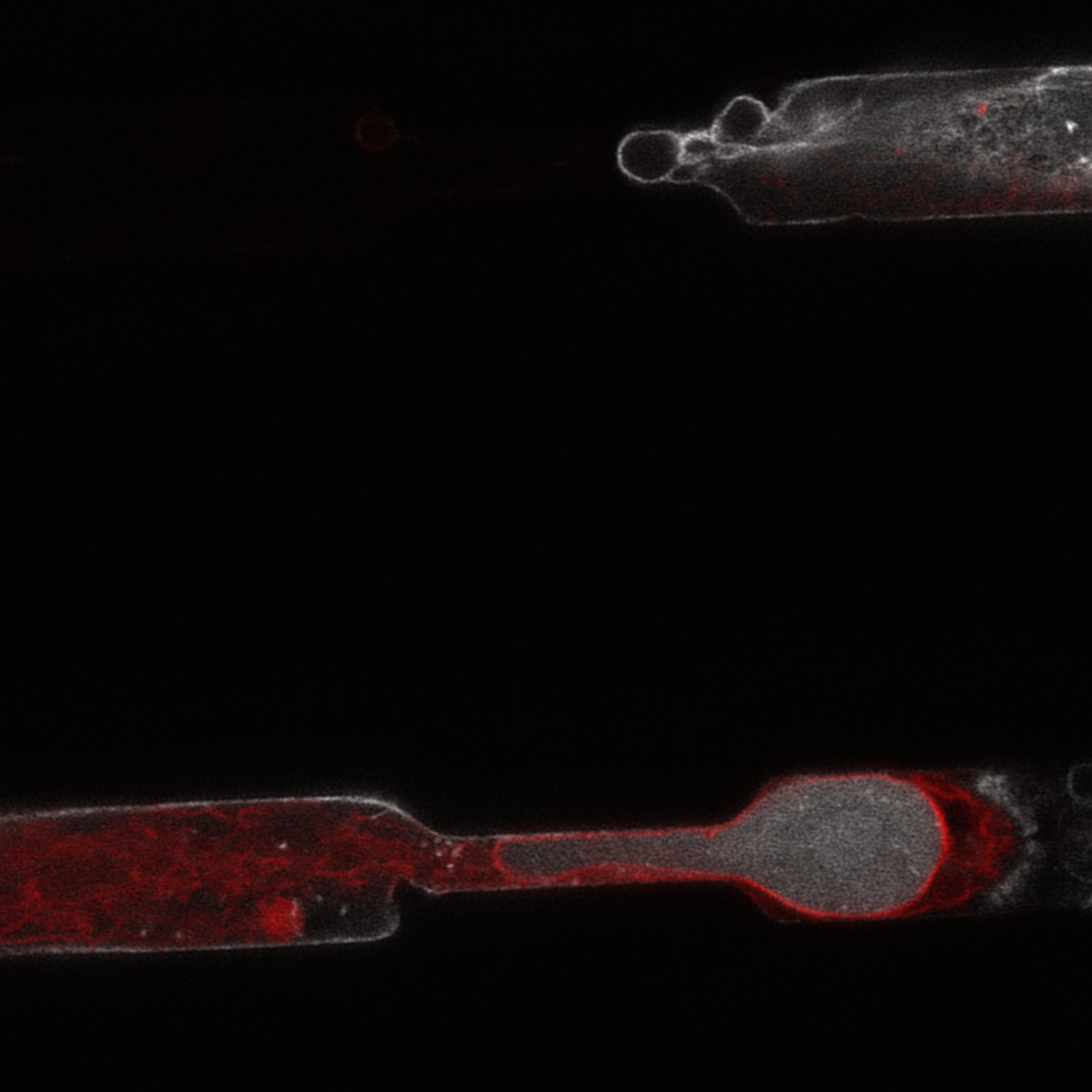

Supplement: Supplementary file 24 — Figure EV1 Source Data [file 44318_2025_566_MOESM24_ESM.zip › Fig EV1/Fig EV1A/Dn.KASH/DN.KASH_NE rupture_Merge.tif]

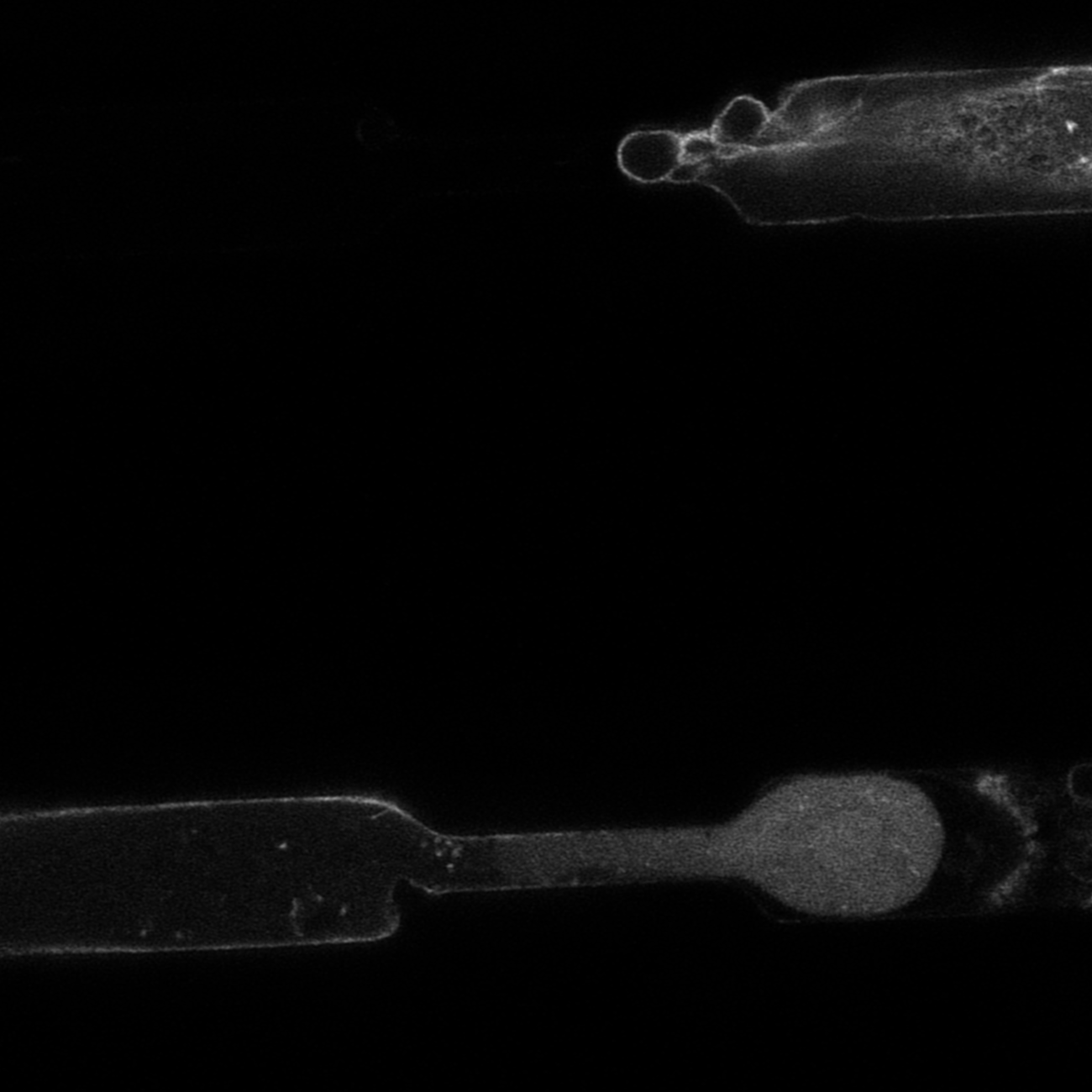

Supplement: Supplementary file 24 — Figure EV1 Source Data [file 44318_2025_566_MOESM24_ESM.zip › Fig EV1/Fig EV1A/Dn.KASH/DN.KASH_NE rupture_nAC-GFP.tif]

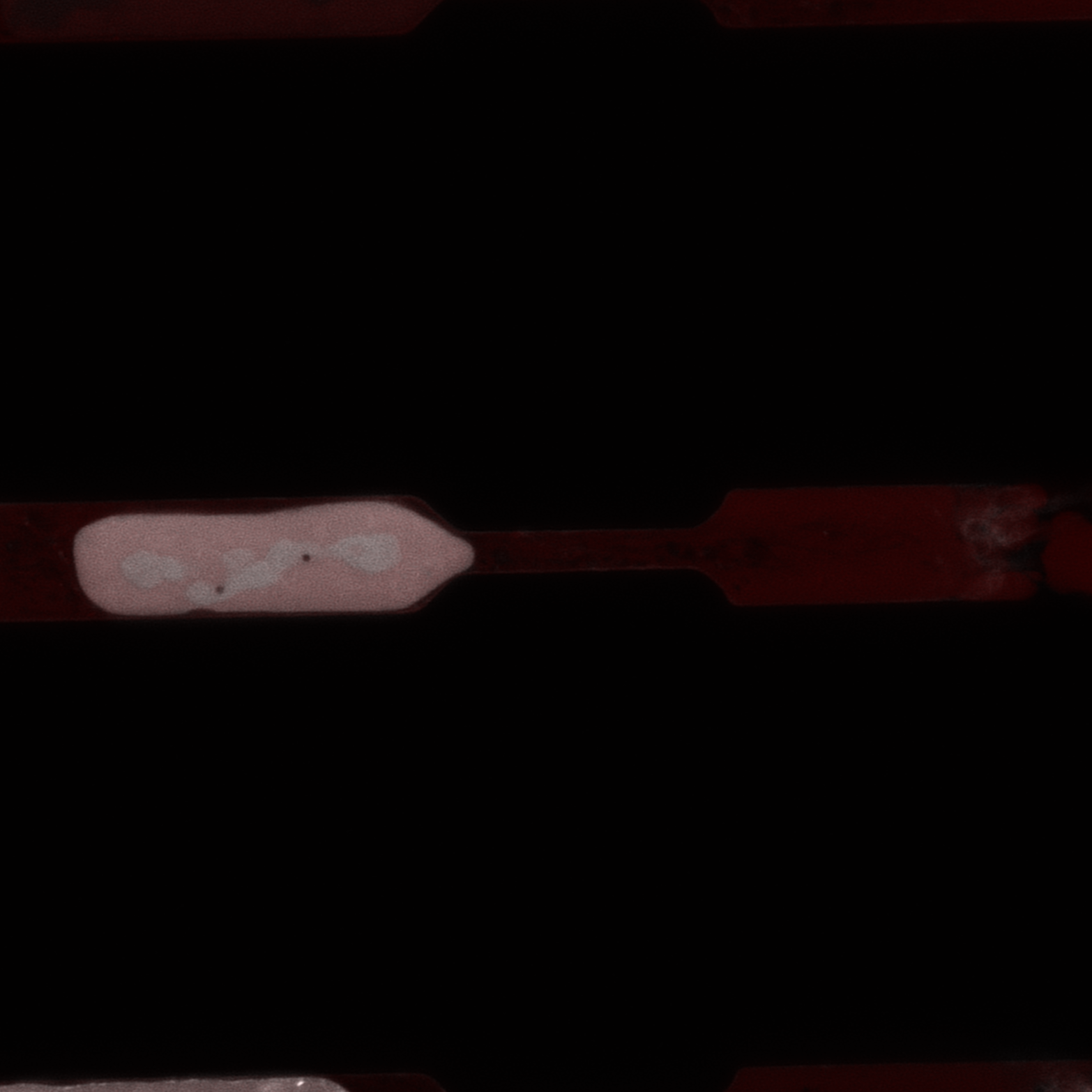

Supplement: Supplementary file 24 — Figure EV1 Source Data [file 44318_2025_566_MOESM24_ESM.zip › Fig EV1/Fig EV1A/TdTomato/Merge_Before NE rupture.tif]

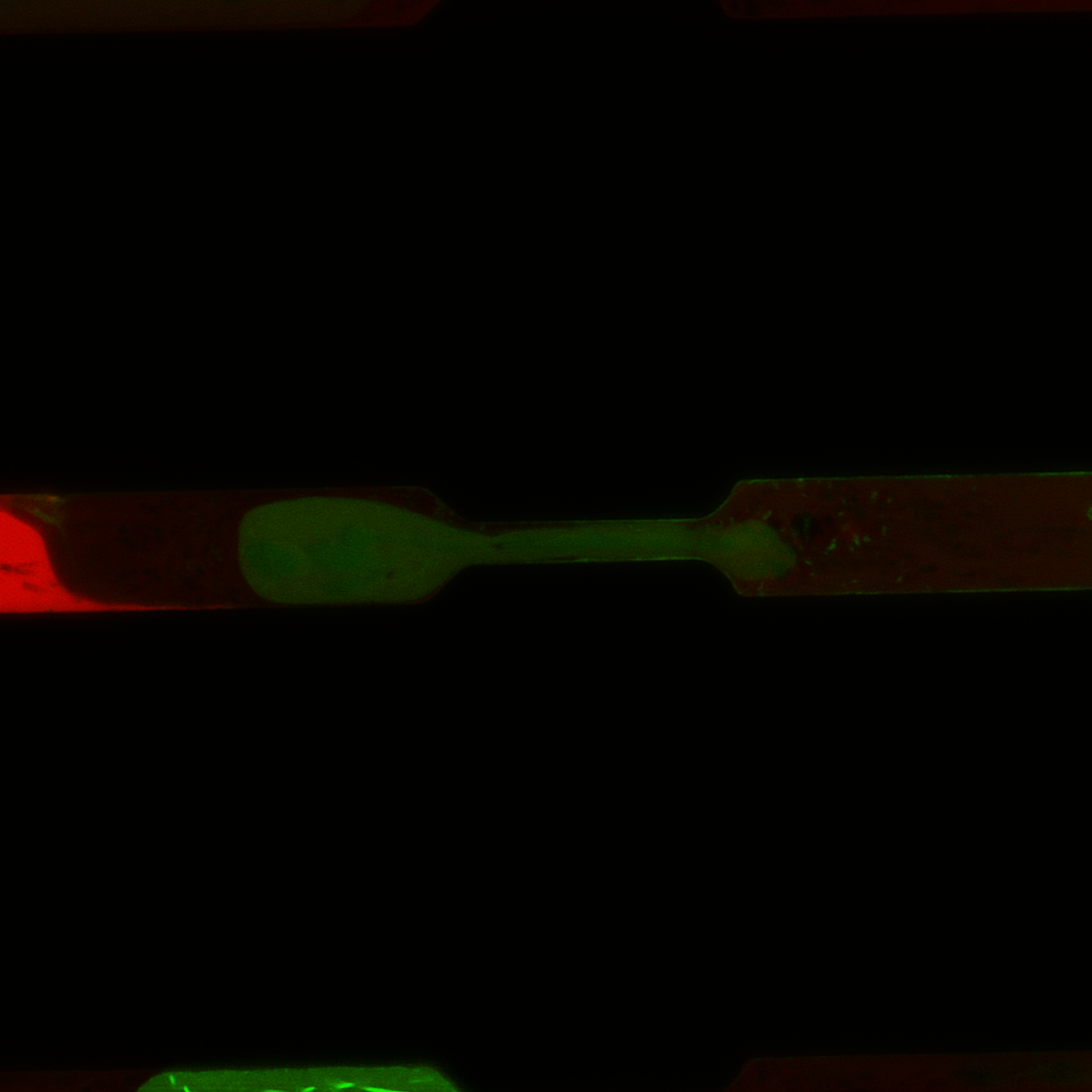

Supplement: Supplementary file 24 — Figure EV1 Source Data [file 44318_2025_566_MOESM24_ESM.zip › Fig EV1/Fig EV1A/TdTomato/Merge_NE rupture.tif]

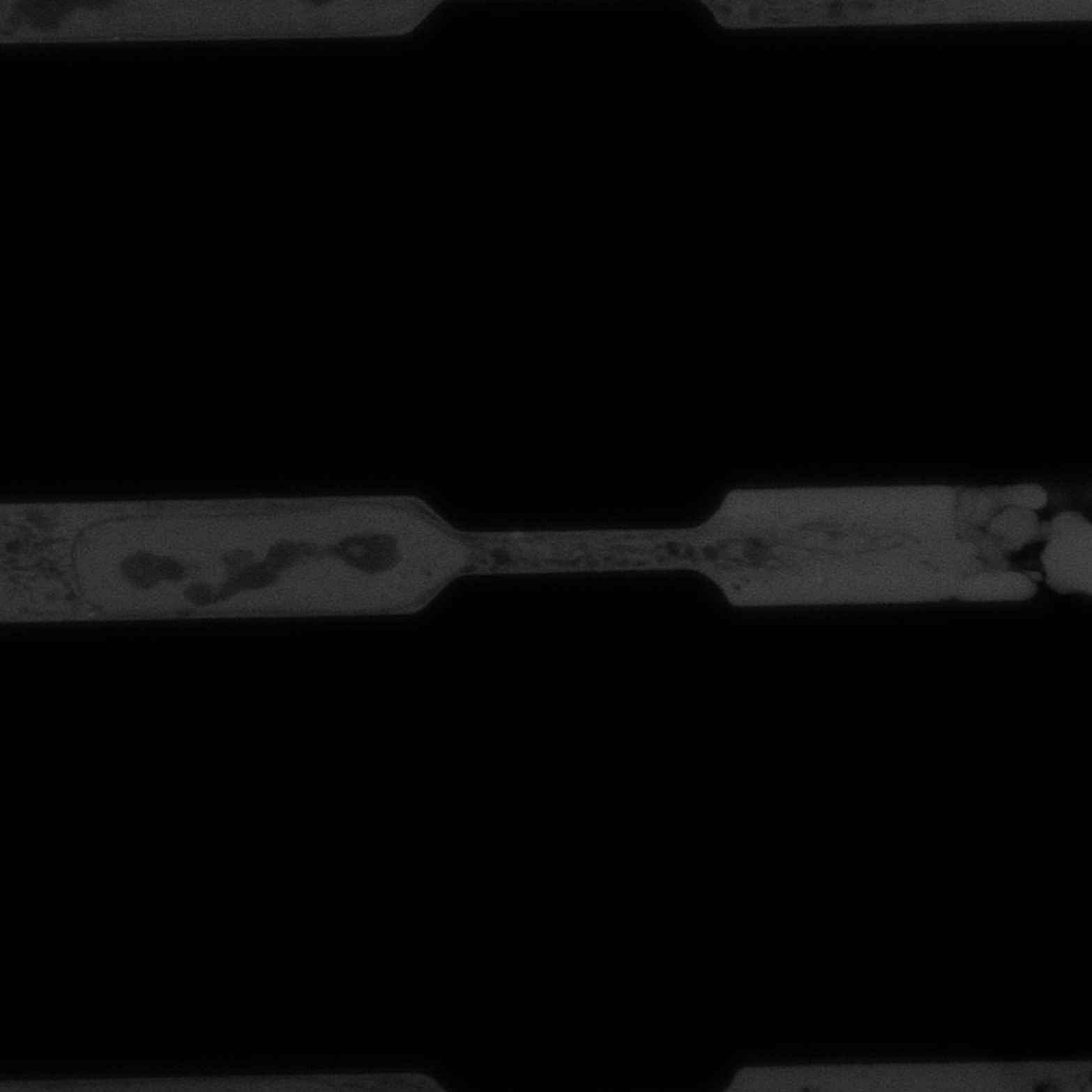

Supplement: Supplementary file 24 — Figure EV1 Source Data [file 44318_2025_566_MOESM24_ESM.zip › Fig EV1/Fig EV1A/TdTomato/TdTomato_Before NE rupture.tif]

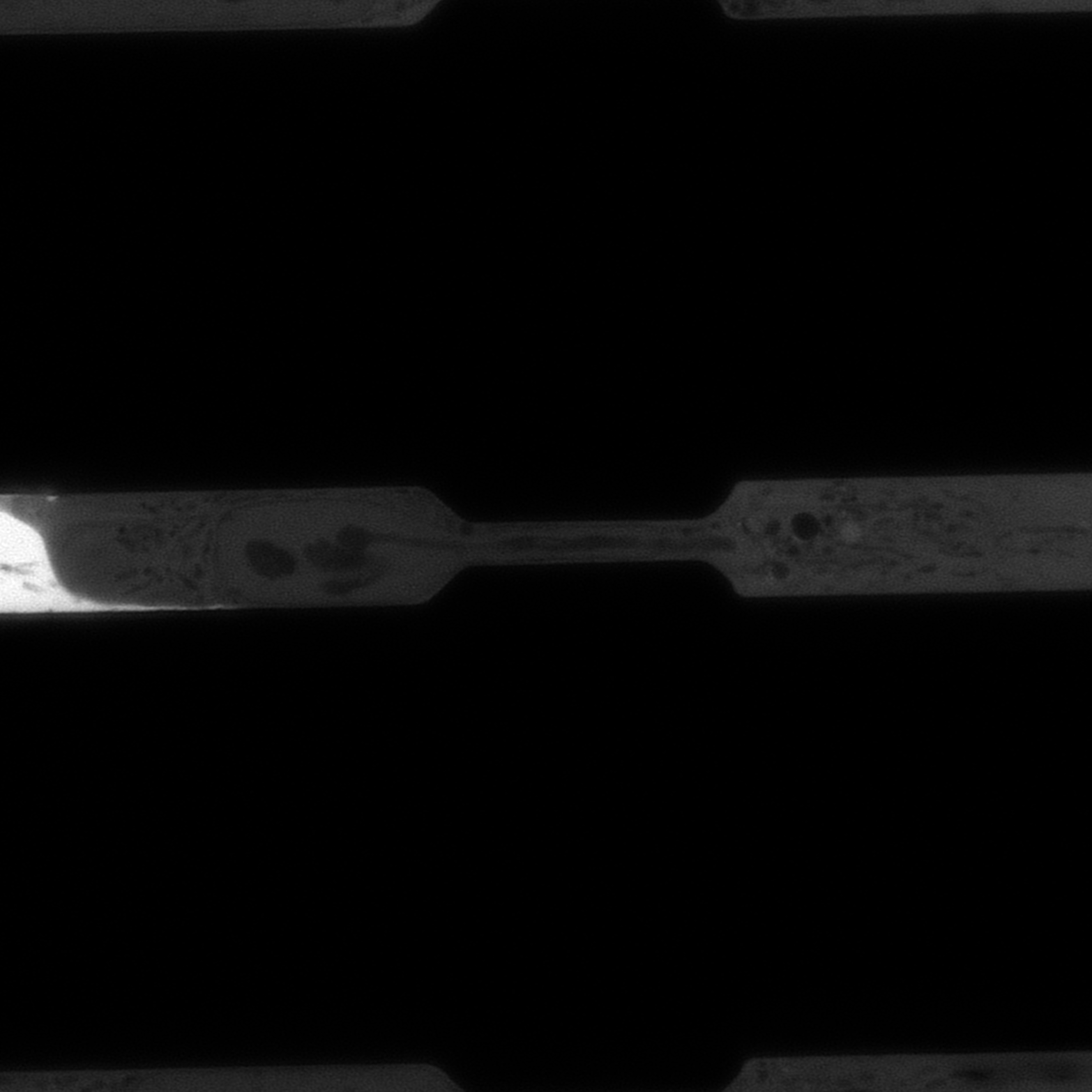

Supplement: Supplementary file 24 — Figure EV1 Source Data [file 44318_2025_566_MOESM24_ESM.zip › Fig EV1/Fig EV1A/TdTomato/TdTomato_NE rupture.tif]

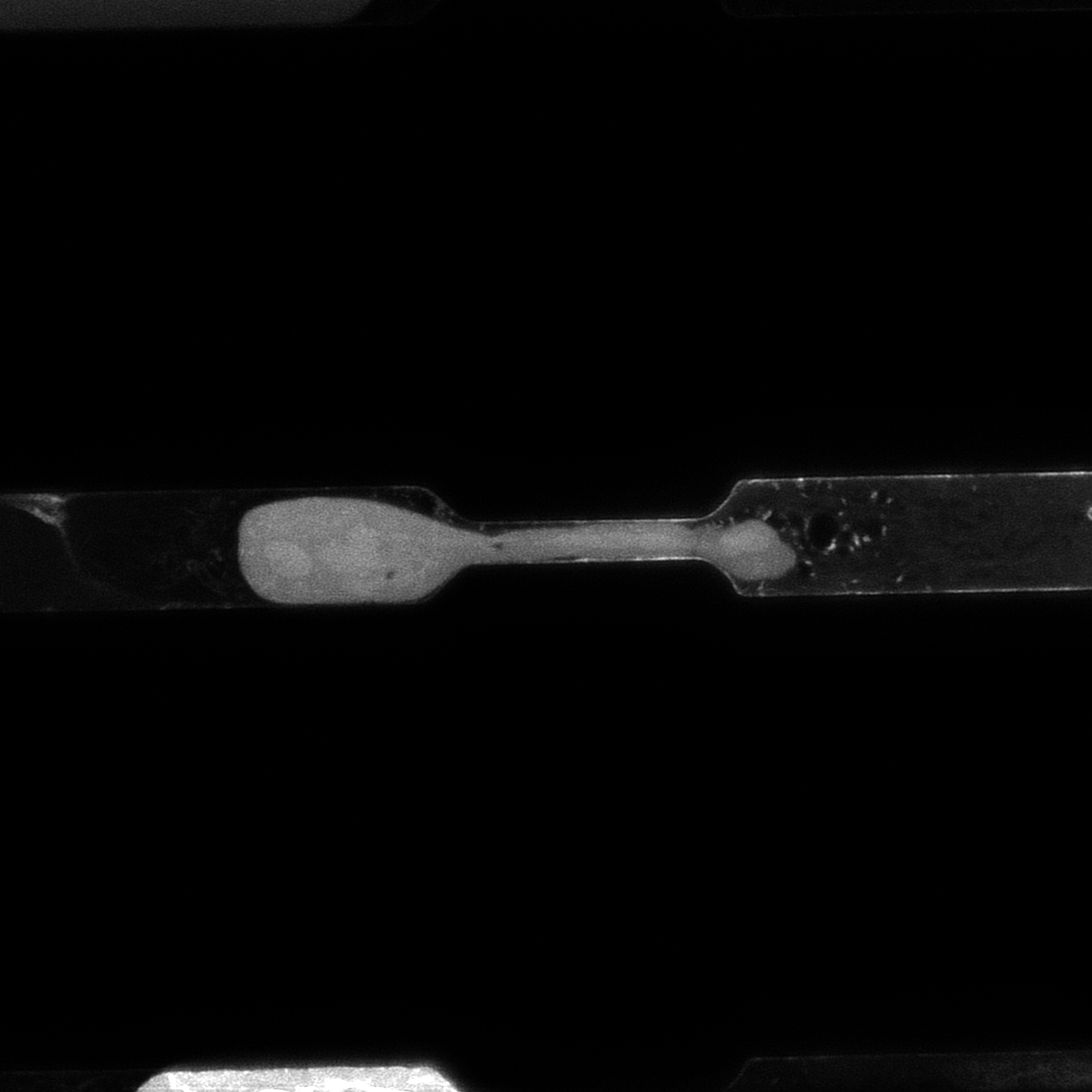

Supplement: Supplementary file 24 — Figure EV1 Source Data [file 44318_2025_566_MOESM24_ESM.zip › Fig EV1/Fig EV1A/TdTomato/TdTomato_NE rupture_nAC-GFP.tif]

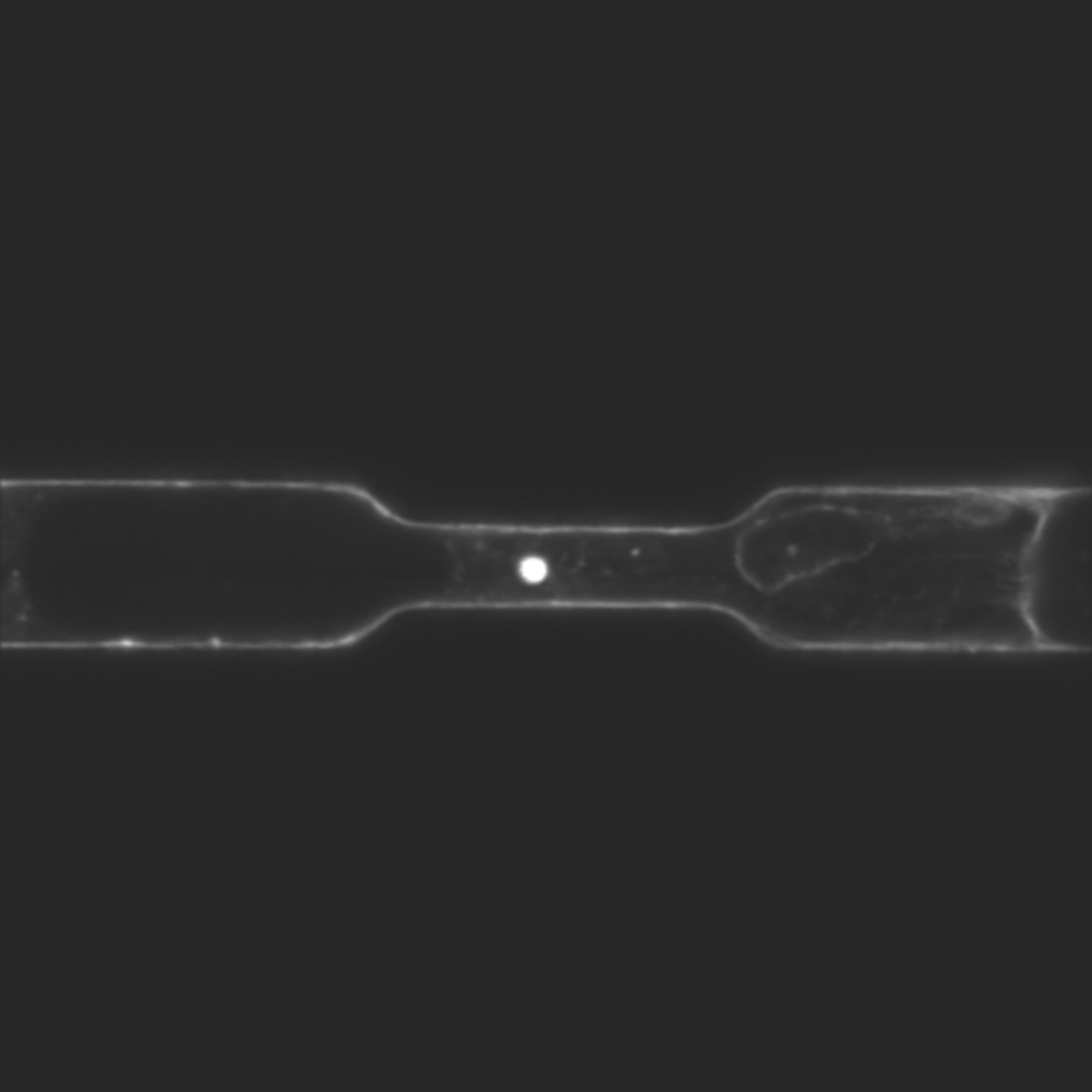

Supplement: Supplementary file 24 — Figure EV1 Source Data [file 44318_2025_566_MOESM24_ESM.zip › Fig EV1/Fig EV1C/FastAct_Before NE rupture.tif]

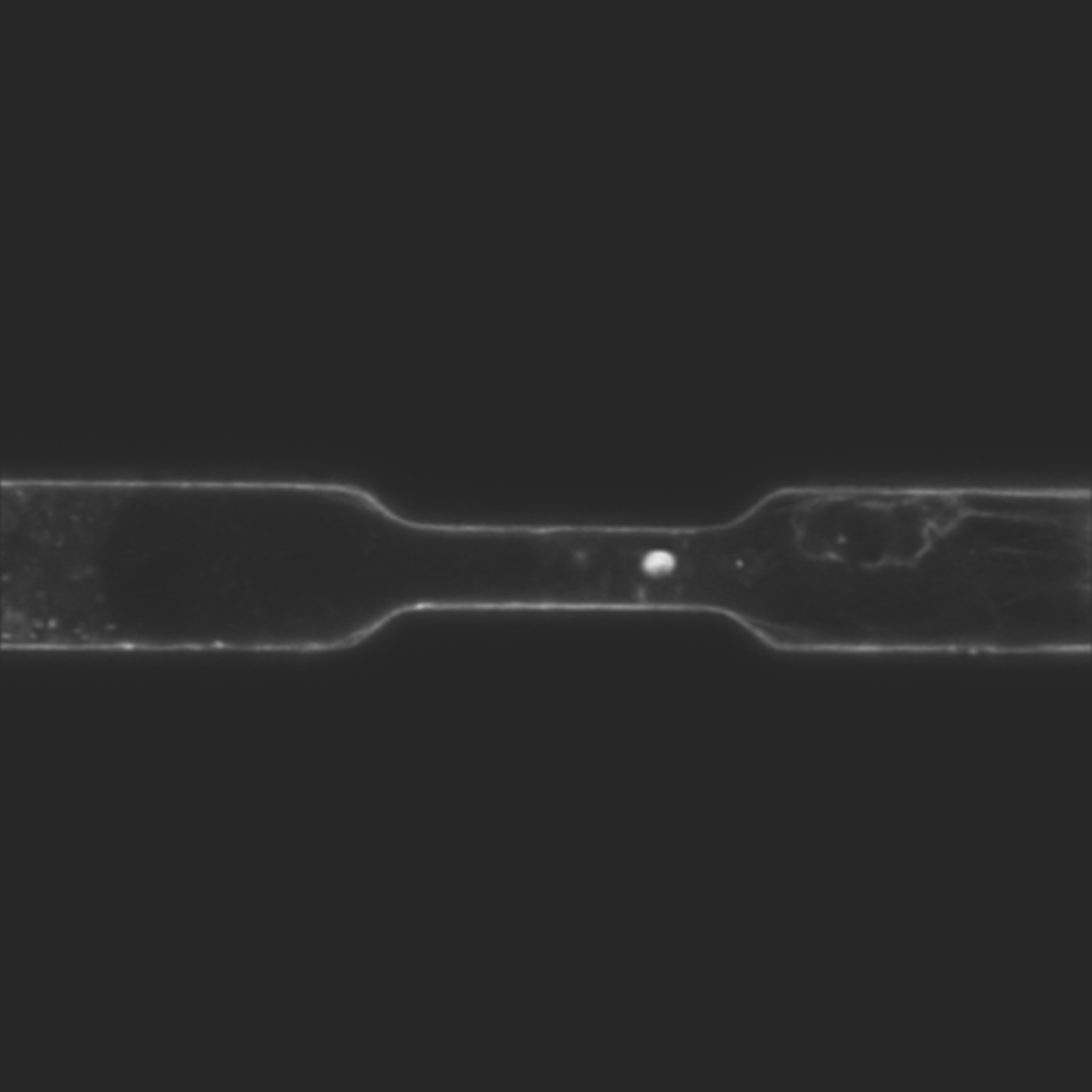

Supplement: Supplementary file 24 — Figure EV1 Source Data [file 44318_2025_566_MOESM24_ESM.zip › Fig EV1/Fig EV1C/FastAct_NE rupture.tif]

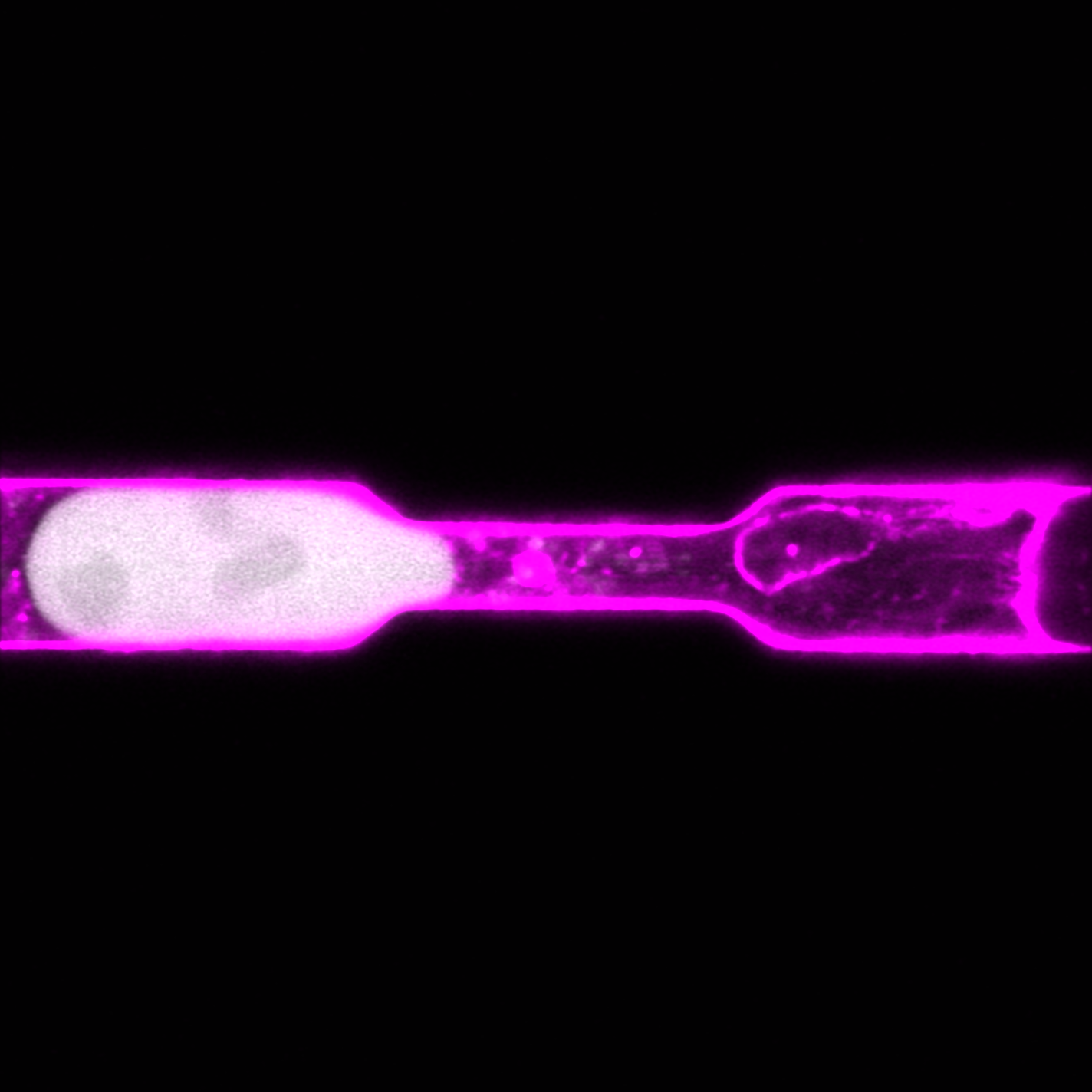

Supplement: Supplementary file 24 — Figure EV1 Source Data [file 44318_2025_566_MOESM24_ESM.zip › Fig EV1/Fig EV1C/Merge_Before NE rupture.tif]

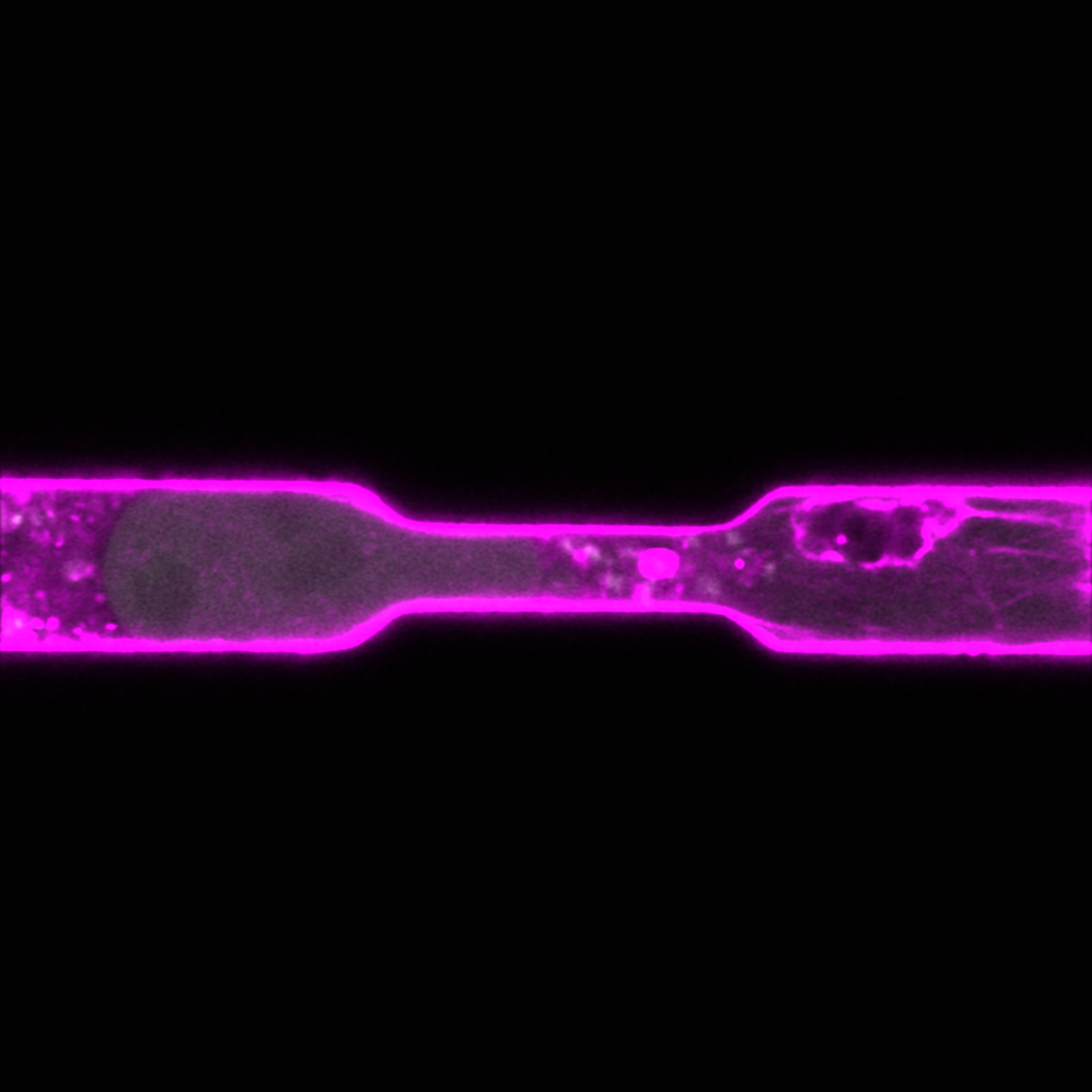

Supplement: Supplementary file 24 — Figure EV1 Source Data [file 44318_2025_566_MOESM24_ESM.zip › Fig EV1/Fig EV1C/Merge_NE rupture.tif]

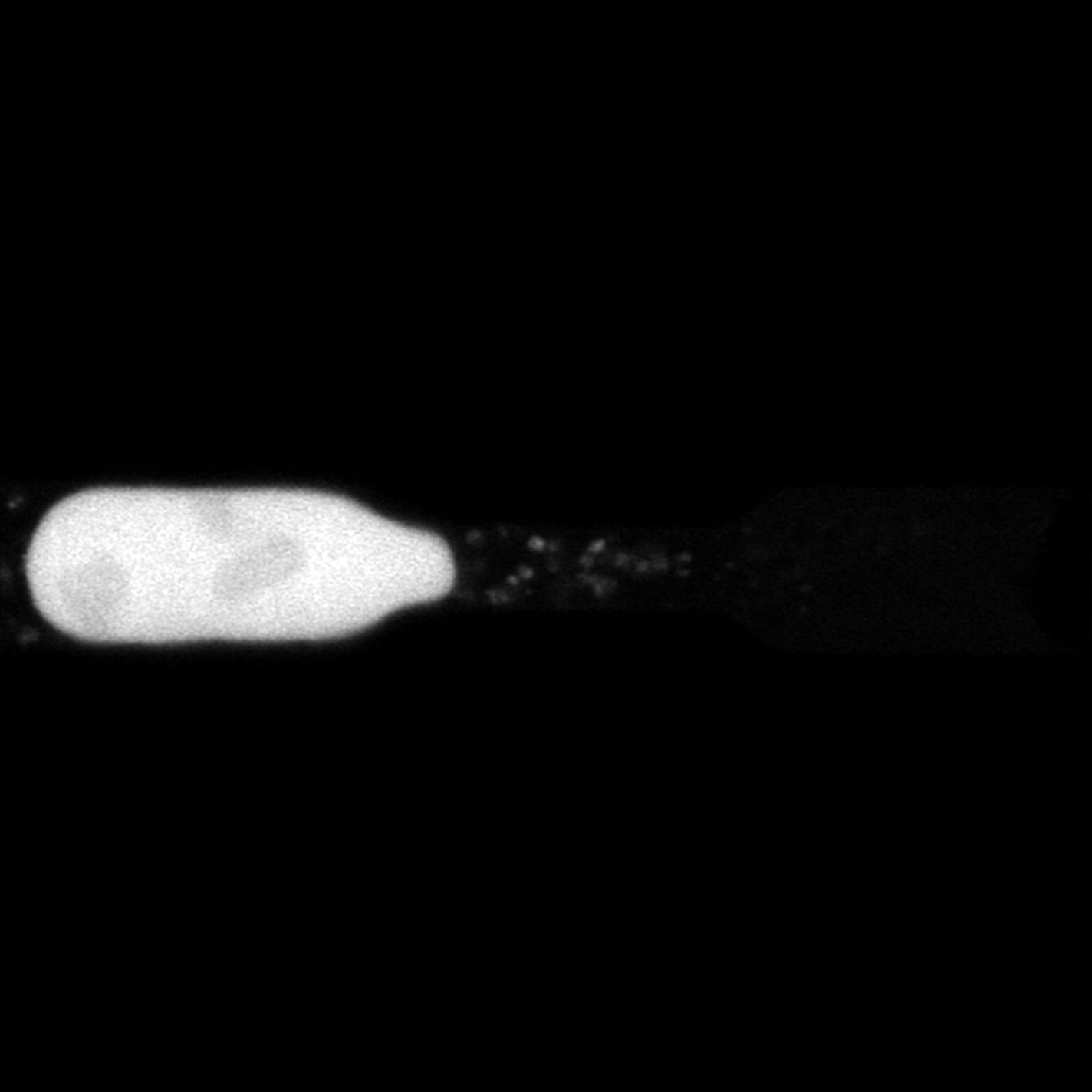

Supplement: Supplementary file 24 — Figure EV1 Source Data [file 44318_2025_566_MOESM24_ESM.zip › Fig EV1/Fig EV1C/NLS-BFP_Before NE rupture.tif]

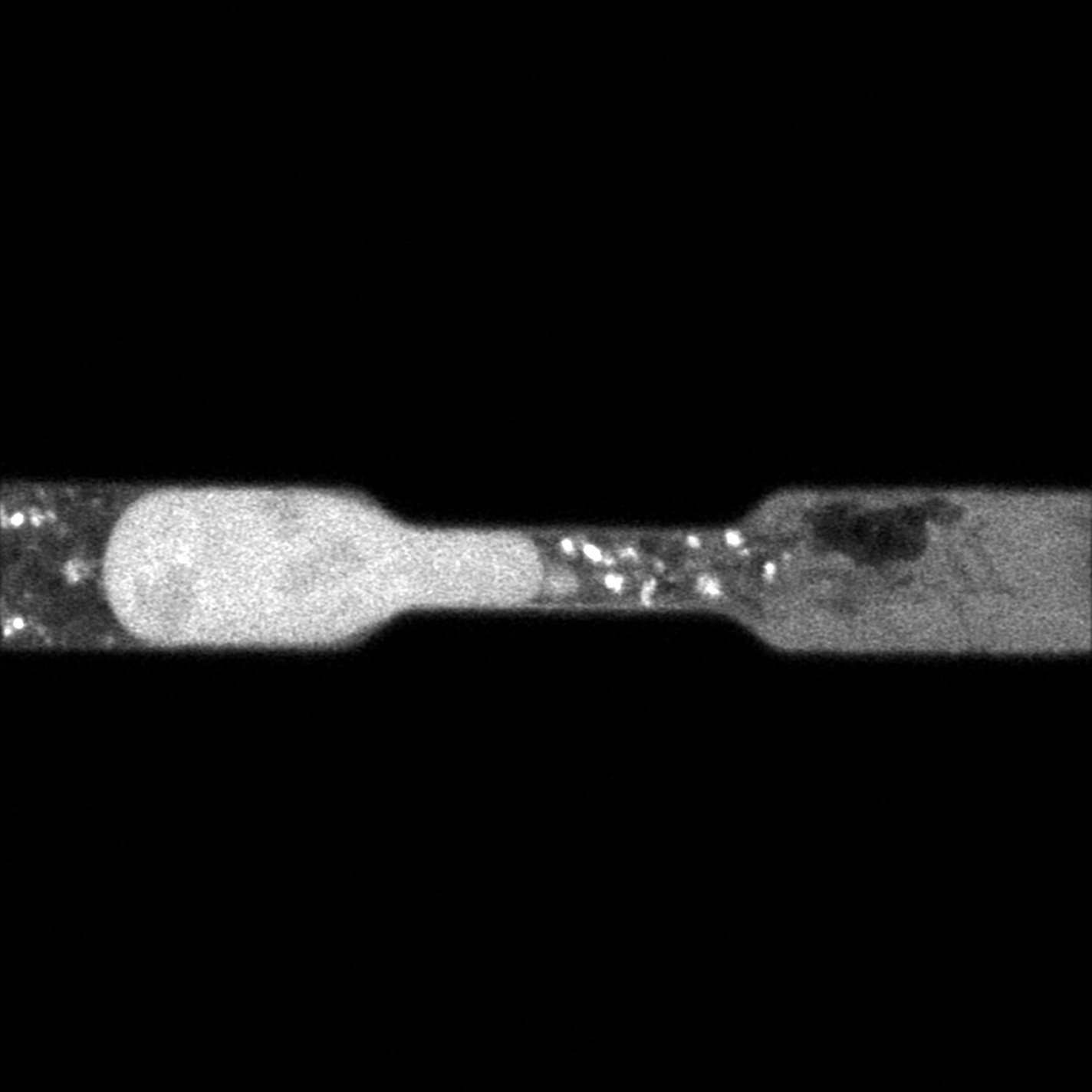

Supplement: Supplementary file 24 — Figure EV1 Source Data [file 44318_2025_566_MOESM24_ESM.zip › Fig EV1/Fig EV1C/NLS-BFP_NE rupture.tif]

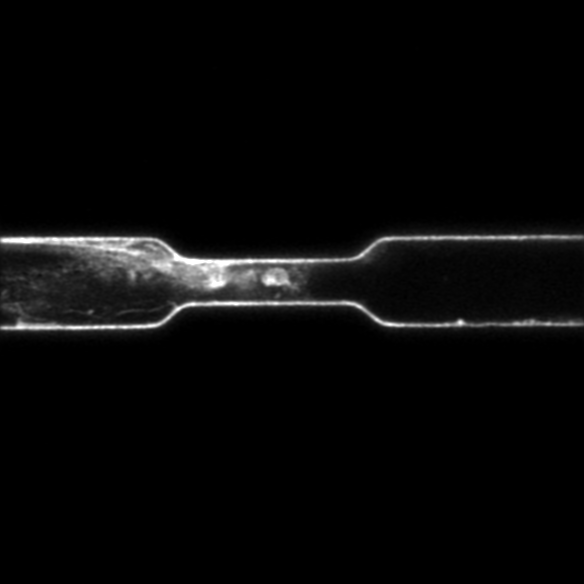

Supplement: Supplementary file 24 — Figure EV1 Source Data [file 44318_2025_566_MOESM24_ESM.zip › Fig EV1/Fig EV1D/Before NE rupture_FastAct.tif]

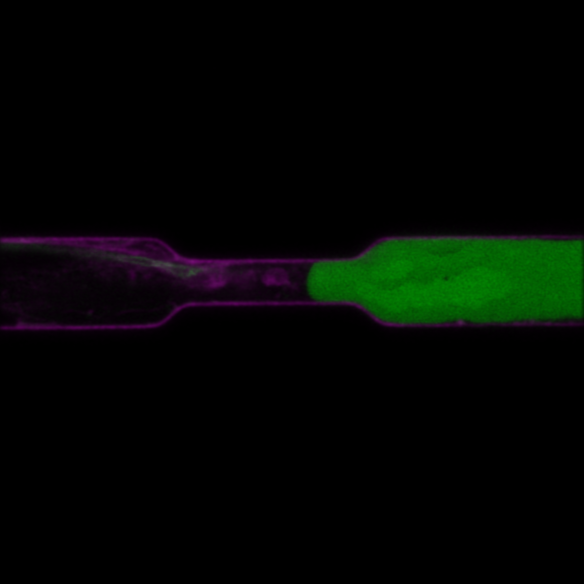

Supplement: Supplementary file 24 — Figure EV1 Source Data [file 44318_2025_566_MOESM24_ESM.zip › Fig EV1/Fig EV1D/Before NE rupture_Merge.tif]

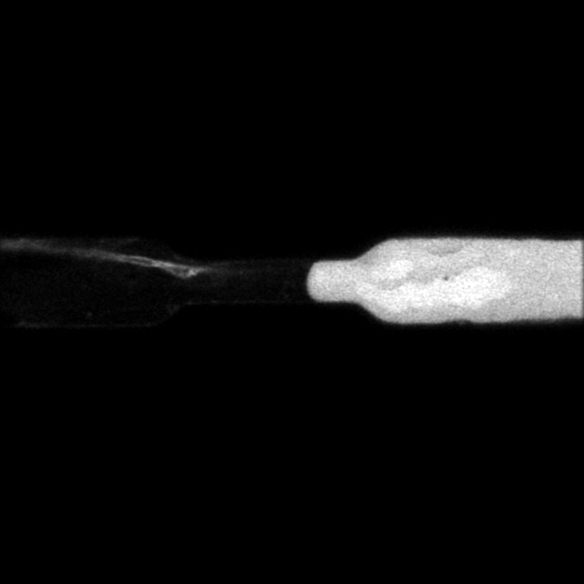

Supplement: Supplementary file 24 — Figure EV1 Source Data [file 44318_2025_566_MOESM24_ESM.zip › Fig EV1/Fig EV1D/Before NE rupture_nAC-GFP.tif]

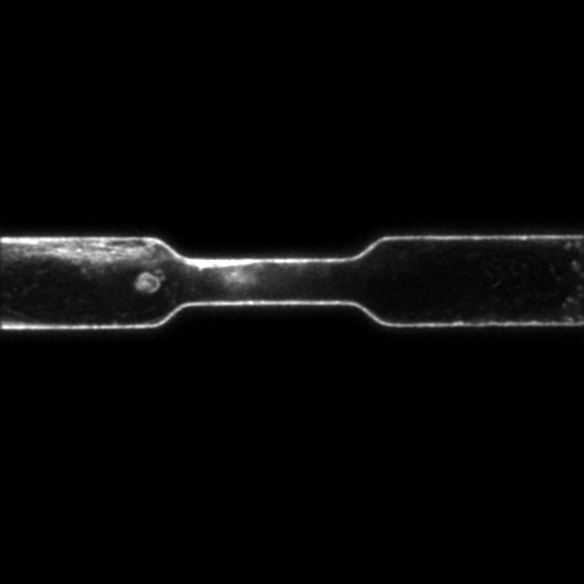

Supplement: Supplementary file 24 — Figure EV1 Source Data [file 44318_2025_566_MOESM24_ESM.zip › Fig EV1/Fig EV1D/NE rupture_FastAct.tif]

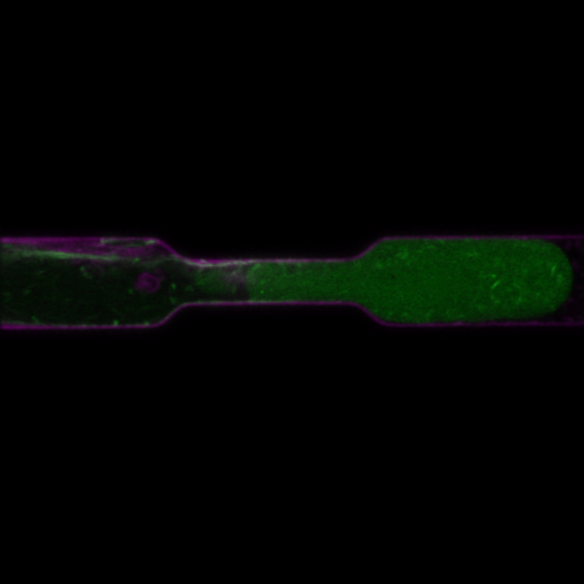

Supplement: Supplementary file 24 — Figure EV1 Source Data [file 44318_2025_566_MOESM24_ESM.zip › Fig EV1/Fig EV1D/NE rupture_Merge.tif]

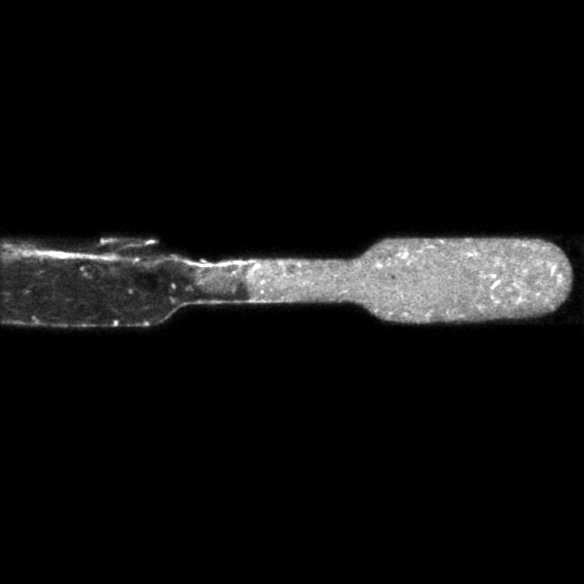

Supplement: Supplementary file 24 — Figure EV1 Source Data [file 44318_2025_566_MOESM24_ESM.zip › Fig EV1/Fig EV1D/NE rupture_nAC-GFP.tif]

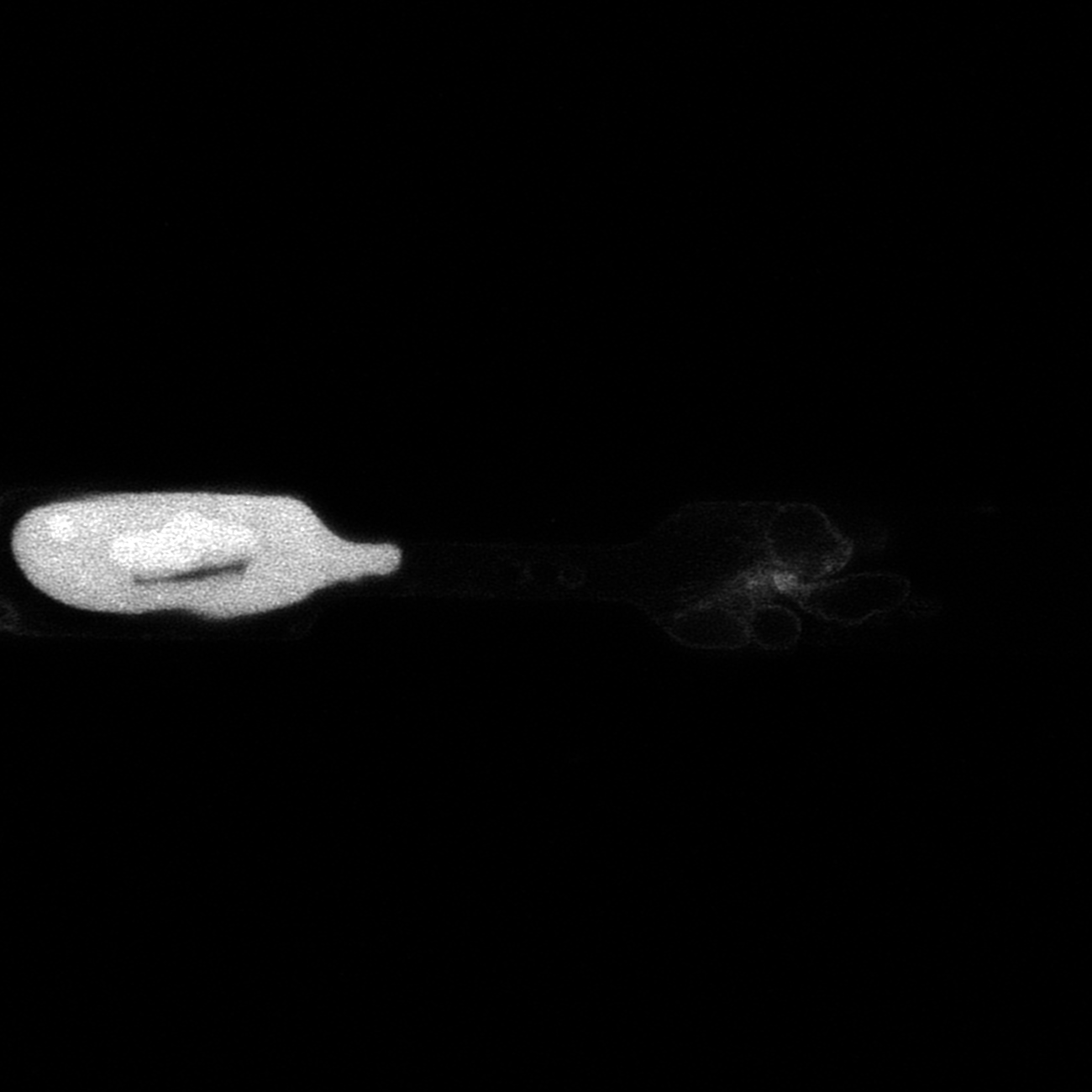

Supplement: Supplementary file 25 — Figure EV2 Source Data [file 44318_2025_566_MOESM25_ESM.zip › Fig EV2/Fig EV2A/CK-666_Before NE rupture.tif]

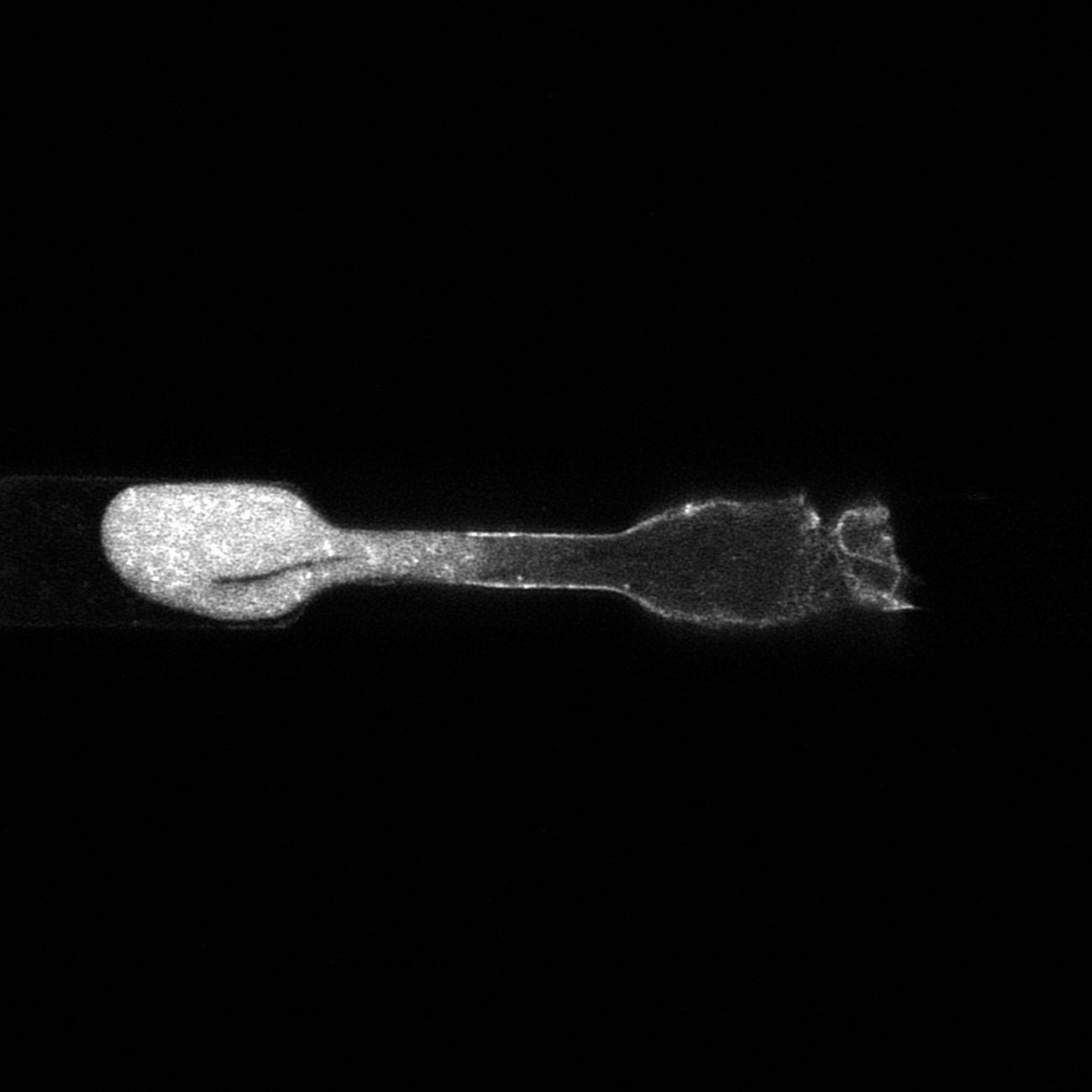

Supplement: Supplementary file 25 — Figure EV2 Source Data [file 44318_2025_566_MOESM25_ESM.zip › Fig EV2/Fig EV2A/CK-666_NE rupture.tif]

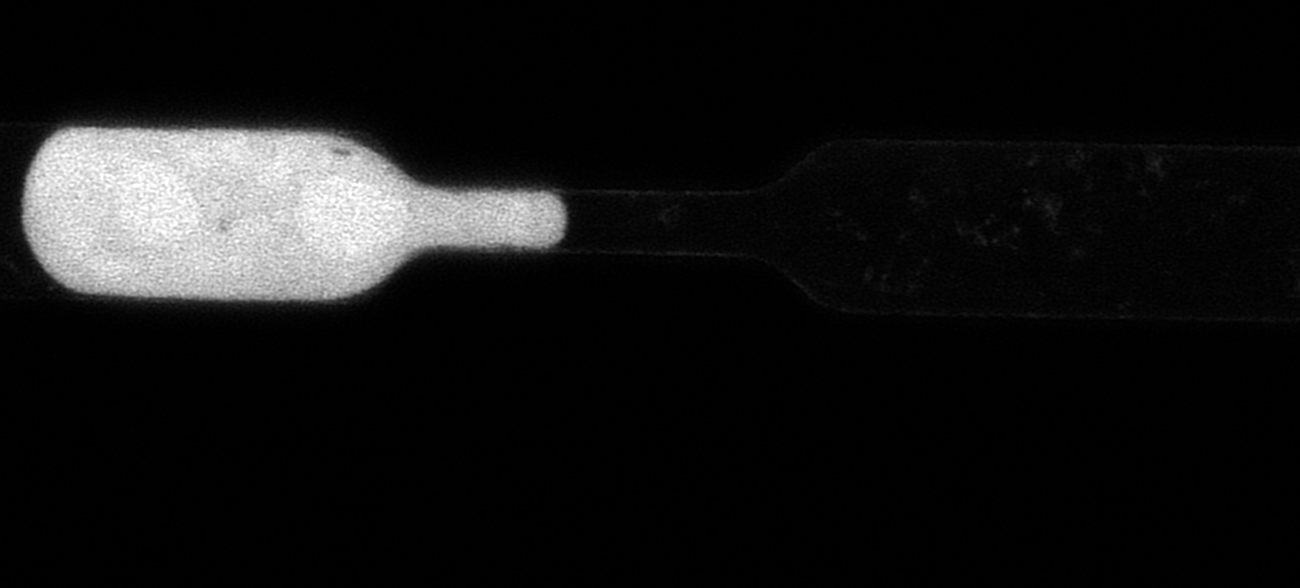

Supplement: Supplementary file 25 — Figure EV2 Source Data [file 44318_2025_566_MOESM25_ESM.zip › Fig EV2/Fig EV2A/DMSO_Before NE rupture.tif]

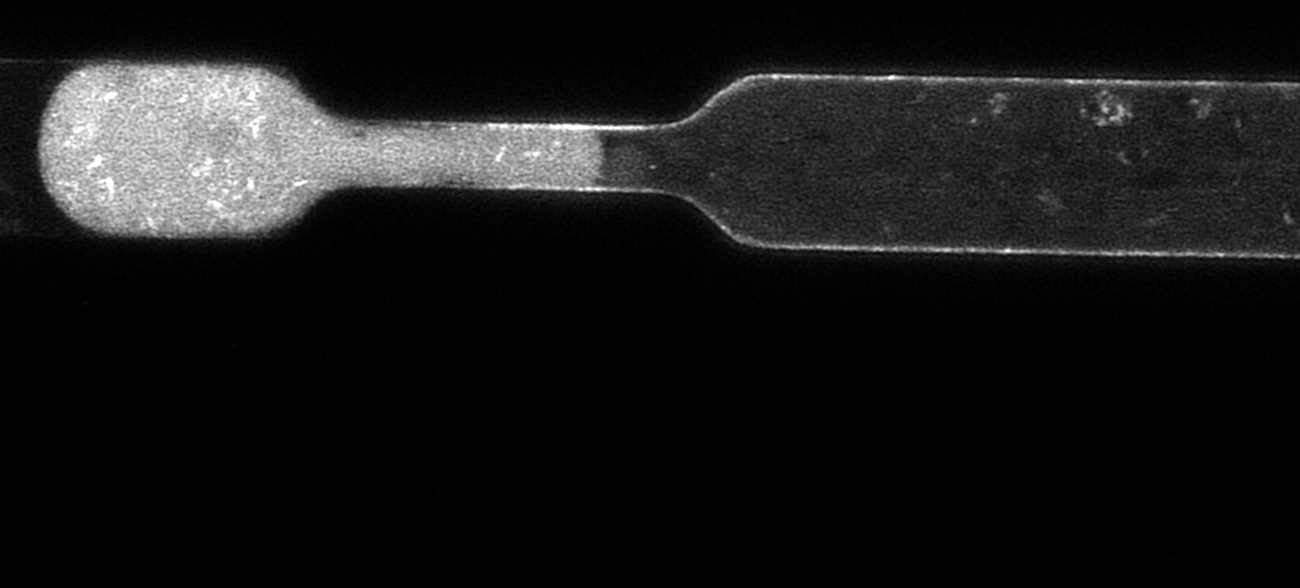

Supplement: Supplementary file 25 — Figure EV2 Source Data [file 44318_2025_566_MOESM25_ESM.zip › Fig EV2/Fig EV2A/DMSO_NE rupture.tif]

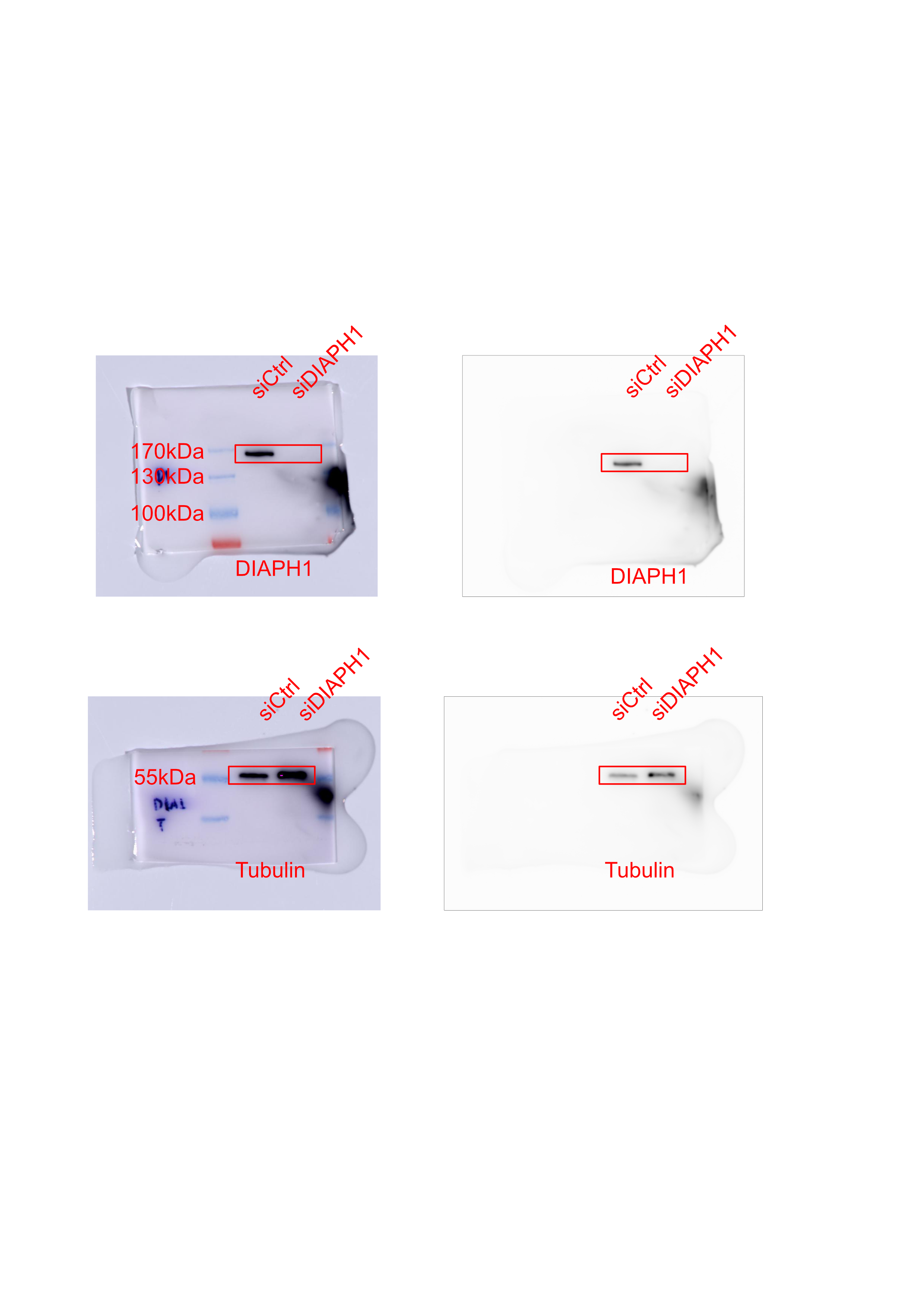

Supplement: Supplementary file 25 — Figure EV2 Source Data [file 44318_2025_566_MOESM25_ESM.zip › Fig EV2/Fig EV2C/Fig EV3C - siDIAPH1.tiff]

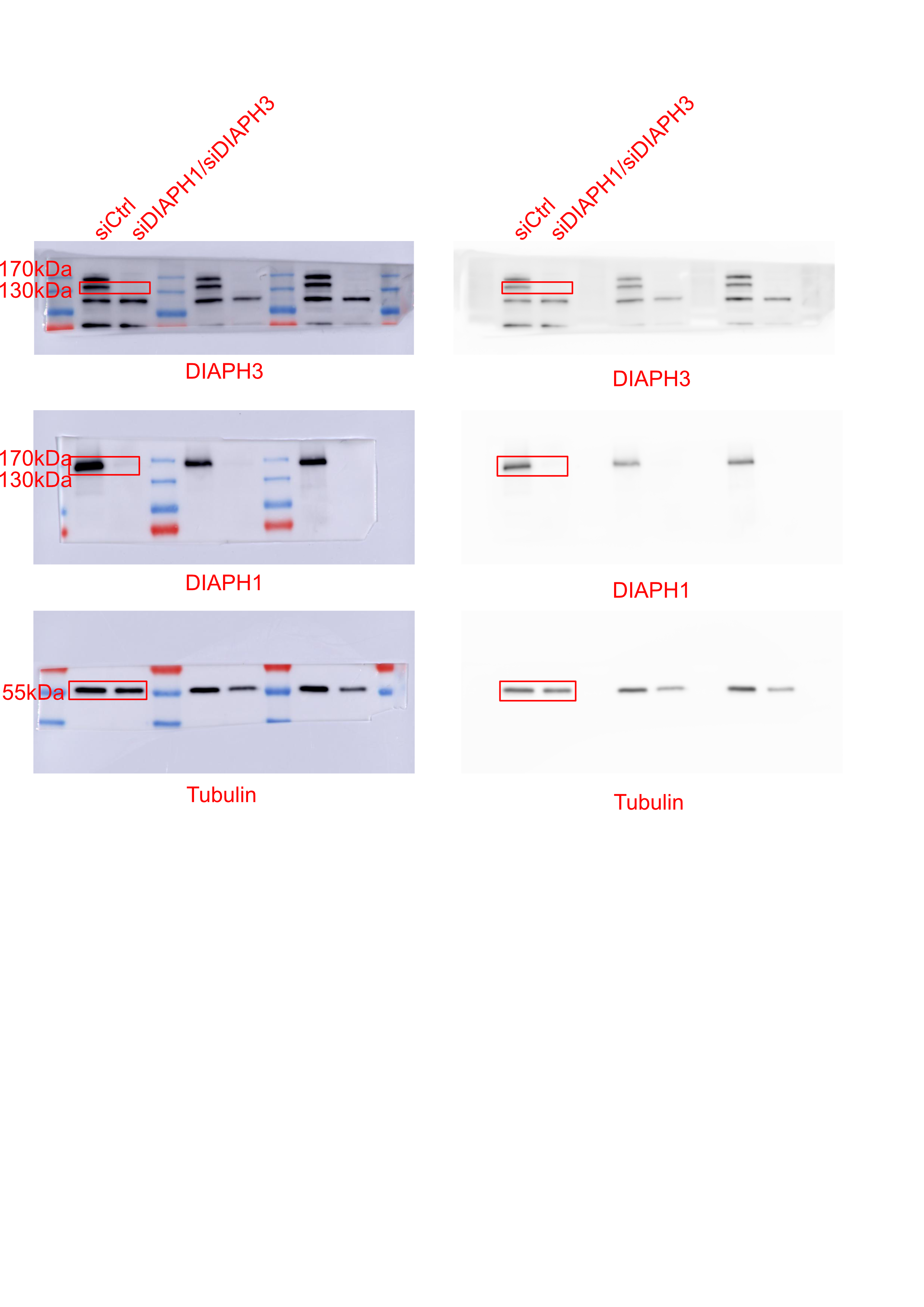

Supplement: Supplementary file 25 — Figure EV2 Source Data [file 44318_2025_566_MOESM25_ESM.zip › Fig EV2/Fig EV2C/Fig EV3C - siDIAPH1_3.tiff]

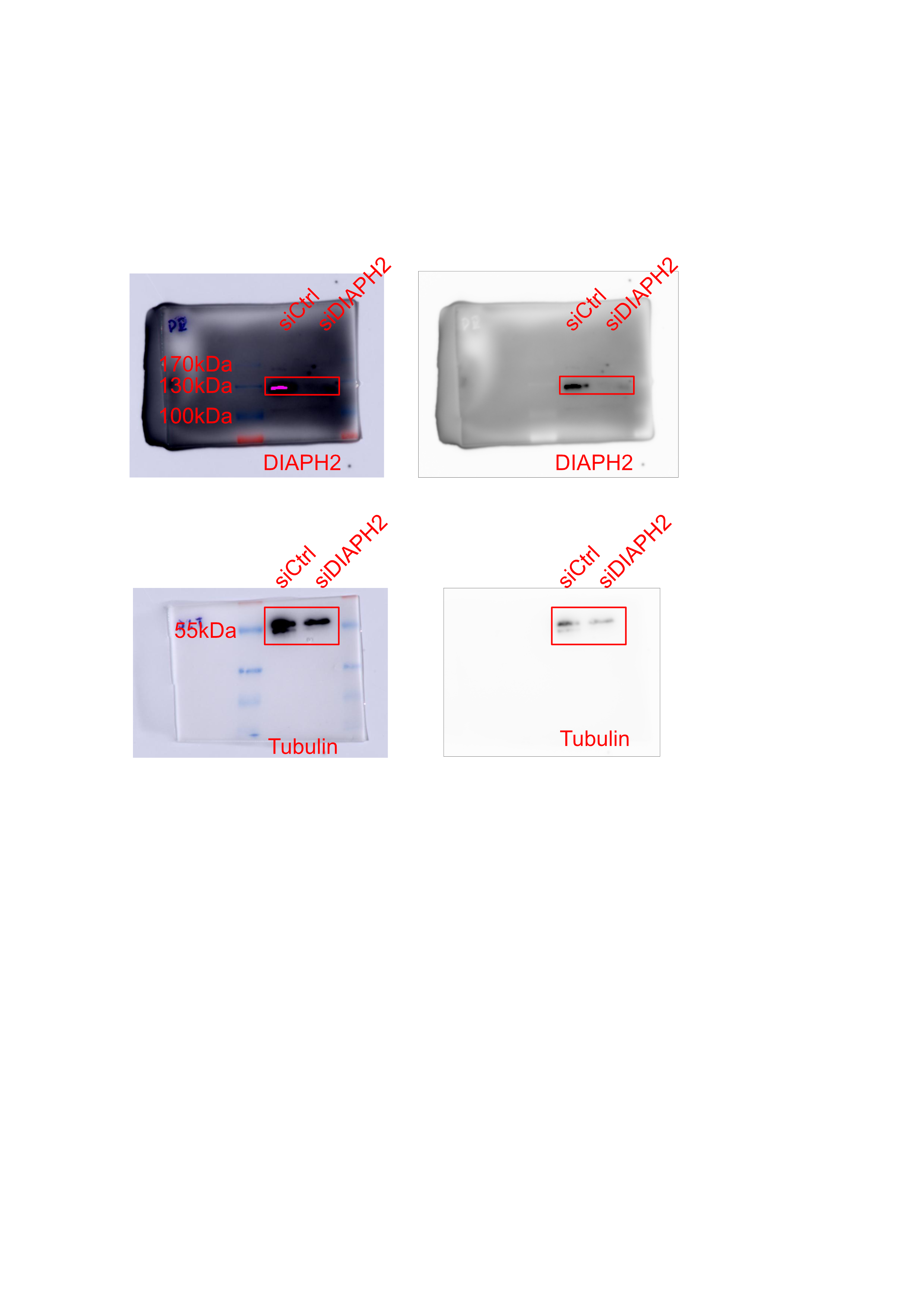

Supplement: Supplementary file 25 — Figure EV2 Source Data [file 44318_2025_566_MOESM25_ESM.zip › Fig EV2/Fig EV2C/Fig EV3C - siDIAPH2.tiff]

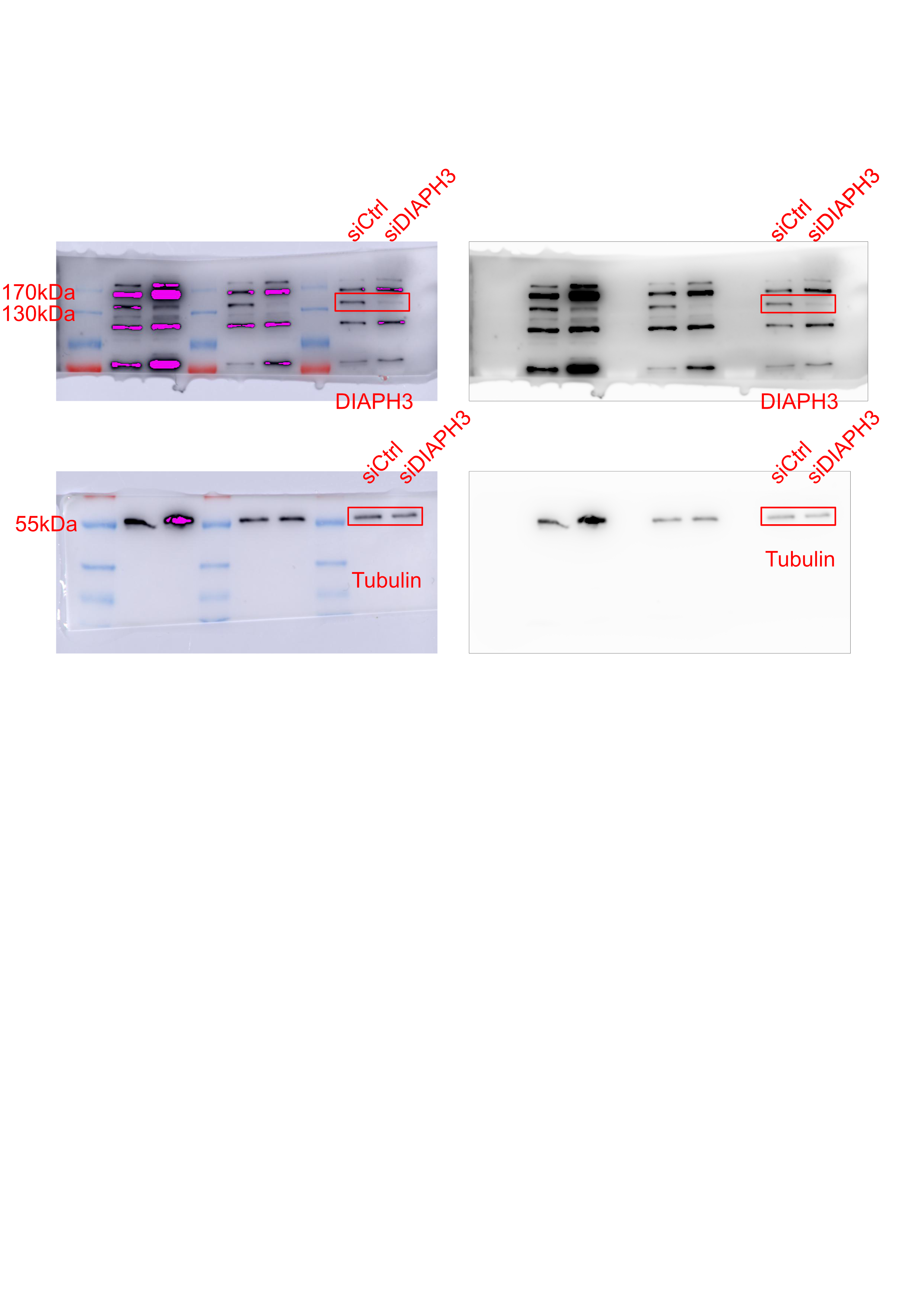

Supplement: Supplementary file 25 — Figure EV2 Source Data [file 44318_2025_566_MOESM25_ESM.zip › Fig EV2/Fig EV2C/Fig EV3C - siDIAPH3.tiff]

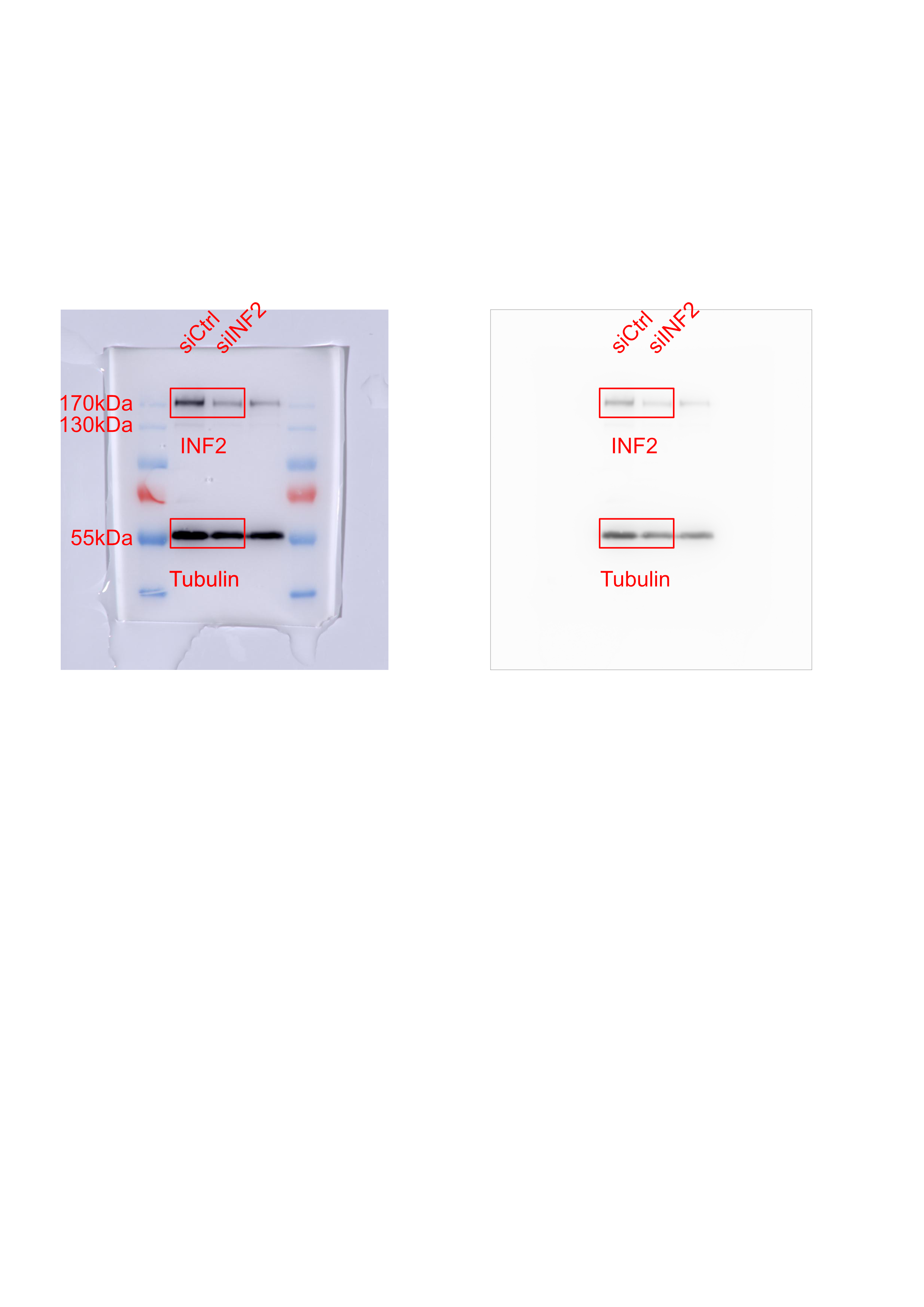

Supplement: Supplementary file 25 — Figure EV2 Source Data [file 44318_2025_566_MOESM25_ESM.zip › Fig EV2/Fig EV2C/Fig EV3C - siINF2.tiff]

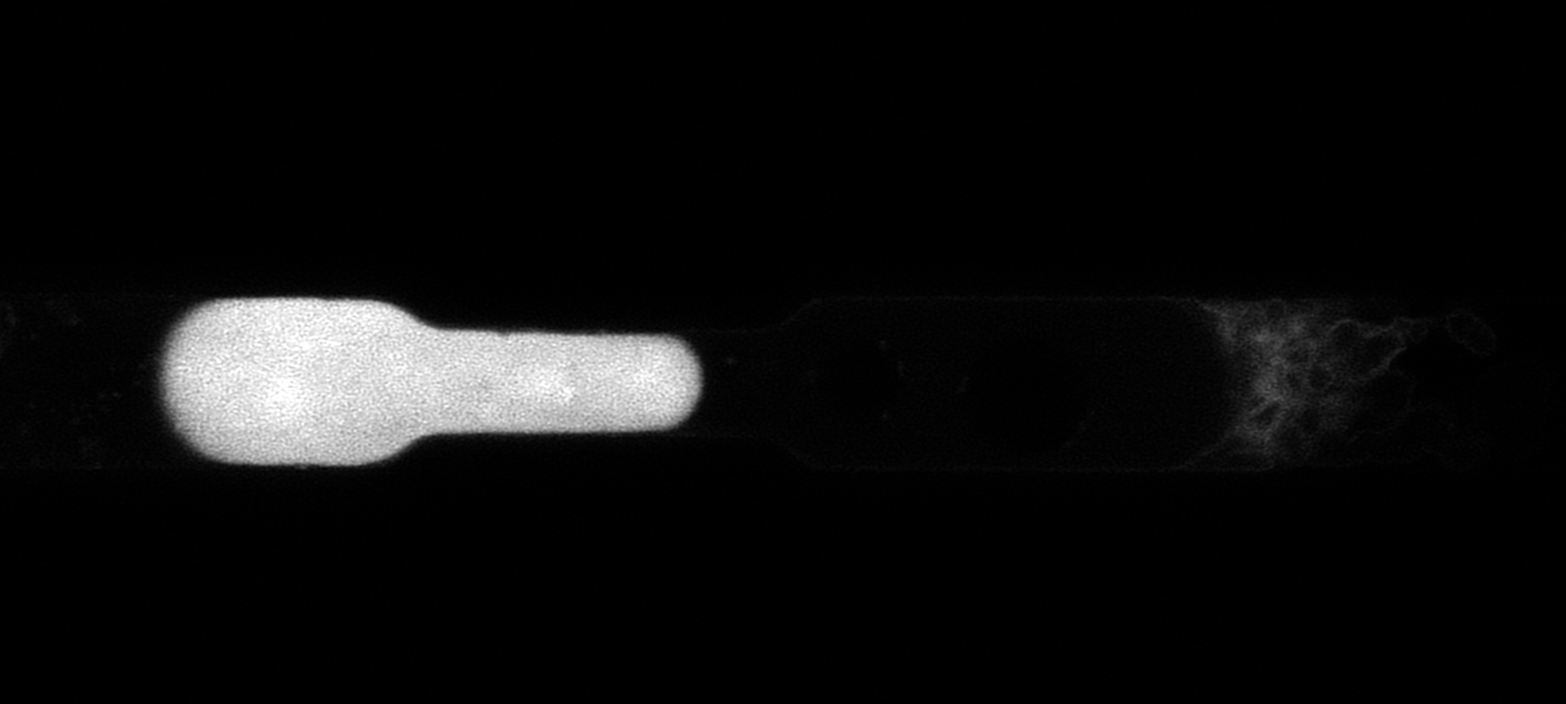

Supplement: Supplementary file 25 — Figure EV2 Source Data [file 44318_2025_566_MOESM25_ESM.zip › Fig EV2/Fig EV2D/DMSO_Before NE rupture.tif]

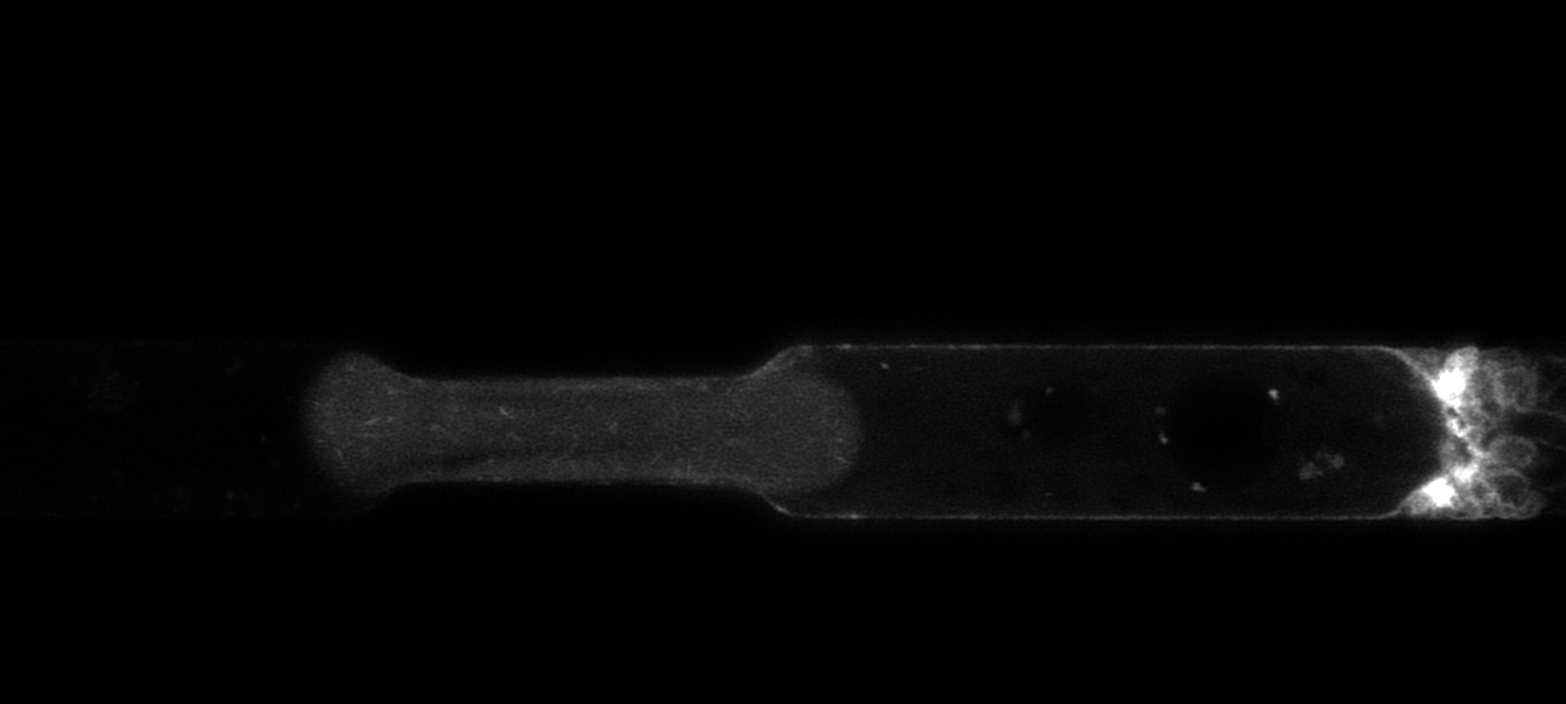

Supplement: Supplementary file 25 — Figure EV2 Source Data [file 44318_2025_566_MOESM25_ESM.zip › Fig EV2/Fig EV2D/DMSO_NE rupture.tif]

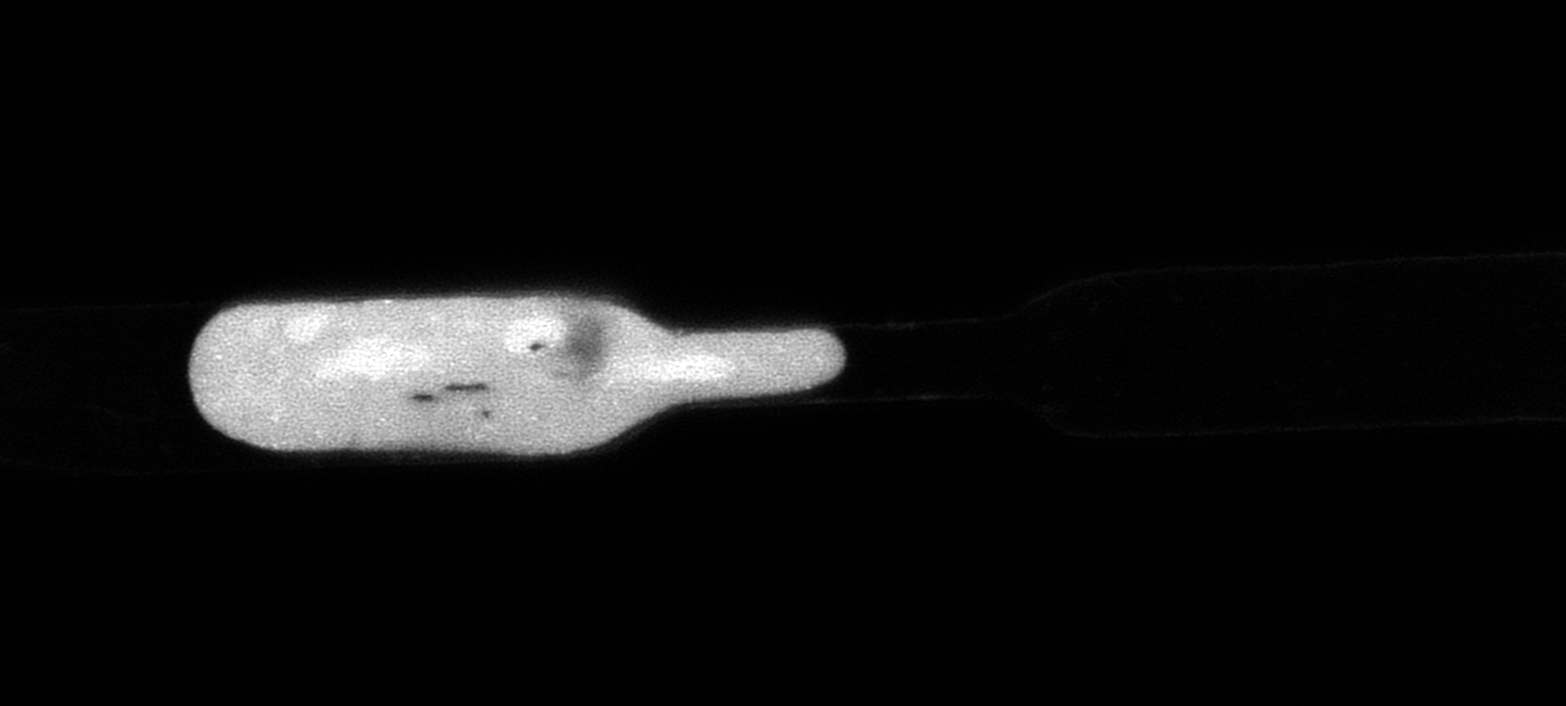

Supplement: Supplementary file 25 — Figure EV2 Source Data [file 44318_2025_566_MOESM25_ESM.zip › Fig EV2/Fig EV2D/Y27132_Before NE rupture.tif]

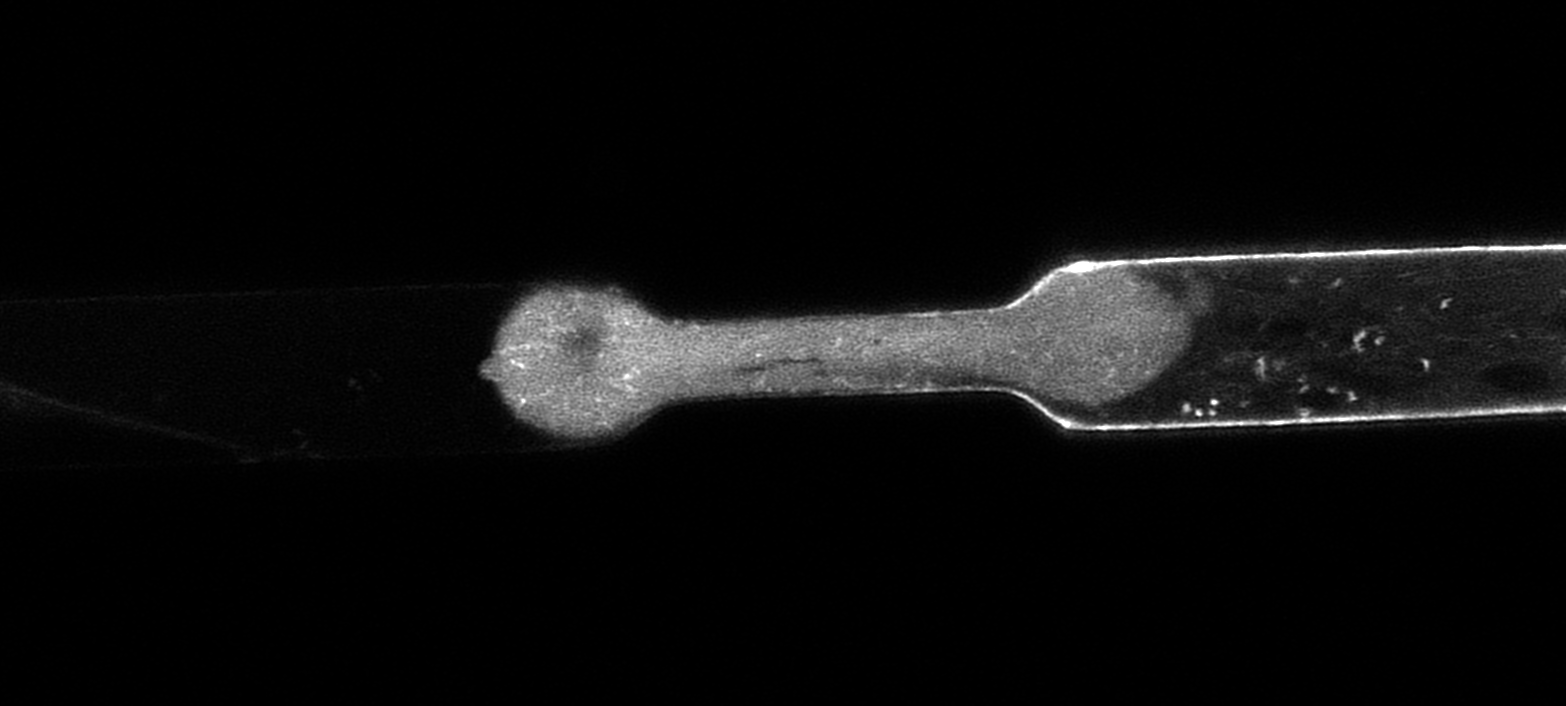

Supplement: Supplementary file 25 — Figure EV2 Source Data [file 44318_2025_566_MOESM25_ESM.zip › Fig EV2/Fig EV2D/Y27132_NE rupture.tif]

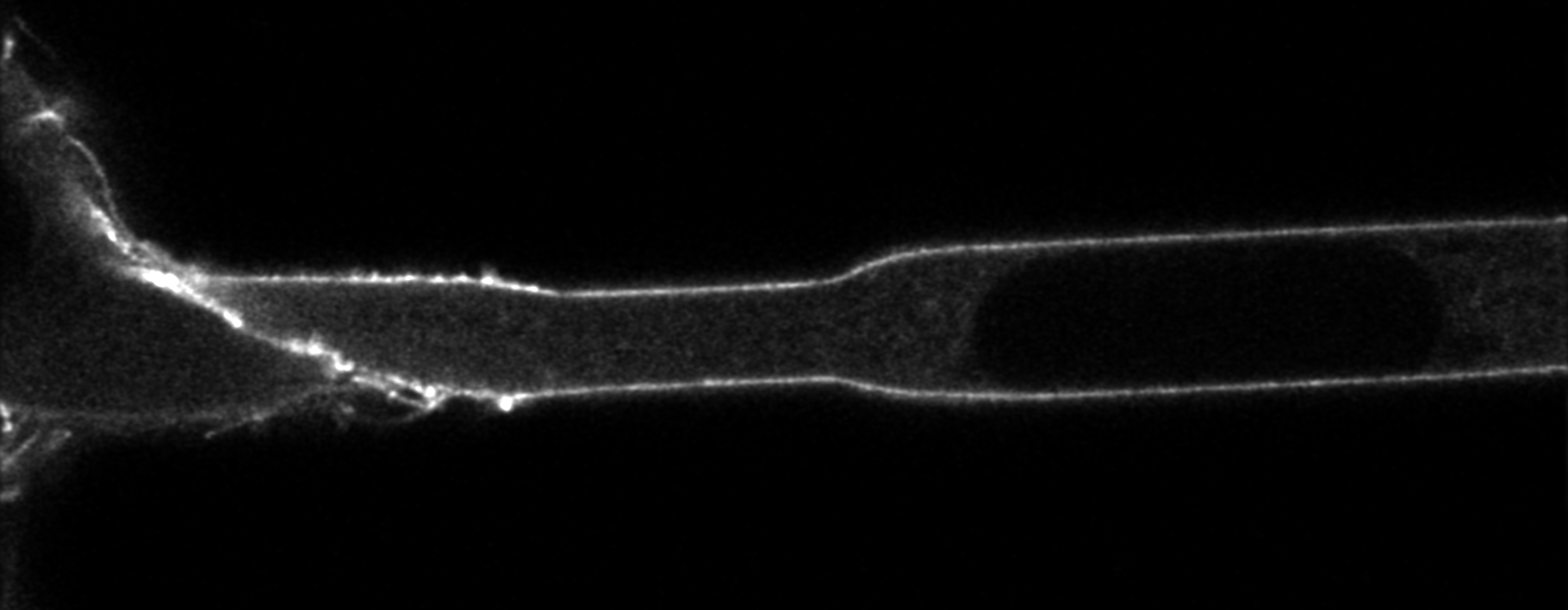

Supplement: Supplementary file 25 — Figure EV2 Source Data [file 44318_2025_566_MOESM25_ESM.zip › Fig EV2/Fig EV2F/FMNL2-GFP_After exit.tif]

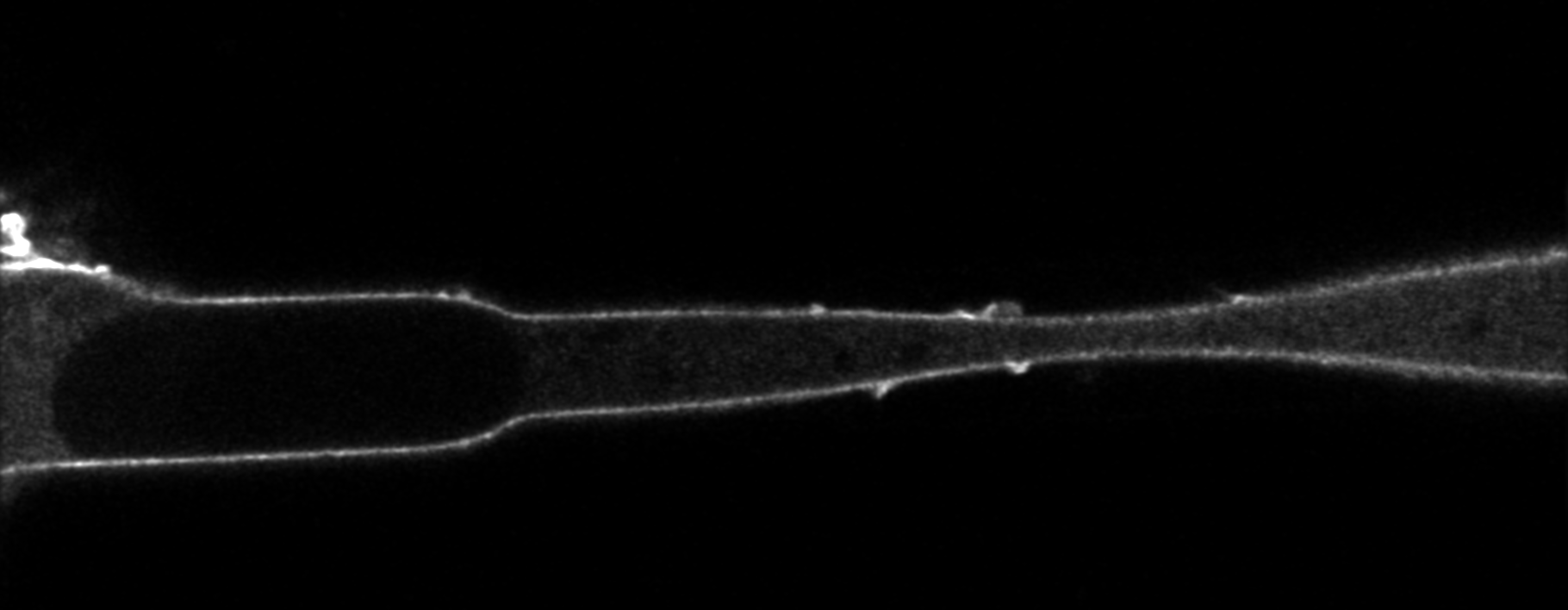

Supplement: Supplementary file 25 — Figure EV2 Source Data [file 44318_2025_566_MOESM25_ESM.zip › Fig EV2/Fig EV2F/FMNL2-GFP_Before NE rupture.tif]

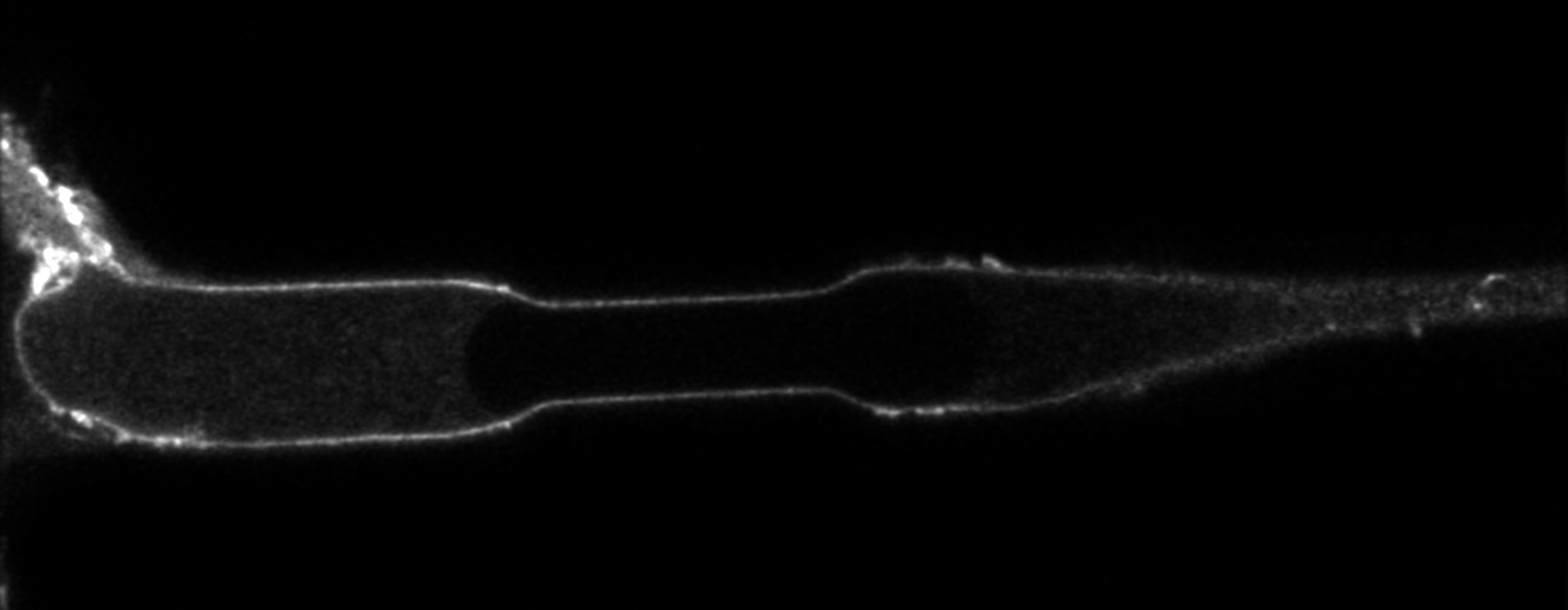

Supplement: Supplementary file 25 — Figure EV2 Source Data [file 44318_2025_566_MOESM25_ESM.zip › Fig EV2/Fig EV2F/FMNL2-GFP_NE rupture.tif]

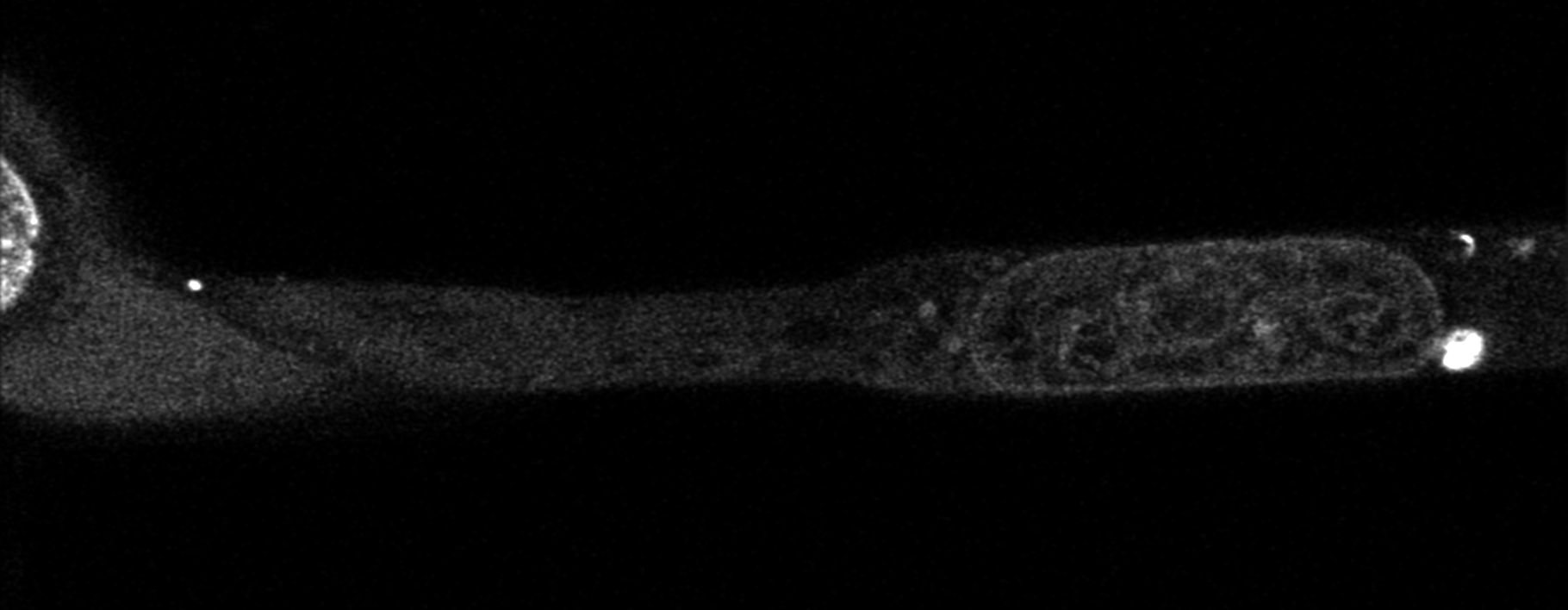

Supplement: Supplementary file 25 — Figure EV2 Source Data [file 44318_2025_566_MOESM25_ESM.zip › Fig EV2/Fig EV2F/icGAS-mCherry_After exit.tif]

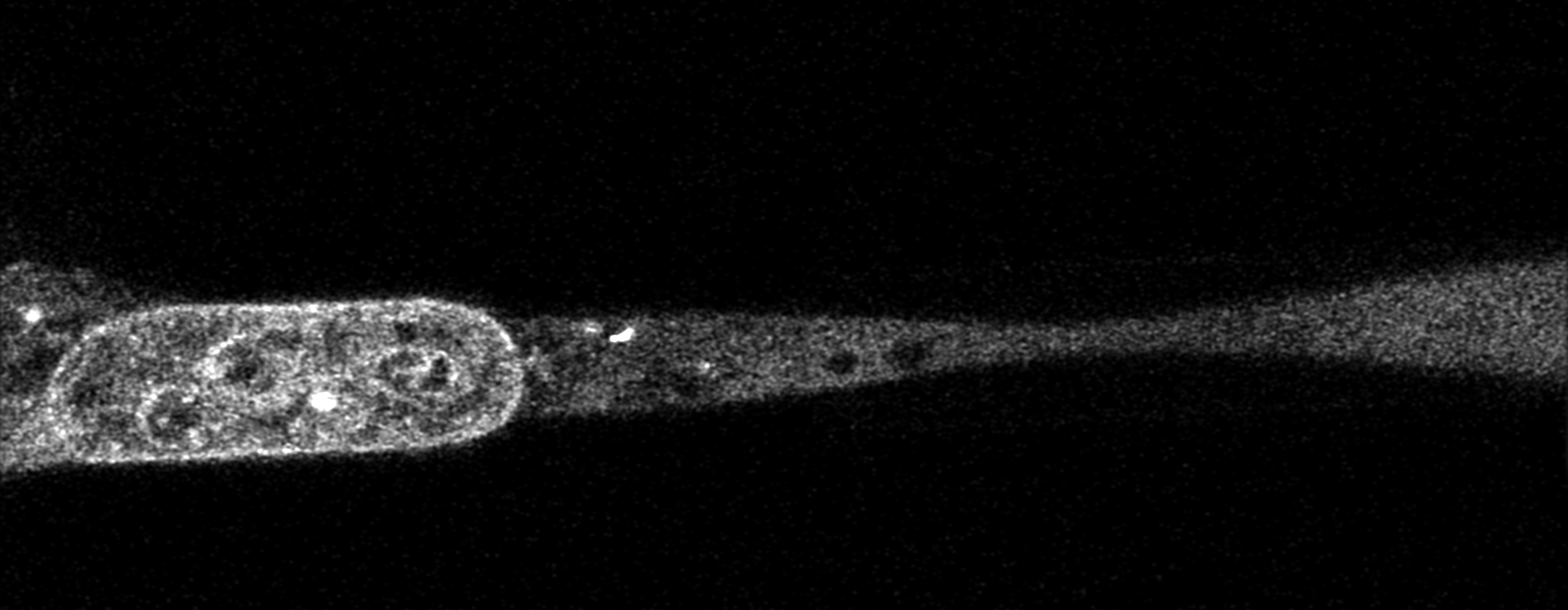

Supplement: Supplementary file 25 — Figure EV2 Source Data [file 44318_2025_566_MOESM25_ESM.zip › Fig EV2/Fig EV2F/icGAS-mCherry_Before NE rupture.tif]

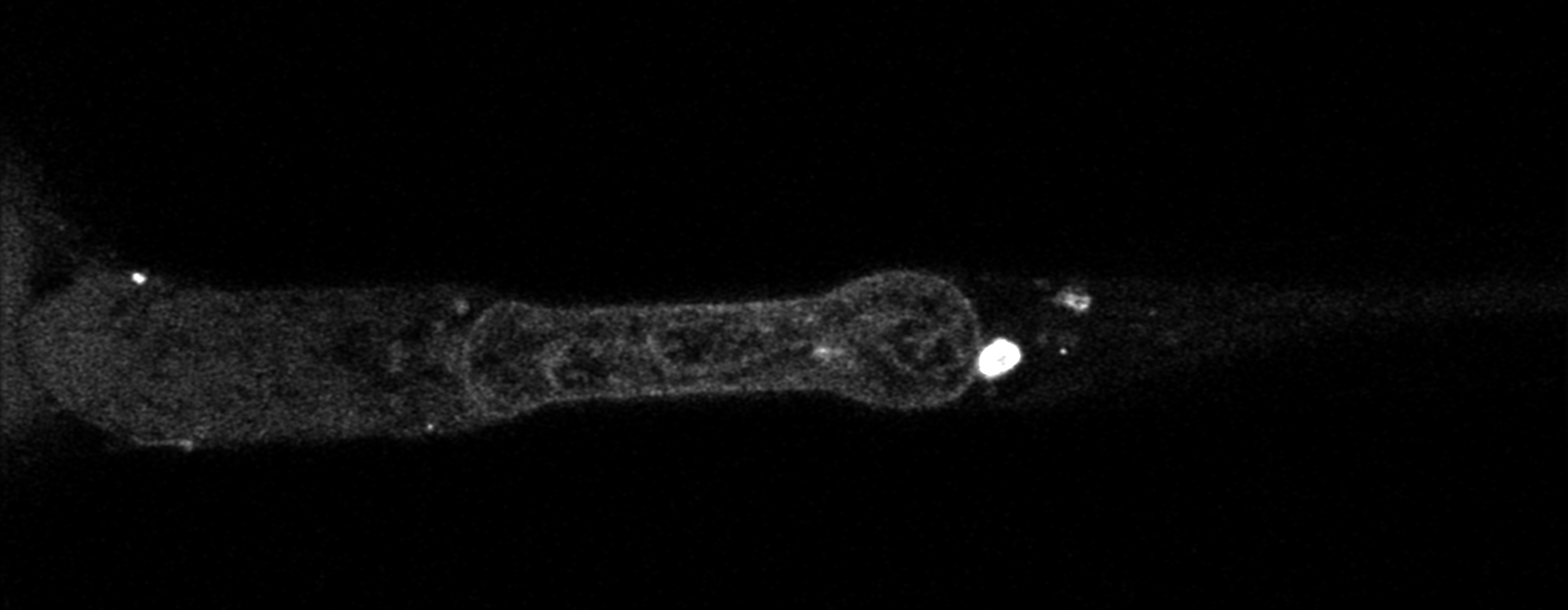

Supplement: Supplementary file 25 — Figure EV2 Source Data [file 44318_2025_566_MOESM25_ESM.zip › Fig EV2/Fig EV2F/icGAS-mCherry_NE rupture.tif]

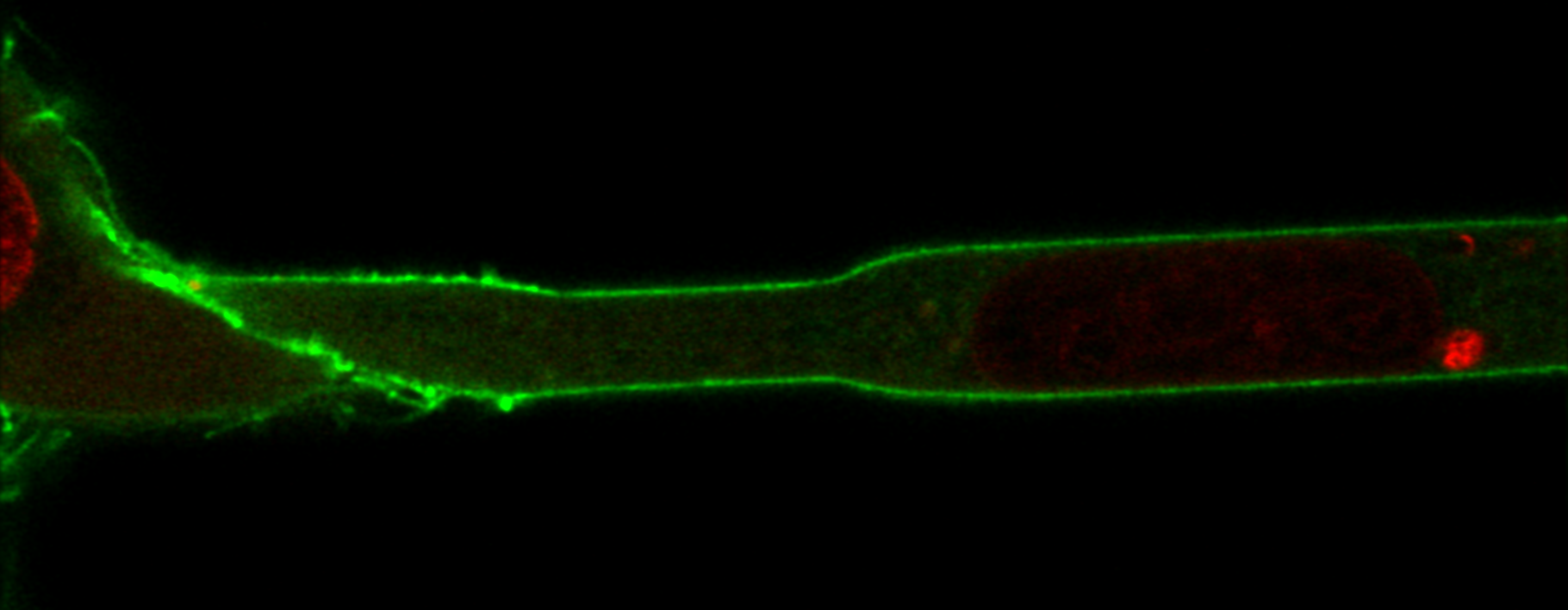

Supplement: Supplementary file 25 — Figure EV2 Source Data [file 44318_2025_566_MOESM25_ESM.zip › Fig EV2/Fig EV2F/Merge_after exit.tif]

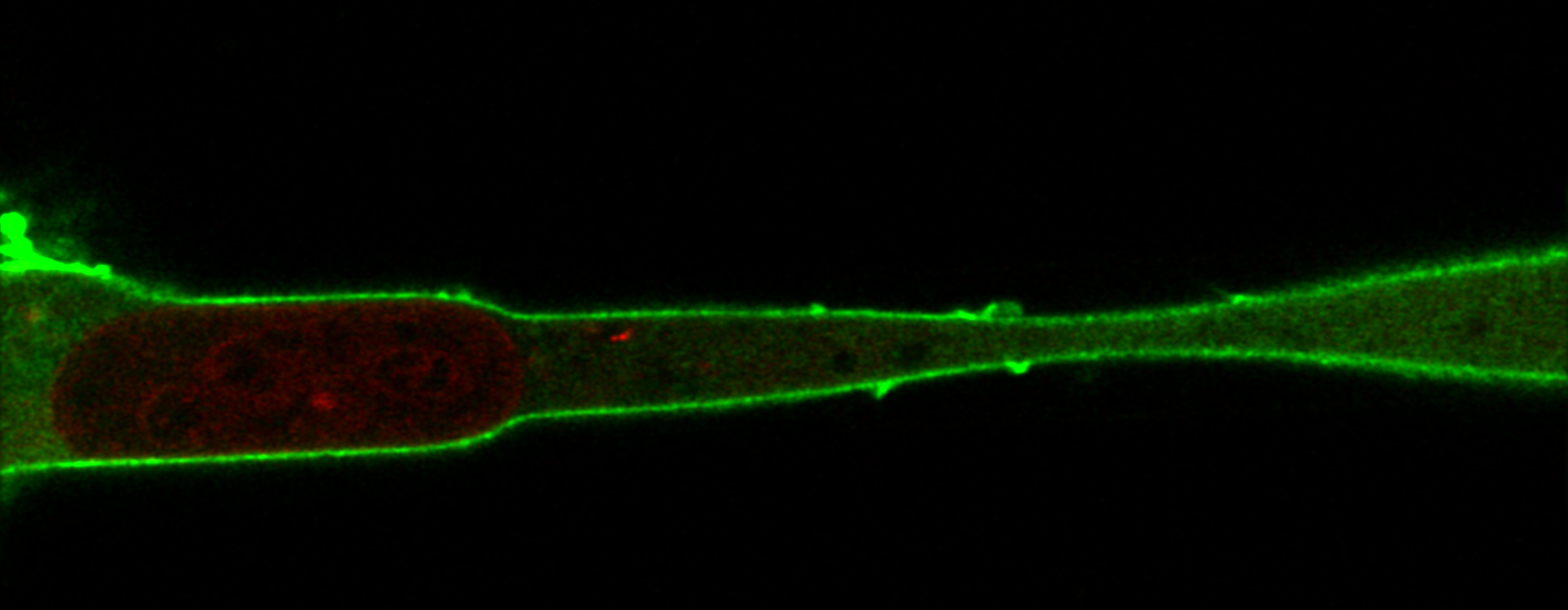

Supplement: Supplementary file 25 — Figure EV2 Source Data [file 44318_2025_566_MOESM25_ESM.zip › Fig EV2/Fig EV2F/Merge_Before NE rupture.tif]

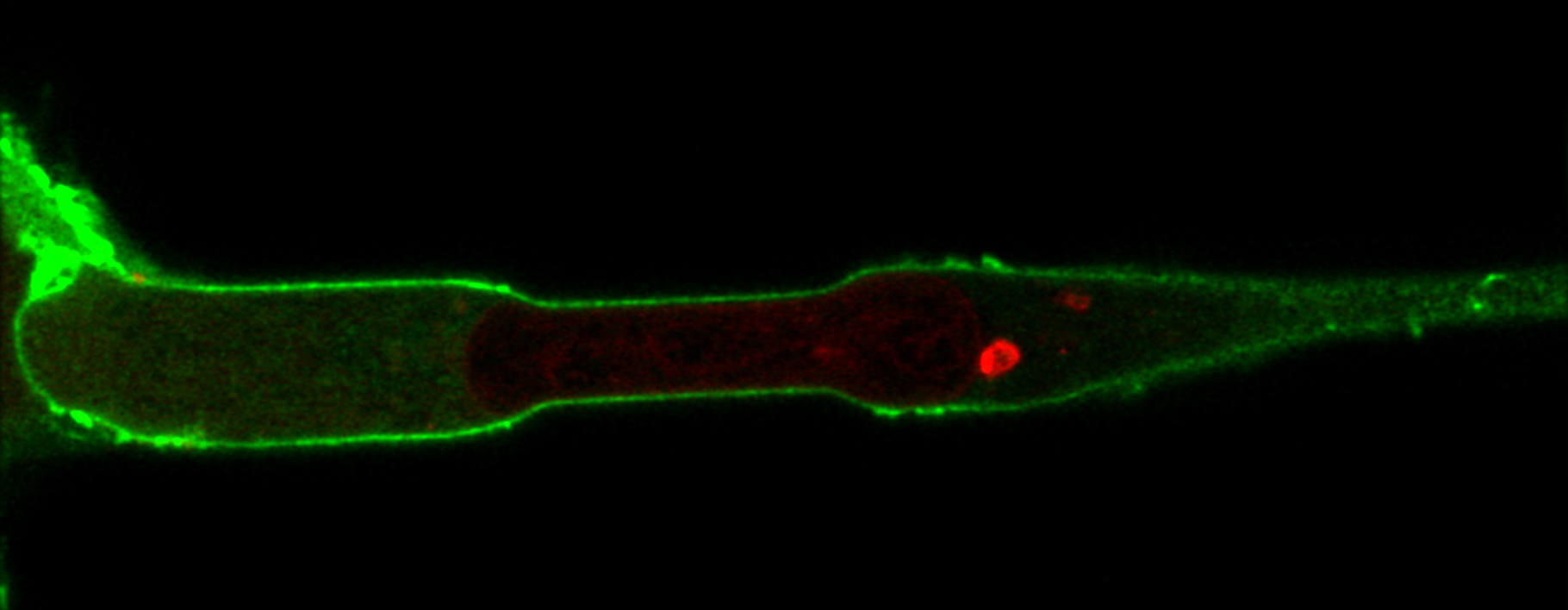

Supplement: Supplementary file 25 — Figure EV2 Source Data [file 44318_2025_566_MOESM25_ESM.zip › Fig EV2/Fig EV2F/Merge_NE rupture.tif]

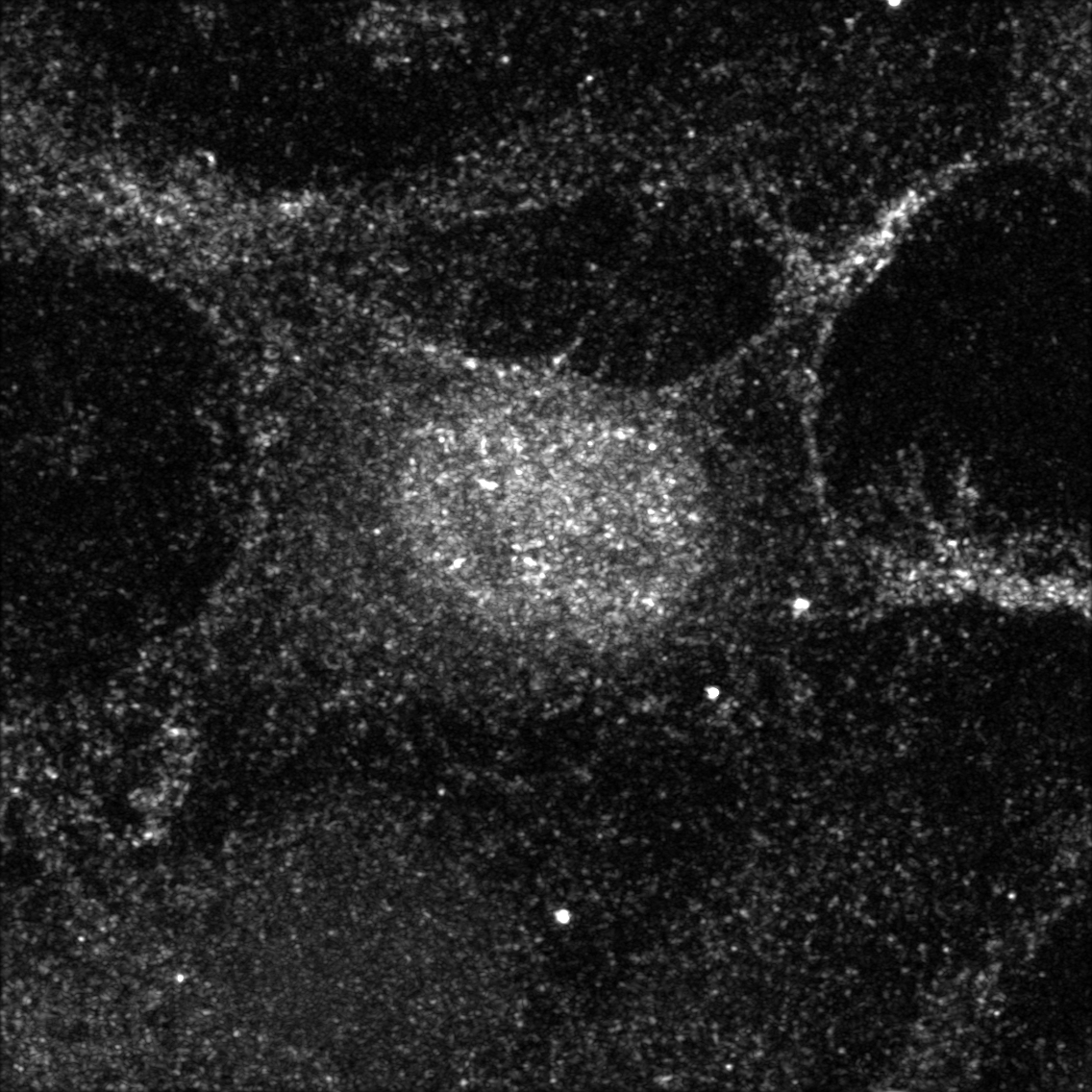

Supplement: Supplementary file 26 — Figure EV3 Source Data [file 44318_2025_566_MOESM26_ESM.zip › Fig EV3/Fig EV3A/Fig EV3A - NLS-Actin.tif]

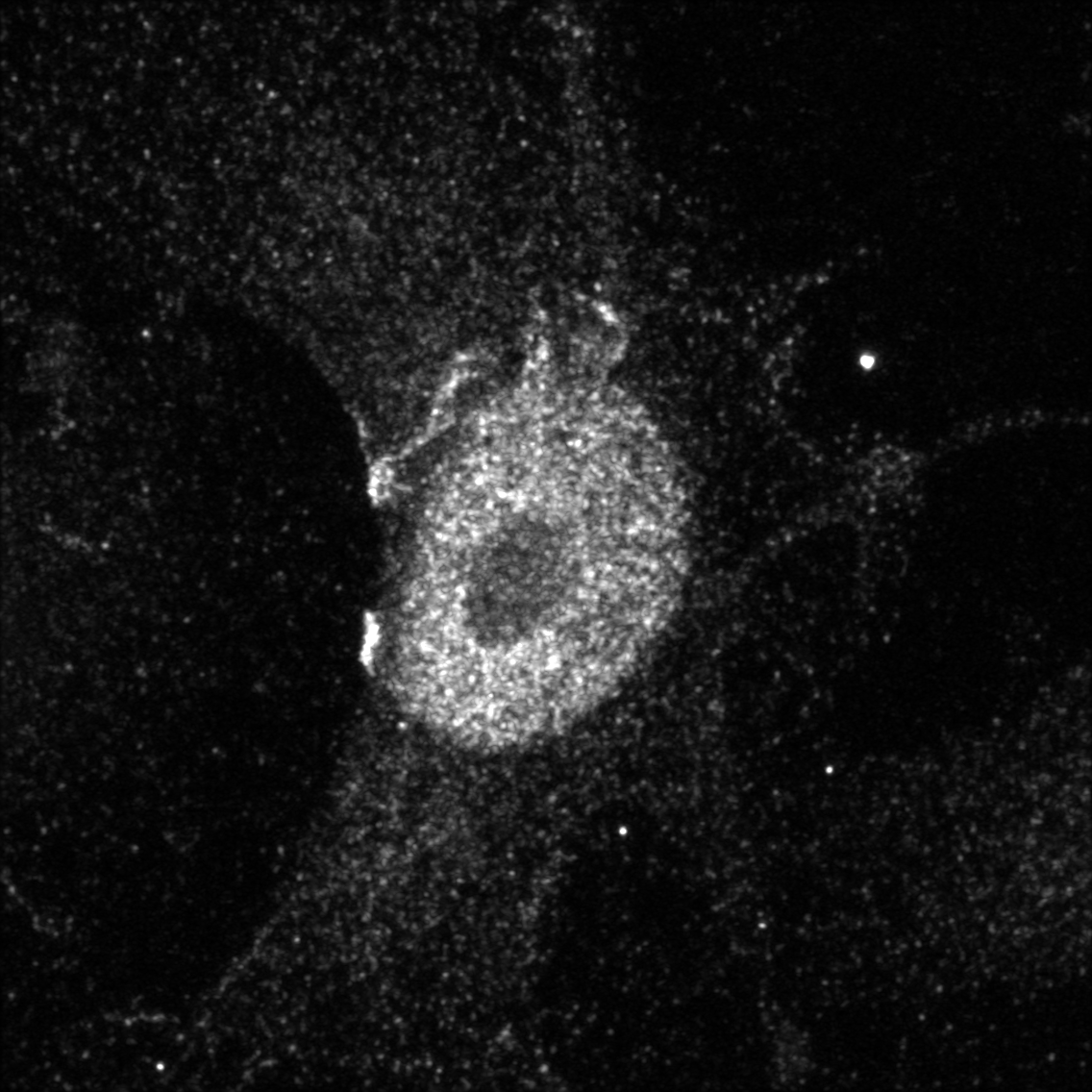

Supplement: Supplementary file 26 — Figure EV3 Source Data [file 44318_2025_566_MOESM26_ESM.zip › Fig EV3/Fig EV3A/Fig EV3A - R62D-NLS.tif]

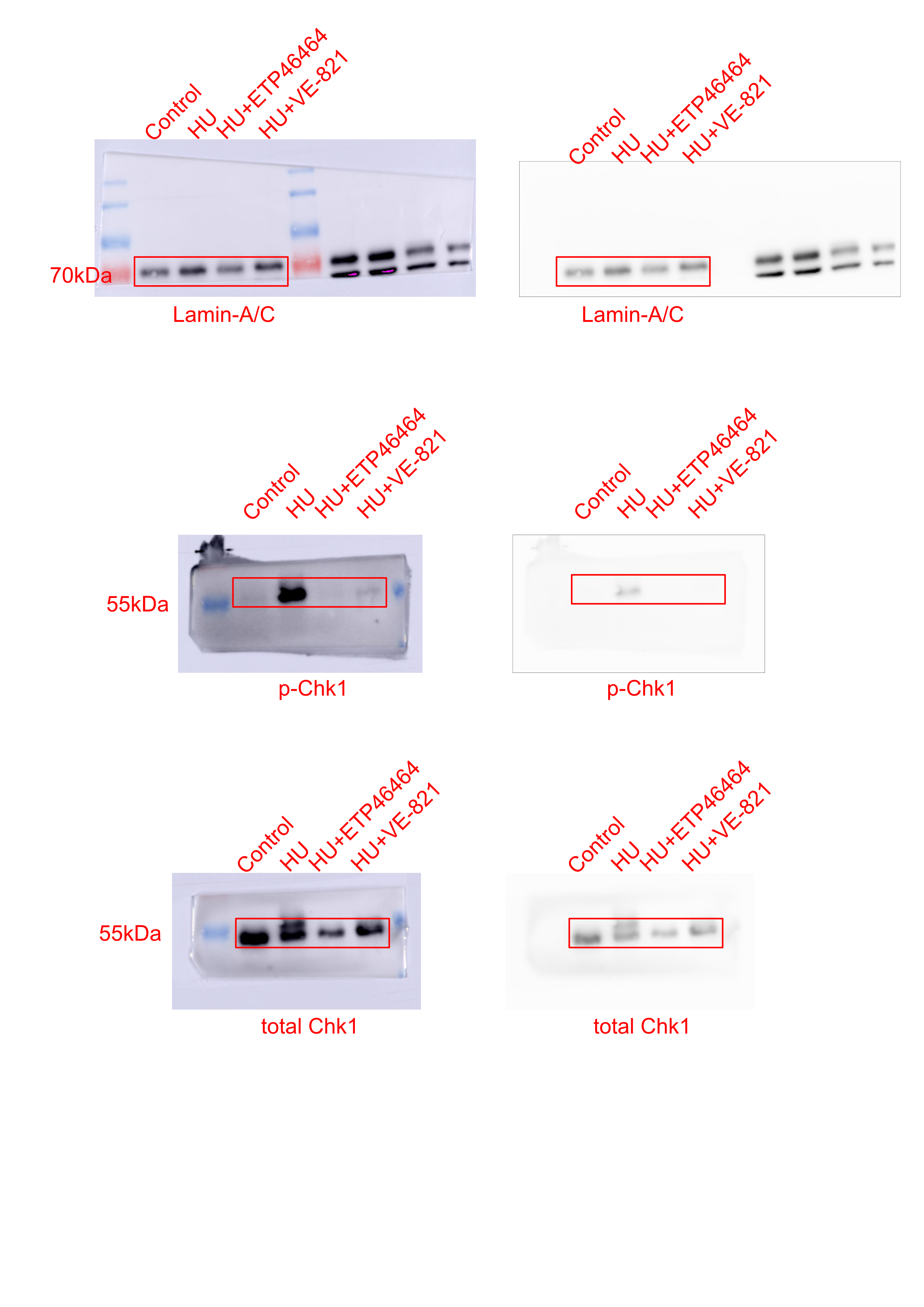

Supplement: Supplementary file 27 — Figure EV4 Source Data [file 44318_2025_566_MOESM27_ESM.zip › Fig EV4/Fig EV4A/ATR validation.tiff]

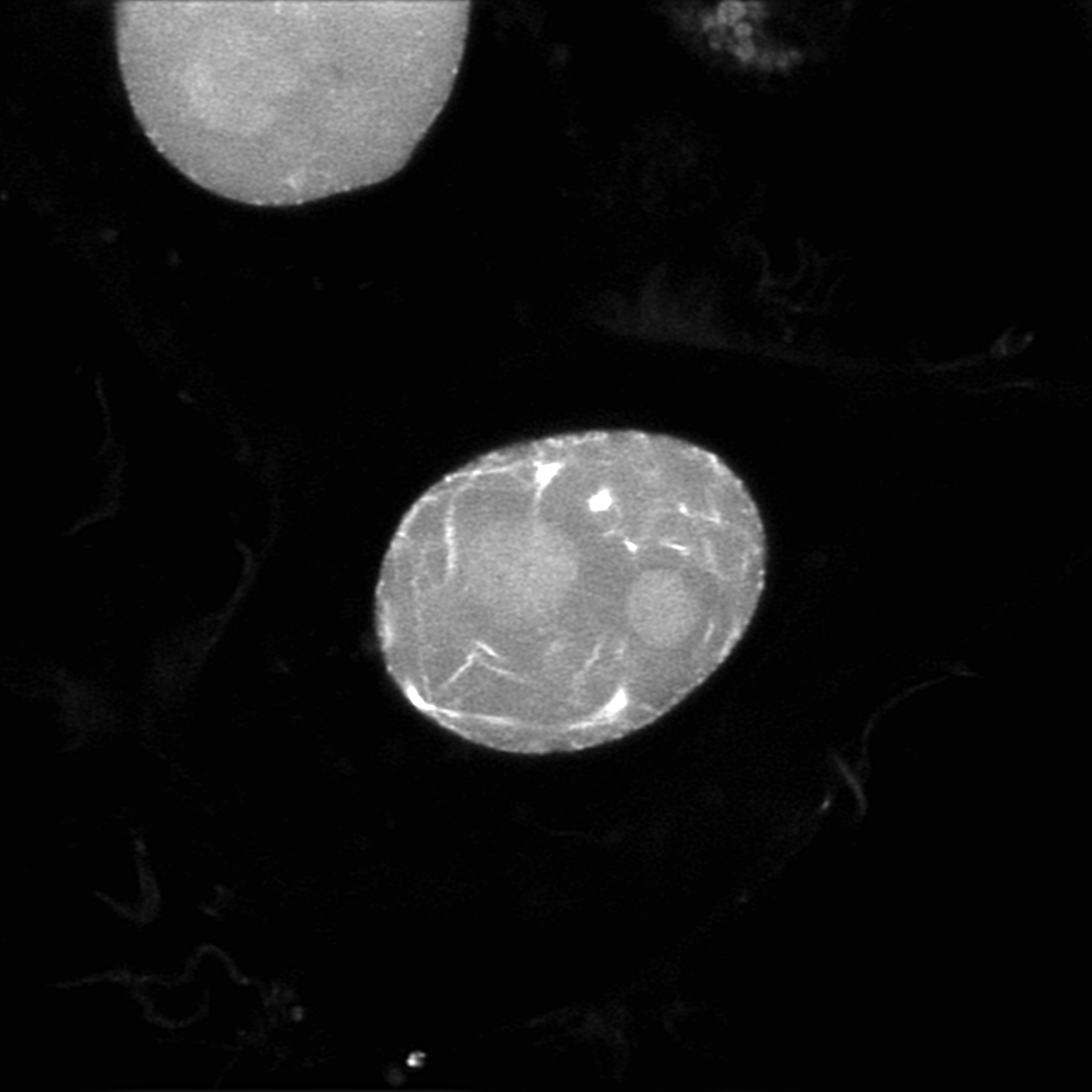

Supplement: Supplementary file 27 — Figure EV4 Source Data [file 44318_2025_566_MOESM27_ESM.zip › Fig EV4/Fig EV4C/Fig EV4C - DMSO.tif]

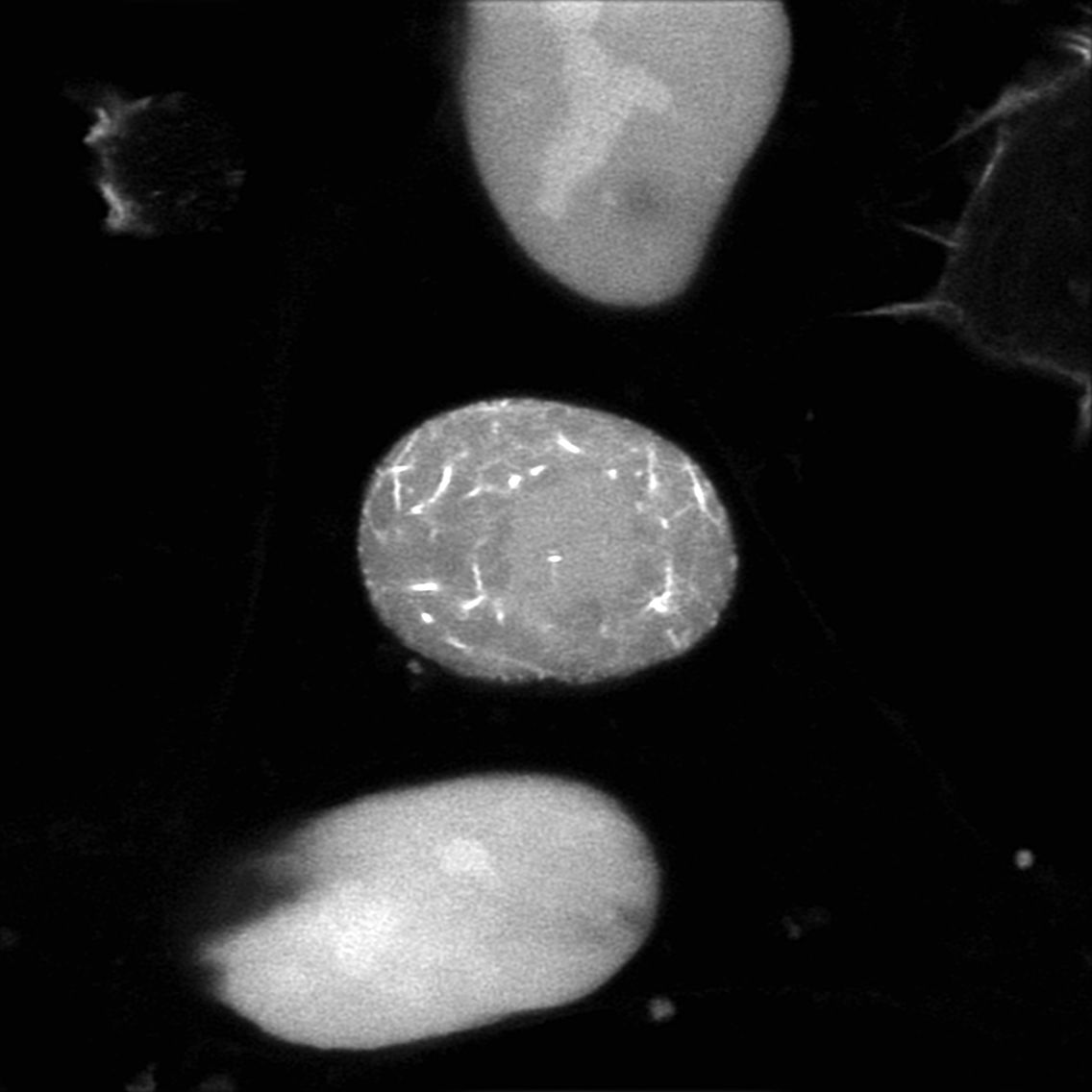

Supplement: Supplementary file 27 — Figure EV4 Source Data [file 44318_2025_566_MOESM27_ESM.zip › Fig EV4/Fig EV4C/Fig EV4C_Chk1i.tif]

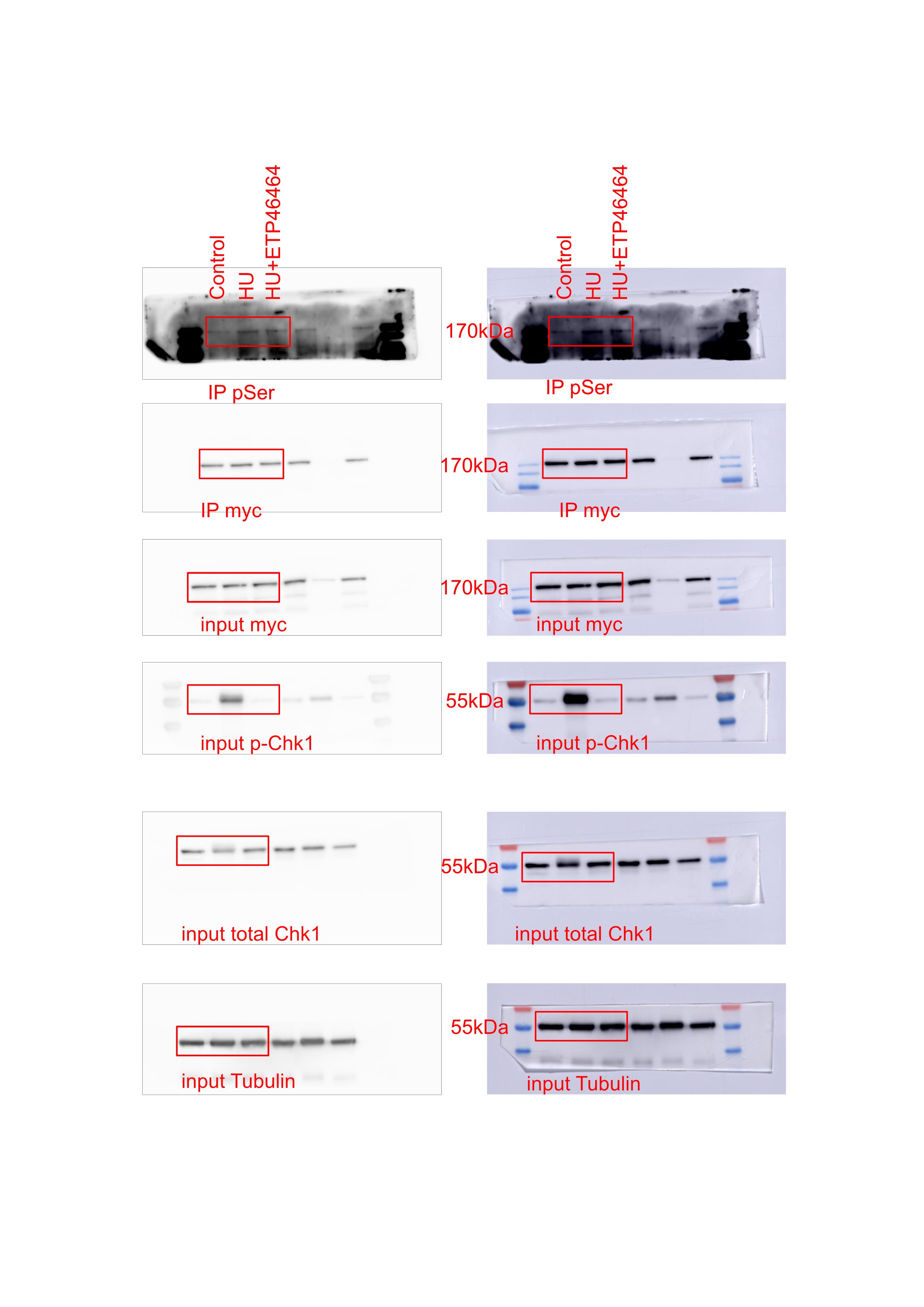

Supplement: Supplementary file 27 — Figure EV4 Source Data [file 44318_2025_566_MOESM27_ESM.zip › Fig EV4/Fig EV4E/Fig EV4E - Diaph3-WT.tiff]

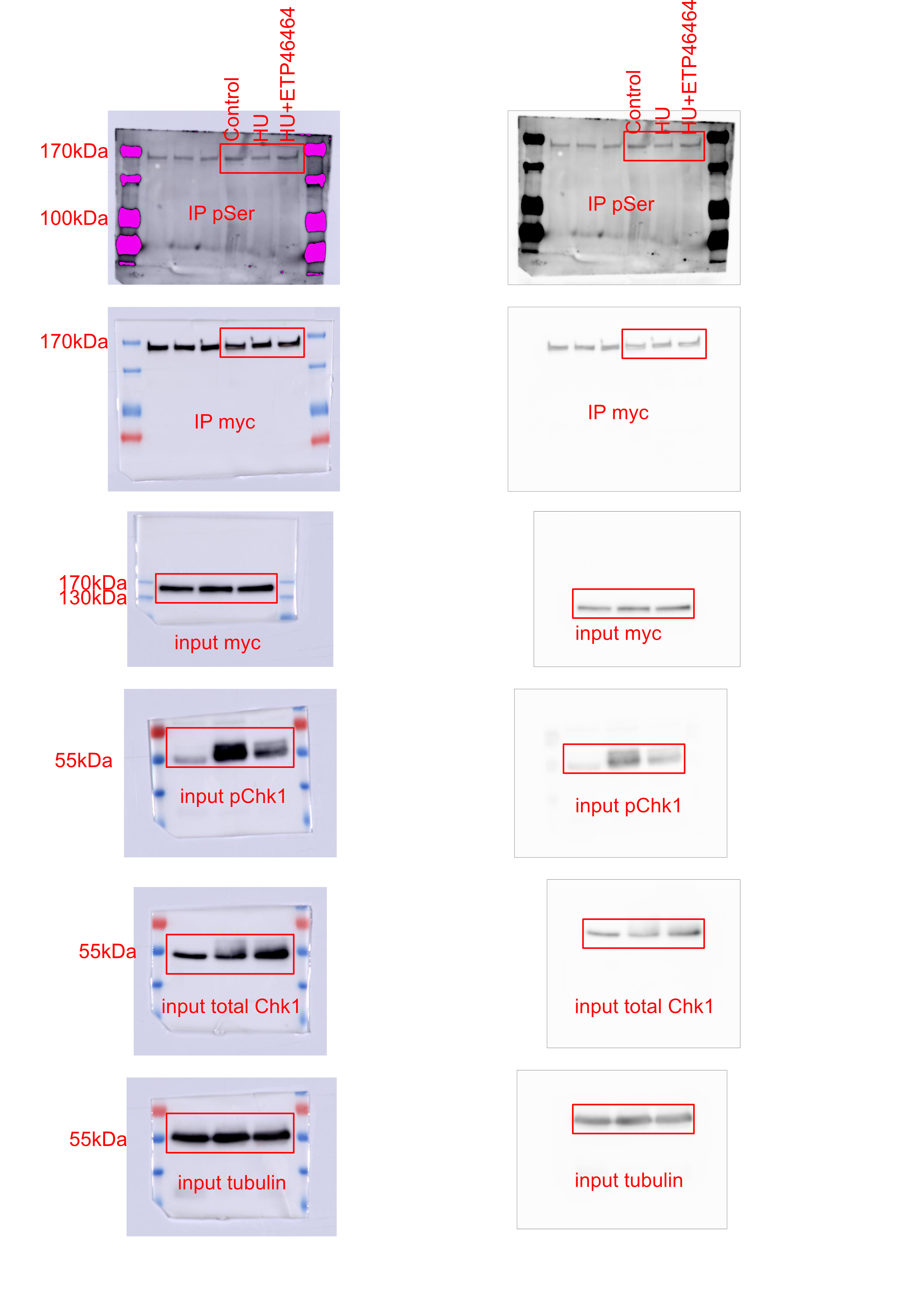

Supplement: Supplementary file 27 — Figure EV4 Source Data [file 44318_2025_566_MOESM27_ESM.zip › Fig EV4/Fig EV4G/Fig EV4G - Diaph3-S1072A.tiff]
