# Supplementary material for: Nuclear rupture in confined cell migration triggers nuclear actin polymerization to limit chromatin leakage
Source: EMBO J. 2025 Sep 22;44(21):6112–36. doi: 10.1038/s44318-025-00566-2 (PMC12583611; doi:10.1038/s44318-025-00566-2)
Supplement: Supplementary file 28 — Expanded View Figures [file 44318_2025_566_MOESM28_ESM.pdf]

Expanded View Figures

Figure EV1. Nuclear actin polymerization during confined cell migration is independent of the LINC complex or genetically encoded actin probes.

(**A**) Representative image sequences of representative HT1080-nAC-GFP cells expressing either mScarlet or the dominant-negative transmembrane domain of nesprin (DN.KASH-mScarlet), migrating through 3 µm microchannels. Actin is visualized via nAC-GFP. Scale bar, 5 µm. (**B**) Percentage of HT1080-nAC-GFP cells displaying nuclear F-actin formation following migration through 3 µm microchannels after co-expression of mScarlet or DN.KASH-mScarlet. Data shown as mean ± s.d. Three independent experiments per condition with $n \geq 6$ per experiment. Statistical analysis was performed with an unpaired parametric *t*-test. ns not significant. (**C**) Image sequences of an HT1080 cell stained with the live-cell actin dye FastActX-SPY650 and expressing NLS-BFP as a NE rupture marker migrating through a microchannel. Scale bar, 5 µm. Before NE rupture; corresponds to the time before a NE rupture event had occurred. NE rupture; corresponds to a frame in which a NE rupture event is detected via leakage of NLS-BFP. (**D**) Image sequences of an HT1080-nAC-GFP cell stained with the live-cell actin dye FastActX-SPY650 migrating through a microchannel. The white arrowhead indicates a nuclear actin filament co-labeled by nAC-GFP and FastActX. Before NE rupture; corresponds to the time before a NE rupture event had occurred. NE rupture; corresponds to a frame in which a NE rupture event is detected via leakage of nAC-GFP. Scale bar, 5 µm. Source data are available online for this figure.

 © The Author(s)

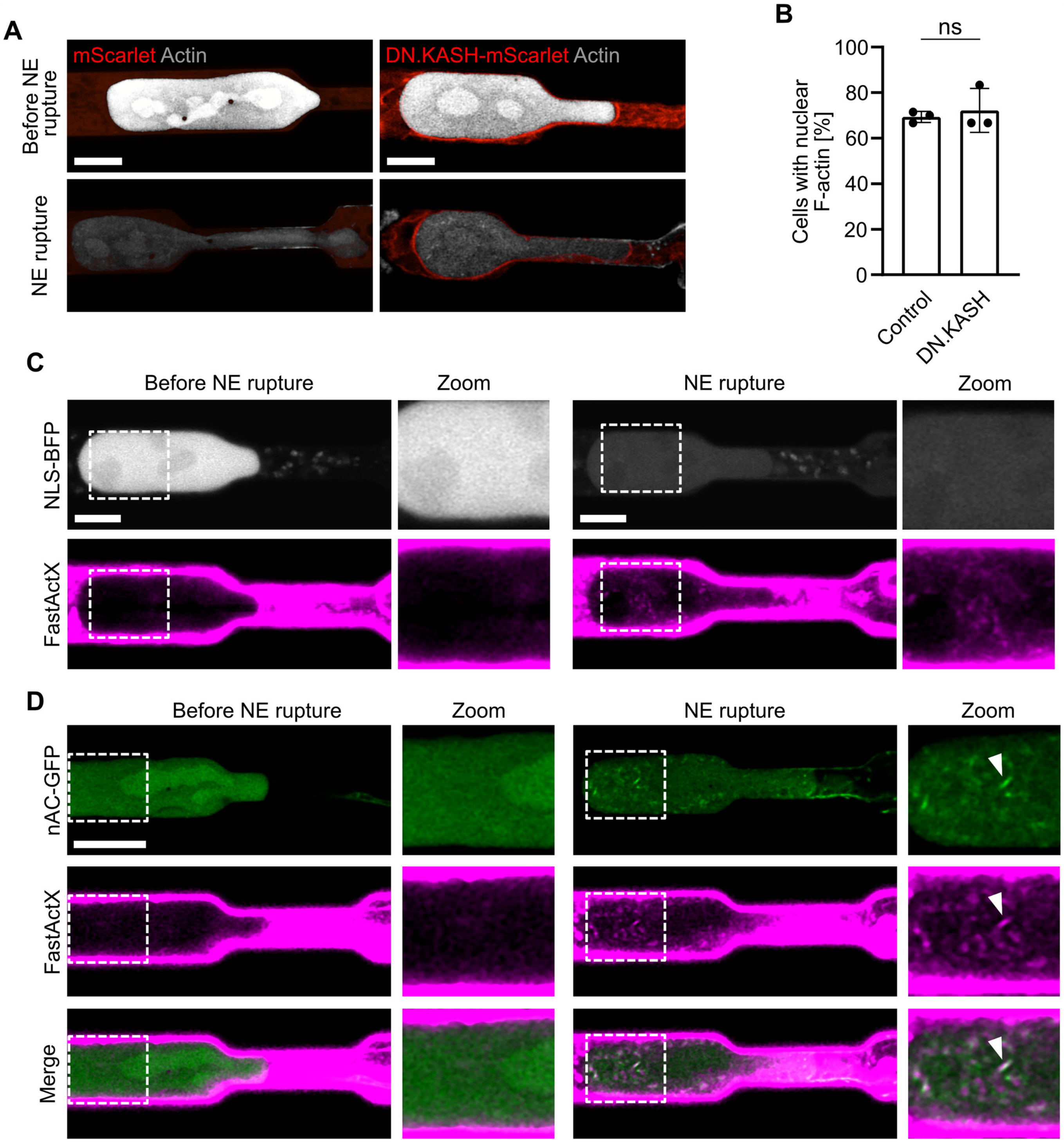
A
mScarlet Actin
DN.KASH-mScarlet Actin
Before NE rupture
NE rupture
B
ns
Cells with nuclear F-actin [%]
100
80
60
40
20
0
Control
DN.KASH
C
Before NE rupture
Zoom
NE rupture
Zoom
NLS-BFP
FastActX
D
Before NE rupture
Zoom
NE rupture
Zoom
nAC-GFP
FastActX
Merge

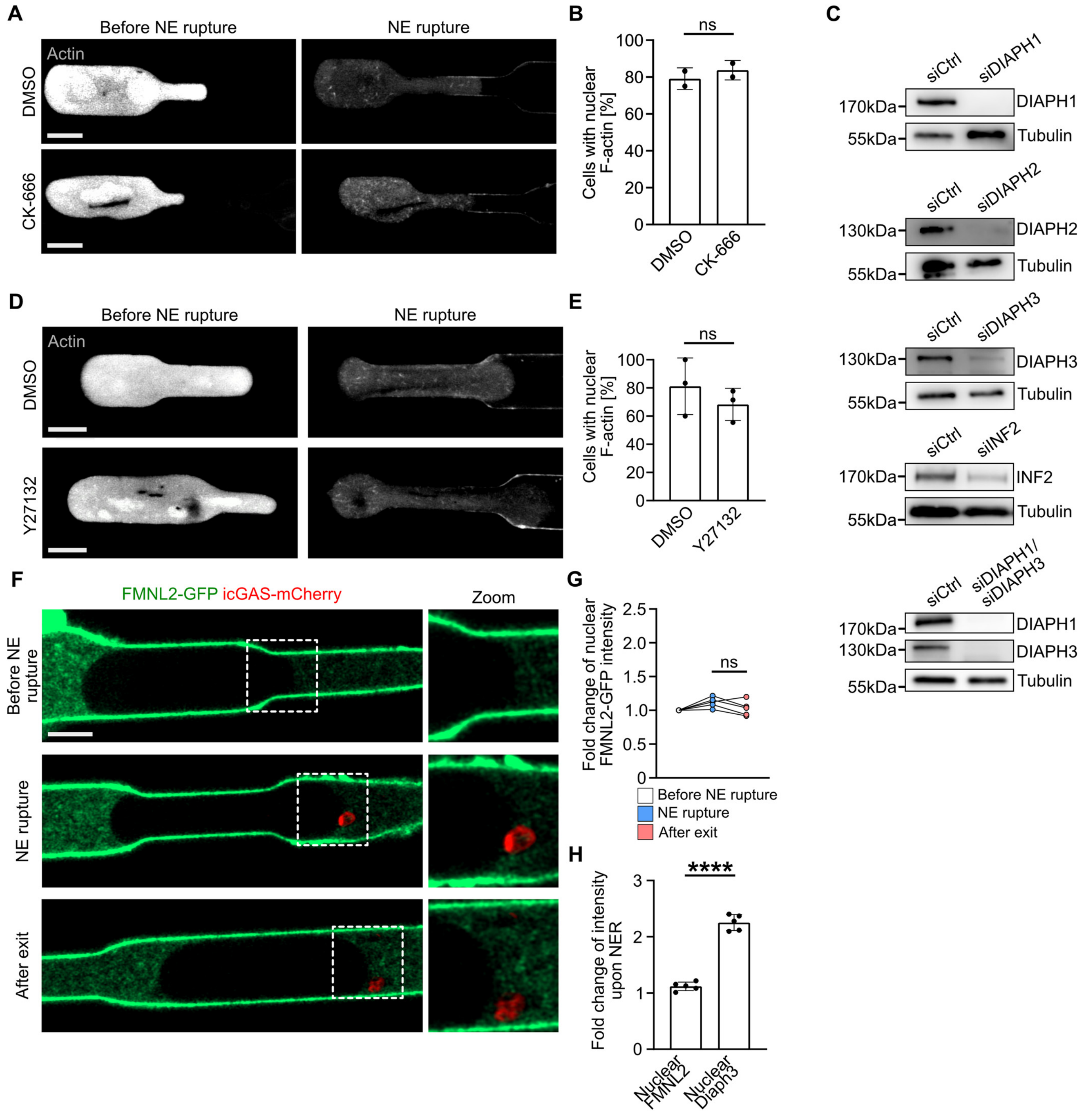
A
Before NE rupture
NE rupture
Actin
DMSO
CK-666
B
ns
Cells with nuclear F-actin [%]
100
80
60
40
20
0
DMSO
CK-666
C
siCtrl
siDIAPH1
170kDa
DIAPH1
55kDa
Tubulin
siCtrl
siDIAPH2
130kDa
DIAPH2
55kDa
Tubulin
siCtrl
siDIAPH3
130kDa
DIAPH3
55kDa
Tubulin
siCtrl
siINF2
170kDa
INF2
55kDa
Tubulin
siCtrl
siDIAPH1/ siDIAPH3
170kDa
DIAPH1
130kDa
DIAPH3
55kDa
Tubulin
D
Before NE rupture
NE rupture
Actin
DMSO
Y27132
E
ns
Cells with nuclear F-actin [%]
DMSO
Y27132
F
FMNL2-GFP icGAS-mCherry
Zoom
Before NE rupture
NE rupture
After exit
G
Fold change of nuclear FMNL2-GFP intensity
2.5
2.0
1.5
1.0
0.5
0
ns
Before NE rupture
NE rupture
After exit
H
****
Fold change of intensity upon NER
3
2
1
0
Nuclear FMNL2
Nuclear Diaph3

 © The Author(s)

Figure EV2. Nuclear actin polymerization upon NE rupture is mediated by DIAPH1 and DIAPH3.

(**A**) Image sequences of representative HT1080-nAC-GFP cells migrating through 3 µm microchannels, after being treated with either 0.01% DMSO or 100 µM of the Arp-2/3 inhibitor CK-666. Actin is visualized via nAC-GFP. Scale bar, 5 µm. (**B**) Percentage of HT1080-nAC-GFP displaying nuclear F-actin structures after treatment with either 0.01% DMSO or 100 µM CK-666. Data shown as mean ± s.d. Two independent experiments per condition. $n \geq 8$ cells per independent experiment. (**C**) Representative immunoblots for the silencing efficiency of DIAPH1 (siDIAPH1), DIAPH2 (siDIAPH2), DIAPH3 (siDIAPH3), DIAPH1 and DIAPH3 (siDIAPH1/siDIAPH3), and INF2 (siINF2). Tubulin serves as a loading control. (**D**) Image sequences of HT1080-nAC-GFP cells that were treated with 0.01% DMSO or ROCK inhibitor 10 µM Y27132 before and during NE rupture. Actin is visualized via nAC-GFP. Scale bar, 5 µm. (**E**) Percentage of cells displaying nuclear F-actin upon NE rupture after being treated with either DMSO or Y27132. Data shown as mean ± s.d. Three independent experiments per condition with $n \geq 6$ cells per experiment. Statistical analysis was performed with an unpaired parametric *t*-test. (**F**) Image sequences of a representative HT1080-icGAS-mCherry cell that expresses FMNL2-GFP migrating through a microchannel. Before NE rupture; corresponds to the time before a NE rupture event had occurred. NE rupture; corresponds to the first frame in which a NE rupture event is detected via accumulation of icGAS-mCherry. After exit; corresponds to the time point at which the nucleus has exited from the narrow part of the microchannel. Scale bar, 5 µm. (**G**) Quantification of nuclear FMNL2-GFP intensity before NE rupture, during NE rupture and after exit. Data shown as individual values from $n = 5$ cells. Statistical analysis was performed by a paired parametric *t*-test. (**H**) Fold change of nuclear FMNL2-GFP and nuclear GFP-Diaph3 mean fluorescence intensities upon NE rupture. Data shown as individual values from $n = 5$ cells. Statistical analysis was performed by an unpaired parametric *t*-test. Exact *P* value: 3.0×10^{-7}. *****P* < 0.0001 and ns not significant. Source data are available online for this figure.

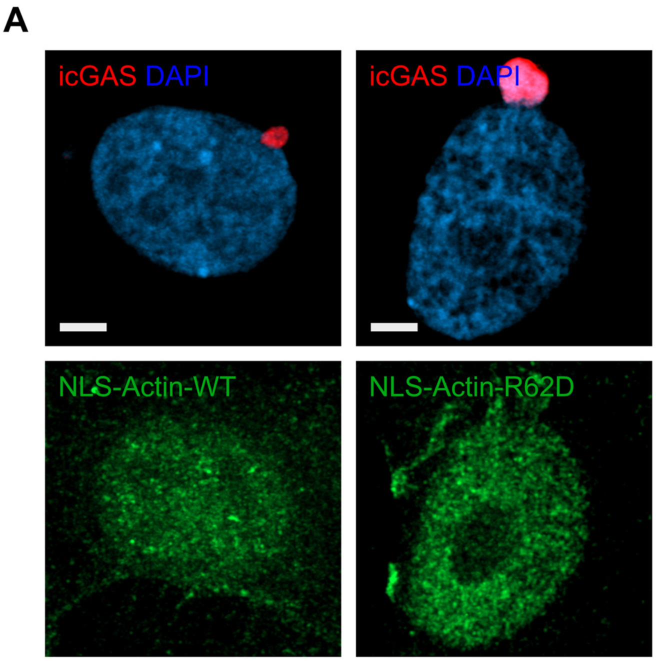

Figure EV3. Nuclear actin polymerization maintains nuclear integrity during confined cell migration.

(**A**) Representative immunofluorescence images of HT1080 cells stably expressing icGAS-mCherry (red) that transiently express either NLS-Actin-WT or NLS-Actin-R62D (green). These images were reused from Fig. 4D to show the expression of NLS-actin-WT or NLS-actin-R62D in these examples. Scale bar, 5 µm. Source data are available online for this figure.

 © The Author(s)

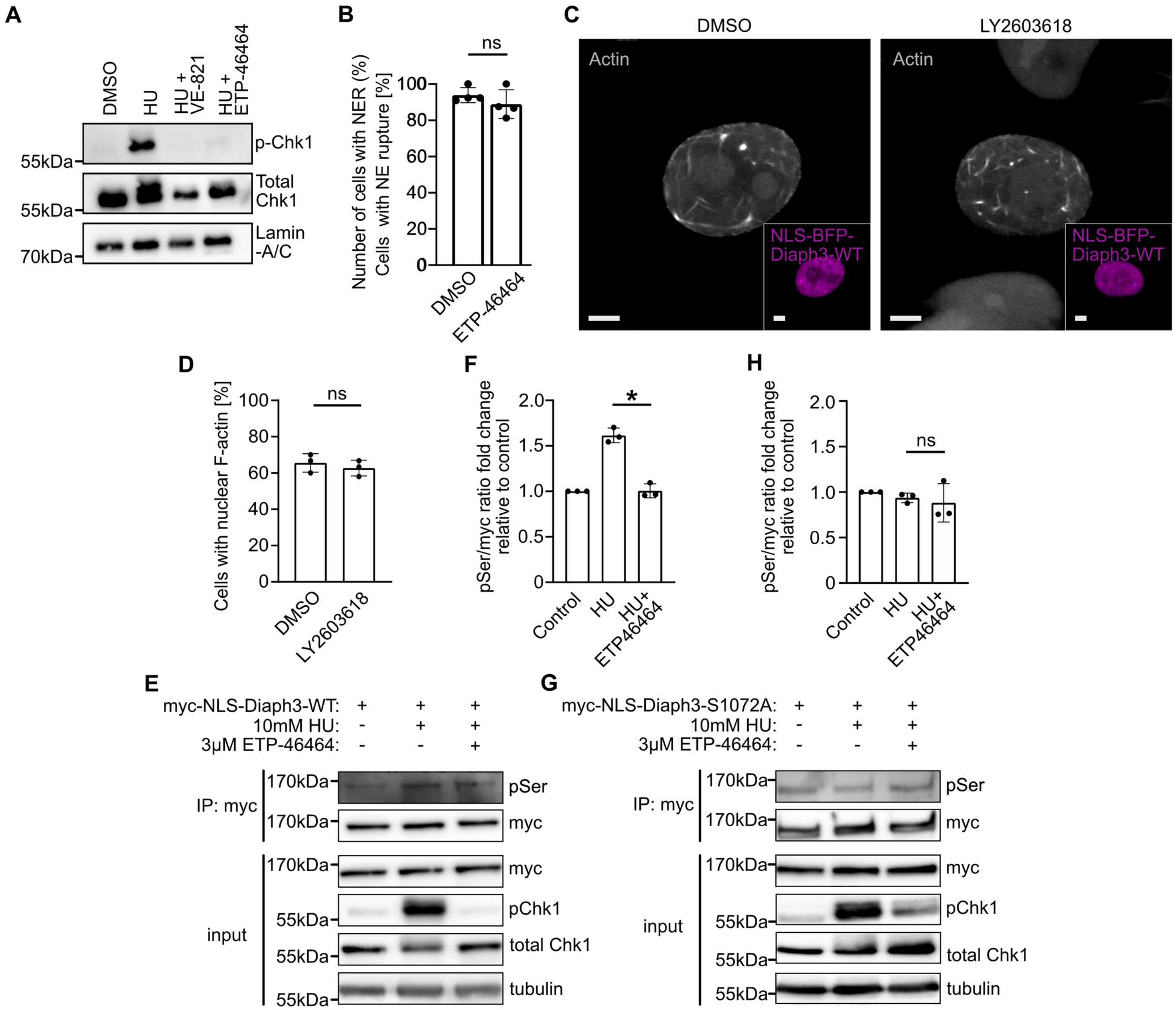

Figure EV4. ATR is necessary for nuclear actin polymerization upon NE rupture.

(**A**) Representative immunoblot of phosphorylated Chk1 (pChk1) (stripped and reprobed for total-Chk1) for the efficiency of ATR inhibitors ETP-46464 and VE-821. Loading control Lamin-A/C. (**B**) Percentage of HT1080 cells undergoing NE rupture after being treated with siCtrl or siATR. Data shown as mean ± s.d. Four independent experiments per condition with $n \geq 8$ cells per experiment. Statistical analysis was performed with the Mann–Whitney test. ns not significant. (**C**) Representative images of HT1080-nAC-GFP (white) that transiently express NLS-BFP-Diaph3-wt in the presence or absence of LY2603618, with bottom right images showing nuclear expression of this construct (magenta); Scale bar, 4 μm. (**D**) Percentage of HT1080-nAC-GFP cells transiently expressing NLS-Diaph3-wt that display nuclear F-actin formation after being treated with either 0.01% DMSO or 1 μM LY2603618 for 16 h. Data shown as mean ± s.d. Three independent experiments per condition with $n = 30$ cells per experiment. Statistical analysis between DMSO and LY2603618 treatment after expression of NLS-BFP-Diaph3-WT was performed by an unpaired parametric *t*-test. (**E**) HT1080-WT cells were transfected with myc-NLS-Diaph-WT, and cells were treated with Hydroxyurea (HU) in the presence or absence of ETP-46464. Immunoprecipitation was carried out by myc-agarose beads. The amount of phosphorylated serine residues was determined via western blot and detected with a general anti-phosphoserine antibody (pSer) (stripped and reprobed for myc). pChk1 (stripped and reprobed for total-Chk1 and Tubulin) was used as a marker for ATR activation and to monitor the activity of ETP-46464. (**F**) Quantification of the phosphorylation of serine residues of myc-NLS-BFP-Diaph3-WT upon HU treatment in the presence or absence of ETP-46464. Fold change of the pSer/myc ratio was calculated. Data shown as mean ± s.d. Three independent experiments per condition. Statistical analysis was performed with a paired parametric *t*-test. Exact *P* value: 0.01948. (**G**) HT1080-WT cells were transfected with myc-NLS-Diaph-S1072A, and cells were treated with HU in the presence or absence of ETP-46464. Immunoprecipitation was carried out by myc-agarose beads. The amount of phosphorylated serine residues was determined via western blot and detected with a general anti-phosphoserine antibody (pSer) (stripped and reprobed for myc). pChk1 (stripped and reprobed for total-Chk1 and Tubulin) was used as a marker for ATR activation and to monitor the activity of ETP-46464. (**H**) Quantification of the phosphorylation of serine residues of myc-NLS-BFP-Diaph3-S0172A upon HU treatment in the presence or absence of ETP-46464. Fold change of the pSer/myc ratio was calculated. Data shown as mean ± s.d. Three independent experiments per condition. Statistical analysis was performed with a paired parametric *t*-test. $^{*}P < 0.05$ and ns was not significant. Source data are available online for this figure.
